# Supplementary material for: The incidence of candidate binding sites for β-arrestin in Drosophila neuropeptide GPCRs
Source: PLoS One. 2022 Nov 1;17(11):e0275410. doi: 10.1371/journal.pone.0275410 (PMC9624432; doi:10.1371/journal.pone.0275410)
Supplement: S1 Fig — Drosophila Rhodopsin-like GPCRs with BBS-like sequences in ICL2 (bold, italicized) following the conserved Pro (capitalized). No other Rhodopsin-like GPCRs had a BBS-like sequence in either D. melanogaster or D. virilis. (PDF) [file pone.0275410.s001.pdf]

S1 Figure. *Drosophila* Rhodopsin-like GPCRs with BBS-like sequences in ICL2 following the conserved P (capitalized)

No other Rhodopsin-like GPCRs had a BBS-like sequence in either *D. melanogaster* or *D. virilis*

AstA R1

M(180) drflavvh**P**v***tsmslrt***ernat
V(200) drflavvh**P**v***tsmslrt***ernat

CCKL R 17D1

M(272) eryyaich**P**lr***srtwqt***inh
V(136) eryyaich**P**lr***srtwqt***inh

CCKL R 17D3

M(208) eryyaich**P**lr***srswqt***ish
V(212) eryyaich**P**lr***srswqt***is

PK2 R1

M(205) eryiaich**P**frqh***tmskls***rai
V(208) eryiaich**P**frqh***tmskls***rai

PK2 R2

M(164) eryiaich**P**frqh***tmskls***rav
V(171) eryiaich**P**frqh***tmskls***rai
