# Supplementary material for: The incidence of candidate binding sites for β-arrestin in Drosophila neuropeptide GPCRs
Source: PLoS One. 2022 Nov 1;17(11):e0275410. doi: 10.1371/journal.pone.0275410 (PMC9624432; doi:10.1371/journal.pone.0275410)
Supplement: S2 Fig — Drosophila Rhodopsin-like GPCRs that do not contain a conserved Pro 6 AAs past the DRY sequence of ICL2, instead substituting an Ala or His residue. SP-R has a Pro but it is 7 AAs past the DRY sequence. (PDF) [file pone.0275410.s002.pdf]

S2. Figure.

***Drosophila* Rhodopsin-like GPCRs do not contain a conserved P**

6 AAs past the DRY sequence of ICL2

Asterisk marks the +6 position

SP R has one (bold and capitalized) but it is 7 AAs past the DRY sequence

LGR1

```
                      *
M(593)          erwlaitqamylnhriklrpaa
V(575)          erwfaithamylnkritlrqaa
                      *
```

Moody PC

```
                      *
M(133)          nryvmithhglyariykrh
V(142)          nryvmithhgcyariykrh
                      *
```

Rickets

```
                      *
M(848)          ernyaithaihlnkrlslkqag
V(889)          ernyaithaihlnkrlslrqag
                      *
```

SP R

```
                      *
M(192)          qryiyvchaPmartwctmprvrr
V(256)          qryiyvchaPmartwctmprvr
                      *
```
