# Supplementary material for: The incidence of candidate binding sites for β-arrestin in Drosophila neuropeptide GPCRs
Source: PLoS One. 2022 Nov 1;17(11):e0275410. doi: 10.1371/journal.pone.0275410 (PMC9624432; doi:10.1371/journal.pone.0275410)
Supplement: S3 Fig — BBS-like and surrounding sequences in the third ICL of Trissin-R PB, PC and PD isoforms. The BBS-like sequence present in the PB and PD isoforms is converted in the PC by alternative splicing (asterisks) yet retains a precise BBS-like character. The correlated change in the surrounding downstream sequence is underlined. Numbers in parentheses identify the predicted AA positions of the first residues illustrated for each isoform (cf. S1 Text and S7 Text). (PDF) [file pone.0275410.s003.pdf]

S3 Figure

The BBS-like sequence in ICL3 of the Trissin-R display sequence refinement due to alternative slicing among its PB, PC and PD isoforms

The BBS-like sequence is shown in **RED** and bold

Alternative splicing alters the final two AAs of the BBS-like sequence in PC (asterisks)

Alternative splicing also alters the peptide sequence immediately following the BBS (underlined)

```
Dm PB(469):  RKQSSKYEKRGVSITESQLDNCKVSLEADRPIVSACRKTSFYHHG
Dv PB(501):  RKHSSKYEKRGVSITESQLDNCKVSLEADRPIVSACRKTSFYHHS
                                 **
Dm PC(469):  RKQSSKYEKRGVSITESQVSLEADRPIVSACRKTSFYHHG
Dv PC(501):  RKHSSKYEKRGVSITESQVSLEADRPIVSACRKTSFYHHS
                                 **
Dm PD(469):  RKQSSKYEKRGVSITESQLDNCKVSLEADRPIVSACRKTSFYHHG
Dv PD(501):  RKHSSKYEKRGVSITESQLDNCKVSLEADRPIVSACRKTSFYHHS
```
