# Supplementary material for: The incidence of candidate binding sites for β-arrestin in Drosophila neuropeptide GPCRs
Source: PLoS One. 2022 Nov 1;17(11):e0275410. doi: 10.1371/journal.pone.0275410 (PMC9624432; doi:10.1371/journal.pone.0275410)
Supplement: S1 Text — (PDF) [file pone.0275410.s005.pdf]

M = ***D. melanogaster*** **V = *D. virilis***

BBS candidates in bold

Predicted TM domains

D. melanogaster

D. virilis

M AKH R – PA (Genbank ref # AAF52426)

1 makvaeendh rdlsnwsnvn dtngtihltk dmvfndghrl sitvysilfv istignstvl
61 ylltkrrlrg plridimlmh laiadlmvtl llmpmeivwa wtvqwlstdl mcrlmsffrv
121 fglylssyvm vcisldryfa ilkplkrsyn rgrimlacaw lgsvvcsipq aflfhleehp
181 avtgyfqcvi fn***sfrsdfd***e klyqaasmcs myafplimfi ycygaiylei yrksqrvlkd
241 viaerfrrsn ddvlsrakkr tlkmtitivi vfiicwtpyy tismwywldk hsagkinpll
301 rkalfifast nscmnplvyg lynirgrmnn nnpsvnnrht slsnrldssn qlmqkqltnn
361 sllngrgqvm aaavsattkl anvvslkgta ngngsaaaag tvpitppltv tiaplatdde
421 andd***sclsav t***ircqdqspi rqk

(***sfrsdfe*** sequence in ECL2)

M AKH R-PC (Genbank ref # AAS64647)

1 makvaeendh rdlsnwsnvn dtngtihltk dmvfndghrl sitvysilfv istignstvl
61 ylltkrrlrg plridimlmh laiadlmvtl llmpmeivwa wtvqwlstdl mcrlmsffrv
121 fglylssyvm vcisldryfa ilkplkrsyn rgrimlacaw lgsvvcsipq aflfhleehp
181 avtgyfqcvi fn***sfrsdfd***e klyqaasmcs myafplimfi ycygaiylei yrksqrvlkd
241 viaerfrrsn ddvlsrakkr tlkmtitivi vfiicwtpyy tismwywldk hsagkinpll
301 rkalfifast nscmnplvyg lynirgrmnn nnpsvnnrht slsnrldssn qlmqkqltnn
361 sllngrgqvm aaavsattkl anvvslkgta ngngsaaaag tvpitppltv tiaplatdde
421 andd***sclsav t***ircqdqspi rqkcgdsiel tsvvk

V AKH R PC (Genbank ref #XP_002051398.2)

1 maqsgevnev vydhrilrdw snvnntngtm hlskdmifnd ghrlsitvys ilfvistign
61 stvlylltkr rlrgplridi mlmhlaiadl mvtlllmple iawawtvqwr stdlmcrlms
121 ffrvfglyls sfvmvcisld ryyailkplq rsynrgriml acawlgsvic sipqaflfhl
181 eehpivkgyf qcv***tfhsfvs efd***nwlyqia tmcamyafpl iafiycygai yleiyrknqr
241 vhkdviaerf rrsnddvlsr akkrtlkmti sivivfiicw tpyyficmwy sldktsvdkv
301 nslvrkalfi fastnscmnp lvyglynirg rmnnnnnvsv nnrhtslsnr ldssnqllqk
361 pintlpnnng nvmaaavaat tklahvvrlk tngaagdsqs pvaaaplaap ldindd***vsvs***
421 ***vvt***tkcqeqt pkqpktpiic lncgdsieva svekt

M AstA R1 PB (Genbank Ref # AAF45884.3)

```
  1 maghqslall latlisswpk aswgatgngs iisvsnssgn nyaftsehtd hsdhnandsm
 61 eydaesvale rivsti
 77 vpvffgiigf agllgnglvi lvvvanqqmr sttnlliinl avsdilfvif cvpftatdyv
147 lpewpfgnvw ckfvqymivv tchcsvytlv lmsfdrflav vhpvtsmslr ternatlaim
207 cawitivtta ipvalshsvr iyqyhgnagt acvfsteeei wslvgfqvsf flssyvaplt
267 licflymgml arlwksapgc kpsaesrkgk rrvtrmvvvv vlafaicwlp ihvilvlkal
327 nlyggshlsv iiqiishvva ytnscinpil yaflsdnfrk afrkvvwcgs ppplmtnqqv
387 tkttrtatgn gtsnieml
```

V AstA R1 PB (Genbank Ref # XP_002055362.1)

```
  1 mglsshrlam alaltvlisf wptgshgaaa vdcadwlsps ecsnstgnnn nnnnnnnkig
 61 nnssnyaags taldhlnssm rpdnllemdl eqdgdnwple rivsii
107 vpvffgiigf agllgnalvi lvvvvnqqmr sttnlliinl avsdilfvif cvpftatdyv
167 lpewpfgnlw ckfvqymivv tchcsvytlv lmsfdrflav vhpvtsmslr ternatlaim
227 cawitivtta ipvalahsvr iyqyhgragt acvfsteeev wslvgfqvsf flssyvaplt
287 licflymgml arlwksapgc kpsaesrkgk rrvtrmvvvv vlafaicwlp ihvilvlkal
347 nmyggthltv iiqiishvla ytnscinpil yaflsdnfrk afrkvvwcgs pppivtnqqm
380 tkttrtatgn gtsnieml
```

M AstA R2 PA **(Genbank Ref #** AAF56809)

1 menttmlani slnatrneen itsfftdeew laingtlpwi vgfffgviai tgffgnllvi
61 lvvvfnnnmr sttnlmivnl aaadlmfvil cipftatdym vyywpygrfw crsvqylivv
121 tafasiytlv lmsidrflav vhpirsrmmr tenitliaiv tlwivvlvvs vpvafthdvv
181 vdydakknit ygmctfttnd flgprtyqvt ffissyllpl miisglymrm imrlwrqgtg
241 vrmskesqrg rkrvtrlvvv vviafaslwl pvqlilllks ldvietntlt klviqvtaqt
301 layssscinp llyaflsenf rkafyk/avnc ssryqnytsd lppprktsca rtsttgl

V AstA R2 (PA-like) (Genbank Ref # XP_002053983.1)

1 mnlsnitltl psnsswlesq lelttassnl dnstlssfya eaeairatvr wvvpfffgii
61 aisgffgnll vilvvllnkn mhsttnlliv nlaaadllfv ifcvpftaid yvtqhwpfgk
121 mwcrsvqyli vvtayasiyt lvlmsidrfl avvhpirsrm lrtehitkia iftlwtvvlt
181 vsmpvtfahd vvvdydnqtn vtyamcryid ndvldlstfq vsffissyll plmvisglyv
241 rmimrlwhqg tgvrmskesq rgrkrvtrlv vvvviafasl wlpvqlilll kaldmyeins
301 mfnvilqiva htmaytssci npllyaflsd nfrkafyk/ai ncsnryhnyt sdlppprkts
361 cgrtsttgl

M Ast-A R2-PB **(Genbank Ref #** AAF56809)

1 menttmlani slnatrneen itsfftdeew laingtlpwi vgfffgviai tgffgnllvi
61 lvvvfnnnmr sttnlmivnl aaadlmfvil cipftatdym vyywpygrfw crsvqylivv
121 tafasiytlv lmsidrflav vhpirsrmmr tenitliaiv tlwivvlvvs vpvafthdvv
181 vdydakknit ygmctfttnd flgprtyqvt ffissyllpl miisglymrm imrlwrqgtg
241 vrmskesqrg rkrvtrlvvv vviafaslwl pvqlilllks ldvietntlt klviqvtaqt
301 layssscinp llyaflsenf rkafyk/glqs nrlgmw***ttth qd***vssektty

V AstA R2 (PB-like) (not annotated; obtained by direct inspection of genomic DNA – see SXX document [AstA R2] isoform annotations)

1 mnlsnitltl psnsswlesq lelttassnl dnstlssfya eaeairatvr wvvpfffgii
61 aisgffgnll vilvvllnkn mhsttnlliv nlaaadllfv ifcvpftaid yvtqhwpfgk
121 mwcrsvqyli vvtayasiyt lvlmsidrfl avvhpirsrm lrtehitkia iftlwtvvlt
181 vsmpvtfahd vvvdydnqtn vtyamcryid ndvldlstfq vsffissyll plmvisglyv
241 rmimrlwhqg tgvrmskesq rgrkrvtrlv vvvviafasl wlpvqlilll kaldmyeins
301 mfnvilqiva htmaytssci npllyaflsd nfrkafyk/ek ikikpn***swhs vgt***rgrrsap
361 lcftyf

M AstC R1 – PA ((Genbank Ref # AAF56809)

1 mftwlmmdvl qfvkgemtad seanatnwyn tneslyttel nhrwisgsst iqpeeslygt

61 dlptyqhcia trnsfadlft vvlygfvcii glfgntlviy vvlrfskmqt vtniyilnla

121 vadecfligi pfllytmric swrfgefmck aymvstsits ftssifllim sadryiavch

181 pisspryrtl hiakvvsaia wstsavlmlp vilyastveq edginyscni mwpdaykkhs

241 gttfilytff lgfatplcfi lsfyylvirk lrsvgpkpgt kskekrrahr kvtrlvltvi

301 svyilcwlph wisqvalihs npaqrdlsrl eiliflllga lvysnsavnp ilyaflsenf

361 rksffkaftc mnkqdinaql qlepsvftkq gskkrggskr llltsnpqipp llplnagnnn

421 ***sstttssttt*** ***ae***k***tgttgtq*** ***kscns***ngkvt appenliicl seqqeafctt arrgsgavqq

481 tdl

V AstC R1 – PA (Genbank Ref # XP_002048373.1)

1 mwllivglhl vggsptesqp ewllmgntst apynypndti ysthsseylp ttgssihstd

61 lptyqhciat rnsfadlftv vlyglvcivg lfgntlviyv vlrfskmqtv tniyilnlai

121 adecfligip fllytmrics wrfgelmcka ymvstsitsf tssifllims adrymavchp

181 isspryrtlh nakvvsalaw stsavlmlpv mlyastveqe dginyscnim wpdaykkhsg

241 ttfilytffl gfatplcfil sfyylvirkl rsvgpkhttk skekrrahrk vtrlvltvit

301 vyiscwlphw msqlalinsn paqrdlsrle iliflllgal vysnsavnpi lyaflsenfr

361 ksffkaf***tcm*** ***tkqd***inaqlq lepsvftkqg srrrggsrrl lttnpqqqqe qqppllalhv

421 gnnn***ssttts*** ***stttae***ktgs tnapkscssn gkltvpgsst apeaenliic lseqheafct

481 ttrrgsslvq qtdl

M AstC R2 – PB (Genbank Ref # AAN11677.2)

1 meggwwrggg gggrlggkai meghstpnga aashrnnstr tniatngcah sgillfvlta
61 mtltslitpt eqlavapngt tlhqlesves esypsingtq netmvtsvrp hldhrnrptq
121 qngshyleyd ddgpdcsysy nfilklitmi lyalvciigl fgntlviyvv mrfskmqtvt
181 niyilnlaia decfligipf llytmqvgnw pfgnymckay mvstsitsft ssiflimsa
241 dryiavchpi sspryrtpfv sklvsafawm tsvllmlpvi lfastvqssn gnvscniewp
301 dtqnshtdst filyslvlgf atpltfilvf yclvirklht vgpkhkskek krshrkvtkl
361 vltvisayif cwlphwisqv alissapqrc asrlelavfl acgclsysns amnpilyafl
421 sdnfkksfmk actcaarkdv naqlqlensf fpkfgkgrqs erllggngkg gaqrgaltkk
481 kclatrnnna pma***ttttttt tttgt***davtc lqppvhqvpa eiqvgnpatv lvvnaetnnc
541 kppvlhtdl

V AstC R2 PB (Genbank Ref # XP_002048370.2)

1 mkgypkpnga anrschmdhv hggcgvllfl ltaltltsli tpteqltats tsvatlangt
61 atvqpmptyt kssdadaaad sdidvdvnse gfspalelsd yykhmnanlv nggsgsgfif
121 psgfngtlpi gfggdppngs gpynpmqgcr rdgsfgleli tmilyalvci vglfgntlvi
181 yvvlrfskmq tvtniyilnl aiadecflig ipfllytmqv gnwpfgnymc kaylvstsvt
241 qftssiflli msadryiavc hpisspryrt pfvskvvsav awttsvllml pvilfastfe
301 sgpghvscsi nwpeafniqs dsafilyslv lgfvtplifi mifyclvirk lhtvgpkhks
361 kekkrshrkv tklvltvitv yimcwlphwi sqvalinstp gcasrlelav flacgclsys
421 nsamnpilya flsdnfkksf mkactcaark dvnaqlqlen sffpkfgkgr qserligpna
481 aannskakkr alanarnnna qm***tttttttt agt***dvvtsiq pvaithtape vtptagaall
541 vvnaetnckp pvlhtdl

M AstC R2 – PD (Genbank Ref # AGB94708.1)

1 meggwwrggg gggrlggkai meghstpnga aashrnnstr tniatngcah sgillfvlta
61 mtltslitpt eqlavapngt tlhqlesves esypsingtq netmvtsvrp hldhrnrptq
121 qngshyleyd ddgpdcsysy nfilklitmi lyalvciigl fgntlviyvv mrfskmqtvt
181 niyilnlaia decfligipf llytmqvgnw pfgnymckay mvstsitsft ssiflimsa
241 dryiavchpi sspryrtpfv sklvsafawm tsvllmlpvi lfastvqssn gnvscniewp
301 dtqnshtdst filyslvlgf atpltfilvf yclvirklht vgpkhkskek krshrkvtkl
361 vltvisayif cwlphwisqv alissapqrc asrlelavfl acgclsysns amnpilyafl
421 sdnfkksfmk actcaarkdv naqlqlensf fpkfgkgrqs erllggngkg gaqrgaltkk
481 kclatrnnna pma***ttttttt tttgtdavt***c lqppvhqvpa eiqvgnpatv lvvnaetnnc
541 kppvlhtdl**x** drapsmplet vvfiarr

X = stop suppression

M AstC R2 – PF (Genbank Ref # AGB94708.1)

1 meggwwrggg gggrlggkai meghstpnga aashrnnstr tniatngcah sgillfvlta
61 mtltslitpt eqlavapngt tlhqlesves esypsingtq netmvtsvrp hldhrnrptq

121 qngshyleyd ddgpdcsysy nfilklitmi lyalvciigl fgntlviyvv mrfskmqtvt
181 niyilnlaia decfligipf llytmqvgnw pfgnymckay mvstsitsft ssiflllimsa
241 dryiavchpi sspryrtpfv sklvsafawm tsvllmlpvi lfastvqssn gnvscniewp
301 dtqnshtdst filyslvlgf atpltfilvf yclvirklht vgpkhkskek krshrkvtkl
361 vltvisayif cwlphwisqv alissapqrc asrlelavfl acgclsysns amnpilyafl
421 sdnfkksfmk actcaarkdv naqlqlensf fpkfgkgrqs erllggngkg gaqrgaltkk
481 kclatrnnna pma***ttttttt tttgtdavt***c lqppvhqvpa eiqvgnpatv lvvnaetnnc
541 kppvlhtdl**x** drapsmplet vvfiarr**x**dh qveldldtai dcqaiarqpe cll

X = stop suppression

M CAPA R PB (Genbank Ref # AGB94708.1)

1 mnsstdptfs elnasftntp dtlfatsvss dpshgfgeed yacgtfncsp kefvafvlgp

61 qtlplykavl itiifggifi tgvvgnllvc iviirhsamh tatnyylfsl avsdllyllf

121 glptevflyw hqypdlfgmp fckirafise actyvsvfti vafsmerfla ichplhlyam

181 vgfkrairii talwivsfis aipfgllsdi qylnypldhs rieesafcsm spkivneipv

241 fevsfciffv ipmiliilly grmgakirsr tnqklgvqqg tnnretrnsq mrkktvirml

301 aavvitffvc wfpfhlqrli flyaknmdny ldinealfsi agfayyvsc//t vnpivysvms

361 rryrvafrel lcgkavgayy nsgfardhss fressaydrv hs//vhvrasqh pnkfetdsss

421 anrvlikkty slplpk/nads ***tvlsttd***ivi vlen***shtvce*** epkvendiwi eneetci

(/ = intron)

V CAPA R X2 PB (Genbank Ref # XP_002047623.2)

1 mnmnmstnms mdtnlstylg tsdatalpyp gmddygcphm nctamefvqf vlgpqtlplh

61 kallisiifs gifitgvlgn vlvcmviirh aamhtatnyy lfslavsdll ylllglpaev

121 flywhqypyl fglpfcklra fvseactyvs vftivafsme rflaichplh vcamsgfqra

181 lriitalwiv sflsaipfgv kteiqylnfp n/dgsrilesa fcsielefpe efplfevsfc

241 iffiipmili illygrmgaq irsratdklg/vqqgsrnres rssqkkkravirml/aavvit

301 ffvcwfpfhl qrlwflyakn ianyqdvnew lfsiagfayy vs//ctinpivy nvmsqryrva

361 fkeilcgkka gayynsgfar dqssfirdes sfrrgssatp nlrgs***trysr vs***ang/madcs

421 llntttkivi vlgnnspqrd vdrnipeete akkqen

M CCAP R PC (Genbank Ref # AGB94708.1)

1 mlhlrlfdss lyytlasase ssglasstst ersfngtqga ggvaaggesl tptdvaavnl
61 tyftpaishv mlapttiatt tasatmvqiq ttaapshdle tggnstssdp gefdnlnsfy
121 fyeteqfavl wilftvivlg nsavlfvmfi nknrksrmny fikqlala/dl cvgllnvltd
181 iiwritiswr agnlackair fsqvcvtyss tyvlvamsid rydaithpmn fsks/wkrarh
241 lvagawlisa lfslpilvly eekliqghpq cwielgspia wqvymslvsa tlfaipalii
301 sacyaiivkt iwakgsifvp t/eragfgaap arrassrgii prakvktvkm tltivfvfii
361 cwspyiifdl lqvfgqiphs qtniaiatfi qslaplnsaa npliyclfss qvfrtlsrfp
421 pfkwftccck syrnnsqqnr chtvgrrlhn ***scdsmrtltt sltvsrrs***tn ktnarvvice
481 rptkvvtvpa msev

V CCAP R PC (Genbank Ref # XP_002053465.1)
1 mlhlrlfdss lyytlasvss gmlpppqasn gsqltltgaa agiapaestv nltyftpais
61 hvmlaptptt asaaqtteaa ttttatptta stttpgsdfe aaaataasgd nltspyagel
121 dnlnsfyfye teqfavlwil ftiivlgnsa vlfvmfinkn rksrmnyfir qlaladlcvg
181 llnvltdiiw ritiswragn vackvirfsq vcvtysstyv lvamsidryd aithpmnfsk
241 swkrarhlva gawllsalfs lpilvlyeek liqghpqcwi elgspmawqi ymclvsaalf
301 avpaliisac yaiivktiwa kgsifvpter vgfggaatrr assrgiipra kvktvkmtlt
361 ivfvfilcws pyiifdllqv fgqiphsqtn iaiatfiqsl aplnsaanpl iyclfssqvf
421 rtlsrfppfk wltccccksyr nnsqqnrcht vgrrlhn***scd smrtlttslt vsrrs***tnktn
481 arvvicerpn kvitvpamse v

M CCAP R PD (Genbank Ref # AAS65092.1)
1 mlhlrlfdss lyytlasase ssglasstst ersfngtqga ggvaaggesl tptdvaavnl
61 tyftpaishv mlapttiatt tasatmvqiq ttaapshdle tggnstssdp gefdnlnsfy
121 fyeteqfavl wilftvivlg nsavlfvmfi nknrksrmny fikqlala/dl cvgllnvltd
181 iiwritiswr agnlackair fsqvcvtyss tyvlvamsid rydaithpmn fsks/wkrarh
241 lvagawlisa lfslpilvly eekliqghpq cwielgspia wqvymslvsa tlfaipalii
301 sacyaiivkt iwakgsifvp t/eragfgaap arrassrgii prakvktvkm tltivfvfii
361 cwspyiifdl lqvfgqiphs qtniaiatfi qslaplnsaa npliyclfss qvfrtlsrfp
421 pfkwftccck syrnnsqqnr chtvgrrlhn ***scdsmrtltt sltvsrrs***tn ktnarvvice
481 rptkvvtvpa msev**laht srkrsafsih gpt***sfsdae***f l

** Stop Suppression

V CCAP R (PD-like) (not annotated – obtained by direct inspection of genomic DNA; see SXX Document de novo [CCAP R] isoform annotations)
1 mlhlrlfdss lyytlasvss gmlpppqasn gsqltltgaa agiapaestv nltyftpais
61 hvmlaptptt asaaqtteaa ttttatptta stttpgsdfe aaaataasgd nltspyagel
121 dnlnsfyfye teqfavlwil ftiivlgnsa vlfvmfinkn rksrmnyfir qlaladlcvg
181 llnvltdiiw ritiswragn vackvirfsq vcvtysstyv lvamsidryd aithpmnfsk

241 swkrarhlva gawllsalfs lpilvlyeek liqghpqcwi elgspmawqi ymclvsaalf

301 avpaliisac yaiivktiwa kgsifvpter vgfggaatrr assrgiipra kvktvkmtlt

361 ivfvfilcws pyiifdllqv fgqiphsqtn iaiatfiqsl aplnsaanpl iyclfssqvf

421 rtlsrfppfk wltccckksyr nnsqqnrcht vgrrlhn***scd smrtlttslt vsrrs***tnktn

481 arvvicerpn kvitvpamse v**lahstrk rstfavhgpt ***sfsdae***fl

M CCHa1 R-PA (Genbank Ref # AAF57819)

1 mianlvsmet dlamnigldt sgeaptalpp mpnvtetlwd lamvvsqstq wplldtgsse

61 nfselvttet pyvpygrrpe tyivpilfal ifvvgvlgng tlivvflsvr qmrnvpntyi

121 lslaladllv iittvplast vytveywpyg sflcslsefm kdvsigvsvf tltalsgdry

181 faivdplrkf hahgggrrat rmtlatavsi wllailcglp aligsnlkhl gineksivic

241 ypypeewgin yaksmvllhf lvyyaiplvv iavfyvlial hlmysasvpg eiqgavrqvr

301 arrkvavtvl afvvifgicf lpyhvfflwf yfwptaqddy nafwhvlriv aycmsfansc

361 anpvalyfvs gafrkhfnry lfcrgasgrr kkrgqhdtfc mhrdt***sltst as***krfqsrhs

421 cyqstirscr lqettittlp nggnqngani savelalpvl qapghneaha ppsygflpln

481 eivqqtrssp akfqeslln

V CCHa1 R (Genbank Ref # XP_002049859.1)

1 mmtsvlsdis tiamdmelgs gsesplspln atralwelam tttaetplfd engslvpvte

61 ipyvpygrrl etyivpilfa iifvvgvlgn gtlivvflsv rqmrnvpnty ilslaladll

121 vilttvplvs tvyaveywpw gsflcsvsef mkdvsigvsv ftltalssdr yfaivdplrk

181 fhahgggrra trmtlaiavs iwllaiicgl paligsnlkp vginqeksiv icypypeawg

241 dnyaklmvml hflvyyaipl viiaafyvmi alhlmysasv pgemqgavrq vrarrkvavt

301 vlafvvifgi cflpyhvffl wfyywptaqq dynmfwhvlr ivgfcmsfan scanpvalyf

361 vsgafrkhfn rylfcrgisg rrkkrnqhnd tfcmhrnt***sl tstas***krfqs rhscyqstvr

421 scrlqettit tlpngglnga ps***tavthne***a pgyeftplsd fgplkasqla qrlqespln

M CCHa2 R-PA (Genbank Ref # AAF57285.4)

```
  1 myaslmdvgq tlaarladsd gngandsgll atgqgleqeq eglaldmghn asadggivpy
 61 vpvldrpety ivtvlytlif ivgvlgngtl viiffrhrsm rnipntyils laladllvil
121 vcvpvativy tqeswpfern mcriseffkd isigvsvftl talsgeryca ivnplrklqt
181 kpltvftavm iwilaillgm psvlfsdiks ypvftatgnm tievcspfrd peyakfmvag
241 kalvyyllpl siigalyimm akrlhmsarn mpgeqqsmqs rtqararlhv armvvafvvv
301 fficffpyhv felwyhfypt aeedfdefwn vlrivgfcts flnscvnpva lycvsgvfrq
361 hfnrylccic vkrqphlrqh statgmmdnt svmsmrrsty vggtagnlra slhrnsnhgv
421 ggagggvggg vgsgrvgsfh rqdsmplqhg nahgggaggg ssglgaggrt aavsek/sfin
481 ryesgvmry
```

V CCHa-2 R PA (Genbank Ref #XP_002049178.2)

```
  1 mpknmlaalm dmsqtlaasl ayaplesnaa ataaaaaaaa vvlnvsqlgn ssqldgslat
 61 aaattttav ttststhnas geeypqyvkv ldrpetyivt vlytlifivg vlgngtlvii
121 ffrhrsmrni pntyilslal adllvilvcv pvativytqe swpfernmcr iteffkdisi
181 gvsvftltal sgerycaivn plrklqtkpl tvftaviiwv faimlgmpsf vvsdiqgytl
241 ptpngnitie vcspfrskiy akymvvakas iyylvplsii gvlyimmakr lhisardmpg
301 eqlsiqsrsq ararrhvarm vvafvvvffi cffpyhvfel wyhfyptaee dfddfwhvvr
361 ivgfctsfln scvnpvalyc vsgvfrqhfn rylccicvkr qphlrqhsta tgvmdtsvts
421 mrrstyvggg gggavggsla ahraslhmnn nhgvavgggg ggggrggsfh rqdsmplqha
481 gsgnghahnv ggpgagigra siinek/slik rydertry
```

M CCHa2 R PB (Genbank Ref # AAF57285.4)

```
  1 myaslmdvgq tlaarladsd gngandsgll atgqgleqeq eglaldmghn asadggivpy
 61 vpvldrpety ivtvlytlif ivgvlgngtl viiffrhrsm rnipntyils laladllvil
121 vcvpvativy tqeswpfern mcriseffkd isigvsvftl talsgeryca ivnplrklqt
181 kpltvftavm iwilaillgm psvlfsdiks ypvftatgnm tievcspfrd peyakfmvag
241 kalvyyllpl siigalyimm akrlhmsarn mpgeqqsmqs rtqararlhv armvvafvvv
301 fficffpyhv felwyhfypt aeedfdefwn vlrivgfcts flnscvnpva lycvsgvfrq
361 hfnrylccic vkrqphlrqh statgmmdnt svmsmrrsty vggtagnlra slhrnsnhgv
421 ggagggvggg vgsgrvgsfh rqdsmplqhg nahgggaggg ssglgaggrt aavsekr
```

M CCHa2 R PC stop suppression **(Genbank Ref #** QCD26194)

```
  1  myaslmdvgq tlaarladsd gngandsgll atgqgleqeq eglaldmghn asadggivpy
 61  vpvldrpety ivtvlytlif ivgvlgngtl viiffrhrsm rnipntyils laladllvil
121  vcvpvativy tqeswpfern mcriseffkd isigvsvftl talsgeryca ivnplrklqt
181  kpltvftavm iwilaillgm psvlfsdiks ypvftatgnm tievcspfrd peyakfmvag
241  kalvyyllpl siigalyimm akrlhmsarn mpgeqqsmqs rtqararlhv armvvafvvv
301  fficffpyhv felwyhfypt aeedfdefwn vlrivgfcts flnscvnpva lycvsgvfrq
361  hfnrylccic vkrqphlrqh statgmmdnt svmsmrrsty vggtagnlra slhrnsnhgv
```

421 ggagggvggg vgsgrvgsfh rqdsmplqhg nahgggaggg ssglgaggrt aavsekr**x**gt

481

M CCK-R 17D1 PA (Genbank Ref # ABW09450)

1 mlprlcadac rqcfakiarr dthrgtrtpy gcadtqsrpk pnfllrevde vcctaasasp
61 rllvlfrdhk rasffgltid afyhylrqal plakeaaihl nasneisavg dgvtitgtpg
121 dllnysglel dlgldldlnl dmdlattpss stlapavtvr tpgnrsvvrv sadvpiwvvp
181 cysaillcav vgnllvvltl vqnrrmrtit nvfllnlais dillgvfcmp vtlvgtllrh
241 fifgellckl iqfaqaasva vsswtlvais ceryyaichp lr***srtwqt***in hankiiaiiw
301 lgslvcmtpi aafsqlmpts rpglrkcreq wpadslnyer aynlfldlal lvlpllalsf
361 tylfitrtly vsmrneramn fgssgpevtt sssaavaeag sqrrangshc qsldtivphq
421 hnphqqhhhh sqyyydyghc gskrrlisgg gpcegrrhly cmr***sasvks***l rhqqinggggg
481 tlsgtgagng eccsrvhrmr qqmqlqqqgy vsdnesrrks lsqpslrite aglrrsnetk
541 sleskkrvvk mlfvlvleff icwtplyvin tmtmllgptv yeyvgytsis flqllaysss
601 ccnpitycfm nasfrrafvd tfkgmrvcer lcapccfwrr rsknetnlsv agnsialans
661 vm***sshtile***s prl

V CCK-R 17D1-like (Genbank Ref # XP_032295977.1)

1 msgslaseat mtsatasatv mptplvpsnr smsrviadvp iwvipcysii llcavvgnll
61 vvltlvqnrr mrtitnvfll nlaisdillg vlcmpvtlvg tllrnfifge slckliqfaq
121 aasvavsswt lvaisceryy aichplr***srt wqt***inhanki iafiwlgslv cmtpiaifsq
181 lmptsrqglr kcreqwpans lgyeraynif lnlallvlpl malsfaylfi trtlyvsmrn
241 eramnfgssg pdvglttnss ssnnnssctg irrtygnsny lmrmqmrqea laidgsakld
301 mllqqqkasg ppqyyyaegy tqggskrrlf gscdgrrhly cmr***sasvks***l rqqqqqqql
361 ggsgdccarm qrmrqqqlnm tagndgerrk slstpslrit eatlrrsnes kskleskkrvv
421 kmlfvlvlef ficwtplyvi ntmtmligpv vyeyvdytai sflqllayss sccnpitycf
481 mnasfrrafv dtfkgmrlcd ggrfgfcrrr sknetnlsva gnsialansa m***sshtile***sp
541 rl

M CCK R 17D3 PB (Genbank Ref # ABW09450**)**

1 mfnyeegdad qaamaaaaay ralldyyana psaaghivsl nvapyngtgn ggtvslagna
61 tssygdddrd gymdtepsdl vtelafslgt ssspspsstp asssststgm pvwlipsysm
121 illfavlgnl lvistlvqnr rmrtitnvfl lnlaisdmll gvlcmpvtlv gtllrnfifg
181 eflcklfqfs qaasvavssw tlvaiscery yaichplr***sr*** ***swqt***ishayk iigfiwlggi
241 lcmtpiavfs qliptsrpgy ckcrefwpdq gyelfynill dflllvlpll vlcvayilit
301 rtlyvgmakd sgrilqqslp vsattaggsa pnpgtssssn cilvltatav ynensnnnng
361 nsegsagggs tnma***tttltt*** rp***taptvitt*** ***ttttt***vtlak tsspsirvhd aalrrsneak
421 tleskkrvvk mlfvlvleff icwtplyvin tmvmligpvv yeyvdytais flqllaysss
481 ccnpitycfm nasfrrafvd tfkglpwrrg agasggvgga aggglsasqa gagpgaya***sa***
541 ***ntnis***lnpgl amgmgtwr***sr*** ***srhe***flnavv ttnsaaaavn spql

M CCK-R 17D3-like (Genbank Ref # XP_032295927.1**)**

1 mysasaedaa saaatykall dyyanarsaa shivsltlap lneslslgla eagngnasgn
61 anssasyedd alsaenifli tesvatessr gaaaaangva vgsgrsssss aptempawli
121 psyslillca vvgnllvist lmqnrrmrti tnlfllnlai sdmllgvlcm pvtlvgtllr
181 nfifgeflck liqfaqassv avsswtlvai sceryyaich plr***srswqt***i shaykiigfi
241 wlggilcmtp iavfsrlipt srpgfckcre hwpdqqgyerf ynimldlill vlpllvlcaa
301 yilitrtlyv gmnvgkdarm pa***ssgsaqt***v ataaiaatpg ssscvlvlna aseynessnn
361 nnaattstta aa***tattttta*** ***tatttatatt*** ***tlttittttl*** ***ttvt***pakssn aspslrihda
421 alrrsnetrt leskkrvvkm lfvlvleffi cwtplyvint lsmfigqtly eyidyttisf
481 lqllaysssc cnpitycfmn asfrrafvdt fkglpwkrng taagglsasq ggvpnlsnpn
541 pglamgmdtw r***srsrnd***qll nsvvytnsaa aaadspql

M CNMa R (Genbank Ref # ABW09450)

1 mdmeyitsss gnitattead fssslgesnv teynttemda nesagedeem lriaffighf
61 vhqyyipvlc ctgsignils vfvffrtklr klsssfylaa lavsdtcfla glfaqwlnfl
121 nvdiynqnyf cqfftffsyl asfcsvwfvv aftverfiav iyplkrqtmc tvrrakivlf
181 cltlvgclhc lpyiviakpv fmpklnttic dlnseykeql alfnywdtiv vyavpfttia
241 vlntctgctv wkfatvrrtl tmhkmkpqtn ***smpsnssnss*** ggassavasy rlsaslkrqk
301 stgthpsgqh nvanrqtddq eqqqqsqqhq inncqhhcei tqkparrkvq nssqlkvtkm
361 llivstvfvc lnlpscllri eaywetesar nqnstialqy ifhaffitnf ginfvlycvs
421 gqnfrkavls ifrrvssaqr eagn***tqvtvs e***ycrntgtst rrrmmtqhcw nemhelhplk

V CNMa R (Genbank Ref # XP_002047272.3)

1 manpyepttm tamsntsqdi sapttttttt tattatvttt lsyttdssrn isidleymsv
61 edeemlhiaf lisdfvnryy vpiicctgsi gnilsvfvff mtklrklsss fylaalaisd
121 tcflcglfmq wlnflnvniy nqnyfcqfft fisylasfcs vwfvvaftve rfiavmyplk
181 rqimctvrra kivllgltla gcvhcvpyil iakpvyspkl ndticdlnts ykeqlalfny
241 wdsivvyavp fttitvlntc tgctvwkfat vrrtltmhkm kpqitnvpan attgggvaaa
301 tyrisaslrr qkstgthpsg qh***svsrqt***eq qqqqqqqqrh darsqhhcei tqktgrrkvq
361 nssqlkvtkm llivstvfvc lnlpscllri etywetqtsk tqnttivlqy ifnaffitnf
421 ginfvlycvs gqnfrkavls ifrrvssaqr egi***tqvtvse*** ycrntgtstr rrmmtqhcwn
481 emhelhplr

M 4313 R PD (Genbank Ref # AAF45710)

```
  1 mfppksgptp hpisialpls dpyatdhmad qdavlpvpls dsldldvdvd lnlnmnlnln
 61 vddrrqvlfe gysdelltia wvacivfiiv gvpgnlltiv alsrgrqtrn staifiinls
121 csdllfgcfn lplaastfke rawthsdllc rlfpmlrygl lavsllsvsl itinryiiia
181 hprqypriyq rrylalmvag twittfsimi ptwrgvwgif gldvsigscs imhdrygrsp
241 keflfiaafm vpcicivicy arifllvrka airagtagkt nvsdvtpssa pqhqiqamat
301 pkkpekvtts sgeanepiag rpfvveenla yiddnastds lpisysirrr dqdqqpppvd
361 anvvlkerek erdrdqekvs lgrsqtqlem gkthgknpit tslrttsftr fsprkshyas
421 mgntsnassi ypgrmsakdr rllkmilvif vwfvicylpi tvakiwksat evhwfniagy
481 lliylttcin pliyvlmsse yrraywnllr chgspdtqkq rnqanakrkh lesnrqvkat
541 t
```

V 4313 R PD-like (Genbank Ref # XP_002057364.1)

```
  1 mihqldsqaq gldiqllgvp gavaqplala rkaggtnrsn smdqenelfe gysdelltfa
 61 wiacivfiiv gvpgnlltiv alsrgkqtrn staifiinls csdllfgcfn lplaastfke
121 rawthsdllc rlfpllrygl lavsllsvsl itinryiiia hprqypriyq rrylalmvag
181 twlvtfsimi ptwrgvwgrf gldtsigscs ilhdkydrsp keflfmaafm lpcvcivicy
241 arifllvrqa amragaksse laitppiqtp tqtqkppvad kpkdkskvks kdrqqeldkd
301 adfgtsrpfv vaeslayidd nassesfpis ysikqeapid anvvlqdnnl annkhsvsgs
361 apatgpatad atvnksqsql eqqrnsknpi ttslrtsftr fsprkshyvs mgntsnassi
421 ypgrmsvkdr rllkmilvif vwfvicylpi tvakiwksan dvhvfnimgy lliylttcin
481 pliyvlmsse yrraywnllr chqteqqqqq qralkkhles nralkt
```

M 12290 R PA (Genbank Ref # AAF45710) **(starts just before TM2)**

1 maidllilal llvsflinll alcafwitpg lrttanrfti nllainligc cilaptlflg
61 lpgksaeast snaetleffs kpgnhqvilr rngqlveqdg vvvrrnisen gdtvetffkc
121 natycrelti dergdggfvi tetetheenl safeslptea pilppvqlrc wsidmtaalg
181 alavllvvgd twcavtdplr yhsrisgvkt wifialtwvv gilfgalsaf rvldfeadal
241 fsrqrrlavt yfnisstnsi fgvvyasvyf iviillpfgf vcgmywrifs eargnglrmr
301 qngsspllqs alnltagqqa aqanqfsnsl cvhrh***sissa ss***hggnsslg lgglqmqidq
361 rqqprsspsc lrrdsaakvl lptisddggs daesgagvql mpvqehslsd rnqnimltlq
421 tasgeikrny sarqlpllgt ssqdlretnr lqgirqvhss pnlhkytelr qdslseecgs
481 phllghaqrq qqqqlhlqhq qqqhhhhqqh hphfssprhq qhghalqipa ihaspkalsy
541 msslrhrlsn asslfkyree sraarisilv vvmfvvsylp fgllvllqsr lsaanfggss
601 qlaifmilla nlsspfifay rnkrvrrgvk rlfgldsssg lqrnc***sssvk tngt***agpaas
661 gaqlqrns***sk lsqyssns***ck yltpqsslvs qvpvhttltl rpnsscstii nfggargsad
721 sdeqppatpp ptvavapppt rpqrpklqrg itivehiait ptmpqkfqnr rarlfdmffr
781 sskklqagcq sqslptev

V 12290 R (Genbank Ref # XP_002053408.1)

1 maidflilav llisfiinll alcafwitpg lrttanrfti nlliinligc cilaptlfls
61 gfkslsgqee qlsgvnaagd siefyskpgn hqltirhhgq lvekdgivlr knitsgnsns
121 ndssgdtiem iykcnatycr eltidergdg gliitetetr ednlsvsvhs ntslpavqlr
181 cwsidmtaal galavllvvg dtwcavtdpl ryhsrisgvk awifitltwv vgivfgalsa
241 frvldfetdt llsrqrrlaa tyfnisstss ifgviyacvy fiviillpfg fvcgmywrif
301 seargnglrm rqngsspllq salnltnhaq aapatpyans lcvhrh***siss ass***hggggvl
361 sgglqmqidq raprnspscl rrdsaakvll ptisddgsdv dvesshggqq lmsvpeqtva
421 drnqnilltl qtasgeikrn ysarqlpllg tssqdlrelh rlqgirqvhs spnlhkytel
481 rqd***sltlse***e cssphllqah rqqqqhsrql hqqqqqlqql paqqqlhthf stgpqqvaag
541 halqipaiha spkalsymss lrhrlsnass lfkyreesra arisilvvvm fvvsylpfgl
601 lvllqsrlsa anftgstqla ifmillanls spfifayrnk rvrrgvkrlf gldaasalkr
661 qhssslknhg hsaaassapq lqrns***srlsq yssns***crylt pqsslvsqcq tttpvhttlt
721 lrpnsscsti inygghhkgs adsdevpsle atpppqrqtr pklqrgitiv ehihiagspp
781 kfqsrrglld mffrgskkmq sscseampte v

M 13229 R PA (Genbank Ref # AAF58717)

```
  1 mmqetgnqmg qthmhqrvpf ndtvlkdyhl tstdiekfvk lwqeyqmknm tpqvdecqgy
 61 cqgeiynwlr aynsihgyvs lmicifgtia nilnimvltr kemaktpinn ilkwlavadm
121 fvmleyipyt syqyiymgpg ekdlsytwav cllvhmhftq ilhtisiglt vtlavwryva
181 irhpnggcan fllahsreai llpfilspil clptyfvfqv retydvdkvn seamyhvyfd
241 kdsvlyrfnf wihsvlikll pcgilivisa vlmhvlceas rrrlklrdyn npakyaiqln
301 lnetkskkpp rcdrrndrtt lllvavlvlf litefpqgll gllsgvmekc ffahcyppfg
361 elmdllalin aavgfvlygl mskqfrttfr slfmkrhfgs temtrltrvt ttcv
```

V 13229 R PA (Genbank Ref # XP_015024303.1)

```
  1 mqetnmaqrq rephvalnet llkdleitss diqdfvkllf dfqkknhsqq decqgycqge
 61 iynwlrayng ihgyvsllic ifgtianiln imvltrrema kapinnilkw lavadmfvml
121 eyipyttyqy iymkpgekdl syawavyllv hmhftqilht isigltvtla vwryvairhp
181 ngscanflla hsreailfpf iispivclpt yfvfkvretl evdtrehevm yhvyfdvdsm
241 lfrfnfwihs viikllpcci ltvislvlmh vlceasrrrl klkdydnptk yaiqlnlnet
301 ksrrpprcdr rndrttlllv avlilflvte fpqgllglls gvlekcffah cyppfgelmd
361 llalinaavg fvlyglmskq frttfrslff krhfgssemt rltrvtttcv
```

M 13575 R PA (Genbank Ref # AAF58717**)**

1 mallhytfdq lelylewafa qhgeatpips iqpypgvfvg dlsqlnrfkr hafsavvgtl
61 fvlafcgnls tlyvnsrrkl rpffraclis lacsdlvssi fctvsymaqf qaqylqlwti
121 ggfmckfvpf itttsvlsgs ltlvaialdr ylavmrpvlg fwspdkrfst lsmlliwacs
181 igssgpllgi ydyrkiylld vedsseesee vvtavpeelv vtelemvhmc lagdhdvgly
241 yvilftlifl pcivsflwln aviarqlwlr rhyhqeqqeq hqepkegqfk tmanggdllm
301 pstlvsamgv avpfaldntp lppkstvnap gkkttaaala rearhrkmvv vvllmmavfi
361 clrlpawvfl imrlygsyse pidwllyfsf gilnlfscal npifytfltq tirtltlvkh
421 kiqgflgcpp gkvpdgmptd qmdksgcccg lrpptftwrc hpsrdraaat virdvdqpdp
481 psdqvqpdps slrrflsykq evftiykqcg ds***ssasies***s a

V 13575 R PA (Genbank Ref # EDW60637.1**)**

1 malrsliidt ddlypivias dlcqihdtpr yifmalvtvl flctfvgnvs alyvntrrkl
61 rpffraclis lacsdliycv nfttsntamf naeyleywil gpfmcnfvpf vntttvlcss
121 fmlvaialdr ymairraaig iwnpgfvfcg vciagiwlac maaavplffi ytpiqvyiqn
181 tdelliseld qatmcvgrrt qigiynsvsl slvfvpciva fvflnatiar qlwqlrhqqr
241 nlqqqqqqqr eqdqprfvhl lnkpettyam mtafsvaasf dmstaqltgl ppplplplpe
301 klspaaaarv arhrrmvrvv llmmgafmcl rlpawtfllm rvygsfsspv swlfyfsfgl
361 lnltscalnp lfytflpqti rvlsklkral srllccrras klesdatmpq etaerarrcl
421 ccglqvtwrc hlksppaaag svvtqtvavi eapsaasslp pvghckddyk dlaiynen***sl***
481 ***qttasmks***sr

M 13995 R PA **(Genbank Ref #** AAF52333)

1 mnrdnlqqww ensyrrqhpe ptddlgldsa elhlalqepn qlpadydygn fslgnpydvd

61 sehsispltl lllavsyglv vfggvvgnst lvl***tlcsass*** vrlrnpllla vciadllvtg

121 isapvtllnl amnrrtrslp lvlckvihyv qvmpvsasti sffmlsldry atvkhprlaq

181 lrqrrylhvs lallswlasa aistpflfay kiiaksmvvk gggaanttpn pvsisctsdl

241 ganamfmsfi ifhtiavfvl pgigvllnhy gvrrklcals ltaraahgel plpipilrrq

301 thmvivtgcp naqqaacggg ttaddtsngn gtgtgggpma vspgdiqlht lqprqpgsag

361 salepgsyrs snpispramr eirahsqrqr inragrgpat pgiplpq***tst*** ***lrs***rrhlanm

421 liasavifia cwaphvfcif yknfgnnqqc sqtsvyfsll lgyfysaisp viywalnhns

481 lrqspcapii rlrsmqnflr srfrthtapp ppsstneaal gafnpklikl tpkqyraqas

541 shyly

V 13995 R PA **(Genbank Ref #** XP_015028190.1)

1 mnsnnlqqww ensyrrqhqq qptvdsnidn dndsdateql hyalleqnnf ggnsggidgn

61 fggqlsssfd ydsygnftlt npydidtgad tehaipslti lllaisyglv vfggvvgnst

121 lvl***tlcsass*** vrlrnpllla vciadllvtg isapvtllnl amnrgtrslp lllcklihyv

181 qvmpvaasti sffmlsldry atvkhprlaq lrqrrylhvs lailswiasa aistpflfay

241 kiiaksviik gagtgttpnp vsisctselg anamfmsfii fhtiavfvlp gvgvllnhyg

301 vrrklcalsl taraahgelp lpmpilrrqt hmvivtgcan aqqagcggat taddtsngng

361 ngggqiainp gdiqlhnlqp crpgssaale pgsyrssnpi spramreira hsqrqrifra

421 grgpatpgip lpq***tstlrs***r rhlanmlias alifivcwap hvfcifyknf gykqycskts

481 vyfslllgyf ysaispviyw alnhntlrqs pcapiirlrs mqnflrsrfr shtvppaass

541 tneaalgafn pklikltpkq yraqasshyl y

M 30340 R PA (Genbank Ref # AAF52333)

1 masvsssddf dfgkwdfpae riwlhkpnge itwkictflp liafglygnf smvyvia***tnr***
61 ***slrs***ptnlii anmavadllt laicpamfmv ndfyqnyqlg cvgcklegfl vvvflitavl
121 nlsvvsydrl taivlpmetr ltirgvqivv vctwvsgill asplafyrsy rvrvwknfte
181 ryckentsvl pkywyvliti lvwlplgiml icyiaifykl dryekrvlsr enpltvsykr
241 svaktlfivv vvfaalrlpf tilvvlreky fgedvsvssg mqlfwyisqy lmflnaavnp
301 liygfnnenf rrayyqiswv rrwrdatqmk kfsrspdhcc ycafmkngkr tseaaqkagn
361 lekdiskdms saqqsakstk ivenefvsei eadgfi

V 30340 R PA (Genbank Ref # XP_032293011.1)

1 mtaynysiqq fdfsqwdfpa eriwlhkane eiawkiisfl pliifglygn yiliyliatn
61 ralrsptnli ianmamadfl tllicpamfl indfyqnyql gcvgcklegf lvvvflitav
121 lnlsvvsydr ltaivlpqet rltlcgariv iagtwlagll lalplaiyrq yrvriwrnft
181 eryckenmtv lpkywyvlit vlvwlplgim licytaifvk ldryekrvls renplsvryk
241 rsvaktlfiv vivfvllrlp ftifvvlrek yy***stessvd***c gmkyfsyfsq ylifvnaavn
301 piiygfnnen frrayaqiac mqkrraanan rihhclycdf iqnnksgqan aeqrsdkeis
361 qsaaretkkl gatsnidetl mpqlkgegfi

M 32547 R PC (Genbank Ref # AAX52506)

1 mspaeqlrlv gvsdayqlaa ssggaggggg gggggggggg lggyggggsg gdaggsgekm
61 kdvpdkyvta lshfldwhsn gtvdmerlsg pilkssiksv ywlfliqyaa lallgvvlnv
121 iivvyimyhr lykdvthafi inlalchfvq calvlpvslm vmliqnwifg qflcfflpml
181 qdiplhvami shiliawdrm rwlndplkgr lpgfvcccat wltgmvialp ypiytiyvel
241 gdympqlsgi glcvvnlmdd mqeytrglfl lmycgpaill sylyirtsqe lrppdgpfav
301 mmyehradlr mrqrns***stss ve***prhlsggg vaglsngggn sarsydlysa eldvhrekrk
361 qrnfgsmaat qvvcmcplmi lrfarlslee tyenakhfdf tylmfvwvaf lptvifpciy
421 asqilprdeq erlrgyfrls skrkqksqrr sdagggsgvg gssredsiek deasnttsvh
481 haaephkhss klrherevrl pghgssaggd rytgapyrqg kdrerererd rdrerdrdre
541 rererqragg rgagdagvvn nlgkdvrgkk hdvkiniisd gvghgatshs haahpahrkq
601 tggnnnnnnn ssrsrtnpgq rhggskltad cl***snvtast***f cngsgsvtag atgngngpsg
661 vlipddssts nygdgeessv vssmmvppqr wpgsgggvlr hkdv***sfsecs stfssstle***r
721 dleimdqler ersmdiqeml qrererekvr rqlpdiekly aqrspkgkrg eaagagagtg
781 aglalvmpgs dplsslsq***sr sisteyslcs*** **tlet**sgagvs vghdevlphh leeveeevvp
841 pdfydsytpg gtqnlppnag gavpvataav payqyqysrn rlsrkssgss sghhhhghhh
901 gqhggggvgg vaggrgskrd sfnslngtdl iagafceldp tqplnkmavi egrmrrsspr
961 na***sfssgsgv sgrs***smksss rdydyghgss shsahpeldf renifael

V 32547 R PC (Genbank Ref # XP_002059063.1)

1 mppaehhvat pqaagnddfq leastsaekw kdvpdkyllv iahllaghsn etedmerfng
61 pilkasiksv ywlfliqyaa lallgvilni aiivyimyhr lykdvthafi inlafchfvq
121 calvlpislm vmllqnwifg qflcfflpml qdiplhvami shiliawdrm rwlndplkgr
181 ipgfvcccat wltgmvialp ypiytiyvel gdylpqlsgi glcvvnlmdd mqeytrglfl
241 imycgpavll sylyirtsqe lrppdgpfav mmyehrvdlr mrqrn***sstss e***prthsgggv
301 aglsnghgts trsydlysae ldvcrekrkq rnfgsmaatq vvclcplmil rfarlsleet
361 yenqkhfdft ylmfvwvafl ptvifpciya sqilprdeqe rlrgyfrlsa krkpksqrrs
421 nag***tgslqd***d siekdehtnt tsvavnvagq tqppaesqkh ppkgnavslr herspghgls
481 stdrysgaph rqgqsavker drerdrdrer erdrdrerer erererqrdr ererrerggh
541 vlsgrgagda gvvnnlgkhm rgkkhdvkin iisdghgstg sgrkqpp***sss snns***sgrskp
601 aagqarpkpt ieym***svsnns*** vsnltassyc ngnnsallddd sgtsnygdge essvvssvmh
661 ppqrwphvag lrhk**d**i***sfse csstfssst***l erdleiidll erersmdiqe mmqreqqgek
721 vrlsvggvgr qlpdieklya qrspkpkrds gygydkgala lvmpgsdpls msla***sssqpt***
781 ***eyslcs***nlqe hqlhqqaqqr lghlqqqqlq qqqqqlhqhq qldeeevpqd fydsytpggs
841 nnlppavqav qvaapapppp tyqyqytrrl srkssgpgsh hvpgggtarg skrdsfnsln
901 gtdliagafc eldptqplnk maglegrmrr ssprnasfn***s nsars***smksr dyghgsthsa
961 hpeldfreni fael

M 33639 R PA (Genbank Ref # AAF48813) (PA-specific underlined)

```
  1 mitrlyntee dpaycsfiwg snltssvdvl aanatsvfss dlrddfyrdv edprteslre
 61 ycyglvlpii camgiignvl nlvvltrrnm rgtayiymra ystaallaiv faipfgirml
121 vhkdrgqwee fgpafytahl elylgngclg vgvmmllvlt ieryvsvchp gfarpvmgpp
181 gvvvfltcla tvivylpsif rgelikcilg ssdvyvylrr dntiyqqtif yrvykimlev
241 ifklvptlvi gglnmrimmv yrrtcerrrk mvlsrphaqg hghghghghg hghghahghg
301 ylkdddprkf aeerrlflll gstsilflvc vspmailhmt iasevypsfp fqvfrasanl
361 lelinysltf yiyclfsedf rntlvrtikw pwlkgkfchq aehevsaspp atagtvavag
421 tgnghvsifh paipaltltp aepderprca ngvlh
```

M 33639 R PD (Genbank Ref #AGB95518.1)

```
  1 mitrlyntee dpaycsfiwg snltssvdvl aanatsvfss dlrddfyrdv edprteslre
 61 ycyglvlpii camgiignvl nlvvltrrnm rgtayiymra ystaallaiv faipfgirml
121 vhkdrgqwee fgpafytahl elylgngclg vgvmmllvlt ieryvsvchp gfarpvmgpp
181 gvvvfltcla tvivylpsif rgelikcilg ssdvyvylrr dntiyqqtif yrvykimlev
241 ifklvptlvi gglnmrimmv yrrtcerrrk mvlsrphaqg hghghghghg hghghahghg
301 ylkdddprkf aeerrlflll gstsilflvc vspmailhmt iasevypsfp fqvfrasanl
361 lelinysltf yiyclfsedf rntlvrtikw pwlkgkfchq aehenptngp gvpmacftkv
421 drghqkhhit ttsglgrsss i
```

V 33639 R PD-like (Genbank Ref # AGB95518)

```
  1 mitrlyntee dpaycsfiwg anltssndlv lgttansthi yandlrddly advedprtes
 61 lreycyglml pvicalgiig nvlnlivltr rnmrgtayiy mraystaall aivfaipfgi
121 rmlvhkdrgq weefgpafyt ahlelflgng clgvgvmmll vltieryvsv chpgftrpvm
181 gppgvvvflt cfatfiiylp sifrgelikc mltsnnvyvy lrrdnniyqr tifysvykim
241 levifklipt vliaglnlri mlvyrrtcer rrqmvltran yvkdddprkf aeerrlflll
301 gstsilfllc vspmailhmt iasevlpsfp fqvfralanl lelinysitf yiyclfsedf
361 rntlmrtikw pwlksklchq vdetqtikgv pmvrfdkvtl rnhhitttsg igrsssi
```

the *D melanogaster* PB and PC isoforms matches PA; PD differs by an alternative splice but does not introduce a BBS. The only *D virilis* isoform received by Blastp search was orthologous to PD.

M CNMa R PA (Genbank Ref AAF50229.3)

1 mdmeyitsss gnitattead fssslgesnv teynttemda nesagedeem lriaffighf
61 vhqyyipvlc ctgsignils vfvffrtklr klsssfylaa lavsdtcfla glfaqwlnfl
121 nvdiynqnyf cqfftffsyl asfcsvwfvv aftverfiav iyplkrqtmc tvrrakivlf
181 cltlvgclhc lpyiviakpv fmpklnttic dlnseykeql alfnywdtiv vyavpfttia
241 vlntctgctv wkfatvrrtl tmhkmkpqtn ***smpsnssnss*** ggassavasy rlsaslkrqk
301 stgthpsgqh nvanrqtddq eqqqqsqqhq inncqhhcei tqkparrkvq nssqlkvtkm
361 llivstvfvc lnlpscllri eaywetesar nqnstialqy ifhaffitnf ginfvlycvs
421 gqnfrkavls ifrrvssaqr eagn***tqvtvs e***ycrntgtst rrrmmtqhcw nemhelhplk

V CNMa R PA (Genbank Ref # XP_002047272.3)

1 manpyepttm tamsntsqdi sapttttttt tattatvttt lsyttdssrn isidleymsv
61 edeemlhiaf lisdfvnryy vpiicctgsi gnilsvfvff mtklrklsss fylaalaisd
121 tcflcglfmq wlnflnvniy nqnyfcqfft fisylasfcs vwfvvaftve rfiavmyplk
181 rqimctvrra kivllgltla gcvhcvpyil iakpvyspkl ndticdlnts ykeqlalfny
241 wdsivvyavp fttitvlntc tgctvwkfat vrrtltmhkm kpqitnvpan attgggvaaa
301 tyrisaslrr qkstgthpsg qh***svsrqt***eq qqqqqqqrh darsqhhcei tqktgrrkvq
361 nssqlkvtkm llivstvfvc lnlpscllri etywetqtsk tqnttivlqy ifnaffitnf
421 ginfvlycvs gqnfrkavls ifrrvssaqr egi***tqvtvse*** ycrntgtstr rrmmtqhcwn
481 emhelhplr

M CRZ R PA **(Genbank Ref #** AAF49928)

1 medewgsfdr lpsvpsasmd letenevvsn wstlanftrl vagaapeivn ytlnmidvgv

61 gmatdisnls vsttplpaya isnssslaht nsrheappma eqvpehvmdh apqlsrsgll

121 kvyvlavmal fsllgnllti wniyktrisr rnsrhtwsai yslmfhlsia dvlvtwfcii

181 geaawcytvq wlaneltckl vklfqmfsly lstyvlvlig vdrwiavkyp mkslnmakrc

241 hrllggtyil slvlslpqff ifhvargpfv eefyqcvthg fytadwqeqm yatftlvftf

301 llplcilfgt yms***tfrtiss*** ***se***kmfqgskl anystaklpt qtnrqrlihk akmkslrisv

361 viiiaflicw tpyyvmmimf mflnpdkrlg ddlqdaifff gmsnslvnpl iygafhlcpg

421 kggkssgggg nnnayslnrg dsqr***tpsmlt*** ***avtqvd***gtgg ssrqmrafrq qsyyr***sssng***

481 ***t***agpgaapfk eqvgllhvgp gngtpggg***svs*** ***sgat***pqlirk gsallarqps clreqehqqr

541 lllhekpstl vlsydsqrgg vgvgvasgll dnnervssv

V CRZ R P **(Genbank Ref #** XP_032290616.1)

1 meglediaha rlpvgnttvp ihsdlldnay netdsnwstv anftrliiaa asevapnian

61 ytlnmldvgi alateatnae satstampgs nrgteaayai mtnsrlnsst lghinslhel

121 spvaeqvpeh vmdhapqlsr sgllkvyvlt vmalfsllgn lltiwniykt ritrrnsrht

181 wsaiyslmfh lsiadvlvtg fcligeaawc ytvqwlanel tcklvklfqm fslylstyvl

241 vligvdrwia vkypmkslnm akrchrllgg tyilslvlsl pqffifhvar gpfveefyqc

301 vthgfytavw qeqmyatftl vftfllplci lfgtyms***tfr*** ***tissse***kmfq gsklanystt

361 kqlptqtnrq rlihkakmks lrisvviiia flicwtpyyv mmiifmfwnp dkrlgddlqd

421 aifffgmsns lvnpliygaf hlcpgksnks gtgggnnnay slnrgdsqr***t*** ***psmltavtqv***

481 ***d***aaggsarqm rtfrqqsyyr ***sssngt***aggp fkeqvgllqv ggaggsgatp hmmrk***sssvr***

541 ***s***phpnhssai atrhsgclre qeelllqtkp stlvlnydsq rggvgvgvan gnklmtgasl

601 rvdnkecvss v

M ETH R PA (Genbank Ref # AAF47700)

1 mlpqipsyir ttamffcivi mllgvvgnvm vpivivktkd mrnstniflt nlsiadllvl
61 lvctptvlve vntrpetwvl ghemckavpf veltvahasv ltilaisfer yyaiceplka
121 gyvctkgrai licvlawgia alftspilwv aeyklaeyid gssvavcltq aisdwtlaff
181 lmtisvffvv pfvtlvvlyg iiarnlvsnr aamlrarptk pelslkarkq vvlmlgavvl
241 sffvcllpfr vltlwiilst dqtlhdlglv ryysllyfcr imlylnsamn pilynlmstk
301 frrgfkrlcq dagrlllelv tlgrrkedss rgrrgtlslg mgtntntn***tn*** ***ssnatgatss***
361 ***silsrss***nrr csedisrtrl kiemqmpcgs dleamamlqh stlgkgiarr vsdsrlmplr
421 nhqprrhkpq isfdeeslee nkrseakipt kcreklpgia reivnltent l

V ETH R PA-like (Genbank Ref # XP_002058475.1)

1 mlpqipsyir ttamffciii mllgvignvm vpivivktkd mrnstniflt nlsiadllvl
61 lvctptvlve vntrpetwvl ghemckavpf veltvahasv ltilaisfer yyaiceplka
121 gyvctkarai licvlawgia alftspilwv aeyklveyid gssvpvcltq aigvstvgff
181 lmtisvffvl pflmlvvlyg iiarnlvsnq gamlrarptk pelslkarkq vvlmlgavvl
241 sffvcllpfr vltlwiilst eqtlhdmglv ryysllyfcr imlylnsamn pilynlmstk
301 frkgfarlch dvarfllkll tlgrrranpa dsrgr***sgtls*** ***tgt***g***tqtnss*** ***nataat***nssi
361 lsrssnrrgs edisrtrlki elqlpcgsdl eamamlqnst lrskrrqsds rllgekkprr
421 pkpqisfdea avgrapketv ipa

M ETH-R PB (Genbank Ref # AAF47700)

1 mlpqipsyir ttamffcivi mllgvvgnvm vpivivktkd mrnstniflt nlsiadllvl
61 lvctptvlve vntrpetwvl ghemckavpf veltvahasv ltilaisfer yyaiceplka
121 gyvctkgrai licvlawgia alftspiiai stysvepygd gtdapvctta adgfwsifyf
181 vgcitvfffl pfgilvllya aiaykllrpn nafhrptspq pqqpsggats gssqvpstkg
241 nshqqsngmr khrkqvifml vavvssffvc llpfraftlw vilasaedve glgiagyynl
301 lyfsrfmlyl nsamnpilyn lmsskfrsgf wrllltclgq rphhhhrhhy hqrqhptagg
361 sgrnastrqe qdaeegaala gttsarhprr tlrreatfli nsi***stssgtd*** ***rttss***sawrs
421 nsl***sisgls***e rergilgaai igt***taatvtt*** aclqerrask i

V ETH R PB-like (Genbank Ref # XP_015024858.1)

1 mlpqipsyir ttamffciii mllgvignvm vpivivktkd mrnstniflt nlsiadllvl
61 lvctptvlve vntrpetwvl ghemckavpf veltvahasv ltilaisfer yyaiceplka
121 gyvctkarai licvlawgia alftspiiai ssysvepygd gteapvctta adgfwpilyf
181 vgtitlfffl pfgillllya aiaykllrpn safhrptspq gp***tptaat***aa snkgnshqqs
241 ngmrrhrkqv ifmlvavvas ffvcllpfra ftlwvitass edverlgiag yynllyfsrf
301 mlylnsamnp ilynlmsskf rsgfwrllls clgrrphhhr hhhhyptiae rslrqtqrrq
361 dhepteavpg tdnaqrprrt lrreatflin si***stssgte***r h***tsstwrsns*** ***lst***slglger
421 erdrgalgaa iigt***taatvt*** ***t***aclqerras kt

M FMRFa R PA (Genbank Ref # AAF47700)

1 msgtavarll lrlelpspgv mppptdydy ggpisddefl asamategpt vrydlfpqnn

61 sqptlqivln htevqtdlqy phyedlgldp dpnwtriced vynpllennr iefwvcgvli

121 nivgvlgilg niismiilsr pqmrssinyl ltglarcdtv liitsillfg ipsiypytgh

181 ffgyynyvyp fispavfpig miaqtasiym tftvtleryv avchplkara lctygrakiy

241 fivcvcfsla ynmprfwevl tvtypepgkd vilhcvrpsr lrrsetyini yihwcylivn

301 yiipfltlai lncliyrqvk ranrerqrls rsekreigla tmllcvvivf fmlnflplvl

361 niseafysti dhkitkisnl litinssvnf liyiifgekf krifllliffk rrlsrdqpdl

421 ihyessisnn gdgtlnhrss grfsrhg***tqr stttt***ylvat ggpggggcgg gggnnslnnv

481 rltqvsgspg lvkikrnrap spgpvvyfpa remqr***sastt ns***ttnnntsi gydwtlpdsk

541 klghvssgf

V FMRFa R PA (Genbank Ref # XP_002047744.1)

1 msaaaarlll rleqpvpgvm pppapaeydw spisvdqfla satgrsqeda vryeladplg

61 stlapliydv gqrllfngss gtqngndtyt dlfpqmedmg depnwtrice evynpqlenn

121 riefwvcgvi lnivgilgil gnvismiils rpqmrssiny lltglarcdt mliissmllf

181 gipsiypytg qffgyynyvy pfispamfpi gmiaqtasiy mtftvtlery vavchplrar

241 alctygraki yfivcvcfal aynmprfwev ltvsyqlpns tdvlhcvrps plrrnptyin

301 iyihwcyliv nyiipfltla ilncliyrqv kranrerqrl srsekreigl atmllcvviv

361 ffmlnflplv lniseafysv idhkvtkvsn llitinssvn fliyiifgek fkrifllliff

421 krrlsrdqpd lihyessisn ngdgtinhrs sgrfsrhg***tq rsttt***tylva tgggggnssl

481 nsvrltqvsg spgllkikrn rapspgpvvy ypgpremhr***s vstsns***ttnn ntalgydwtv

541 dgkklghvss gf

M LGR1 PA (Genbank Ref # AAF55460)

```
  1 mekhpslsqr mgttyrprkg lkclsfefqc rlllhhlllt slsgrhfvya tsavggalsa
 61 nnchdihhgf dvypnltavs laqstdtplt atmprsawkc ccwnasnqae evecrcegdg
121 lnrvpqtltl piqrltiasa glprlrhtgl kvygstlldv aftdclqlel iqdgafanlt
181 llrtiyitna pkltflskdv flgisdtvdi iriinsgltr vpdlghlpph nilqmidldn
241 nqitridsks ikvktaqlil tnneisyvdd saffgskiak lslkenkklq mmhpnafdgi
301 iditeldlss tslvglpsag lqniealyiq nthtlktips iynfrnlqra ylthsfhcca
361 fqfpsrhdpq rhaqrmleie kwrkqcksds gtrkerstld npfnmpedfg sfggtddsat
421 ditpitfasf dymaddtmnk gtfhekiiln pgddssaelc gnftfrkpni ecypmpndln
481 pcedvmgyqw lrisvwivva lavvgnvavl tvilsirpes tpvprflmch lafadlclgl
541 ylllvacida hsmgeyfnfa ydwqyglgck vagfltvfas hlsvftltvi tierwlaitq
601 amylnhrikl rpaalimlgg wiysmlmssl plfgisnyss tsiclpmenr dvydtiylia
661 ilgsngvafs iiavcyaqiy lslgretrqa hqnspgelsv akkmallvft nfacwspiaf
721 fgltalagyp linvtkskil lvffyplnsc adpylyailt sqyrqdlftl lsklglcqqs
781 alkykdslsg qattrftihg siqrhssltc kmqtvmgaet qkmlknsedy v
```

V LGR1 PA-like (Genbank Ref # XP_032289845.1)

```
  1 mkcmviisql dfltfillsl ahgtrqantn chdnnngfnt vfnnlsidng netdmsvimt
 61 qaptmstnpt sdasvwkccc weatnqnefe crcegealtr vpqtlklpll rltiasaglp
121 rlrsmglkvy attlldvafi dclqleaiqn gafsnltflr tiyisnapkl tylpknvfeg
181 isdtieiiri insgltsvpd fgylppnnil qmidldnnqi sridsksiqv ktaqfvlann
241 dihfiddsaf lgskiaklsl kdnrrltdvh pnafygiidi teldlsstsl vslpsaglqt
301 vevlyiinth tlktipsiyn fqnlqrahlt hsfhccafqf psrhdpkrha erlqelqkwr
361 eqcnverdly rdvdkklkkn tsvkdtagvh ytnqsgtatd nlltdaasns ydymadstmn
421 nigifheqit inpddnqlae ycgnftfrnp diqcfpmpna lnpcedvmgy qwlrisvwiv
481 valaivgnla vltvtlsiks espsvprfli chlafadlcl glylllliasi dahsmgeyfn
541 yafdwqyglg ckiagfltvf ashlsiftlt iitierwfai thamylnkri tlrqaagiml
601 tgwiysiims slplfgisny sstsiclpme irdiydsiyl ililgcnfva ftiiaicysq
661 iylslgqetr rarrnnpgem svakkmlllv finftcgapi affgltalag cplinvtksk
721 illvffypln scadpylyai ltsqyqqdll tflsklgicr qnalkykhsd slhgtshyti
781 rgsieqqssl cqkpqqegaa etqtmlknne dyv
```

M LGR3 PA (Genbank Ref # AAF56490)

1 mvygrsiavg fclmtvvlll aavifylslg pcpaasfacd ngtlcvprrq mcdsrndcad

61 ssdenpvecg llygskeiad kivrnaiekk qqrlisavsn asgadsttsm vprnqsltln

121 mtcdivtypk acqcgqgtil ycgryaklrr fprlssevtn liiirnnltl rdnifanftr

181 lqkltlkynn isrvplgsfs glfhlerlel shnnvshlph gvflglhslq wlflvnnhlh

241 hlpveqlrff rrlewlvlsr nrltlrnvql pkiptlyevy ldfnrieyig eetfsqldnl

301 hlldlqhnli thihgrafan ltnmrdirlv gnpikelsge tflhntrlea lslalmpihi

361 ssslmeplni sflnltgiry dhidfeains mrnltyiiyd rffycsmtpr vrmckpstdg

421 vssfqdllsk pvlrysawvm atltiagnvl vlwgrfiyrd envavtmvir nlaladmlmg

481 fylvtigvqd yryrneyykv vldwitswqc tligtlavss sevsmlilaf mslerfllia

541 dpfrghrsig nrvmwlalic iwitgvglav apvllwrtst lpyygsysgt cfplhiheaf

601 pmgwlysafv flgvnllllv miamlytall isiwrtrsa***t pltlld***cefa vrfffivltd

661 flcwvpiivm kiwvffnyni sddiyawlvv fvlplnsavn pllytfttpk yrnqiflrgw

721 kkitsrkrae agngnvattt tg***tatgss***qh pddftifaka amrch

V LGR3 PA (Genbank Ref # XP_002056481.1)

1 mvygrsiavg icfmtvvvvl sgliyylslg pcptgsfacn ndiqcvpqrq mcdkhldced

61 gsdenpvecg nlygskelad kivrnaiekk kqqqqqqen qrlmivtsns sawdspslag

121 srnqsmpipc aittyprqcq cgggtmlycg rfaklrrwpr iseevtnlfi irnnvtlren

181 lfanltrlqk ltlkhnnisr lppgcfsglp hlerlelghn nfsqlphfil kdlpalqwlf

241 lvnnqlrsfp mdhlaamhkl ewlvlshnrl tlrneqlsks pklveifldn nrieyigekt

301 faqldnlell dlqynlithi hvkafanlta isdirlignp ikelsgetfl ynthlealsl

361 aymplpidrn lmkslnvsfl nltgieferi dfaalneirn lkyivfdrfy ycsmtphvlm

421 ckpksdgvss lkdllskpsl rhstwfmatl siagnlmvlw grfiyrdenv avtmvirnla

481 ladllmgfyl liigvqdfrf rdeyhkvard wisswqckai gtlavsssev smlilafmsl

541 erflliadpf rghraisvri iyfsllciwv tgvglaitpv viw***sssttd***f ygthsgtcfp

601 lfiheayplg wqysafvflg vnlllllmia llytallisi wrtrsa***tpls lld***cefavrf

661 ffivltdvlc wapiiamkiw vffkynisdd iyawlvvfil plnsavnpll ytfttpkyrn

721 qillrgwkki tsrkrtetgh gnvantttg***t atgss***qnpde yttatgiksm plaltlsh

M LGR4 PB (Genbank Ref # ABW09404)

1 mciahlpitf tlaillaias negaqgvesa trtaieairt gigtkpetei adateaeapv
61 revisllgii dgaesdilvp daddkcpggy fhcnttaqcv pqrancdgsv dcddasdevn
121 cvnevdakyw dhlyrkqpfg rhdnlrigec lwpnenfscp crgdeilcrf qqltdiperl
181 pqhdlatldl tgnnfetihe tffselpdvd slvlkfcsir eiashafdrl adnplrtlym
241 ddnklphlpe hffpegnqls ililarnhlh hlkrsdflnl qklqeldlrg nrignfeaev
301 farlpnlevl ylnenhlkrl dpdrfprtll nlhtlslayn qiediaantf pfprlrylfl
361 agnrlshird etfcnlsnlq glhlnenrie gfdleafacl knlssllltg nrfqtldsrv
421 lknltsldyi yfswfhlcsa amnvrvcdph gdgissklhl ldnqilrgsv wvmasiavvg
481 nllvllgryf yk***srsnve***hs lylrhlaasd flmgiyltli acadisfrge yikyeetwrh
541 sgvcafagfl stfscqsstl lltlvtwdrl msvtrplkpr dtekvrivlr llllwgisfg
601 laaapllpnp yfgshfygnn gvclslhihd pyakgweysa llfilvntls lifilfsyir
661 mlqairdsgg gmr***sthsgre*** nvvatrfaii vttdcacwlp iivvklaals gceispdlya
721 wlavlvlpvn salnpvlytl ttaafkqqlr rychtlpscs lvnnetrsq***t qtaye***sglsv
781 slahlgggvg ggsgrkrmsh rqmsyl

V LGR4 PB (Genbank Ref # XP_015025567.1)

1 mtimmmplls lgalvallsl tsmtlasity sveqprgtir ssvgqgntmf mlgssvvdae
61 ademdeldsg illpdekdnc pagyfhcntt aqcvpqranc dgsvecddrs dewncvnevd
121 akywdhlfrk ssfgrqgmdd kpigvcywpn aknfscpcrg neilcrfqql talppqlptn
181 nlstldltgn nfgvidetff gglpaveslv lklcaireia shafdrlaai plktlymddn
241 elsrlpehff apgnqlrili larnqlssls sgdfrylqhl edldlrgnli shfeaqvfaq
301 lhslevlyln nnrlqqlrpg mfpsnlvhlh tlslahnqis siaantftfp qlrhlflagn
361 qlsyirdgtf cnlsylqglh lnenqiekfd lhafdclenl ssllltgnrf ktlepavlqn
421 lssldfiyfs wfhlcraalh vrvcdprgdg isstfhlldn qllrgsvwim asiavvgnll
481 vlagryfyk***s rsnve***hslyl rhlavsdflm giyltliaca disfrgqyir heeswrhsgf
541 cafagflstf scqsstlllt lvtwdrlmsv trplkprdte kirivlrlll vwgisfglaa
601 apllpndyfg ahfygnngvc lslhihdpya kgweysallf icintislif ilfsyvrmlq
661 airdsgggmr ***sthsgre***svv atrfaiivtt dcacwlpiiv vkvaalsgca ispdlyawla
721 vlvlpvnsal npvlytltta afkqqlrryc htlptcslgn netrsq***tqtg ye***sglsvsla
781 hlgggggagg giggsgrkrm shrhmsyl

M LK R PA (Genbank Ref # ABW09404)

```
  1 mamdlieqes rleflpgaee eaeferlyaa paeivallsi fyggisivav igntlviwvv
 61 attrqmrtvt nmyianlafa dviiglfcip fqfqaallqs wnlpwfmcsf cpfvqalsvn
121 vsvftltaia idrhraiinp lrarptkfvs kfiiggiwml allfavpfai afrveelter
181 frennetynv trpfcmnknl sddqlqsfry tlvfvqylvp fcvisfvyiq mavrlwgtra
241 pgnaqdsrdi tllknkkkvi kmliivviif glcwlplqly nilyvtipei ndyhfisivw
301 fccdwlamsn scynpfiygi ynekfkrefn krfaacfckf ktsmdahert fsmhtrassi
361 rstyanssmr irsnlfgpar ggvnngkpgl hmprvhgsga nsgiyngssg qnnnvngqhh
421 qhqsvvtfaa tpgvsapgvg vamppwrrnn fkplhpnvie ceddvalmel psttppseel
481 asgagvqlal lsressscic eqefgsqtec dgtcilsevs rvhlpgsqak dkdagkslwq
541 pl
```

V LR R PA (Genbank Ref # XP_002047380.1)

```
  1 meadiiynqh iheellpgae eeaeferlya apaeivalls ifyggisiva vigntlviwv
 61 vattrqmrtv tnmyianlaf adviiglfsi pfqfqaallq rwnlpyfmcg fcpfvqalsv
121 nvsvftltai aidrhraiin plrarptkfi skfiiagiwl lalvfaapfp iafrveeitd
181 rfrendvyfn ltrpfcmnkn lsdeqlkayr yalvfvqylv pfcvisfvyi qmavrlwgth
241 apgnaqdsrd itllknkkkv ikmliivvvi fglcwlplql ynilyvtipe indyhfisiv
301 wfccdwlams nscynpfiyg iynekfkref nrrfaacfck fktslepher tfsmhtrass
361 irstyanssm rirnnffavr nagngktgln igrsayqang ngtgtgianv netssynnng
421 hqtvvtfags gstmppwrrn qfkplhpnva eceddlalme lpstneeapp smaeagvpla
481 lfkaesress sciceqefgs rtecdgtcil sevsrvqqpa tkgpdgvggi gsgseseilw
541 qpl
```

M moody PA (Genbank Ref # NP_569970.2)

1 msdettisle dgypplealt tmvppadatg fsqslltfaa vmtflimivg icgnlltvva
61 llkcpkvrnv aaafiislci adllfcalvl pfqglrfvqg twrhgqvlcr lipfiqygni
121 gvsllciami tinryvmith hglyariykr hwiavmiaac wlfsygmqlp tllgewgrfg
181 ydsrlqtcsi mtddhghssk ttlfitafvi pclviiacya kifwvvhkse qrlkrhatkq
241 nsipnnlrpl astgsgalps gaecqpsnrv ssdssssfsi dvpetapsgk qqptrvkdqr
301 evrakrnewr itkmvlaifl sfvvcylpit ivkvadknve hpslhicsyi llylsacinp
361 iiyvimnkqy rkayktvvfc qparlllpfg ktngassaae kwkdtglsnn h***srtivsqms***
421 ***ggt***gaasgag tatgtaavav mqtppevqqa qalemvsrgp dlisksnlpq pnvtppppsv
481 ltatpng***sns*** ***nslt***lrlplk knnhcy***tnsg*** ***fns***stpspss glgigissss iyrpgvgslg
541 sgsasirrit mvgddiilee eelpptppat sapttpappp pssplhpl***st*** ***dsstttis***gg
601 avvagssapk patptphiym nvdspkrnqy ymdrntnava pesdsgpant satvsisgsk
661 ltakmkfpkd

V moody PA-like (not annotated; obtained by direct inspection of genomic DNA – see SXX Document – "de novo moody isoform annotations"))

1 mnddttasps pglghgvddm ltaaptgtas gldavdesgf sqslltfaai mtflimivgi
61 cgnfltvval lkcpkvrnva aafiislcia dllfcavvlp fqglrfvqgt wrhgpvlcrl
121 ipfiqygnig vsllciamit inryvmithh gcyariykrh wiaimiaacw lvsygmqlpt
181 llgawgrfgy darlqtcsim sdahghsskt tlfitafvip clviiacyak ifwvvhkseq
241 rlkrhankqn sipnnlrpva aaspagqdgs vqptaaasrv ssdsssystd vpegkqpppq
301 srvkdqrevr akrnewritk mvlaiflsfv icylpitivk vadkdvehps lhifsyimly
361 lsacinpiiy vimnkqyrka yktvvlcqpa rlllpfgktn gassaaekwk dtglsnnh***sr***
421 ***tlvsqms***aga aaaaaaaaaa avaaasastp ppspasdcqs lpatkaspqs lelmsrcpdl
481 inrtnqpplv tpppp***svtla*** ***t***aqp***sssnss*** ***nssnsggs***rl plkksnhsyi nngfns***sghs***
541 ***qns***avyrpaa gsppsaaaap lrritmvgdd iileeeelpa vpsp***tatals*** ***vskvt***kspiy
601 mnvnspkrnq sysdkasvkd alqqasvpsd qgkdqqdask vpikfpnpkd

M moody PC **(Genbank Ref #** NP_001188535.1)

1 msdettisle dgyppleal t tmvppadatg fsqslltfaa vmtflimivg icgnlltvva
61 llkcpkvrnv aaafiislci adllfcalvl pfqglrfvqg twrhgqvlcr lipfiqygni
121 gvsllciami tinryvmith hglyariykr hwiavmiaac wlfsygmqlp tllgewgrfg
181 ydsrlqtcsi mtddhghssk ttlfitafvi pclviiacya kifwvvhkse qrlkrhatkq
241 nsipnnlrpl astgsgalps gaecqpsnrv ssdssssfsi dvpetapsgk qqptrvkdqr
301 evrakrnewr itkmvlaifl sfvvcylpit ivkvadknve hpslhicsyi llylsacinp
361 iiyvimnkqy rkayktvvfc qparlllpfg ktngassaae meryrveqqp qphnrlpdvg
421 gngsrigsrd gngngsgggd adptggpt***sp slgd***gvagtg pnkqieppaa eryasatlgp
481 hgdtqrkqqq qphpataaqe eqsllhqqrl qqqhsqpqqr lgdrdqqqlh lparswltge
541 rlrldpshnn gggrhntggr gaaahasgdf cadnagtspt liptasavhg linnnnqrrs
601 sgsgeqctka gdanaphlhe crqpeekpil hgs

V moody PC-like **(Genbank Ref #** XP_002057362.1)

1 mnddttasps pglghgvddm ltaaptgtas gldavdesgf sqslltfaai mtflimivgi
61 cgnfltvval lkcpkvrnva aafiislcia dllfcavvlp fqglrfvqgt wrhgpvlcrl
121 ipfiqygnig vsllciamit inryvmithh gcyariykrh wiaimiaacw lvsygmqlpt
181 llgawgrfgy darlqtcsim sdahghsskt tlfitafvip clviiacyak ifwvvhkseq
241 rlkrhankqn sipnnlrpva aaspagqdgs vqptaaasrv ssdsssystd vpegkqpppq
301 srvkdqrevr akrnewritk mvlaiflsfv icylpitivk vadkdvehps lhifsyimly
361 lsacinpiiy vimnkqyrka yktvvlcqpa rlllpfgktn gassaaekwk emeryrieqq
421 pqphpclpdv rrrssssssgg gggggcsgig vdaaavarir lpvlagdqsv a***svtgad***vpm
481 ***srsnksnes***t aigyapaaig nagngsteqq qqqqqqqqrr qsaaaqeeqs llhqqrlqqq
541 raqpeqrrls arswlpavgs ggaiashhhg ggryhtgggg ***tarsaqsdgd*** gavsqqsnqi
601 ahlherqqse ekpil

M Ms R1 PA (Genbank Ref # AAF47635.2)

```
  1 masgnnetep lycgsgmdnf htsyknmhgy vslvvcilgt iantlniivl trremrsptn
 61 ailtglavad lavmleyipy tihdyiltds lpreeklsys wacfikfhsi faqvlhtisi
121 wltvtlavwr yiavgypqkn rvwcgmrtti itittayvvc vlvvspslyl itaiteyvdq
181 ldmngkvins ipmtqyvidy rnellsarta alnatptsap lnetvwlnas tlltstttaa
241 pptpspvvrn vtvyrlyhsd lalhnaslqn atfliysvvi klipcialti lsvrlilall
301 eakrrrkklt skpatpgasn gtkspangka adrprknskt lekekqtdrt trmllavlll
361 flitefpqgi mgllnavlgd vfylqcylrl sdlmdilali nssinfilyc smskqfrttf
421 tllfrpkfld kwlpvaqdem aaaraersav apvlekgrqq pqvvmasttt nitqvtnl
```

V MS R1 PA-like (Genbank Ref # XP_002047415.1)

```
  1 maggsndttp lycgtgtdnf htsyknmhgy vslvvcilgt iantlniivl trremrsptn
 61 ailtglavad lavmleyipy tvhdyilkds lpreqklsyg wacfikfhsi faqvlhtisi
121 wltvtlavwr yiavghpqkn rvwcgmrtti itittayvvc vlvvspslyl itaiaefmdq
181 mdvegnliss ipmsqyvidy rnemqakmsv avnatptspt anvtqwlnms vlatspmppt
241 pvppaslvvr nvtvyrlyhs dlalhntslq tatfliysvl iklipcialt ilsvrlimal
301 leakrrrkkl tskpaasngt ktlvngksae rprknsktle kekqtdrttr mllavlllfl
361 itefpqgilg llnsvlgnef lmqcylrlsd lmdvlalins sinfilycsm skqfrstftl
421 lfrpkfldkw lpvaqdelaa sradglqrsv vaavpdkyqs thkqpqvvtl aattttnnat
481 evtnl
```

M MsR1 PB (Genbank Ref # AAF47635.2) (stop suppression)

```
  1 masgnnetep lycgsgmdnf htsyknmhgy vslvvcilgt iantlniivl trremrsptn
 61 ailtglavad lavmleyipy tihdyiltds lpreeklsys wacfikfhsi faqvlhtisi
121 wltvtlavwr yiavgypqkn rvwcgmrtti itittayvvc vlvvspslyl itaiteyvdq
181 ldmngkvins ipmtqyvidy rnellsarta alnatptsap lnetvwlnas tlltstttaa
241 pptpspvvrn vtvyrlyhsd lalhnaslqn atfliysvvi klipcialti lsvrlilall
301 eakrrrkklt skpatpgasn gtkspangka adrprknskt lekekqtdrt trmllavlll
361 flitefpqgi mgllnavlgd vfylqcylrl sdlmdilali nssinfilyc smskqfrttf
421 tllfrpkfld kwlpvaqdem aaaraersav apvlekgrqq pqvvmasttt nitqvtnlxh
481 rrsrgrrtll srllsvlkrg rrrssgeggg vggggaplag ndavepafqa ivvvvdkvsg
541 atenqlytae qarivt
```

M Ms R2 PA (Genbank Ref # AAF47633)

```
  1 mvtnmsqphy cgtgiddfht nykyfhgyfs livcilgtia ntlniivltr remrsptnai
 61 ltglavadla vmleyipytv hdyilsvrlp reeqlsyswa cfikfhsvfp qvlhtisiwl
121 tvtlavwryi avsypqrnri wcgmrttlit iataywcvl vvspwlylvt aiakfletld
181 angktiasvp lsqyildynr qdevtmqvms sttpdvswai psdsangtav sllslttvip
241 lttlstgvtt ssslgernvt vyklyhsala lrdrqfrnat fliysvlikl ipcfaltils
301 vrligallea krrrkilach aandmqpivn gkvviptqpk sckllekekq tdrttrmlla
361 vlllflvtef pqgimgllnv llgdafflqc ylklsdlmdi lalinssinf ilycsmsrqf
421 rstfallfrp rwldkwlpls qhdgegrvgg sgglggyggy grqrllhtda vsksmaidlg
481 lttqvtnv
```

V MS R2 PA (Genbank Ref # XP_015031310.1)

```
  1 mvtnmsqphy cgasvddfht nykyihgyfs livcilgtla ntlniivltr remrsptnai
 61 ltglavadla vmleyipytv hdyilsarlp reqqlsyswa cfikfhsifa qvlhtisiwl
121 tvtlavwryi avsypqrnri wcgmrtttit iataywcvl vvspwlylvs siakfletld
181 adgktirsvp lsqyildynn eehitmqvls sttpdmawsl apgssnttav nllgpftdlp
241 tttmttttvp ydvgernvtv yklyhselal hdrplrnatf liysvlikli pcfaltvlsv
301 rligalleak krrkilacha andmqpivng kvvtpsqpks ckllekekqt drttrmllav
361 lllflitefp qgimgllnal lgdaflmqcy lklsdlmdil alinssinfi lycsmsrqfr
421 stftllfrpr wldkwlplsq hdgdardgag grldgygrqr lvhtdavsks maidlglttq
481 vtnv
```

M MsR2 PB (GenbankRef # AAN12219) **(stop suppression)**

```
  1 mvtnmsqphy cgtgiddfht nykyfhgyfs livcilgtia ntlniivltr remrsptnai
 61 ltglavadla vmleyipytv hdyilsvrlp reeqlsyswa cfikfhsvfp qvlhtisiwl
121 tvtlavwryi avsypqrnri wcgmrttlit iataywcvl vvspwlylvt aiakfletld
181 angktiasvp lsqyildynr qdevtmqvms sttpdvswai psdsangtav sllslttvip
241 lttlstgvtt ssslgernvt vyklyhsala lrdrqfrnat fliysvlikl ipcfaltils
301 vrligallea krrrkilach aandmqpivn gkvviptqpk sckllekekq tdrttrmlla
361 vlllflvtef pqgimgllnv llgdafflqc ylklsdlmdi lalinssinf ilycsmsrqf
421 rstfallfrp rwldkwlpls qhdgegrvgg sgglggyggy grqrllhtda vsksmaidlg
481 lttqvtnvxq essgraamsa aaggaaasva lalaatdvdg cppatdaavs tndislvekl
541 hlqpsprgta issgqhrrrr sgsgtkciwp ttdwlrklrn qkareteqss eqdielgkss
601 inrrssvllm vllsssdevk akavlvseqp pspadedved aidalwl
```

M NPF R PA **(Genbank Ref #** AAF51909)

1 miismnqtep aqladgehls gyasssnsvr ylddrhpldy ldlgtvhaln ttaintsdln

61 etgsrpldpv lidrflsnra vdspwyhmli smygvlivfg algntlvvia virkpimrta

121 rnlfilnlai sdlllclvtm pltlmeilsk ywpygscsil cktiamlqal cifvstisit

181 aiafdryqvi vyptrdslqf vgavtilagi walalllasp lfvykelint dtpallqqig

241 lqdtipycie dwpsrngrfy ysifslcvqy lvpilivsva yfgiynklks ritvvavqas

301 saqrkvergr rmkrtnclli siaiifgvsw lplnffnlya dmerspvtqs mlvryaichm

361 igmssacsnp llygwlndnf rkefqellcr csdtnvalng httgcnvqaa arrrrklgae

421 lskgelkllg pggaqsgtag gegglaatdf mtghhegglr saitesvalt dhnpvpsevt

481 klmpr

Dm NPFR isoforms do not differ by BBS, only by alternative CT sequences and a small exon

V NPF R **(Genbank Ref #** XP_002058565.3)

1 miiamnrtef gtlpqfessa eifkaiarns nfdligerrh lanfpslhtg dvsssssssi

61 snsisnninn ssnsnsssnn ishsnmtill lnstnnesnf mpadmdpvlm dqylhnraie

121 spwyhlliam ysvlivfgam gnimvviavv rkpimrtarn lfilnlaisd lllclvtmpl

181 tlmeilskfw pygscavlck tiatlqalsi fvstisitai afdryqvivy ptrdslqfvg

241 availagiwi laltlasplf iykqlismdm ppvlqrlgvp hrisyciedw plsdgrfyys

301 ifslcvqylv pilivsvayf giynklk***sri tvvtvqs***ssq rkvergrrmk rtnrllisia

361 iifgvswlpl nffnlyadlq hpsavtqrml vayaichmig mssacsnpll ygwlndnfrk

421 efqellcrst estnvalhgh ttgsnmqaaa arrrrkhelq llgnsvqrga sdgdclggms

481 iaatefntrh tvng***trsavt*** esvaltespm psemttlvpr

M Pk1 R PD (Genbank Ref # AAX52950)

1 msagnmshdl gpprdplaiv ipvtvvysli fitgvvgnis tcivikknrs mhtatnyylf

61 slaisdflll lsgvpqevsy iwskypyvfg eyicigrgll aetsanatvl titaftvery

121 iaichpflgq amsklsrair iivlvwimai vtaipqaaqf giehysgveq cgivrvivkh

181 sfqlstfiff lapmsiilvl ylligvhlyr stlvegpasv arrqqlksvp sdtilyrygg

241 ***sgtams***fngg gsgagtaglm ggsgaqlssv rgrlnhygtr rvlrmlvavv vcfflcwapf

301 haqrliaiya pargaklrdq hefvytvmty vsgvlyylst cinpllynim shkfreafka

361 vlfgkkv***skg slns***rnnies rrlrral***tns sqt***qrfsies aeqpkpsimq nptnkppvaa

421 qyamigvqvn

V Pk1 R (Genbank Ref # XP_002054764.2)

1 mspastppai nqsaatitql lgaqrdplai vipvtvvycl ifltgvvgni stcivikknr

61 smhtatnyyl fslaisdfml llsgvpqemy fiwskypyvf geyfcigrgl laetsanatv

121 ltitaftver ymaichpflg qamsklsrai riivliwlla vltaipqaaq fginsyagvd

181 kcvvvrvivq hsfqlstfif ffapmsiilv lylciglhly rssvigggsp tapvtessar

241 rqqplkavas dtilyryags ssvlannagp qlssvrgrlt hygtrrvlrm lvavvicffl

301 cwapfhaqrl iaiyaparga qlhdqhelly tvmtyvsgvl yylstcinpl lynlmsnkfr

361 eafkavllgk kyskgmqnsr hqlesrrlrr ***tttlnsstqr dsieskht***lm qtalnerllq

421 tksqlvl

M Pk2 R1 PA (Genbank Ref # AAF54930)

1 mlqqgvaitia ndsnddginq sfmahvspsp nqspsigvgi giasstmanp sespemlllk

61 ndkflthvah llnittenls nllgstngtn astmaadspv desltlrtal tvcyalifva

121 gvlgnlitci visrnnfmht atnfylfnla vsdlillvsg ipqelynlwy pdmypftdam

181 cimgsvlsem aanatvltit aftveryiai chpfrqh***tms kls***raikfif aiwlaaflla

241 lpqamqfsvv yqnegysctm endfyahvfa vsgfiffggp mtaicvlyvl igvklkrsrl

301 lqslprrtfd anrglnaqgr virmlvavav afflcwapfh aqrlmavygl nlinigisrd

361 afndyfrild ytsgvlyfls tcinpllyni mshkfreafk itltrqfgla rnhhhqqsqh

421 hqhnysallr qngsmrlqpa scsvnnnale pygsyrvvqf rcrdanhqls lqd***sirtttt***

481 ***tttins***nsma agngvgggag gggggrrlrk qelygpgpgt avphrmlqaq vsqlsslgda

541 nslleaevvd rhyasgrakr allatksgal lvtppqsgdp sevsqpatrl kltrvisrrd

601 evantstppf cgshslpdpe tcqsasvagr ssrkfpwrkr rqktedpsse gltygspksq

V PK2 R1 PA-like (Genbank Ref # XP_002056087.1)

1 mmqgvdfala sdnddglnqs fmahmlpnar pslrpstsrs psatsspell llhndkflth

61 vaqmlnmtte nltnllaans tngssatati tattsttnma nsteesptll iilticyali

121 fvagvlgnli tcivisrnnf mhtatnfylf nlavsdlill vsgipqelyn lwcpnsypft

181 dgicivesvl semaanatvl titaftvery iaichpfrqh ***tmskls***raik fifaiwlaaf

241 llalpqamqf svvnqdngys ctmennfyah vfavsgfiff cgpmtaicvl yvligmklkr

301 srllqslprr aydanrglna qsrvirmlva vavafflcwa pfhaqrlmav ygvslinacr

361 crdafndyfh ildytsgvly flstcinpll ynimshkfre afkitltrqf glarnhqqqs

421 qyrqhnysal mrlqgsmrlq pvscsnnnna lepygsyrvv qfrcrdanhq lslqd***sirtn***

481 ***tttttins***ss lagagaasvp gsgggcggsg vgggnggasg rrlrkqefya tapgsavphr

541 llq***sqvsrls*** dgn***shslld***t nvadvgrhca agrakrtlla tnngalllat aqpddaqppt

601 rlklsrvisr rdepaysgss slpeqe***tnns tnt***tdpngge lrkfpwrkkr qkqnvannne

661 tgggrngyat pksl

M Pk2 R2 PA (Genbank Ref # AAF54929.2)

1 mavkmlptns sgvlatdlql fhnekfllnl tqvlnisadn ltsllqglep eellptvtpm

61 tplsllatls vgyalifiag vlgnlitciv isrnnfmhta tnfylfnlai sdmillcsgm

121 pqdlynlwhp dnypfsdsic ilesvlseta anatvltita ftveryiaic hpfrqh***tmsk***

181 ***ls***ravkfifa iwiaallal pqaiqfsvvm qgmgtsctmk ndffahvfav sgflffggpm

241 taicvlyvli gvklkrsrll qalprrcydv nrgisaqtrv irmlvavava fficwapfha

301 qrlmavygst sgiesqwfnd vfsildytsg vlyflstcin pllynimshk freafkvtla

361 rhfglggknq grglphtysa lrrnqtgslr lh***ttdsvrtt mtsmatttt***g lngsangsgn

421 g***tttgqs***vrl nrvsldsvqm qgqnrsrqdl fdnprrmlq***t qisqlss***vgd ahslleedlq

481 fpgeplqrqp tmcsideltd dlaisrsrlk ltritrppgg vtggvaggst tgaagsggvs

541 gdessgkvrk akvkvlksss pfkglrtkfn wrarrkgshk phekgatvng gdteeraaf

V PK2 R2 PA-like (Genbank Ref # XP_032289636.1)

1 msammlatnv salgegtppl selqmfhsek fllnltqvln isadnltsll qelephdlle

61 qsprpmathm gllatlsvgy alifvagvlg nlitcivisr nnfmhtatnf ylfnlaisdl

121 illcsgmpqd lynlwhpnny pfsdgicile svlsetaana tvltitsftv eryiaichpf

181 rqh***tmskls***r aikfifaiwi aalllalpqa iqfsvvtqga gssctmnndf fahvfavsgf

241 lffggpmtai cvlyvligik lkrsrllqal prrsydvnrg isaqtrvirm lvavavaffi

301 cwapfhaqrl mavygstfgi esqwfndvfn ilnytsgvly flstcinpll ynimshkfre

361 afkvtlarqf rlsgkhqgqg lphnysalrr nqtgslrlh***t dsvrttttlt t***lngssngaa

421 vggcrssqrl qrgsl***sssha t***llstq***srsr qd***lfaarvva adaaattaag aaaaatagaa

481 ahsqphr***tlq tqisqlss***vg dahslleael qlpeehyela arhkcphaam gaidelscqs

541 svvggaaaag lsrarlkltr itrhpqmqts gaalasepaa ttataaataa sasasavaks

601 tgsgkvrkak akrsntlkgl raklnwrgrh kesandsadg qqmphssvas tpsgvy

M Proc R PC (Genbank Ref # AAX52477.1)

1 mtmsststat atstatatld eanatvgemf sdadmaevrh vvq rilvpcv fvigllgnsv

61 siyvltr krm rcttniylta laitdiaylt cqlilslqhy dypkyhfkly wqly gyfvwl

121 cdsfgyisiy iavcfti erf iairyplkrq tfcteslakk viaavaifcl lstlst afeh

181 titigtrqid dayqpcnqtv anispmpppp vavtpplatp plptpatiwq spdsamestt

241 sgssnqlvdw gsgsgdgepe niprhrrhwq ssgfvtlptl rktleeqdqk vadaaqrsgv

301 tesllqlwrr krsaenhnin ntdafafnvt eycqnvtyyn hglselgyde ly sylwnlft

361 llvfvvfpll llatfn sili llvhrsknlr gdltnassir rtkrksnsgl kgsvsqenrv

421 titliavvlm fivcqlpwai ylivnqymei qigtq vvagn vcnllaslha asnfflycvl

481 sdkyrktvre litgyryrrr harnntslyv ph***ttttlt***qi ngdhygsnyg gagsrrnrnt

541 grlia

V Proc R PC-like (Genbank Ref # XP_015026487.1)

1 mttlaatsts tampvdgigt akmfsdadva evrhvvqril vpcvfvigll gnsvsiyvlt

61 rkrmrcttni ylsalaitdi ayltfvlils lkhyeyikyh celywrlygf imwlcdacay

121 isiyiavcft ierfiairyp lkrqtfctes lakkviaava lfcllttlst afehtydinw

181 klidgayrpc nltlanvspt paqptlataw hvdehannaa tprylepfdl ssgsgsgage

241 sdhipsrqhr lpastsasvg vtaaatepat aaatasllql srarrsedny dseassnnny

301 nynnnsafaf niteycqnmt vytnglsslg qnalyfniws vytlivfvvl plfvlatfnc

361 flillvhrsk slrgdltnas sirrtkrk***sn sgit***gsvsqe nrvtitliav vllfivcqlp

421 waiylilvqy veiemniqri agnvcnllva inaaanffly cvlsdkyrkt vrelitgyry

481 rhrharnnis lyaphttttl ngdgagggga sgyg***ssysga ss***rrcraksa varrlia

M rkts PA (Genbank Ref # AAF53367)

1 maarcrwswr lalcplllql llqlllllpps amghdetken papdmqnsqe qepyvhlqhl

61 qqqqqqnpqt vqqlsqitvn rtsksasvtp tgirenvmlp sadpekeaqi lyekslqeyh

121 gsqlstasta tdviagkrtl hsicerwlqk hchctgslev lrlscrgigi lavpvnlpne

181 vvvldlgnnn ltkleansff mapnleeltl sdnsiinmdp nafyglaklk rlslqncglk

241 slppqsfqgl aqltslqlng nalvsldgdc lghlqklrtl rlegnlfyri ptnalaglrt

301 lealnlgsnl ltiindedfp rmpnlivlll krnqimkisa galknltalk vlelddnlis

361 slpeglskls qlqelsitsn rlrwindtel prsmqmldmr anplstispg afrgmsklrk

421 lilsdvrtlr sfpeleacha leilkldrag iqevpanlcr qtprlkslel ktnslkripn

481 lsscrdlrll dlssnqieki qgkpfnglkq lndlllsynr ikalpqdafq gipklqlldl

541 egneisyihk eafsgftale dlnlgnnifp elpesglral lhlktfnnpk lrefpppdtf

601 priqtlilsy ayhccaflpl vamssqkkts qvqeavlfps daefdmtlwn nsmmniwpqm

661 hnlskqlgas mhdpwetain fneeqlqtqt ggqiatsyme eyfeehdvsg patgygfgtg

721 lfsgmstedf qpgsvqclpm pgpflpcadl fdwwtlrcgv wvvfllsllg ngtvvfvllc

781 srskmdvprf lvcnlaaadf fmgiylgila ivdaatlgef rmfaipwqms vlcqlsgfla

841 vlsselsvyt lavitlerny aithaihlnk rlslkqagyi msvgwvfali malmplvgvs

901 dyrkfavclp fetttgpasl tyvislmfin gcafltlmgc ylkmywairg sqawntndsr

961 iakrmallvf tdflcwspia ffsitaifgl qlisleqaki ftvfvlplns ccnpflyaim

1021 tkqfkkdcvt lckhfeesrv vggggpggrg avartkrgdl pppllpaaav ahppgcrclr

1081 mlpsempnwh kmeqtpsmwq rlrtfccgen rrrrkqrrqp qqrrqrayta aaanpyqyqf

1141 aelrqqrqnr assissenfc ssrssswrhg ppssapvppg ncsmplkmle phahphghgr

1201 rrhsawlitr kt***sqdsnls***s srnd***ssasat*** ***tastst***frl***s*** ***rssags***stpl psiiahngka

1261 qldavkprlv rqeavqeeed sspprlgvrf lp***tipsaad***s svvmedgd***sa*** ***ntgvas***flgm

1321 plpgassgfl iapttaatts pppvvlqpak pppdpndapl

V Rk PA-like (Genbank Ref # XP_002057499.2)

1 maahsrrrrk qkrsdagrav trvaaatsit afglqlsmpp lllltlllmr tttalltpts

61 mhlnetkenr gtakqlsgis vnnmsstpta tptttptatp vgirenvmlp ssdpereaqi

121 lyekslqeyh gkaaaaaaaa aasassnvds ngvsgsgsgs atkttarslh svcelwllkh

181 chctgslenl klscrsigil avpvnlpsev lfldlgnnnl trleansffm vphleeltls

241 dnsiinmdpy afyglaklkr lslqncglka laphsfqgls qlvslqlngn alvsldgncl

301 gnlqqlrtlr legnlfyrip tnalaglktl ealnlgsnll tiindedfpr mpnlivlllk

361 rnqimkisag alknltalkv lelddnliss lpeglgklpq lqelsmtsnr lrwindtelp

421 rsmqildlra nplstittga frgmsklrkl ilsdvrtlrn fpeleachal eilkldragi

481 qevpsnlcrq tprlkslelk tnslksipnl sscrdlrlld lssnqietlq grpfhglkql

541 hdlllsynri ktlpqdafqg ipklqlldle gneivhihkd afaaftaled lnlgnnifph

601 lpeaglrall hlktfnnpkl refpppdtfp riqtlilsya yhccaflplv amsaqrktsq

661 vqeavlfpsd aefdmtlwnn smmniwpqmh nlskqlgaam hdlwdsplnn ynlnedlqqs

721 ptgsqsassy meeyfdehdv sgpgtgygfg tglfsgitad dlqpgsvqcl pmpgpflpca

781 dlfdwwtlrc gvwvvfllal lgngtvvfvl vcsrskmdvp rflvcnlaaa dffmgiylgi

841 laivdaatlg efrmfaipwq msllcqlagf lavlsselsv ytlavitler nyaithaihl
901 nkrlslrqag yimsvgwifa lcmallpllg vsdyrkfavc lpfetttgva sltyvislmf
961 ingcafltlm gcylkmywai rgsqawntnd sriakrmall vftdflcwsp iaffsitaif
1021 glqlisleqa kiftvfvlpl nsccnpflya imtkqfkkdc vtlckhfeet rvvgggataa
1081 araargkrgg elppplpa***t*** ***ataat***aagva hppgcrclrm lpsempnwhk meqttptlwq
1141 rlkrlccgqr qrrkqrrqpq qrrqraytaa aanpyqyqfa elrqqrknra ssissenfcs
1201 srssswrhga nsstpapqsn csmplklmep gqshthgrrr hsawlitrkt sqdsnlsssr
1261 nd***ssasatta*** ***stst***frl***srs*** ***sags***stplps iiahngkqqh etltkprlvr qeavqeeeds
1321 spprlgvrfl p***tipsaad***ss vimddgdsan aaagsagclg mpqpgvssgf lvtppaqlqp
1381 ekpppapnda pg

M RYa R PA (Genbank Ref # AAF56655.3)

1 mehhnshllp ggsekmyyia hqqpmlrned dnyqegyfir pdpasliynt talpaddegs
61 nygygstttl sglqfetyni tvmmnfscdd ydllsedmws sayfkiivym lyipififal
121 igngtvcyiv ystprmrtvt nyfiaslaig dilmsffcvp ssfislfiln ywpfglalch
181 fvnysqavsv lvsaytlvai sidryiaimw plkpritkry atfiiagvwf ialatalpip
241 ivsgldipms pwhtkcekyi cremwpsrtq eyyytlslfa lqfvvplgvl iftyaritir
301 vwakrppgea etnrdqrmar skrkmvkmml tvvivftccw lpfnilqlll ndeefahwdp
361 lpyvwfafhw lamshccynp iiycymnarf rsgfvqlmhr mpglrrwccl rsvgdrmnat
421 sgtgpalpln rmn***tsttyis*** arrkpratsl ranplscget splr

V RYa R PA (Genbank Ref # XP_002053912.2)

1 mefnsfvwqr rrldlqrtvl nderlyyiap qqpllrnedd yqdtagalmy nsssstelslg
61 aedadyisst patehfnvtv llnfsctdvs shsddlwssd yfksvvylly ipififallg
121 ngivcyivqs tprmrtvtny fianlalgdi lmslfcvpss fisqyilnyw pfgivlchfv
181 nysqvvsvlv saytlvaisi dryiaimwpl rpritkryak fiiagvwfia latafpipvv
241 srlmvpssiw hekcekyicr evwpsteqdy yytlalftlq fivpllvlif tytriaiavw
301 gkrppgeaen srdqrmarsk rkmikmmltv vivftscwlp fnilqlmlnd eefanwkplp
361 yvwfafhwla mshscynpii ycymnarfrg gflqimyrvp glrrccclhr ylrsrgersy
421 eatg//tedafh lhrvn***tctty is***trrklrtn smqm//***sqfsca et***tvlr

M RYa R PB (Genbank Ref # AAF56655.3) (difference from PA underlined)

1 mehhnshllp ggsekmyyia hqqpmlrned dnyqegyfir pdpasliynt talpaddegs
61 nygygstttl sglqfetyni tvmmnfscdd ydllsedmws sayfkiivym lyipififal
121 igngtvcyiv ystprmrtvt nyfiaslaig dilmsffcvp ssfislfiln ywpfglalch
181 fvnysqavsv lvsaytlvai sidryiaimw plkpritkry atfiiagvwf ialatalpip
241 ivsgldipms pwhtkcekyi cremwpsrtq eyyytlslfa lqfvvplgvl iftyaritir
301 vwakrppgea etnrdqrmar skrkmvkmml tvvivftccw lpfnilqlll ndeefahwdp
361 lpyvwfafhw lamshccynp iiycymnarf rsgfvqlmhr mpglrrwccl rsvgdrmnat
421 sgemttkyhr hvgdalfrkp kicir/ng***sst ssqs***nehihh lhqrsskats difasepiiv
481 rrdvttavav isknktdspv rr***sgssggt***e anirstef

V Rya R PB-like (not annotated; predicted based on inspection of genomic DNA documented in SXX Document – "de novo RYaR annotations")

1 mefnsfvwqr rrldlqrtvl nderlyyiap qqpllrnedd yqdtagalmy nsssstelslg
61 aedadyisst patehfnvtv llnfsctdvs shsddlwssd yfksvvylly ipififallg
121 ngivcyivqs tprmrtvtny fianlalgdi lmslfcvpss fisqyilnyw pfgivlchfv
181 nysqvvsvlv saytlvaisi dryiaimwpl rpritkryak fiiagvwfia latafpipvv
241 srlmvpssiw hekcekyicr evwpsteqdy yytlalftlq fivpllvlif tytriaiavw
301 gkrppgeaen srdqrmarsk rkmikmmltv vivftscwlp fnilqlmlnd eefanwkplp
361 yvwfafhwla mshscynpii ycymnarfrg gflqimyrvp glrrccclhr ylrsrgersy

421 eatg//emtkynrrngdglvrkpkirir/nrrcipptscehlhhlhqhktkaahefyane/pifm crdhsvavapgtaqepkprlqprqptgsfhln***sasyls***stqf

M RYa R PC (Genbank Ref # AHN57552)

1 mehhnshllp ggsekmyyia hqqpmlrned dnyqegyfir pdpasliynt talpaddegs
61 nygygstttl sglqfetyni tvmmnfscdd ydllsedmws sayfkiivym lyipififal
121 igngtvcyiv ystprmrtvt nyfiaslaig dilmsffcvp ssfislfiln ywpfglalch
181 fvnysqavsv lvsaytlvai sidryiaimw plkpritkry atfiiagvwf ialatalpip
241 ivsgldipms pwhtkcekyi cremwpsrtq eyyytlslfa lqfvvplgvl iftyaritir
301 vwakrppgea etnrdqrmar skrkmvkmml tvvivftccw lpfnilqlll ndeefahwdp
361 lpyvwfafhw lamshccynp iiycymnarf rsgfvqlmhr mpglrrwccl rsvgdrmnat
421 sgemttkyhr hvgdalfrkp kicircktlh lvsvsvflfv llrfwfi

M SIF R PA (Genbank Ref # AAN13859.2)

1 mmaasgrirk rkhkshtsgd vpstttsvpm piptmapgkm vaetmeeaaa lagdynnfth
61 nfvdlqnlls fnelngtsgs ggtavsslgs ssaiklnnsa itdtllgtvl ttatatvapa
121 assllatlaa tttasargsl agkslaiada tsstyysnll nlspattsli saaaatksyn
181 dsalrweqld gsvdfgfdpl yrhslamsmv ycvayivvfl vglignsfvi avvlraprmr
241 tvtnyfivnl aiadilvivf clpatligni fvpwmlgwlm ckfvpyiqgv svaasvysli
301 avsldrfiai wwplkqmtkr rarimiigiw vialvttipw llffdlvpae evfsdalvsa
361 ysqpqflcqe vwppgtdgnl yfllanlvac yllpmslitl cyvliwikvs trsipgeskd
421 aqmdrmqqks kvkvikmlva vvilfvlswl plyvifarik fgsdisqeef eilkkvmpva
481 qwlgssnsci npilysvnkk yrrgfaaiik srsccgrlry ydnvaia***sst tstrks***shyh
541 qnssrkspss kgnavsyiye hnslrrhnmm lkqdsnlsqq mllkqdshgs rqflikqe***ss***
601 ***csdas***girrp lcqqd***sngsk vslskqdsiv syme***arrsag hglndtlvdr dsvsmdvgrr
661 qga***tpsslld*** krqkfvkqd***s visfvd***qrpe qrrhqlvkqd ***svisfad***qrr gllhkqdslm
721 anrtgdapth hvsilkk***tds qlsygssts***p rrnadlye

V SIFa R PA-like (Genbank Ref # XP_002058467.3)

1 mavggrtrkr khrshaagdt ptttaaaaaa agtaatataa itnssssssgn tpatgglgrg
61 wpmleqrdll edvtqgagea nnfthnfvdl qnllnfndaa ssnnsnnssn nvvgvsfsps
121 aiklsngait dtllgtiltt atatvapaas slissltaat atattttaat atssssssqla
181 aigmpgaviv adatsssyya sllnmspatt slitaaaatk syndsllrwd qldgnvdfgf
241 dplyrhslam sivycvayiv vflvgligns fviavvlrap rmrtvtnyfi vnlaiadilv
301 ivfclpatli gnlfvpwmlg wlmckfvpyi qgvsvaasvy sliavsldrf iaiwwplkqm
361 tkrrarimii giwvialvtt ipwllffdlv paeevfsdal vssytqpqyl cqevwppgtd
421 gnlyfllanl vacyllpmsl itlcyvliwi kvstrsipge lskdaqmdrm qqkskvkvik
481 mlvavvilfv lswlplyvif arikfgsdis qeefeilkkv mplaqwlgss nscinpilys
541 vnkkyrrgfa aiiksrsccg rlryydnvai a***ssttstrks*** shyhpngsrk spsspglrkt
601 navsyiyehn slrrhnlmmk qdsnlsqqml lkqdshgsrq flikqe***sscs das***gtrrllc
661 qqd***sngskvs lskqdsivsy me***srrvaala aqer***svdsal t***qqd***tisies*** rrggaqa***tpa***
721 ***slld***krqkfv kqd***svisfvd*** qrpeprrhql vkqd***svisfa d***qrrgllhkq d***slmtnrs***gd
781 apthhvsilk ktdsqlsyg***t sssssas***pr rnvelye

M sNPF R PA (Genbank Ref # AAF49074)

1 manlswlsti tttsssists qlplvsttnw sltspgttsa iladvaasde drsggiihnq

61 fvqiffyvly atvfvlgvfg nvlvcyvvlr nramqtvtni fitnlalsdi llcvlavpft

121 plytfmgrwa fgrslchlvs faqgcsiyis tltltsiaid ryfviiypfh prmklstcig

181 iivsiwvial latvpygmym kmtnelvngt qtgnetlvea tlmlngsfva qgsgfieapd

241 stsatqaymq vmtagstgpe mpyvrvycee nwpseqyrkv fgaitttlqf vlpffiisic

301 yvwisvklnq rarakpg***sks srre***eadrdr kkrtnrmlia mvavfglswl pinvvnifdd

361 fddksnewrf yilfffvahs iamsstcynp flyawlnenf r//kefkhvlpc fnpsnnniin

421 itrgynrsdr ntcgprlhhg kgdggmgggs ldaddqdeng itqetclpke klliiprept

481 ygngtgavsp ilsgrginaa lvhggdhqmh qlqpshhqqv eltrrirrrt detdgdylds

541 gdeqtvevrf setpfvstdn t***tgisile***ts tshcqdsdvm velgeaigag ggaelgrrin

V sNPF R PA-like (Genbank Ref #XP_002048039.1)

1 manvsnetvn awlvsvstql phvlgidvgv nwsssst tst ttmaipsssm gttttastvs

61 slssdantda aadadksgii hnqfvqiffy vlyttvfvlg vfgnvlvcyv vlrnramqtv

121 tnifitnlal sdillcvlav pftplytfmg rwafgrtlch lvsfaqgcsi yistltltsi

181 aidryfviiy pfhprmklst cigiivsiwv iallatvpyg mymkmtnevm dnttqlvgan

241 qtsssrygny glatpdatsa aqaymqvmtd gvtvceenwp sehyrkvfga itttlqfvlp

301 ffiisicyvw isvklnqrar akpg***skssrr e***eadrdrkkr tnrmliamva vfglswlpin

361 lvnifddfdd ksnewrlyml fffvahsiam sstcynpfly awlnenfrke fkhvlpcfnp

421 snnniinitr gynrsdrntc gprlhhgnge ggaggsldad dqddngitqe tclpkeklli

481 ipreptygng ngavspilsg rginaallha sqpqqqqqqv qqqqveltrr irrrtdddtd

541 fidsgdeqtv evrfsetpfv ssdnt***tgism le***tsesqfqd sgelpelsav vvgdasrrcn

M SP R PA (Genbank Ref # AAF46037**)**

```
  1 mdnytdvlyq yrlapsaspe memeladprq mvrgfhlptn esqleipdyg nesldypnyq
 61 qmvggpcrme dnnisywnlt cdspleyamp lygycmpfll iitiisnsli vlvlskksma
121 tptnfvlmgm aicdmltvif papglwymyt fgnhykplhp vsmclaysif neimpamcht
181 isvwltlala vqryiyvcha pmartwctmp rvrrctayia llaflhqlpr ffdrtymplv
241 iewngsptev chletsmwvh dyigvdlyyt
271 syylfrvlfv hllpciilvt lnillfaamr qaqerrkllf renrkkeckk lretncttlm
331 livvvsvfll aeipiavvta mhivssliie fldyglanic imltnfflvf sypinfgiyc
391 gmsrqfretf keiflgrlma kkdsstkysi vngartctnt netvl
```

V SP R PA-01like (Genbank Ref #XP_002055695.1**)**

```
  1 marsvdqsll ieleevstlt aaatavakss smdnntvysy disitdvlyq wsaaasgara
 61 iaqsaasgpl ptasvaavae rlpelvgsml etrasqasln esqfllvdya nsmndsldya
121 syqqqlgsse crqmdgnmsy wnltcdspld yalplygycm pfllfmtiis nslivlvlsk
181 ksmatptnfv lmgmaicdll tvvfpapglw ymytfgnhyk pmhpvsmcla ysifneimpa
241 mchtisvwlt lalavqryiy vchapmartw ctmprvrrct fyiallaflh qlprffdrty
301 mpmeiewngn etevchlets lwvheyvgvd lyyt
335 syylfrvlfv nllpciilvt lnillfaalr qaqerrkllf renrkkeckr lrdsncttlm
395 livvvsvfli aeipiavvta mhivssliie fldygianif imltnfflvl sypinfgiyc
391 gmsrqfretf reifmgrvag kkessskysi vngprtctnt netil
```

M SPR PD (Genbank Ref # AAF46037**) (stop suppression)**

```
  1 mdnytdvlyq yrlapsaspe memeladprq mvrgfhlptn esqleipdyg nesldypnyq
 61 qmvggpcrme dnnisywnlt cdspleyamp lygycmpfll iitiisnsli vlvlskksma
121 tptnfvlmgm aicdmltvif papglwymyt fgnhykplhp vsmclaysif neimpamcht
181 isvwltlala vqryiyvcha pmartwctmp rvrrctayia llaflhqlpr ffdrtymplv
241 iewngsptev chletsmwvh dyigvdlyyt
271 syylfrvlfv hllpciilvt lnillfaamr qaqerrkllf renrkkeckk lretncttlm
331 livvvsvfll aeipiavvta mhivssliie fldyglanic imltnfflvf sypinfgiyc
391 gmsrqfretf keiflgrlma kkdsstkysi vngartctnt netvlxxlvm lvprrgssdh
451 rrsststttt tttktiggsm iiggeasaqh qhlvthhlqt hsqpsqqrrv stmdiiteer
511 il
```

M Tk R 86C PA (Genbank Ref # AAF46037**)**

```
  1 mseivdtell vnctilavrr felnsivntt llgslnrtev vsllssiidn rdnlesinea
 61 kdflteclfp sptrpyelpw eqktiwaiif glmmfvaiag ngivlwivtg hrsmrtvtny
121 fllnlsiadl lmsslncvfn fifmlnsdwp fgsiyctinn fvanvtvsts vftlvaisfd
181 ryiaivhplk rrtsrrkvri ilvliwalsc vlsapcllys simtkhyyng ksrtvcfmmw
241 pdgryptsma dyaynliilv ltygipmivm licyslmgrv lwgsrsigen tdrqmesmks
301 krkvvrmfia ivsifaicwl pyhlffiyay hnnqvastky vqhmylgfyw lamsnamvnp
361 liyywmnkrf rmyfqriicc ccvgltrhrf dspksrltnk nssnrhtrae tksqwkrstm
421 etqiqqapvt sscreqrsaq qqqppgsgtn raavecimer padgsssplc lsinnsiger
481 qrvkikyisc dednnpvels pkqm
```

V Tk 86C R PA- (Genbank Ref #XP_002053610.1**)**

```
(virilis lost the intron/exon encoding the additional CT sequence
  1 mseivdtell vnctilavrr felntivntt llntlnrtev vgllsgiien rdnldsinea
 61 kdflteclfp sptrpyelpw eqktiwaivf glmmfvaiag ngivlwivtg hrsmrtvtny
121 fllnlsiadl lmstlncvfn fifmvnsdwp fgsiyctinn fvanvtvsts vftlvaisfd
181 ryiaivhplk rrtsrrkvrf ilvliwalsc vlsapcllys simtkhyyng ksrtvcfmmw
241 pdgryptsmt dyvynvtilv ltygipmivm licyslmgrv lwgsrsigen tdrqmesmks
301 krkvvrmfia ivsifaicwl pyhlffiyay hnnqvastky vqhmylgfyw lamsnamvnp
361 iiyywmnkr/f rmyfqriifc cclglmryrf espksrmank nssnrhtrae tksqwkrstm
421 etqiqqmpkt ssrdkdagvq glnhtaveci ierpiddnss piclsiknsa gerqrvkiky
481 iscdednnpi eegsennssh dsnhshghgh scgrshgqna kaiqql
```

(155) virilis adds 25 bp at end (new exon or read through?) which includes a BBS

M Tk R 86C PB (Genbank Ref # ABW08638)

(underline is the difference with D melanogaster PA, due to an unspliced intron)

```
  1 mseivdtell vnctilavrr felnsivntt llgslnrtev vsllssiidn rdnlesinea
 61 kdflteclfp sptrpyelpw eqktiwaiif glmmfvaiag ngivlwivtg hrsmrtvtny
121 fllnlsiadl lmsslncvfn fifmlnsdwp fgsiyctinn fvanvtvsts vftlvaisfd
181 ryiaivhplk rrtsrrkvri ilvliwalsc vlsapcllys simtkhyyng ksrtvcfmmw
241 pdgryptsma dyaynliilv ltygipmivm licyslmgrv lwgsrsigen tdrqmesmks
301 krkvvrmfia ivsifaicwl pyhlffiyay hnnqvastky vqhmylgfyw lamsnamvnp
361 liyywmnkrf rmyfqriicc ccvgltrhrf dspksrltnk nssnrhtrgg ytvahslpns
421 sppttqtlla vlaqtltqpk pqtqlllshh sphptqpsaa etksqwkrst metqiqqapv
481 tsscreqrsa qqqqppgsgt nraavecime rpadgssspl clsinnsige rqrvkikyis
541 cdednnpvel spkqm
```

V Tk R 86C PB

```
  1 mseivdtell vnctilavrr felntivntt llntlnrtev vgllsgiien rdnldsinea
 61 kdflteclfp sptrpyelpw eqktiwaivf glmmfvaiag ngivlwivtg hrsmrtvtny
121 fllnlsiadl lmstlncvfn fifmvnsdwp fgsiyctinn fvanvtvsts vftlvaisfd
181 ryiaivhplk rrtsrrkvrf ilvliwalsc vlsapcllys simtkhyyng ksrtvcfmmw
241 pdgryptsmt dyvynvtilv ltygipmivm licyslmgrv lwgsrsigen tdrqmesmks
301 krkvvrmfia ivsifaicwl pyhlffiyay hnnqvastky vqhmylgfyw lamsnamvnp
361 iiyywmnk/at ecrrfntlv vmghgnsdii isstessfmvg ysgs/icclg lmryrfespk
421 srmanknssnr htraetksq wkrstmetqi qqmpktssrd kdagvqglnh taveciierp
481 iddnsspicls iknsagerq rvkikyiscd ednnpieegs ennsshdsnh shghghscgr
541 shgqna kaiqql
```

ATECRRFNTLVVMGHGNSDIII

SSTESSFMVGYSGSI

M Tk R 99D PA (Genbank Ref # ABW08638)

1 menrsdfead dygdiswsnw snwstpagvl fsamssvlsa snhtplpdfg qelalstssf
61 nhsqtlstdl pavgdvedaa edaaasmetg sfafvvpwwr qvlwsilfgg mvivatggnl
121 ivvwivmttk rmrtvtnyfi vnlsiadamv sslnvtfnyy ymldsdwpfg efycklsqfi
181 amlsicasvf tlmaisidry vaiirplqpr mskrcnlaia aviwlastli scpmmiiyrt
241 eevpvrglsn rtvcypewpd gp***tnhstme***s lyniliiilt yflpivsmtv tysrvgielw
301 gsktigectp rqvenvrskr rvvkmmivvv lifaicwlpf hsyfiitscy paiteapfiq
361 elylaiywla msnsmynpii ycwmnsrfry gfkmvfrwcl fvrvgtepfs rrenltsrys
421 csgspdhnri krndtqksil ytcpsspksh rishsgtgrs atlrnslpae **slssggs**ggg
481 ghrkrlsyqq emqqrwsgpn ***satavtnsss tant***tqlls

V Tk R 99D PA-like (Genbank Ref # XP_032294790)

1 mldsittttt gagqvmenne elldntwsnw styapvliyn amnsvlanqt plsseygpsl
61 aynlsrvlpt gitglpaadv dedaatpvas fafvvpwwrq vlwsilfggm vivatggnli
121 vvwivlttkr mrtvtnyfiv nlsiadamvs slnvtfnyyy mldsdwifge fyckvsqfia
181 mlsicasvft lmaisidryv aimkplqprm skrrnlaiaa liwlsstlis cpmllffrte
241 evpvtmenkt rivcfpewpd gq***tnhskqe***h iynilililt yflpiismtv tysrvgielw
301 gsktigeytp rqtenvrskr rvvkmmivvv lifgfcwlpf htyfivtscy paiteapfiq
361 elylviywla msnsmynpii ycwmnsrfry gfkmvfrwcp fvnvgaesln rrenltsrys
421 csgspdhnri krndtqksil yacpsspksq rvshcgkdd***s lsgkd***lslsh afsysysvag
481 vpprslpvqq lsssrrgqrs syqqemqerw sgdk***ssnsln s***ttecrttql ls

tnhstme – present in ECL

M Tk R 99D PC (Genbank ref #ABW08638)

1 menrsdfead dygdiswsnw snwstpagvl fsamssvlsa snhtplpdfg qelalstssf
61 nhsqtlstdl pavgdvedaa edaaasmetg sfafvvpwwr qvlwsilfgg mvivatggnl
121 ivvwivmttk rmrtvtnyfi vnlsiadamv sslnvtfnyy ymldsdwpfg efycklsqfi
181 amlsicasvf tlmaisidry vaiirplqpr mskrcnlaia aviwlastli scpmmiiyrt
241 eevpvrglsn rtvcypewpd gp***tnhstme***s lyniliiilt yflpivsmtv tysrvgielw
301 gsktigectp rqvenvrskr rvvkmmivvv lifaicwlpf hsyfiitscy paiteapfiq
361 elylaiywla msnsmynpii ycwmnsrfry gfkmvfrwcl fvrvgtepfs rrenltsrys
421 csgspdhnri krndtqksil ytcpsspksh rishsgtgrs atlrnslpae **slssggs**ggg
481 ghrkrlsyqq emqqrwsgpn ***satavtnsss tant***tqlls**x** qpaiqvvppe mqtqivcssp
541 ynnnyrranp glsdrdssdd ktwl

M Tre1 PA (Genbank Ref # NP_524792.1)

```
  1 mdqdmgmatg yfqdadmqmd epaaatqsiy phsatlfaai sacvfvtigv lgnlitllal
 61 lksptireha ttafvislsi sdllfcsfsl pltavrffqe swtfgttlck ifpvifygnv
121 avsllsmvgi tlnryiliac hsrysqiykp kfitlqllfv wavsfllllp pilgiwgemg
181 ldeatfscti lkkegrsikk tlfvigfllp clviivsysc iyitvlhqkk kirnhdnfqi
241 aaakgssssg ggsymtttct rkarednrlt vmmvtiflcf lvcflplmla nvvddernts
301 ypwlhiiasv mawassvinp iiyaasnrny rvayykifal lkfwgeplsp mpsrnyhqsk
361 nskelsgvir stplfhavqk nsinqmcqty sv
```

V Tre1 PA-like (Genbank Ref # XP_002056799.1)

```
  1 mdaptqsiyp hsatlfaaic acvfvtigvf gnlitllall ksptireha t tafvislsis
 61 dlffcsfslp ltavrffqes wtfgstlcki fpvifygnva vsllsmvgit lnryiliach
121 srysqiykpk litlqlvfvw avsfllllpp ilgiwgemgl deatfsctil kkegksikkt
181 lfligfllpc lviiisysci yitvlhqkkk irshdnfqig aaagaktgsa sgsyvtttst
241 rkarednrlt vmmvtiflcf licflplmla nvvdderktn ypwlhiiasv mawassvinp
301 iiyaasnrny rvayykifal lkfwgeplsp mpsrnyhqsk nskelsgvir stplfhavqk
361 nsinqmcqty sv
```

both present in ICL1 and 3

adjusted TM3 prediction to end just before SRYSQIYKP

M Trissin R PB (Genbank Ref # AAF52294)

1 mimtmmqtvr awqqesdveh rkqhkqrwrp dgahisaayd lnsdnddghh rvvhnqnngs

61 pnsspnqsts afrqrqphhp ptgqqpprlp ctvthfsahw ktllilltll sastltasan

121 vtstisppin gsstdyilly gesttslvpa lttglsgdgs gaviedeeda ekaseyifdr

181 tdvriifitl ytlvfcccff gnllvilvvt lsrrlrsitn fflanlafad fcvglfcvmq

241 nlsiyliesw vfgeflcrmy qfvhslsyta sifilvvicm eryfaivhpi tckqiltaar

301 lrmvivtvwi tsavystpkf vfsktiknih tqdgqeeeic vldremfnsk lldminfvll

361 yvmpllvmtv lyskiaialw rssrgltphv vqhqhqqpqq pscqdigmgm hnsmyhhhph

421 hhhhhhqhhq lqsaassagv vgvglggggg ggpgp***slasg*** ***gssttsls***rk qsskyekrgv

481 si***tesqld***nc kvsleadrpi vsacrktsfy hhghahhqra gnasvgggsg gagaga***thms***

541 ***hss***snvlrar rgvvrmliif vltfalcnlp yharkmwqyw srsyrgdsnf nalltpltfl

601 vtyfnsgvnp llyaflsrnf rkgmkelllc swkkgkgk***ss*** ***snss***mhhkrk alqth***slptd***

661 ***tt***higneql

V Trissin R PB* (not annotated; predicted based on inspection of genomic DNA documented in SXX Document – "de novo TrissinR annotations")

1 msvhslqgms tstaalptsc wqrqrqrlrr rrqqeteaas hrrahkqmrr tgsanisaay

61 ednddnnnnn nnnynnnnnn nnnskasksp ttttnysisg lpdcqqqkte qsthhkltts

121 ihpralpwlf lsplllllvll vdrslstagn ysslalnesg iestlpinat tailataapt

181 ggsgaattal alsepttteq adeedaenss eyvfdrtdvr iifitlytiv fcccffgnll

241 vilvvtlsrr lrsitnffla nlafadfcvg lfcvmqnlsi ylidswvfge flcrmyqfvh

301 slsytasifi lvvicmeryf aivhpitckq iltaarlrmv ivtvwitsav ystpkfvfsk

361 tiknihtedg qeeeicvldr emfnsklldm infvllyvmp llvmtvlysk iaialwrssr

421 glsphvtqhq qqqqqhqqq qqqaiqensm slhnsmyhhh hhhpqhphhh qlhqqhqlpa

481 saaaaaaas***sl*** ***asgssstsls*** rkhsskyekr gvsi***tesqld*** **nckvsleadr** pivsacrkts

541 fyhhshshnq rqgnggaggg ga***thmshss***s nvlrarrgvv rmliifvltf alcnlpyhar

601 kmwqywsrsy rgdsnfnall tpltflvtyf nsgvnpllya flsrnfrkgm kelllcswkk

661 gkgk***sssnss*** mhhkrkalqt h***slptdtt***hi gneql

M Trissin PC (Genbank Ref # AAF52294)

1 mimtmmqtvr awqqesdveh rkqhkqrwrp dgahisaayd lnsdnddghh rvvhnqnngs

61 pnsspnqsts afrqrqphhp ptgqqpprlp ctvthfsahw ktllilltll sastltasan

121 vtstisppin gsstdyilly gesttslvpa lttglsgdgs gaviedeeda ekaseyifdr

181 tdvriifitl ytlvfcccff gnllvilvvt lsrrlrsitn fflanlafad fcvglfcvmq

241 nlsiyliesw vfgeflcrmy qfvhslsyta sifilvvicm eryfaivhpi tckqiltaar

301 lrmvivtvwi tsavystpkf vfsktiknih tqdgqeeeic vldremfnsk lldminfvll

361 yvmpllvmtv lyskiaialw rssrgltphv vqhqhqqpqq pscqdigmgm hnsmyhhhph

421 hhhhhhqhhq lqsaassagv vgvglggggg ggpgp***slasg*** ***gssttsls***rk qsskyekrgv

481 si***tesqvs***le adrpivsacr ktsfyhhgha hhqragnasv gggsggagag a***thmshss***sn

541 vlrarrgvvr mliifvltfa lcnlpyhark mwqywsrsyr gdsnfnallt pltflvtyfn
601 sgvnpllyaf lsrnfrkgmk elllcswkkg kgk***sssnss***m hhkrkalqth ***slptdtt***hig
661 neql

PC form does not include the micro exon(at 486) present in PB which inserts ~6 AA; in both there is a precise BBS, but sequence varies (TESQLD vs. TESQVS)

V Trissin R PC-like (Genbank Ref # XP_002051478.2)

1 msvhslqgms tstaalptsc wqrqrqrlrr rrqqeteaas hrrahkqmrr tgsanisaay
61 ednddnnnnn nnnynnnnnn nnnskasksp ttttnysisg lpdcqqqkte qsthhkltts
121 ihpralpwlf lsplllllvll vdrslstagn ysslalnesg iestlpinat tailataapt
181 ggsgaattal alsepttteq adeedaenss eyvfdrtdvr iifitlytiv fcccffgnll
241 vilvvtlsrr lrsitnffla nlafadfcvg lfcvmqnlsi ylidswvfge flcrmyqfvh
301 slsytasifi lvvicmeryf aivhpitckq iltaarlrmv ivtvwitsav ystpkfvfsk
361 tiknihtedg qeeeicvldr emfnsklldm infvllyvmp llvmtvlysk iaialwrssr
421 glsphvtqhq qqqqqhqqq qqqaiqensm slhnsmyhhh hhhpqhphhh qlhqqhqlpa
481 saaaaaas***sl asgssstsls*** rkhsskyekr gvsi***tesqvs*** leadrpivsa crktsfyhhs
541 hshnqrqgng gaggga***thm shss***snvlra rrgvvrmlii fvltfalcnl pyharkmwqy
601 wsrsyrgdsn fnalltpltf lvtyfnsgvn pllyaflsrn frkgmkelll cswkkgkgk***s***
661 ***ssnss***mhhkr kalqth***slpt dtt***higneql

M Trissin R PD (Genbank Ref # AGB92644**)**

1 mimtmmqtvr awqqesdveh rkqhkqrwrp dgahisaayd lnsdnddghh rvvhnqnngs
61 pnsspnqsts afrqrqphhp ptgqqpprlp ctvthfsahw ktllilltll sastltasan
121 vtstisppin gsstdyilly gesttslvpa lttglsgdgs gaviedeeda ekaseyifdr
181 tdvriifitl ytlvfcccff gnllvilvvt lsrrlrsitn fflanlafad fcvglfcvmq
241 nlsiyliesw vfgeflcrmy qfvhslsyta sifilvvicm eryfaivhpi tckqiltaar
301 lrmvivtvwi tsavystpkf vfsktiknih tqdgqeeeic vldremfnsk lldminfvll
361 yvmpllvmtv lyskiaialw rssrgltphv vqhqhqqpqq pscqdigmgm hnsmyhhhph
421 hhhhhhqhhq lqsaassagv vgvglggggg ggpgp***slasg gssttsls***rk qsskyekrgv
481 si***tesqld***nc kvsleadrpi vsacrktsfy hhghahhqra gnasvgggsg gagaga***thms***
541 ***hss***snvlrar rgvvrmliif vltfalcnlp yharkmwqyw srsyrgdsnf nalltpltfl
601 vtyfnsgvnp llyaflsrnf rkgmkelllc swkkgkgk***ss snss***mhhkrk alqsafftp

V Trissin PD* (not annotated; predicted based on inspection of genomic DNA documented in SXX Document – "de novo TrissinR annotations")

1 msvhslqgms tstaalptsc wqrqrqrlrr rrqqeteaas hrrahkqmrr tgsanisaay
61 ednddnnnnn nnnynnnnnn nnnskasksp ttttnysisg lpdcqqqkte qsthhkltts
121 ihpralpwlf lsplllllvll vdrslstagn ysslalnesg iestlpinat tailataapt

181 ggsgaattal alsepttteq adeedaenss eyvfdrtdvr iifitlytiv fcccffgnll

241 vilvvtlsrr lrsitnffla nlafadfcvg lfcvmqnlsi ylidswvfge flcrmyqfvh

301 slsytasifi lvvicmeryf aivhpitckq iltaarlrmv ivtvwitsav ystpkfvfsk

361 tiknihtedg qeeeicvldr emfnsklldm infvllyvmp llvmtvlysk iaialwrssr

421 glsphvtqhq qqqqqhqqq qqqaiqensm slhnsmyhhh hhhpqhphhh qlhqqhqlpa

481 saaaaaas***sl asgssstsls*** rkhsskyekr gvsi***tesqld*** nckvsleadr pivsacrkts

541 fyhhshshnq rqgnggaggg ga***thmshss***s nvlrarrgvv rmliifvltf alcnlpyhar

601 tskmwqywsr syrgdsnfna lltpltflvt yfnsgvnpll yaflsrnfrk gmkelllcsw

661 kkgkgk***sssn ss***mhhkrkal qvsantkyyr vtlsiwh

M Dh31 R PA (Genbank Ref #XP_032292141.1)

```
  1 msdqignpna tfsgsgsgsg tnvasiaesv aesgpdfdal raacetrlna sgqlagsggp
 61 gaeagthcag tfdgwlcwpd tavgtsayel cpdfitgfdp aryahkecgl dgewfkhplt
121 nktwsnyttc vnledlnwrh tvnlisevgy gtsllaills lailgyfksl kcaritlhmn
181 lfasfaanns lwlvwyllvm pnsellhqsp mrcvalhitl hyfllsnysw mlcegfylht
241 vlvaafisek rlvkwliafg wgspaivifv ysmarglggt pednrhcwmn qtnyqnilmv
301 pvcismflnl lflcnivrvv llklnapasi qgscgpsrtv lqafratlll vpllglqyil
361 tpfrpapkhp wentyeiisa ftasfqglcv ailfcfcnge viaqmkrkwr mmcfsnrprt
421 nsytatqvsf vrcgpplpge ekv
```

(in 421: nsy***tatqvs***f)

V Dh31 R PA-like (Genbank Ref # AAN16138.1)

```
  1 maeqtsnsss sssgsvssss sssgsshiaa esgpdfdalr aacnarlnss hqltgkrrcd
 61 mphaachstv qfayklvslg sycagtfdgw lcwpdtaags sayelcpdfi tgfdparyah
121 kecgedgewf khpltnktws nyttcvnldd larnhnvnli yevgysisll aillslails
181 yfkslkcari tlhmnlfssf aansslwliw ylvvvpntel vqlspgycva lhiilhyfll
241 tnyswmlceg fylhtvlvaa fisekklvkw liafgwcspa iviciyglar gftgsweqnl
301 hcwmtdtdfn yilivpvcis iflnllflcn ivrvvllkln apasiqgscg psrtvlqafr
361 atlllvpllg lqymltpfrp gqehpleyty qvisaftasf qglcvatlfc ffngeviaqv
421 krkwrtvcfs nrprtnsyta tqvsfvrcgp pvpgeekv
```

(in 421: nrprtnsy***ta tqvs***fvrcgp)

M Dh31 R PC (Genbank Ref # AGB93483)

1 msdqignpna tfsgsgsgsg tnvasiaesv aesgpdfdal raacetrlna sgqlagsggp
61 gaeagthcag tfdgwlcwpd tavgtsayel cpdfitgfdp aryahkecgl dgewfkhplt
121 nktwsnyttc vnledlnwrh tvnlisevgy gtsllaills lailgyfksl kcaritlhmn
181 lfasfaanns lwlvwyllvm pnsellhqsp mrcvalhitl hyfllsnysw mlcegfylht
241 vlvaafisek rlvkwliafg wgspaivifv ysmarglggt pednrhcwmn qtnyqnilmv
301 pvcismflnl lflcnivrvv llklnapasi qgscgpsrtv lqafratlll vpllglqyil
361 tpfrpapkhp wentyeiisa ftasfqglcv ailfcfcnge viaqmkrkwr mmcfsnrprt
421 nsy***tatqvs***f vrcgpplpge ekvxlkdmsa krrasagpqh hqphhqshql stdqqrar***sq***
481 ***slassss***fle gwrdrmpflk rrqtidhsrq sqplmeegge tvgqaksapg avdrptlmtt
541 iaedvae***tgt aat***shaasts daaaaaavga ggatgaeegh pngmgtvivr meragqqhma
601 deal

V DH31 R PC-like (Genbank Ref # XP_032292141.1) **(stop suppression)**

1 maeqtsnsss sssgsvssss sssgsshiaa esgpdfdalr aacnarlnss hqltgkrrcd
61 mphaachstv qfayklvslg sycagtfdgw lcwpdtaags sayelcpdfi tgfdparyah
121 kecgedgewf khpltnktws nyttcvnldd larnhnvnli yevgysisll aillslails
181 yfkslkcari tlhmnlfssf aansslwliw ylvvvpntel vqlspgycva lhiilhyfll
241 tnyswmlceg fylhtvlvaa fisekklvkw liafgwcspa iviciyglar gftgsweqnl
301 hcwmtdtdfn yilivpvcis iflnllflcn ivrvvllkln apasiqgscg psrtvlqafr
361 atlllvpllg lqymltpfrp gqehpleyty qvisaftasf qglcvatlfc ffngeviaqv
421 krkwrtvcfs nrprtnsy***ta tqvs***fvrcgp pvpgeekvxl kdlsasakrr ssaphrphqq
481 qqqqqqqqqq qqlqqqqqhl laqqqrarsq slgm***tgatss s***srlidgwrh klrllrrpsv
541 dnahqrqplm eetagnsqpq maantqaead rptlmttiae dvaeaattta ***tatgittttt***
601 ***t***nnateqrng tgmglglvsr sglglapgig vvivsvdand qqhiadeal

M Hector R PA (Genbank Ref # AGB93483)

```
  1 mattssdses qnvdvasqaq tqdnlriflk hlyaecvfry qnvtydtddp sfslgpatdy
 61 dsdlpenfsp vprylenaam negvidmrnv deelaekeel matvvsatma tnqkenrlfc
121 plnfdgylcw prtpagtvls qycpdfvegf nrkflahktc lengswyrhp vsnqtwsnyt
181 ncvdyedlef rqfinelyvk gyalsllall isiiiflgfk slrctririh vhlfaslact
241 cvawilwyrl vversetiae nplwciglhl vvhyfmlvny fwmfceglhl hlvlvvvfvk
301 dtivmrwfiv iswfspipia ivyglarhfs spdnkhcwit dslylwifsv pitlsllasf
361 iflinvlrvi vrklhpqsaq paplairkav ratiilvplf glqhfllpyr pdagtqldhf
421 yqmlsvvlvs lqgfvvsflf cfanhdvtfa irtllnkllp slvtpppags ntgqmatttp
481 srelgv
```

V Hector R PA-like (Genbank Ref # XP_032296576)

```
  1 mallraaltm ataayfemtv sgamppqpqd nlrtflkhly aecvyryqnd tataqlatep
 61 ddggllllety tmiprylega vlnegtidmq dvdeeaasen elyatvlsat matnhhshnt
121 semetlycpv nfdgylcwpr tpagtvlsqy cpdfvegfns kflahktcle tgswfrhpvs
181 nqtwsnytnc vdyedfqfrq fvnelyvkgy alsllalfis iviflgfksl rctririhvh
241 lfaslactci awilwyrlvv ehteqiaenp pwcialhlvv hyfmlvnyfw mfceglhlhl
301 vlvvvfvkdt ivmrwfklls wllpllfvlp ygvarhfsan dnahcwmnds fylwifsvpi
361 tlsllasfif linvlrvivr klhpqsaqpa plairkavra tiilvplfgl qhfllpyrpd
421 agtqldrfyq llsvvlvslq gfvvsflfcf anhdvtfamr tllnklmptl vapppagsnt
481 gqlatttpsr elgv
```

M PDF R PA (Genbank Ref # AAF45788)

1 mtllsnildc ggcisaqrft rllrqsgssg pspsaptagt fesksmlept sshslatgrv

61 pllhdfdast tespgtyvld gvarvaqlal eptvmdalpd sdteqvlgnl nssapwnltl

121 asaaatnfen csalfvnytl pqtglycnwt wdtllcwppt pagvlarmnc pggfhgvdtr

181 kfairkceld grwgsrpnat evnppgwtdy gpcykpeiir lmqqmgskdf dayidiarrt

241 rtleivglcl slfalivsll ifctfrslrn nrtkihknlf vamvlqviir ltlyldqfrr

301 gnkeaatnts lsvientpyl ceasyvlley artamfmwmf ieglylhnmv tvavfqgsfp

361 lkffsrlgwc vpilmttvwa rctvmymdts lgeclwnynl tpyywilegp rlavillnfc

421 flvniirvlv mklrq***sqasd ie***qtrkavra aivllpllgi tnllhqlapl ktatnfavws

481 ygthfltsfq gffialiycf lngevravll k***slatqls***vr ghpewapkra smysgaynta

541 pdtdavqpag dpsatgkris ppnkrlngrk pssasivmih epqqrqrlmp rlqnkarekg

601 kdrvektdae aepdptishi hskeagsar***s rtrgs***kwimg icfrgqkvlr vpsassvppe

661 svvfelseq

V PDF R PA-like (Genbank Ref # XP_032288784.1)

1 mptaapssnr tvsthhsttm tststtygtt sttqaaltsf epmavtaagm dvtmagtepl

61 sstsiptfwn ssintasans ydncsalfan ytqpttviyc nwtwdsllcw pptpagatah

121 mhcpagyhgv dtrkfanrkc eldghwagrp nsteqkptgw tdygpcykpe virlmqeikd

181 vnlymdiaqr trtleiiglc lslfaliisl mifcafrslr nnrtkihknl fvamvlqviv

241 rltlyldqfr rgksdsannt slsvientpy lceasyvlle yartamfmwm fieglylhnm

301 itvavfqgnf plvffsllgw gmpvlmtfvw vqctaifmdt slgdclwnyn ltpyywileg

361 prltvimlnf fflvniirvl vmklrq***sqas eie***qtrkavr aaivllpllg itnllhlvpa

421 lktawkfaiw syvthfltsf qgffialiyc flngevravm lksiavwlsv rghpewapkr

481 psmysgaynt apdtdpqlkq gdpqqsgkrl ***sqstkrs***nsr kassvtivis tepqihryvp

541 rrnnnnrast gsarvrgilk ateepasgsa vgqrirstdd gasttgrnsn wmfglcfrgq

601 kvlrvppass vppesvvfel seq

M PDF R PD **(Genbank Ref #** AHN59298**)**

1 mtllsnildc ggcisaqrft rllrqsgssg pspsaptagt fesksmlept sshslatgrv

61 pllhdfdast tespgtyvld gvarvaqlal eptvmdalpd sdteqvlgnl nssapwnltl

121 asaaatnfen csalfvnytl pqtglycnwt wdtllcwppt pagvlarmnc pggfhgvdtr

181 kfairkceld grwgsrpnat evnppgwtdy gpcykpeiir lmqqmgskdf dayidiarrt

241 rtleivglcl slfalivsll ifctfrslrn nrtkihknlf vamvlqviir ltlyldqfrr

301 gnkeaatnts lsvientpyl ceasyvlley artamfmwmf iegl ylhnmv tvavfqgsfp

361 lkffsrlgwc vpilmttvwa rctvmymdts lgeclwnynl tpyywilegp rlavillnfc

421 flvniirvlv mklrq***sqasd*** ***ie***qtrkavra aivllpllgi tnllhqlapl ktatnfavws

481 ygthfltsfq gffialiycf lngevravll k***slatqls***vr ghpewapkra smysgaynta

541 pdtdavqpag dpsatgkris ppnkrlngrk pssasivmih epqqrqrlmp rlqnkarekg

601 kdrvektdae aepdptishi hskeagsar***s*** ***rtrgs***kwimg icfrgqkdkc vmpgsqktqq

661 ifmtsqmppt stlaava***tti*** ***tttstttt***aa k***ttiasiati*** ***at***mtkskaka kaiskshqiq

721 mpka

V PDF R PD-like (Genbank Ref # XP_032288761.1**)**

1 mptaapssnr tvsthhsttm tststtygtt sttqaaltsf epmavtaagm dvtmagtepl

61 sstsiptfwn ssintasans ydncsalfan ytqpttviyc nwtwdsllcw pptpagatah

121 mhcpagyhgv dtrkfanrkc eldghwagrp nsteqkptgw tdygpcykpe virlmqeikd

181 vnlymdiaqr trtleiiglc lslfaliisl mifcafrslr nnrtkihknl fvamvlqviv

241 rltlyldqfr rgksdsannt slsvientpy lceasyvlle yartamfmwm fiegl ylhnm

301 itvavfqgnf plvffsllgw gmpvlmtfvw vqctaifmdt slgdclwnyn ltpyywileg

361 prltvimlnf fflvniirvl vmklrq***sqas*** ***eie***qtrkavr aaivllpllg itnllhlvpa

421 lktawkfaiw syvthfltsf qgffialiyc flngevravm lksiavwlsv rghpewapkr

481 psmysgaynt apdtdpqlkq gdpqqsgkrl sqstkrsnsr kassvtivis tepqihryvp

541 rrnnnnrast gsarvrgilk ateepasgsa vgqrirstdd gasttgrnsn wmfglcfrgq

601 knkcvipnaq vsqqifmtsq lp***tataattt*** ***tttt***vaaaaa a***ttttaat***av aaa***tttsris***

661 saaaaaaaaa aailqkqqtp ka

M Dh44 R1 PA (Genbank Ref # AAF58250)

1 msdhnhidsv nasgsdplld lhnldgiges velqclvqeh ieastygnds ghcltqfdsi

61 lcwprtargt lavlqcmdel qgihydsskn atrfchangt wekytnydac ahlpapesvp

121 efevivelpt iiyyigytls lvslslaliv fayfkelrcl rntihanlff tyimsalfwi

181 lllsvqisir sgvgsciali tlfhfftltn ffwmlvegly lymlvvktfs gdnlrfniya

241 sigwggpalf vvtwavaksl tvtystpeky eincpwmqet hvdwiyqgpv cavliinltf

301 llrimwvlit klr***santvet*** rqyrkaakal lvliplfgit ylvvlagpse sglmghmfav

361 lravllstqg fsvslfycfl nsevrnalrh histwrdtrt iqlnqnrryt tksfskgggs

421 praesmrplt syygrgkre***s*** ***cvssatttt***l vgqhaplslh rgsnnalhtm ptlaanams***s***

481 ***gstls***vmpra isplmrqgle ensv

V Dh44 R1 PA-like (Genbank Ref # XP_032292296.1)

1 mskdnnnnnn nnngnnnpia sealnssvnd alwsldnldg inqsvelhcl lqqqieatty

61 gnasdhcltq fdtilcwprt argtlavlqc mdelqgihyd ssknatrfch sngtwaqytd

121 ydacahlpae tqtvpefeti velptiiyyi gyalslvslt lalivfayyk elrclrntih

181 anlfftyims alfwilllsv qisirsglss cialvtlfhf ftltnffwml veglylymlv

241 vktfsgdnir fniyasigwg gpalfvvtwa vaksltvtyn nnmekydinc pwmeetnvdw

301 ifqgpvcavl iinltfllri mwvlitklr***s*** ***antvet***rqyr kaakallvli plfgitylvv

361 lagpsesglm ghmfavlrav llstqgfwvs lfycflnsev rnalrhhast wrdtrniqrn

421 qnrryttksf skgggsprae smrpltsyyg rgkre***scvss*** ***atttt***lvgqh aplslhrgsn

481 nalhmlptni ag***tgstls***vm praisplmkg leensv

M Dh44 R2 PA (Genbank Ref # AAF58501**)**

1 madddlralv dslddasqed lakvianfsv dmlqrasali gaqqgssggq lqnrtlqcqq
61 qqqreeeqas lealasggkr ilqcpssfds vlcwprtnag slavlpcfee fkgvhydttd
121 natrfcfpng twdhysdydr chqnsgsipv vpdfspnvel paiiyaggyf lsfatlvval
181 iiflsfkdlr clrntihanl fltyitsall wiltlflqvi ttessqagci tlvimfqyfy
241 ltnffwmfve glylytlvvq tfssdnisfi iyaligwgcp avcilvwsia kafaphlene
301 hfngleidca wmreshidwi fkvpaslall vnlvflirim wvlitklr***sa htlet***rqyyk
361 askallvlip lfgityllvl tgpeqgisrn lfeairafli stqgffvalf ycflnsevrq
421 tlrhgftrwr esrnihrnss iknr/rhrask dy***slrsrte***s lrltstspip tghye

V DH44 R2 PA-like (Genbank Ref # XP_032292527.1**)**

1 maedelqalv erlddasaen ianaianfsl emlqrasali gtqqphsgdi linrtledqc
61 kqqaeqqdal yssphlshtg idqysssskas adkptlycpt sfdsvlcwpr tsastwailp
121 cfeefkgvhy dttenatrfc hangtwnhys nysschqqlg svpvvpdfsa svdlpaiiya
181 ggyfisfatl vvaliiflsf kdlrclrnti hanlfltyit sallwiltlf lqvittessq
241 agcitlvimf qyfyltnfsw mfveglylyt lvvqtfssen isfviyalig wgcpalcilf
301 wsiakafash lenehfngle iectwmresh idwifkgpas lailinlvfl irimwvlitk
361 lr***sahtlet***r qyykaskall vliplfgity llvltgpeqg isrnlfeamr afllstqgff
421 valfycflns evrqtlrhrf irwresrnih rssslknr/rh rtskdy***sqrs rte***slrltss
481 spvpaghfe

M DH44 R2 PB (Genbank Ref # AAM68690**)**

1 madddlralv dslddasqed lakvianfsv dmlqrasali gaqqgssggq lqnrtlqcqq
61 qqqreeeqas lealasggkr ilqcpssfds vlcwprtnag slavlpcfee fkgvhydttd
121 natrfcfpng twdhysdydr chqnsgsipv vpdfspnvel paiiyaggyf lsfatlvval
181 iiflsfkdlr clrntihanl fltyitsall wiltlflqvi ttessqagci tlvimfqyfy
241 ltnffwmfve glylytlvvq tfssdnisfi iyaligwgcp avcilvwsia kafaphlene
301 hfngleidca wmreshidwi fkvpaslall vnlvflirim wvlitklr***sa htlet***rqyyk
361 askallvlip lfgityllvl tgpeqgisrn lfeairafli stqgffvalf ycflnsevrq
421 tlrhgftrwr esrnihrnss iknr/steecv iclrpsphtr lgslqryhsi ditdfv

V DH44 R2 PB-like (Genbank Ref # XP_032292526.1**)**

1 maedelqalv erlddasaen ianaianfsl emlqrasali gtqqphsgdi linrtledqc
61 kqqaeqqdal yssphlshtg idqysssskas adkptlycpt sfdsvlcwpr tsastwailp
121 cfeefkgvhy dttenatrfc hangtwnhys nysschqqlg svpvvpdfsa svdlpaiiya
181 ggyfisfatl vvaliiflsf kdlrclrnti hanlfltyit sallwiltlf lqvittessq
241 agcitlvimf qyfyltnfsw mfveglylyt lvvqtfssen isfviyalig wgcpalcilf
301 wsiakafash lenehfngle iectwmresh idwifkgpas lailinlvfl irimwvlitk
361 lr***sahtlet***r qyykaskall vliplfgity llvltgpeqg isrnlfeamr afllstqgff
421 valfycflns evrqtlrhrf irwresrnih rssslknr/st eecviclrps lhtrvgslkh
481 chsidltdfv
