# Supplementary material for: The incidence of candidate binding sites for β-arrestin in Drosophila neuropeptide GPCRs
Source: PLoS One. 2022 Nov 1;17(11):e0275410. doi: 10.1371/journal.pone.0275410 (PMC9624432; doi:10.1371/journal.pone.0275410)
Supplement: S9 Text — (PDF) [file pone.0275410.s013.pdf]

S9. Text Multi-species analysis of Proc-R

Supporting Figure 11

CLUSTAL Line-ups; Genbank Reference IDs below
5th, 6th and 7th Predicted TM domains in YELLOW
BBS sequences in RED

```
Grimshawi         -----MTT-----------------------LTATSTAMPGDGNAAAKMFSDADVAEVRH        32
Mojavensis        --MSTLAA-----------------------ATSTSTAMPVDAIAAAKMFSDADVAEVRH        35
Virilism          ---MTTLA-----------------------ATSTSTAMPVDGIGTAKMFSDADVAEVRH        34
Bipectinate       MTMFSLLTATLTATATATATATVTATATSTESEAEAATLNETEAAGTEMFSDADVVEVRR        60
Anannassae        MTMFSLLTATLTATATATATATATVTPAE--------AETEAEAAGSEMFSDADVAEVRR        52
Serrata           MTMSS-----------------------------TSSQSSDGNETVGEMFSEADTAEVRH        31
Kikkawei          --MSS-----------------------------TSSQSSDGNETVGEMFSEAETAEVRH        29
Fichsuphila       --MSSTSTS------------------TTTATLTATEAATVGNATVGEMFSDAEMAEVRH        40
Eugracilis        MTMSTTSTLTA--------------------TATATATETLGNATVGEMFSDADMAEVRH        40
Rhopaloa          ----------------------------------MEVAATLGNATVGEMFSDADMAEVRH        26
Elegans           --MS----------------------STSTSTEAAPEAATLGNATVGEMFSDADMAEVRH        36
Takahashi         MTMSTTSTATL--------------TEAA--AAAAAEAATLGNATVGEMFSDADMAEVRH        44
Suzuki            MTMSSTSTATL--------------TEAAAEAAAAAEAATLGNATVGEMFSDADMAEVRH        46
Biarmipes         MTMSSTVTATL--------------TEAA--AAAEAEAATLGNATAGEMFSDADMAEVRH        44
Erecta            MTMSSTSTATA--------------TATST----ATATLAEANATVGEMFSDADMAEVRH        42
Melanogaster      MTMSS--TSTA--------------TATST----ATATLDEANATVGEMFSDADMAEVRH        40
Simulans          MTM----SSTS--------------TATST----ATATLAEANATVGEMFSDADMAEVRH        38
Mauritania        MTM----SSTS--------------TATST----ATATLAEANATVGEMFSDADMAEVRH        38
Sechellia         MTM----SSTS--------------TATST----ATATLAEANATVGEMFSDADMAEVRH        38
                                                                 :***:*: .***:

Grimshawi         VVQRILVPCVFVIGLLGNSVSIYVLTRKRMRCTTNIYLSALAITDIAYLTFVLILSLKHY        92
Mojavensis        VVQRILVPCVFVIGLLGNSVSIYVLTRKRMRCTTNIYLTALAITDIAYLTFVLILSLKHY        95
Virilism          VVQRILVPCVFVIGLLGNSVSIYVLTRKRMRCTTNIYLSALAITDIAYLTFVLILSLKHY        94
Bipectinate       VVQRILVPCVFVIGLLGNSVSIYVLTRKRMRCTTNIYLTALAITDIAYLTFQLILSFQHY        120
Anannassae        VVQRILVPCVFVIGLLGNSVSIYVLTRKRMRCTTNIYLSALAITDIAYLTFQLILSLQHY        112
Serrata           VVQRILVPCVFVIGLLGNSVSIYVLTRKRMRCTTNIYLTALAITDIAYLTFQLILSLQHY        91
Kikkawei          VVQRILVPCVFVIGLLGNSVSIYVLTRKRMRCTTNIYLTALAITDIAYLTFQLILSLQHY        89
Fichsuphila       VVQRILVPCVFVIGLLGNSVSIYVLTRKRMRCTTNIYLTALAITDIAYLTCQLILSIQHY        100
Eugracilis        VVQRILVPCVFVIGLLGNSVSIYVLTRKRMRCTTNIYLTALAITDIAYLTCQLILSLQHY        100
Rhopaloa          VVQRILVPCVFVIGLLGNSVSIYVLTRKRMRCTTNIYLTALAITDIAYLTCQLILSLQHY        86
Elegans           VVQRILVPCVFVIGLLGNSVSIYVLTRKRMRCTTNIYLTALAITDIAYLTCQLILSLQHY        96
Takahashi         VVQRILVPCVFVIGLLGNSVSIYVLTRKRMRCTTNIYLTALAITDIAYLTCQLILSLQHY        104
Suzuki            VVQRILVPCVFVIGLLGNSVSIYVLTRKRMRCTTNIYLTALAITDIAYLTCQLILSLQHY        106
Biarmipes         VVQRILVPCVFVIGLLGNSVSIYVLTRKRMRCTTNIYLTALAITDIAYLTCQLILSLQHY        104
Erecta            VVQRILVPCVFVIGLLGNSVSIYVLTRKRMRCTTNIYLTALAITDIAYLTCQLILSLQHY        102
Melanogaster      VVQRILVPCVFVIGLLGNSVSIYVLTRKRMRCTTNIYLTALAITDIAYLTCQLILSLQHY        100
Simulans          VVQRILVPCVFVIGLLGNSVSIYVLTRKRMRCTTNIYLTALAITDIAYLTCQLILSLQHY        98
Mauritania        VVQRILVPCVFVIGLLGNSVSIYVLTRKRMRCTTNIYLTALAITDIAYLTCQLILSLQHY        98
Sechellia         VVQRILVPCVFVIGLLGNSVSIYVLTRKRMRCTTNIYLTALAITDIAYLTCQLILSLQHY        98
                  **************************************:***********  ****::**

Grimshawi         EYIKYHCELYWRLYGFVMWLCDACAYISIYIAVCFTIERFIAIRYPLKRQTFCTESLAKK        152
Mojavensis        EYIKYHCELYWLLYGFVVWLCDACAYISIYIAVCFTIERFIAIRYPLKRQTFCTESLAKK        155
Virilism          EYIKYHCELYWRLYGFIMWLCDACAYISIYIAVCFTIERFIAIRYPLKRQTFCTESLAKK        154
Bipectinate       DYVKYHCEIYWQFYGYFVWLCDSCAYISIYIAVCFTIERFIAIRYPLKRQTFCTESLAKK        180
Anannassae        DYFKYHSEIYWQLYGYVVWLCDSSAYISIYIAVCFTIERFIAIRYPLKRQTFCTESLAKK        172
Serrata           DYVKYHCEIYWQLYGIFVWLCDSSGYISIYIAVCFTIERFIAIRYPLKRQTFCTESLAKK        151
Kikkawei          DYVKYHCEIYWQLYGIFVWLCDSSGYISIYIAVCFTIERFIAIRYPLKRQTFCTESLAKK        149
Fichsuphila       DYAKYHLQIFWELYGYFVWLCDSFGYISIYIAVCFTIERFIAIRYPLKRQTFCTESLAKK        160
Eugracilis        DYTKFHVEIYWQLYGYFVWLCDSFGYISIYIAVCFTIERFIAIRYPLKRQTFCTESLAKK        160
Rhopaloa          DYPKYHFEIYYQLFGYFVWLCDSFGYISIYIAVCFTIERFIAIRYPLKRQTFCTESLAKK        146
Elegans           DYPKYHLEIYYQLYGYFVWLCDSFGYISIYIAVCFTIERFIAIRYPLKRQTFCTESLAKK        156
Takahashi         DYPKYNFKIYWQLYGYFVWLCDSFGYISIYIAVCFTIERFIAIRYPLKRQTFCTESLAKK        164
Suzuki            DYPKYHLKIYWQLYGYFVWLCDSFGYISIYIAVCFTIERFIAIRYPLKRQTFCTESLAKK        166
Biarmipes         DYPKYHLKIYWQLYGYFVWLCDSFGYISIYIAVCFTIERFIAIRYPLKRQTFCTESLAKK        164
Erecta            DYTKYHLKLYWQLYGYFVWLCDSFGYISIYIAVCFTIERFIAIRYPLKRQTFCTESLAKK        162
```

```
Melanogaster     DYPKYHFFKLYWQLYGYFVWLCDSFGYISIYIAVCFTIERFIAIRYPLKRQTFCTESLAKK     160
Simulans         DYPKYHFFKLYWQLYGYFVWLCDSFGYISIYIAVCFTIERFIAIRYPLKRQTFCTESLAKK     158
Mauritania       DYPKYHFFKLYWQLYGYFVWLCDSFGYISIYIAVCFTIERFIAIRYPLKRQTFCTESLAKK     158
Sechellia        DYPKYHFFKLYWQLYGYFVWLCDSFGYISIYIAVCFTIERFIAIRYPLKRQTFCTESLAKK     158
                 :* *:: :::: ::* .:****: .***********************************

Grimshawi        VIAAVALFCLLTTLSTAFEHTYDIDWKLIDGAYRPCNQTLANVSPTPAH-----------     201
Mojavensis       VIAAVALFCLLTTLSTAFEHTYDINWKLIDGAYRPCNLTLANVSPMPTPTPTSTPMPTPA     215
Virilism         VIAAVALFCLLTTLSTAFEHTYDINWKLIDGAYRPCNLTLANVSPTPAQ-----------     203
Bipectinate      VIAAVALFCLLSTLSTAFEHSFARKFRLIDDDYRPCNQTLANVSPQPLALTPSLSMQAPS     240
Anannassae       VIAAVALFCLLSTLSTAFEHSYARGYRLIDDAYRPCNQTLANVSPQPQPLIPSLHMQAPS     232
Serrata          VIAAVSLFCLLSTLSTAFEHTIKVDWKLIDGAYQPCNQTLANVSPTPPSTS---------     202
Kikkawei         VIAAVSLFCLLSTLSTAFEHTIKVDWKLIDGAYQNCNQTLANVSPMPPSTS---------     200
Fichsuphila      VIAAVAVFCLLSTLSTAFEHTITVGWKLIDGAYQPCNQTEANFSPMPPSFALG------T     214
Eugracilis       VIAAVAFFCLLSTLSTAFEHTITVGSKLIDDAYQPCNQTLANYSPMPTS--S--------     210
Rhopaloa         VIAAVALFCLLSTLSTAFEHTITVGYKLIDDAYQPCNLTEANISPTPPAPPF--------     198
Elegans          VIAAVAVFCLLSTLSTAFEHTITVGWKLIDDAYQPCNQTVANFSPTPPVPSF--------     208
Takahashi        VIAAVAIFCLLSTLSTAFEHTITVGWKQIDDAYQPCNQTVANISPTPPP-S---------     214
Suzuki           VIAAVAVFCLLSTLSTAFEHTITVGSKLIDDAYQPCNQTVANISPTPPP-S---------     216
Biarmipes        VIAAVAVFCLLSTLSTAFEHTITVGSRLIDEAYQPCNQSVANISPTPPA-F---------     214
Erecta           VIAAVAIFCLLSTLSTAFEHTITMGSRQIDDAYQPCNQTVANISPTPPPPS---------     213
Melanogaster     VIAAVAIFCLLSTLSTAFEHTITIGTRQIDDAYQPCNQTVANISPMPP-PP---------     210
Simulans         VIAAVAIFCLLSTLSTAFEHTITIGTRQIDDAYQPCNQTLANISPTPPPPP---------     209
Mauritania       VIAAVAIFCLLSTLSTAFEHTITIGTRQIDDAYQPCNQTLANISPTPPPPP---------     209
Sechellia        VIAAVAIFCLLSTLSTAFEHTITIGTRQIDDAYQPCNQTLANISPTPPPPL---------     209
                 *****:.****:********:     : **  *: ** : ** ** *

Grimshawi        -------------QTPGT-AWHVNENASMSN-------TTPS------NVEPPLDLGSGA     234
Mojavensis       -------------QTMTT-AWHIEEPAGNPA-------TSRYLEPFDLSSDSGSGSGSGA     254
Virilism         -------------PTLAT-AWHVDEHANNAA-------TPRYLEPFDLSS----GSGSGA     238
Bipectinate      PLDSTTPLPTAMLPTPVTPHMQDLT-----------RKPEKPLGV--LVSDVFSGGGSGD     287
Anannassae       PSASTTPLPTVMLPTPVTPHWQDSRDSQD----LAGRSTEKPLGGLFLAADVFSGGGSGD     288
Serrata          ---LTPALSTPPLVTPAT-IWQEQDQERESLQDFFTT-ESSSVKSSRLLLDFGSGSG--D     255
Kikkawei         ---VTPALSTPPMVTPPT-IWKEKEL----EQNFFTQ-ESSTAKSSRLLFDFGSGSG--D     249
Fichsuphila      PPLATPPLATPPLATPPT-VWQDFSTES-P---------TAIAGSSSQLIDWGSGSG--D     261
Eugracilis       -VAATPPLATPPLPTPAT-IWQSTELNTES---------TTTAGKSNVLIDWGSGSG--D     257
Rhopaloa         -AVATPPLATPPRPTPAT-I--SQDFTTES---------T-TAGSSNLLVDWGSGSG--D     242
Elegans          -AVATPPLATPPLPTPAT-I--SQHFSTES---------T-TAGSSNLLFDWGSGSG--D     252
Takahashi        -AAATPPLVTPPLPTPAT-IWQSQDLTTES---------T-TAGSSNLLADWGSGSG--D     260
Suzuki           -VAATPPLVTPPLSTPAT-IWQSQDFTTES---------T-TAGSSNLLVDWGSGSG--D     262
Biarmipes        -VAATPPLVTPPLSTPAT-IWKSQDSTTES---------T-TAGSSNLLVDWGSGSG--D     260
Erecta           -AAATPPLATPPLPTPAT-IWQSQDYSTES---------T-TAGSTSLLVDWGSGSGDGD     261
Melanogaster     -VAVTPPLATPPLPTPAT-IWQSPDSAMES---------T-TSGSSNQLVDWGSGSG--D     256
Simulans         -VAATPPLATPPLPTPAT-VWQSPDLAMES---------T-TAGSSNLLVDWGSGSG--D     255
Mauritania       -VAATPPLATPPLPTPAT-AWQSPDFAMES---------T-TAGSSNLLVDWGSGSG--D     255
Sechellia        -GEATPPLATPPLPTPAT-VWQSPDFAMES---------T-TAGSSNLLVDWGSGSG--D     255
                               *  *                                    . *

Grimshawi        -GESDHIPSRQRRLPASTSAFVG----------------VTAPATEPTAAAATVSFLHLS     277
Mojavensis       -GEPDHIPSRQRRLPAFTSASVG----------------VTAAATEPTTASATASLLQLS     297
Virilism         -GESDHIPSRQHRLPASTSASVG----------------VTAAATEPATAAATASLLQLS     281
Bipectinate      GGVPEYKAEKRWPLHSPGFRTTPPMSKVLQQQDIDQDEDQ-----DQNPDHVTESLQFVS     342
Anannassae       GGVPE----KRWHLPSSGFRTTPSMSKVIQEQDIDQD--Q-----DQSPDVVTESLQFHS     337
Serrata          -GEPVNIPRLRRHWQSSGFVTPPTLRRTLQDQDRVLENRGKIGQKQEMVQVTTESLLQLL     314
Kikkawei         -GEPVNIPRLRRHWQSSGFVTPPTLRKTLQDQERELENGGKIGQKQELVQVTTESLLQLL     308
Fichsuphila      -GEPENIPRHRRHWQSTGFVTLPALRRTLEEQEQQVRQPAEEEEEQDEGSRVTESLLQLL     320
Eugracilis       -GEAENIPRHRRQWQSRGFVTLPTLRKTLEEQETDQQQ------DEEQGSSATESLLQLL     310
Rhopaloa         -GEPDNIPRHRRHWQSSGFVTLPNLRKTLEEQDQDQEV-----EEGEQGSRVTESLLQLL     296
Elegans          -GEPENIPRHRRHWQSSGFVTLPNLRKTLEEQDQDEAV-----E--EQGSRVTESLLQLL     304
Takahashi        -GEPDNIPRQRRHWQSSGFVTLPTLRKTLEEQEQDQDVQ---EGEGELGSRVTESLLQLL     316
Suzuki           -GEPENIPRHRRHRQSSGFVTLPTLRKTLEEQDQEG-------DEEEPGSRVTESLLQLL     314
Biarmipes        -GEPENIPRHRRHWHSSGFVTLPTLRKTLEEQDQEG-------EAGEPGSRVTESLLQLL     312
Erecta           -GEPENIPRRRRHWQSSGLVTLPALRKTLEEQDQDQDQE---GGEEEEGSGVTESLLQLL     317
Melanogaster     -GEPENIPRHRRHWQSSGFVTLPTLRKTLEEQDQ----K---VADAAQRSGVTESLLQLW     308
Simulans         -GEPENIPRHRRHWQSSGFVTLPALRKTLEEQDP----E---GADAGQGSGVTESLLQLW     307
Mauritania       -GEPENIPRHRRHWQSSGFVTLPTLRKTLEEQDP----E---GADAGQGSGVTQSLLQLW     307
Sechellia        -GEPENITRHRRHRQSSGFVTLPTLRKTLEEQDP----E---GADAGQGSGVTESLLQLW     307
                  *        :    :                                   .* *:

Grimshawi        RARRNEENYDNDANFKNNYSNNNNNNNNSNSNNNNNNNNNSNKNNSNSSAFEFNVTEYCQNM     337
Mojavensis       RARRSEDNYNYNNDANNNNNN--------NNNDSGNDNNNNNNNNNSAFAFNITEYCQNM     349
```

```
Virilism          RARRSEDNYDSEASSN----------------------NNYNYNNNSAFAFNITEYCQNM      319
Bipectinate       RRLKSL----------------------------------SEVNTTEAFAFNITEYCQNM      368
Anannassae        RRLRST----------------------------------SDVNTTEAFAFNVTEYCQNV      363
Serrata           RRKRSTD-----------------------------------HNITDAFAFNVTEYCQNM      339
Kikkawei          RSKRSTDH----------------------------------HNITDAFAFNVTEYCQNM      334
Fichsuphila       RSKRSADNNNDY--------------------------NNDNSNNTDAFAFNVTEYCQNM      354
Eugracilis        LRSKRSAENNNN-------------------------NNNNNINNTDAFAFNVTEYCQNV      345
Rhopaloa          RRKRNNN-----------------------------------INNTDAFAFNVTEYCQNV      321
Elegans           RRKRNNNDNNNNNNNNNNN--NNNNNNNNNNNNNNNNNNNNNNNNTDAFAFNVTEYCQNV      362
Takahashi         RRKRSAQN--------------------------------SNSNNTDAFAFNVTEYCQNV      344
Suzuki            RRKRSAGNN-------------------------------SN-NNTDAFAFNVTEYCQNV      342
Biarmipes         RRKRSTGNN-------------------------------NNINNTDAFAFNVTEYCQNV      341
Erecta            RRKRRAEN--------------------------------HNINNTDAFAFNVTEYCQNV      345
Melanogaster      RRKRSAEN--------------------------------HNINNTDAFAFNVTEYCQNV      336
Simulans          RRKRSAEI--------------------------------HNINNTDAFAFNVTEYCQNV      335
Mauritania        RRKRSAEI--------------------------------HNINNTDAFAFNVTEYCQNV      335
Sechellia         RRKRSAEI--------------------------------HNINKTDAFAFNVTEYCQNV      335
                     :                                       . ..** **:******:

Grimshawi         TVYANDLSSLGQNALYINIWSVYSLIVFVLLPLLVLATFNCFLILLVHRSKSLRGDLTNA      397
Mojavensis        TFYTNGLSSLGQNALYFNIWSVYTLIVFVLLPLFVLATFNCFLILLVHRSKSLRGDLTNA      409
Virilism          TVYTNGLSSLGQNALYFNIWSVYTLIVFVVLPLFVLATFNCFLILLVHRSKSLRGDLTNA      379
Bipectinate       TFFLHTSSELGMNDLYASVWNLFTLLVFVLLPLLLLATFNSFLILLVHRSKNLRGDLTNA      428
Anannassae        TYFFHASSELGMNYLYASVWNMFTLLVFVLSPLLLLATFNSFLILLVHRSKNLRGDLTNA      423
Serrata           TLYNHGLSELGMNELYGNIWNVFTLLVFVVLPLLLLVTFNSFLILLVHRSKSLRGDLTNA      399
Kikkawei          TLYNHGPSELGMNELYANIWNVFTLLVFVVLPLLLLVTFNSFLILLVHRSKSLRGDLTNA      394
Fichsuphila       TVFGHGYSELGMDELYSNLWSMVTLLIFVVLPLLLLATFNSFLILLVHRSKNLRGDLTNA      414
Eugracilis        TIYNHGLSELGQDELYSNLWNMFTLLVFVVLPLLLLATFNSFLILLVHRSKNLRGDLTNA      405
Rhopaloa          TIYNHGTSALANDELYSNLWNLFTLLVFVVFPLLLLATFNSFLILLVHRSKSLRGDLTNA      381
Elegans           TIYNHGTSELGKDELYSNLWNMFTLLVFVVFPLLLLATFNSFLILLVHRSKSLRGDLTNA      422
Takahashi         TFYNHGLSELGKDELYSNLWNMFTLLVFVVFPLLLLATFNTFLILLVHRSKNLRGDLTNA      404
Suzuki            TFYNHGPSELGMDELYSNLWNMFTLLVFVVFPLLLLATFNTFLILLVHRSKNLRGDLTNA      402
Biarmipes         TFYNHGPSELGMDELYSNLWNLFTLLVFVVLPLLLLATFNTFLILLVHRSKNLRGDLTNA      401
Erecta            TFYNHGLSELGYDELYSYLWNLFTLLVFVVFPLLLLATFNSFLILLVHRSKNLRGDLTNA      405
Melanogaster      TYYNHGLSELGYDELYSYLWNLFTLLVFVVFPLLLLATFNSILILLVHRSKNLRGDLTNA      396
Simulans          TYYNHGLSELGYDELYSYLWNLFTLLVFVVFPLLLLATFNSILILLVHRSKNLRGDLTNA      395
Mauritania        TYYNHGLSELGYDELYSYLWNLFTLLVFVVFPLLLLATFNSILILLVHRSKNLRGDLTNA      395
Sechellia         TYYNHGLSELGYDELYSYLWNLFTLLVFVVFPLLLLATFNSILILLVHRSKNLRGDLTNA      395
                  * : :  * *. : **  :*.: :*::**: **::*.*** :*********.********

Grimshawi         SSMRRTKRKSTSGLTGSVSQENRVTITLIAVVLLFIVCQLPWAIYLILVQYMDIELNIQR      457
Mojavensis        SSIRRTKRKSNSGLTGSVSQENRVTITLIAVVLLFIVCQLPWAIYLILVQYVDIEMNIQR      469
Virilism          SSIRRTKRKSNSGITGSVSQENRVTITLIAVVLLFIVCQLPWAIYLILVQYVEIEMNIQR      439
Bipectinate       NSIRRTKRKSNSGITGTVSQENRVTITLIAVVLLFIVCQLPWAIYLILGQYMEIAPSTQV      488
Anannassae        NSIRRTKRKSNTGITGTVSQENRVTITLIAVVLLFIVCQLPWAIYLILEQYMEIHGSTQV      483
Serrata           SSIRRTKRKSSSGIKGSVSQENRVTITLIAVVLMFIVCQLPWAIYLILNTYMDIQVGTQL      459
Kikkawei          SSIRRTKRKSSSGIKGSVSQENRVTITLIAVVLMFIVCQLPWAIYLILSTYMEIQVGTQL      454
Fichsuphila       SSIRRTKRKSNSGIKSSVSQEHRVTITLIAVVLMFIVCQLPWAIYLVVEQYMTIQVSTQV      474
Eugracilis        SSIRRTKRKSNTGIKGSVSQENRVTITLIAVVLMFIVCQLPWAIYLVVNQYMDIQVGTQV      465
Rhopaloa          SSIRRTKRKSNSGLKGSVSQENRVTITLIAVVLMFIVCQLPWAIYLVLSQYMNFQLGTQV      441
Elegans           SSIRRTKRKSNSGIKGSVSQENRVTITLIAVVLMFIFCQLPWAIYLILSQYMDFQIGTQV      482
Takahashi         SSIRRTKRKSNSGIKGSVSQENRVTITLIAVVLMFIVCQLPWAIYLVVNQYMDIQVGTQV      464
Suzuki            SSIRRTKRKSSSGIKGSVSQENRVTITLIAVVLMFIVCQLPWAIYLVVNQYMDIQVGTQV      462
Biarmipes         SSIRRTKRKSSSGIKGSVSQENRVTITLIAVVLMFIVCQLPWAIYLVVNQYMDIQFGTQV      461
Erecta            SSIRRTKRKSNSGLKGSVSQENRVTITLIAVVLMFIVCQLPWAIYLIVNQYKEIQVGTQV      465
Melanogaster      SSIRRTKRKSNSGLKGSVSQENRVTITLIAVVLMFIVCQLPWAIYLIVNQYMEIQIGTQV      456
Simulans          SSIRRTKRKSNSGLKGSVSQENRVTITLIAVVLMFIVCQLPWAIYLIVNQYMEIQVGTQV      455
Mauritania        SSIRRTKRKSNSGLKGSVSQENRVTITLIAVVLMFIVCQLPWAIYLIVNQYMEIQVGTQV      455
Sechellia         SSIRRTKRKSNSGLKGSVSQENRVTITLIAVVLMFIVCQLPWAIYLIVNQYMEIQVGTQV      455
                  .*:*******.:*:..:****:***********:**.*********::  *  :  . *

Grimshawi         IAGNVCNLLVAINAAANFFLYCVLSDKYRKTVRELITGYRYHHRHARNNFSLYMPHTTTT      517
Mojavensis        IAGNVCNLLVAINAAANFFLYCVLSDKYRKTVRELVTGYRYRHRHARNNISLYVPHTSTT      529
Virilism          IAGNVCNLLVAINAAANFFLYCVLSDKYRKTVRELITGYRYRHRHARNNISLYAPHTTTT      499
Bipectinate       VAGNVCNLLAAFNAASNFFLYCVLSDKYRKTVRELITGYRYRRRHMRNNTSLYVPHTTTT      548
Anannassae        VAGNICNLLAAFNAASNFFLYCVLSDKYRKTVRELITGYRYRRRHMRNNTSLYVPHTTTT      543
Serrata           VAGNVFNLLAALNAASNFFLYCVLSDKYRKTVRELITGYRYRRRHARNNTSLYVPHTTTT      519
Kikkawei          VAGNVFNLLAALNAASNFFLYCVLSDKYRKTVRELITGYRYRRRHARNNTSLYVPHTTTT      514
Fichsuphila       VAGNVCNLLASLHAASNFFLYCVLSDKYRKTVRELITGYRYRHRHNRNNTSLYVPHTTTT      534
Eugracilis        VAGNVCNLLASLHAASNFFLYCVLSDKYRKTVRELITGYRYRRRHARNNTSLYVPHTTTT      525
Rhopaloa          VAGNVCNLLASLHAASNFFLYCVLSDKYRKTVRELITGYRYRRRHIRNNTSLYVPQTTTT      501
```

```
Elegans           VAGNVCNLLASLHAASNFFLYCVLSDKYRKTVRELITGYRYRRRHARNNTSVYVPHTTTT        542
Takahashi         VAGNVCNLLASLHAASNFFLYCVLSDKYRKTVRELITGYRYRRRHARNNTSLYVPHTTTT        524
Suzuki            VAGNVCNLLASLHAASNFFLYCVLSDKYRKTVRELITGYRYKRRHARNNTSLYVPQTTTT        522
Biarmipes         VAGNVCNLLASLHAASNFFLYCVLSDKYRKTVRELITGYRYKRRHARNNTSLYVPQTTTT        521
Erecta            VAGNVCNLLASLHAASNFFLYCVLSDKYRKTVRELITGYRYRRRHARNNTSLYVPHTTTT        525
Melanogaster      VAGNVCNLLASLHAASNFFLYCVLSDKYRKTVRELITGYRYRRRHARNNTSLYVPHTTTT        516
Simulans          VAGNVCNLLASLHAASNFFLYCVLSDKYRKTVRELITGYRYRRRHARNNTSLYVPHTTTT        515
Mauritania        VAGNVCNLLASLHAASNFFLYCVLSDKYRKTVRELITGYRYRRRHARNNTSLYVPHTTTT        515
Sechellia         VAGNVCNLLASLHAASNFFLYCVLSDKYRKTVRELITGYRYRRRHARNNTSLYVPHTTTT        515
                  :***: ***.:::**:******************:*****::** *** *:* *:*:**

Grimshawi         TNGDSASASGGASGYGSYRNANSRRCR------PTGRLIA        551
Mojavensis        LNGDGASSVGSGGGYNAYGGASSRRCRGKAAV--ARRLIA        567
Virilism          LNGDGAGGGGASGYGSSYSGASSRRCRAKSAV--ARRLIA        537
Bipectinate       LTHINGDR---GGGGSYYGGAGNRRSRNKSA--ALGRLIA        583
Anannassae        LTHINGDR---GGGGSYYGGAGNRRSQNKSA--AMGRLIA        578
Serrata           LSHINGHD--------NYGGTGSRRSRNNNKSMATGRLIA        551
Kikkawei          LSHINGHD--H--GGSHYGGAGSRRSRNNNKSMATGRLIA        550
Fichsuphila       LTQINGDH—YGGGGGHYGGAGSRRTRN------TTGRLIA        567
Eugracilis        LTQINGDH--YG---SNYGGAGSRRNR------NINRLIA        554
Rhopaloa          LTQINGDH--YG---SNYGGAGSRRSR------NTSRLIT        530
Elegans           LTQINGDH--YG---SNYGGAGSRRSR------NTSRLIT        571
Takahashi         LTQINGDH--YG---SNYGGAGSRRNR------NTNRLIA        553
Suzuki            LTQINGDH--YG---SNYGGAGSRRNR------NTNRLIA        551
Biarmipes         LTQINGDH--YG---SNYGGAGSRRNR------NTNRLIA        550
Erecta            LTQINGDH--YG---SNYGGAGSRRNR------NTARLIA        554
Melanogaster      LTQINGDH--YG---SNYGGAGSRRNR------NTGRLIA        545
Simulans          LTQINGDH--YG---SNYGGNGSRRNR------NTGRLIA        544
Mauritania        LTQINGDH--YG---SNYGGNGSRRNR------NTGRLIA        544
Sechellia         LTQINGDH--YG---SNYGGNGSRRNR------NTGRLIA        544
```

Melanogaster **NP_001014723.1**

```
1 mtmsststat atstatatld eanatvgemf sdadmaevrh vvqrilvpcv fvigllgnsv
      61 siyvltrkrm rcttniylta laitdiaylt cqlilslqhy dypkyhfkly wqlygyfvwl
     121 cdsfgyisiy iavcftierf iairyplkrq tfcteslakk viaavaifcl lstlstafeh
     181 titigtrqid dayqpcnqtv anispmpppp vavtpplatp plptpatiwq spdsamestt
     241 sgssnqlvdw gsgsgdgepe niprhrrhwq ssgfvtlptl rktleeqdqk vadaaqrsgv
     301 tesllqlwrr krsaenhnin ntdafafnvt eycqnvtyyn hglselgyde lysylwnlft
     361 llvfvvfpll llatfnsili llvhrsknlr gdltnassir rtkrksnsgl kgsvsqenrv
     421 titliavvlm fivcqlpwai ylivnqymei qigtqvvagn vcnllaslha asnfflycvl
     481 sdkyrktvre litgyryrrr harnntslyv phttttltqi ngdhygsnyg gagsrrnrnt
     541 grlia
```

Simulans **XP_016038098.1**

```
  1 mtmsststat statatlaea natvgemfsd admaevrhvv qrilvpcvfv igllgnsvsi
      61 yvltrkrmrc ttniyltala itdiayltcq lilslqhydy pkyhfklywq lygyfvwlcd
     121 sfgyisiyia vcftierfia iryplkrqtf cteslakkvi aavaifclls tlstafehti
     181 tigtrqidda yqpcnqtlan isptppppv aatpplatpp lptpatvwqs pdlamestta
     241 gssnllvdwg sgsgdgepen iprhrrhwqs sgfvtlpalr ktleeqdpeg adagqgsgvt
     301 esllqlwrrk rsaeihninn tdafafnvte ycqnvtyynh glselgydel ysylwnlftl
     361 lvfvvfplll latfnsilil lvhrsknlrg dltnassirr tkrksnsglk gsvsqenrvt
     421 itliavvlmf ivcqlpwaiy livnqymeiq vgtqvvagnv cnllaslhaa snfflycvls
     481 dkyrktvrel itgyryrrrh arnntslyvp httttltqin gdhygsnygg ngsrrnrntg
     541 rlia
```

Suzuki **XP_016939418.1**

```
           1 mtmsststat lteaaaeaaa aaeaatlgna tvgemfsdad maevrhvvqr ilvpcvfvig
      61 llgnsvsiyv ltrkrmrctt niyltalait diayltcqli lslqhydypk yhlkiywqly
     121 gyfvwlcdsf gyisiyiavc ftierfiair yplkrqtfct eslakkviaa vavfcllstl
     181 stafehtitv gskliddayq pcnqtvanis ptpppsvaat pplvtpplst patiwqsqdf
     241 ttesttagss nllvdwgsgs gdgepenipr hrrhrqssgf vtlptlrktl eeqdqegdee
     301 epgsrvtesl lqllrrkrsa gnnsnnntda fafnvteycq nvtfynhgps elgmdelysn
     361 lwnmftllvf vvfpllllat fntflillvh rsknlrgdlt nassirrtkr ksssgikgsv
     421 sqenrvtitl iavvlmfivc qlpwaiylvv nqymdiqvgt qvvagnvcnl laslhaasnf
     481 flycvlsdky rktvrelitg yrykrrharn ntslyvpqtt ttltqingdh ygsnyggags
```

```
541 rrnrntnrli a
```

Mauritania **XP_033170840.1**

```
  1 mtmsststat statatlaea natvgemfsd admaevrhvv qrilvpcvfv igllgnsvsi
 61 yvltrkrmrc ttniyltala itdiayltcq lilslqhydy pkyhfklywq lygyfvwlcd
121 sfgyisiyia vcftierfia iryplkrqtf cteslakkvi aavaifclls tlstafehti
181 tigtrqidda yqpcnqtlan isptpppppv aatpplatpp lptpatawqs pdfamestta
241 gssnllvdwg sgsgdgepen iprhrrhwqs sgfvtlptlr ktleeqdpeg adagqgsgvt
301 qsllqlwrrk rsaeihninn tdafafnvte ycqnvtyynh glselgydel ysylwnlftl
361 lvfvvfplll latfnsilil lvhrsknlrg dltnassirr tkrksnsglk gsvsqenrvt
421 itliavvlmf ivcqlpwaiy livnqymeiq vgtqvvagnv cnllaslhaa snfflycvls
481 dkyrktvrel itgyryrrrh arnntslyvp httttltqin gdhygsnygg ngsrrnrntg
541 rlia
```

Sechellia **XP_002036980.1**

```
  1 mtmsststat statatlaea natvgemfsd admaevrhvv qrilvpcvfv igllgnsvsi
 61 yvltrkrmrc ttniyltala itdiayltcq lilslqhydy pkyhfklywq lygyfvwlcd
121 sfgyisiyia vcftierfia iryplkrqtf cteslakkvi aavaifclls tlstafehti
181 tigtrqidda yqpcnqtlan isptpppplg eatpplatpp lptpatvwqs pdfamestta
241 gssnllvdwg sgsgdgepen itrhrrhrqs sgfvtlptlr ktleeqdpeg adagqgsgvt
301 esllqlwrrk rsaeihnink tdafafnvte ycqnvtyynh glselgydel ysylwnlftl
361 lvfvvfplll latfnsilil lvhrsknlrg dltnassirr tkrksnsglk gsvsqenrvt
421 itliavvlmf ivcqlpwaiy livnqymeiq vgtqvvagnv cnllaslhaa snfflycvls
481 dkyrktvrel itgyryrrrh arnntslyvp httttltqin gdhygsnygg ngsrrnrntg
541 rlia
```

Serrata **KAH8385187.1**

```
  1 mtmsststssqs sdgnetvgem fseadtaevr hvvqrilvpc vfvigllgns vsiyvltrkr
 61 mrcttniylt alaitdiayl tfqlilslqh ydyvkyhcei ywqlygifvw lcdssgyisi
121 yiavcftier fiairyplkr qtfcteslak kviaavslfc llstlstafe htikvdwkli
181 dgayqpcnqt lanvsptpps tsltpalstp plvtpatiwq eqdqereslq dfftesssv
241 kssrllldfg sgsgdgepvn iprlrrhwqs sgfvtpptlr rtlqdqdrvl enrgkigqkq
301 emvqvttesl lqllrrkrst dhnitdafaf nvteycqnmt lynhglselg mnelygniwn
361 vftllvfvvl plllvtfns flillvhrsk slrgdltnas sirrtkrkss sgikgsvsqe
421 nrvtitliav vlmfivcqlp waiylilnty mdiqvgtqlv agnvfnllaa lnaasnffly
481 cvlsdkyrkt vrelitgyry rrrharnnts lyvphttttl shinghdnyg gtgsrrsrnn
541 nksmatgrli a
```

Erecta **XP_001976911.1**

```
  1 mtmsststat atatstatat laeanatvge mfsdadmaev rhvvqrilvp cvfvigllgn
 61 svsiyvltrk rmrcttniyl talaitdiay ltcqlilslq hydytkyhlk lywqlygyfv
121 wlcdsfgyis iyiavcftie rfiairyplk rqtfctesla kkviaavaif cllstlstaf
181 ehtitmgsrq iddayqpcnq tvanisptpp ppsaaatppl atpplptpat iwqsqdyste
241 sttagstsll vdwgsgsgdg dgepeniprr rrhwqssglv tlpalrktle eqdqdqdqeg
301 geeeegsgvt esllqllrrk rraenhninn tdafafnvte ycqnvtfynh glselgydel
361 ysylwnlftl lvfvvfplll latfnsflil lvhrsknlrg dltnassirr tkrksnsglk
421 gsvsqenrvt itliavvlmf ivcqlpwaiy livnqykeiq vgtqvvagnv cnllaslhaa
481 snfflycvls dkyrktvrel itgyryrrrh arnntslyvp httttltqin gdhygsnygg
541 agsrrnrnta rlia
```

Takahashi **XP_017013816.2**

```
  1 mtmsttstat lteaaaaaaa eaatlgnatv gemfsdadma evrhvvqril vpcvfvigll
 61 gnsvsiyvlt rkrmrcttni yltalaitdi ayltcqlils lqhydypkyn fkiywqlygy
121 fvwlcdsfgy isiyiavcft ierfiairyp lkrqtfctes lakkviaava ifcllstlst
181 afehtitvgw kqiddayqpc nqtvanispt pppsaaatpp lvtpplptpa tiwqsqdltt
241 esttagssnl ladwgsgsgd gepdniprqr rhwqssgfvt lptlrktlee qeqdqdvqeg
301 egelgsrnvte sllqllrkr saqnsnsnnt dafafnvtey cqnvtfynhg lselgkdely
361 snlwnmftll vfvvfpllll atfntflill vhrsknlrgd ltnassirrt krksnsgikg
421 svsqenrvti tliavvlmfi vcqlpwaiyl vvnqymdiqv gtqvvagnvc nllaslhaas
481 nfflycvlsd kyrktvreli tgyryrrrha rnntslyvph ttttltqing dhygsnygga
541 gsrrnrntnr lia
```

Biarmipes **XP_016946298.1**

```
1 mtmsstvtat lteaaaaaea eaatlgnata gemfsdadma evrhvvqril vpcvfvigll
       61 gnsvsiyvlt rkrmrcttni yltalaitdi ayltcqlils lqhydypkyh lkiywqlygy
      121 fvwlcdsfgy isiyiavcft ierfiairyp lkrqtfctes lakkviaava vfcllstlst
      181 afehtitvgs rlideayqpc nqsvanispt ppafvaatpp lvtpplstpa tiwksqdstt
      241 esttagssnl lvdwgsgsgd gepeniprhr rhwhssgfvt lptlrktlee qdqegeagep
      301 gsrvtesllq llrrkrstgn nnninntdaf afnvteycqn vtfynhgpse lgmdelysnl
      361 wnlftllvfv vlpllllatf ntflillvhr sknlrgdltn assirrtkrk sssgikgsvs
      421 qenrvtitli avvlmfivcq lpwaiylvvn qymdiqfgtq vvagnvcnll aslhaasnff
      481 lycvlsdkyr ktvrelitgy rykrrharnn tslyvpqttt tltqingdhy gsnyggagsr
      541 rnrntnrlia
```

Eugracilis **XP_017074826.1**

```
1 mtmsttstlt atatatatet lgnatvgemf sdadmaevrh vvqrilvpcv fvigllgnsv
       61 siyvltrkrm rcttniylta laitdiaylt cqlilslqhy dytkfhveiy wqlygyfvwl
      121 cdsfgyisiy iavcftierf iairyplkrq tfcteslakk viaavaffcl lstlstafeh
      181 titvgsklid dayqpcnqtl anyspmptss vaatpplatp plptpatiwq stelntestt
      241 tagksnvlid wgsgsgdgea eniprhrrqw qsrgfvtlpt lrktleeqet dqqqdeeqgs
      301 satesllqll lrskrsaenn nnnnnnninn tdafafnvte ycqnvtiynh glselgqdel
      361 ysnlwnmftl lvfvvlplll latfnsflil lvhrsknlrg dltnassirr tkrksntgik
      421 gsvsqenrvt itliavvlmf ivcqlpwaiy lvvnqymdiq vgtqvvagnv cnllaslhaa
      481 snfflycvls dkyrktvrel itgyryrrrh arnntslyvp httttltqin gdhygsnygg
      541 agsrrnrnin rlia
```

Rhopaloa **XP_044317201.1**

```
        1 mevaatlgna tvgemfsdad maevrhvvqr ilvpcvfvig llgnsvsiyv ltrkrmrctt
       61 niyltalait diayltcqli lslqhydypk yhfeiyyqlf gyfvwlcdsf gyisiyiavc
      121 ftierfiair yplkrqtfct eslakkviaa valfcllstl stafehtitv gykliddayq
      181 pcnlteanis ptppappfav atpplatppr ptpatisqdf ttesttagss nllvdwgsgs
      241 gdgepdnipr hrrhwqssgf vtlpnlrktl eeqdqdqeve egeqgsrvte sllqllrrkr
      301 nnninntdaf afnvteycqn vtiynhgtsa landelysnl wnlftllvfv vfpllllatf
      361 nsflillvhr skslrgdltn assirrtkrk snsglkgsvs qenrvtitli avvlmfivcq
      421 lpwaiylvls qymnfqlgtq vvagnvcnll aslhaasnff lycvlsdkyr ktvrelitgy
      481 ryrrrhirnn tslyvpqttt tltqingdhy gsnyggagsr rsrntsrlit
```

Fichsuphila **XP_017038922.1**

```
            1 mssststttt atltateaat vgnatvgemf sdaemaevrh vvqrilvpcv fvigllgnsv
       61 siyvltrkrm rcttniylta laitdiaylt cqlilsiqhy dyakyhlqif welygyfvwl
      121 cdsfgyisiy iavcftierf iairyplkrq tfcteslakk viaavavfcl lstlstafeh
      181 titvgwklid gayqpcnqte anfspmppsf algtpplatp platpplatp ptvwqdfste
      241 sptaiagsss qlidwgsgsg dgepeniprh rrhwqstgfv tlpalrrtle eqeqqvrqpa
      301 eeeeeqdegs rvtesllqll rskrsadnnn dynndnsnnt dafafnvtey cqnmtvfghg
      361 yselgmdely snlwsmvtll ifvvlpllll atfnsflill vhrsknlrgd ltnassirrt
      421 krksnsgiks svsqehrvti tliavvlmfi vcqlpwaiyl vveqymtiqv stqvvagnvc
      481 nllaslhaas nfflycvlsd kyrktvreli tgyryrhrhn rnntslyvph ttttltqing
      541 dhyggggghy ggagsrrtrn ttgrlia
```

Elegans **XP_017114263.2**

```
1 msststStea apeaatlgna tvgemfsdad maevrhvvqr ilvpcvfvig llgnsvsiyv
       61 ltrkrmrctt niyltalait diayltcqli lslqhydypk yhleiyyqly gyfvwlcdsf
      121 gyisiyiavc ftierfiair yplkrqtfct eslakkviaa vavfcllstl stafehtitv
      181 gwkliddayq pcnqtvanfs ptppvpsfav atpplatppl ptpatisqhf stesttagss
      241 nllfdwgsgs gdgepenipr hrrhwqssgf vtlpnlrktl eeqdqdeave eqgsrvtesl
      301 lqllrrkrnn ndnnnnnnnn nnnnnnnnnn nnnnnnnnnn nnnnnnntda fafnvteycq
      361 nvtiynhgts elgkdelysn lwnmftllvf vvfpllllat fnsflillvh rskslrgdlt
      421 nassirrtkr ksnsgikgsv sqenrvtitl iavvlmfifc qlpwaiylil sqymdfqigt
      481 qvvagnvcnl laslhaasnf flycvlsdky rktvrelitg yryrrrharn ntsvyvphtt
      541 ttltqingdh ygsnyggags rrsrntsrli t
```

Kikkawei **XP_017024118.1**

```
  1 msstssqssd gnetvgemfs eaetaevrhv vqrilvpcvf vigllgnsvs iyvltrkrmr
       61 cttniyltal aitdiayltf qlilslqhyd yvkyhceiyw qlygifvwlc dssgyisiyi
      121 avcftierfi airyplkrqt fcteslakkv iaavslfcll stlstafeht ikvdwklidg
      181 ayqncnqtla nvspmppsts vtpalstppm vtpptiwkek eleqnfftqe sstakssrll
```

241 fdfgsgsgdg epvniprlrr hwqssgfvtp ptlrktlqdq erelenggki gqkqelvqvt
301 tesllqllrs krstdhhnit dafafnvtey cqnmtlynhg pselgmnely aniwnvftll
361 vfvvlplll vtfnsflill vhrskslrgd ltnassirrt krksssgikg svsqenrvti
421 tliavvlmfi vcqlpwaiyl ilstymeiqv gtqlvagnvf nllaalnaas nfflycvlsd
481 kyrktvreli tgyryrrrha rnntslyvph ttttlshing hdhggshygg agsrrsrnnn
541 ksmatgrlia

Bipectinate XP_043066026.1
1 mtmfslltat ltatatatat atvtatatst eseaeaatln eteaagtemf sdadvvevrr
61 vvqrilvpcv fvigllgnsv siyvltrkrm rcttniylta laitdiaylt fqlilsfqhy
121 dyvkyhceiy wqfygyfvwl cdscayisiy iavcftierf iairyplkrq tfcteslakk
181 viaavalfcl lstlstafeh sfarkfrlid ddyrpcnqtl anvspqplal tpslsmqaps
241 pldsttplpt amlptpvtph mqdltrkpek plgvlvsdvf sgggsgdggv peykaekrwp
301 lhspgfrttp pmskvlqqqd idqdedqdqn pdhvteslqf vsrrlkslse vntteafafn
361 iteycqnmtf flhtsselgm ndlyasvwnl ftllvfvllp llllatfnsf lillvhrskn
421 lrgdltnans irrtkrksns gitgtvsqen rvtitliavv llfivcqlpw aiylilgqym
481 eiapstqvva gnvcnllaaf naasnfflyc vlsdkyrktv relitgyryr rrhmrnntsl
541 yvphttttlt hingdrgggg syyggagnrr srnksaalgr lia

Anannassae XP_032309300.1
1 mtmfslltat ltatatatat atatvtpaea eteaeaagse mfsdadvaev rrvvqrilvp
61 cvfvigllgn svsiyvltrk rmrcttniyl salaitdiay ltfqlilslq hydyfkyhse
121 iywqlygyvv wlcdssayis iyiavcftie rfiairyplk rqtfctesla kkviaavalf
181 cllstlstaf ehsyargyrl iddayrpcnq tlanvspqpq plipslhmqa pspsasttpl
241 ptvmlptpvt phwqdsrdsq dlagrstekp lgglflaadv fsgggsgdgg vpekrwhlps
301 sgfrttpsms kviqeqdidq dqdqspdvvt eslqfhsrrl rstsdvntte afafnvteyc
361 qnvtyffhas selgmnylya svwnmftllv fvlsplllla tfnsflillv hrsknlrgdl
421 tnansirrtk rksntgitgt vsqenrvtit liavvllfiv cqlpwaiyli leqymeihgs
481 tqvvagnicn llaafnaasn fflycvlsdk yrktvrelit gyryrrrhmr nntslyvpht
541 tttlthingd rggggsyygg agnrrsqnks aamgrlia

Mojavensis XP_015016788.1

1 mstlaaatst stampvdaia aakmfsdadv aevrhvvqri lvpcvfvigl lgnsvsiyvl
61 trkrmrcttn iyltalaitd iayltfvlil slkhyeyiky hcelywllyg fvvwlcdaca
121 yisiyiavcf tierfiairy plkrqtfcte slakkviaav alfcllttls tafehtydin
181 wklidgayrp cnltlanvsp mptptptstp mptpaqtmtt awhieepagn patsrylepf
241 dlssdsgsgs gsgagepdhi psrqrrlpaf tsasvgvtaa atepttasat asllqlsrar
301 rsednynynn dannnnnnnn ndsgndnnnn nnnnnsafaf niteycqnmt fytnglsslg
361 qnalyfniws vytlivfvll plfvlatfnc flillvhrsk slrgdltnas sirrtkrksn
421 sgltgsvsqe nrvtitliav vllfivcqlp waiylilvqy vdiemniqri agnvcnllva
481 inaaanffly cvlsdkyrkt vrelvtgyry rhrharnnis lyvphtsttl ngdgassvgs
541 gggynaygga ssrrcrgkaa varrlia

Virilism XP_015026487.1
1 mttlaatsts tampvdgigt akmfsdadva evrhvvqril vpcvfvigll gnsvsiyvlt
61 rkrmrcttni ylsalaitdi ayltfvlils lkhyeyikyh celywrlygf imwlcdacay
121 isiyiavcft ierfiairyp lkrqtfctes lakkviaava lfcllttlst afehtydinw
181 klidgayrpc nltlanvspt paqptlataw hvdehannaa tprylepfdl ssgsgsgage
241 sdhipsrqhr lpastsasvg vtaaatepat aaatasllql srarrsedny dseassnnny
301 nynnnsafaf niteycqnmt vytnglsslg qnalyfniws vytlivfvvl plfvlatfnc
361 flillvhrsk slrgdltnas sirrtkrksn sgitgsvsqe nrvtitliav vllfivcqlp
421 waiylilvqy veiemniqri agnvcnllva inaaanffly cvlsdkyrkt vrelitgyry
481 rhrharnnis lyaphttttl ngdgagggga sgygssysga ssrrcraksa varrlia

Grimshawi XP_043072003.1
1 mttltatsta mpgdgnaaak mfsdadvaev rhvvqrilvp cvfvigllgn svsiyvltrk
61 rmrcttniyl salaitdiay ltfvlilslk hyeyikyhce lywrlygfvm wlcdacayis
121 iyiavcftie rfiairyplk rqtfctesla kkviaavalf cllttlstaf ehtydidwkl
181 idgayrpcnq tlanvsptpa hqtpgtawhv nenasmsntt psnveppldl gsgagesdhi
241 psrqrrlpas tsafvgvtap ateptaaaat vsflhlsrar rneenydnda nfknnysnnn
301 nnnnsnsnnn nnnnnsnknn snssafefnv teycqnmtvy andlsslgqn alyiniwsvy
361 slivfvllpl lvlatfncfl illvhrsksl rgdltnassm rrtkrkstsg ltgsvsqenr
421 vtitliavvl lfivcqlpwa iylilvqymd ielniqriag nvcnllvain aaanfflycv
481 lsdkyrktvr elitgyryhh rharnnfsly mphtttttng dsasasggas gygsyrnans
541 rrcrptgrli a
