# Supplementary material for: The incidence of candidate binding sites for β-arrestin in Drosophila neuropeptide GPCRs
Source: PLoS One. 2022 Nov 1;17(11):e0275410. doi: 10.1371/journal.pone.0275410 (PMC9624432; doi:10.1371/journal.pone.0275410)
Supplement: S10 Text — (PDF) [file pone.0275410.s014.pdf]

S10. Text. Multi-species analysis of Tk-R 86C
Supporting Figure 12

CLUSTAL Line-ups; Genbank Reference IDs below
5th, 6th and 7th Predicted TM domain in YELLOW
BBS sequences in RED

```
Grimshawi        ---------------------MPEIVDTELLVNCTILAVLRFELNSIVNITLLNSLNRTE    39
Bipectinate      ---------------------MSETVDTELLVNCTILAVRRFELNSIVNTTLLSSLNRTE    39
Anannassae       ---------------------MSETVDTELLVNCTILAVRRFELNSIVNTTLLSSLNRSE    39
Serrata          ---------------------MSEIVDTELLVNCTILAVRRFELNSIVNTTLLNSLNRTE    39
Kikkawei         ---------------------MSEIVDTELLVNCTILAVRRFELNSIVNTTLLNSLNRTE    39
Rhopaloa         ---------------------MSEIVDTELLVNCTILAVRRFELNSIVNTTLLGSLNRTE    39
Elegans          ---------------------MSEIVDTELLVNCTILAVRRFELNSIVNTTLLGSLNRTE    39
Suzuki           MWHKCGGIITQRSLIPIKGSRMSEIVDTELLVNCTILAVRRFELNSIVNTTLLGSLNRTE    60
Biarmipes        MWRKCGGIITQRSLIPIKGCRMSEIVDTELLVNCTILAVRRFELNSIVNTTLLGSLNRTE    60
Takahashi        ---------------------MSEIVDTELLVNCTILAVRRFELNSIVNTTLLSSLNRTE    39
Eugracilis       ---------------------MSEIVDTELLVNCTILAVRRFELNSIVNTTLLGSLNRTE    39
Fichsuphila      MWHECSGIITQRPRIVIKVWKMSEIVDTELLVNCTILAVRRFELNSIVNTTLLGTLNRTE    60
Erecta           ---------------------MSEIVDTELLVNCTILAVRRFELNSIVNTTLLGSLNRTE    39
Mauritania       ---------------------MSEIVDTELLVNCTILAVRRFELNSIVNTTLLGSLNRTE    39
Melanogaster     ---------------------MSEIVDTELLVNCTILAVRRFELNSIVNTTLLGSLNRTE    39
Simulans         ---------------------MSEIVDTELLVNCTILAVRRFELNSIVNTTLLGSLNRTE    39
Sechellia        ---------------------MSEIVDTELLVNCTILAVRRFELNSIVNTTLLGSLNRTE    39
Mojavensis       ---------------------MSEIVDTELLVNCTILAVRRFELNSIVNTTLLNSLNRTE    39
Virilis         ---------------------MSEIVDTELLVNCTILAVRRFELNTIVNTTLLNTLNRTE     39
                                      * * ************** *****:*** ***.:***:*

Grimshawi        VVSLLTSIIDNRDNLESINEAKDFLTECLFPSPTRPYELPWEQKTIWAIVFGLMMFVAIA    99
Bipectinate      VVGLLSGIIENRDNLENINEAKDFLTECLFPSPTRPYELPWEQKTIWAIIFGLMMFVAIA    99
Anannassae       VVGLLSGIIENRDNLESINEAKDFLTECLFPSPTRPYELPWEQKTIWAIIFGLMMFVAIA    99
Serrata          VVSLLSSIIDNRDNLGSINEAKDFLTECLFPSPTRPYELPWEQKTIWAIIFGLMMFVAIA    99
Kikkawei         VVSLLSSIIDNPDNLGSINEAKDFLTECLFPSPTRPYELPWEQKTIWAIIFGLMMFVAIA    99
Rhopaloa         VVSLLSSIIDNRDNLDSINEAKDFLTECLFPSPTRPYELPWEQKTIWAIIFGLMMFVAIA    99
Elegans          VVSLLSSIIENRDNLQSINEAKDFLTECLFPSPTRPYELPWEQKTIWAIIFGLMMFVAIA    99
Suzuki           VVSLLSSIIDNRDNLESINEAKDFLTECLFPSPTRPYELPWEQKTIWAIIFGLMMFVAIA    120
Biarmipes        VVSLLSSIIDNRDNLESINEAKDFLTECLFPSPTRPYELPWEQKTIWAIIFGLMMFVAIA    120
Takahashi        VVSLLSSIIDNRDNLESINEAKDFLTECLFPSPTRPYELPWEQKTIWAIIFGLMMFVAIA    99
Eugracilis       VVSLLSSIIDNRDNLESINEAKDFLTECLFPSPTRPYELPWEQKTIWAIIFGLMMFVAIA    99
Fichsuphila      VVSLLSSIIENRDNLQSINEAKDFLTECLFPSPTRPYELPWEQKTIWAIIFGLMMFVAIA    120
Erecta           VVNLLSSIIDNRDNLESINEAKDFLTECLFPSPTRPYELPWEQKTIWAIIFGLMMFVAIA    99
Mauritania       VVSLLSSIIDNRDNLESINEAKDFLTECLFPSPTRPYELPWEQKTIWAIIFGLMMFVAIA    99
Melanogaster     VVSLLSSIIDNRDNLESINEAKDFLTECLFPSPTRPYELPWEQKTIWAIIFGLMMFVAIA    99
Simulans         VVSLLSSIIDNRDNLESINEAKDFLTECLFPSPTRPYELPWEQKTIWAIIFGLMMFVAIA    99
Sechellia        VVSLLSSIIDNRDNLESINEAKDFLTECLFPSPTRPYELPWEQKTIWAIIFGLMMFVAIA    99
Mojavensis       VVSLLSGIIENRDNLGSINEAKDFLTECLFPSPTRPYELPWEQKTIWAIVFGLMMFVAIA    99
Virilis         VVGLLSGIIENRDNLDSINEAKDFLTECLFPSPTRPYELPWEQKTIWAIVFGLMMFVAIA     99
                 **.**:.**:* *** .********************************:**********

Grimshawi        GNGIVLWIVTGHRSMRTVTNYFLLNLSIADLLMSSLNCVFNFIFMVNSDWPFGSIYCTIN    159
Bipectinate      GNGIVLWIVTGHRSMRTVTNYFLLNLSIADLLMSSLNCVFNFIFMVNSDWPFGSIYCTIN    159
Anannassae       GNGIVLWIVTGHRSMRTVTNYFLLNLSIADLLMSSLNCVFNFIFMVNSDWPFGSIYCTIN    159
Serrata          GNGIVLWIVTGHRSMRTVTNYFLLNLSIADLLMSSLNCVFNFIFMLNSDWPFGSIYCTIN    159
Kikkawei         GNGIVLWIVTGHRSMRTVTNYFLLNLSIADLLMSSLNCVFNFIFMLNSDWPFGSIYCTIN    159
Rhopaloa         GNGIVLWIVTGHRSMRTVTNYFLLNLSIADLLMSSLNCVFNFIFMLNSDWPFGSIYCTIN    159
Elegans          GNGIVLWIVTGHRSMRTVTNYFLLNLSIADLLMSSLNCVFNFIFMLNSDWPFGSIYCTIN    159
Suzuki           GNGIVLWIVTGHRSMRTVTNYFLLNLSIADLLMSSLNCVFNFIFMLNSDWPFGSIYCTIN    180
Biarmipes        GNGIVLWIVTGHRSMRTVTNYFLLNLSIADLLMSSLNCVFNFIFMLNSDWPFGSIYCTIN    180
Takahashi        GNGIVLWIVTGHRSMRTVTNYFLLNLSIADLLMSSLNCVFNFIFMLNSDWPFGSIYCTIN    159
Eugracilis       GNGIVLWIVTGHRSMRTVTNYFLLNLSIADLLMSSLNCVFNFIFMLNSDWPFGSIYCTIN    159
Fichsuphila      GNGIVLWIVTGHRSMRTVTNYFLLNLSIADLLMSSLNCVFNFIFMLNSDWPFGSIYCTIN    180
Erecta           GNGIVLWIVTGHRSMRTVTNYFLLNLSIADLLMSSLNCVFNFIFMLNSDWPFGSIYCTIN    159
Mauritania       GNGIVLWIVTGHRSMRTVTNYFLLNLSIADLLMSSLNCVFNFIFMLNSDWPFGSIYCTIN    159
Melanogaster     GNGIVLWIVTGHRSMRTVTNYFLLNLSIADLLMSSLNCVFNFIFMLNSDWPFGSIYCTIN    159
```

```
Simulans          GNGIVLWIVTGHRSMRTVTNYFLLNLSIADLLMSSLNCVFNFIFMLNSDWPFGSIYCTIN     159
Sechellia         GNGIVLWIVTGHRSMRTVTNYFLLNLSIADLLMSSLNCVFNFIFMLNSDWPFGSIYCTIN     159
Mojavensis        GNGIVLWIVTGHRSMRTVTNYFLLNLSIADLLMSSLNCVFNFIFMLNSDWPFGSIYCTIN     159
Virilis          GNGIVLWIVTGHRSMRTVTNYFLLNLSIADLLMSTLNCVFNFIFMVNSDWPFGSIYCTIN      159
                  **********************************:**********:**************

Grimshawi         NFVANVTVSTSVFTLVAISFDRYIAIVHPLKRRTSRRKVRFILVMIWALSCVLSAPCLLY     219
Bipectinate       NFVANVTVSTSVFTLVAISFDRYIAIVHPLKRRTSRRKVRFILVLIWALSCVLSAPCLLY     219
Anannassae        NFVANVTVSTSVFTLVAISFDRYIAIVHPLKRRTSRRKVRFILVLIWALSCVLSAPCLLY     219
Serrata           NFVANVTVSTSVFTLVAISFDRYIAIVHPLKRRTSRRKVRFILVLIWVLSCVLSAPCLLY     219
Kikkawei          NFVANVTVSTSVFTLVAISFDRYIAIVHPLKRRTSRRKVRIILVLIWALSCVLSAPCLLY     219
Rhopaloa          NFVANVTVSTSVFTLVAISFDRYIAIVHPLKRRTSRRKVRIILVLIWALSCVLSAPCLLY     219
Elegans           NFVANVTVSTSVFTLVAISFDRYIAIVHPLKRRTSRRKVRIILVLIWALSCVLSAPCLLY     219
Suzuki            NFVANVTVSTSVFTLVAISFDRYIAIVHPLKRRTSRRKVRIILVLIWALSCVLSAPCLLY     240
Biarmipes         NFVANVTVSTSVFTLVAISFDRYIAIVHPLKRRTSRRKVRIILVLIWALSCVLSAPCLLY     240
Takahashi         NFVANVTVSTSVFTLVAISFDRYIAIVHPLKRRTSRRKVRIILVLIWALSCVLSAPCLLY     219
Eugracilis        NFVANVTVSTSVFTLVAISFDRYIAIVHPLKRRTSRRKVRIILVLIWALSCVLSAPCLLY     219
Fichsuphila       NFVANVTVSTSVFTLVAISFDRYIAIVHPLKRRTSRRKVRIILVLIWVLSCVLSAPCLLY     240
Erecta            NFVANVTVSTSVFTLVAISFDRYIAIVHPLKRRTSRRKVRIILVLIWALSCVLSAPCLLY     219
Mauritania        NFVANVTVSTSVFTLVAISFDRYIAIVHPLKRRTSRRKVRIILVLIWALSCVLSAPCLLY     219
Melanogaster      NFVANVTVSTSVFTLVAISFDRYIAIVHPLKRRTSRRKVRIILVLIWALSCVLSAPCLLY     219
Simulans          NFVANVTVSTSVFTLVAISFDRYIAIVHPLKRRTSRRKVRIILVLIWALSCVLSAPCLLY     219
Sechellia         NFVANVTVSTSVFTLVAISFDRYIAIVHPLKRRTSRRKVRIILVLIWALSCVLSAPCLLY     219
Mojavensis        NFVANVTVSTSVFTLVAISFDRYIAIVHPLKRRTSRRKVRFILVLIWALSCVLSAPCLLY     219
Virilis          NFVANVTVSTSVFTLVAISFDRYIAIVHPLKRRTSRRKVRFILVLIWALSCVLSAPCLLY      219
                  ****************************************:***:**.************

Grimshawi         SSIMTKHYYNGKSRTVCFMMWPDGRYPTSMADYVYNLTILVLTYGIPMIVMLICYTLMGR     279
Bipectinate       SSIMTKHYYNGKSRTVCFMMWPDGRYPTSMADYAYNLIILVLTYGIPMIVMLICYTLMGR     279
Anannassae        SSIMTKHYYNGKSRTVCFMMWPDGRYPTSMADYAYNLIILVLTYGIPMIVMLICYTLMGR     279
Serrata           SSIMTKHYYNGKSRTVCFMMWPDGRYPTSMADYAYNLIILVLTYGIPMIVMLICYSLMGR     279
Kikkawei          SSIMTKQYYNGKSRTVCFMMWPDGRYPTSMADYVYNLIILVLTYGIPMIVMLICYSLMGR     279
Rhopaloa          SSIMTKHYYNGKSRTVCFMMWPDGRYPTSMADYAYNLIILILTYGIPMIVMLICYSLMGR     279
Elegans           SSIMTKHYYNGKSRTVCFMMWPDGRYPTSMADYAYNLIILVLTYGIPMIVMLICYSLMGR     279
Suzuki            SSIMTKHYYNGKSRTVCFMMWPDGRYPTSMADYAYNLIILVLTYGVPMIVMLICYSLMGR     300
Biarmipes         SSIMTKHYYNGKSRTVCFMMWPDGRYPTSMADYAYNLIILVLTYGIPMIVMLICYSLMGR     300
Takahashi         SSIMTKHYYNGKSRTVCFMMWPDGRYPTSMADYAYNLIILVLTYGIPMIVMLICYSLMGR     279
Eugracilis        SSIMTKHYYNGKSRTVCFMMWPDGRYPTSMADYAYNLIILVLTYGIPMIVMLICYSLMGR     279
Fichsuphila       SSIMTKHYYNGKSRTVCFMMWPDGRYPTSMADYAYNLIILVLTYGIPMIVMLICYSLMGR     300
Erecta            SSIMTKHYYNGKSRTVCFMMWPDGRYPTSMADYAYNLIILVLTYGIPMIVMLICYSLMGR     279
Mauritania        SSIMTKHYYNGKSRTVCFMMWPDGRYPTSMADYAYNLIILVLTYGIPMIVMLICYSLMGR     279
Melanogaster      SSIMTKHYYNGKSRTVCFMMWPDGRYPTSMADYAYNLIILVLTYGIPMIVMLICYSLMGR     279
Simulans          SSIMTKHYYNGKSRTVCFMMWPDGRYPTSMADYAYNLIILVLTYGIPMIVMLICYSLMGR     279
Sechellia         SSIMTKHYYNGKSRTVCFMMWPDGRYPTSMADYAYNLIILVLTYGIPMIVMLICYSLMGR     279
Mojavensis        SSIMTKHYYNGKSRTVCFMMWPDGRYPTSMADYVYNLTLLVLTYGIPMIVMLVCYSLMGR     279
Virilis          SSIMTKHYYNGKSRTVCFMMWPDGRYPTSMTDYVYNVTILVLTYGIPMIVMLICYSLMGR      279
                  ******:***********************:**.**: :*:****:******:**:****

Grimshawi         VLWGSRSIGENTDRQMESMKSKRKVVRMFIAVVSIFAICWLPYHLFFIYAYHNNQVASTK     339
Bipectinate       VLWGSRSIGENTDRQMESMKSKRKVVRMFIAIVSIFAICWLPYHLFFIYAYHNNHVASTK     339
Anannassae        VLWGSRSIGENTDRQMESMKSKRKVVRMFIAIVSIFAICWLPYHLFFIYAYHNNHVASTK     339
Serrata           VLWGSRSIGENTDRQMESMKSKRKVVRMFIAIVSIFAICWLPYHLFFIYAYHNNQVASTK     339
Kikkawei          VLWGSRSIGENTDRQMESMKSKRKVVRMFIAIVSIFAICWLPYHLFFIYAYHNNQVASTK     339
Rhopaloa          VLWGSRSIGENTDRQMESMKSKRKVVRMFIAIVSIFAICWLPYHLFFIYAYHNNQVASTK     339
Elegans           VLWGSRSIGENTDRQMESMKSKRKVVRMFIAIVSIFAICWLPYHLFFIYAYHNNQVASTK     339
Suzuki            VLWGSRSIGENTDRQMESMKSKRKVVRMFIAIVSIFAICWLPYHLFFIYAYHNNQVASTK     360
Biarmipes         VLWGSRSIGENTDRQMESMKSKRKVVRMFIAIVSIFAICWLPYHLFFIYAYHNNQVASTK     360
Takahashi         VLWGSRSIGENTDRQMESMKSKRKVVRMFIAIVSIFAICWLPYHLFFIYAYHNNQVASTK     339
Eugracilis        VLWGSRSIGENTDRQMESMKSKRKVVRMFIAIVSIFAICWLPYHLFFIYAYHNNQVASTK     339
Fichsuphila       VLWGSRSIGENTDRQMESMKSKRKVVRMFIAIVSIFAICWLPYHLFFIYAYHNNQVASTK     360
Erecta            VLWGSRSIGENTDRQMESMKSKRKVVRMFIAIVSIFAICWLPYHLFFIYAYHNNQVASTK     339
Mauritania        VLWGSRSIGENTDRQMESMKSKRKVVRMFIAIVSIFAICWLPYHLFFIYAYHNNQVASTK     339
Melanogaster      VLWGSRSIGENTDRQMESMKSKRKVVRMFIAIVSIFAICWLPYHLFFIYAYHNNQVASTK     339
Simulans          VLWGSRSIGENTDRQMESMKSKRKVVRMFIAIVSIFAICWLPYHLFFIYAYHNNQVASTK     339
Sechellia         VLWGSRSIGENTDRQMESMKSKRKVVRMFIAIVSIFAICWLPYHLFFIYAYHNNQVASTK     339
Mojavensis        VLWGSRSIGENTERQIESMKSKRKVVRMFIAIVSIFAICWLPYHMFFIYAYHNNQVASTK     339
Virilis          VLWGSRSIGENTDRQMESMKSKRKVVRMFIAIVSIFAICWLPYHLFFIYAYHNNQVASTK      339
                  ************:**:***************:************:*********:*****

Grimshawi         YVQHMYLGFYWLAMSNAMVNPIIYYWMNKRFRMYFQRIVCCCCLGFIRYRFDSPKSQATN     399
Bipectinate       YVQHMYLGFYWLAMSNAMVNPIIYYWMNKRFRMYFQRIICCCCMGFTRHRFDSPKSRLTN     399
```

```
Anannassae        YVQHMYLGFYWLAMSNAMVNPIIYYWMNKRFRMYFQRIICCCCMGFTRHRFDSPKSRLTN      399
Serrata           YVQHMYLGFYWLAMSNAMVNPIIYYWMNKRFRMYFQRIICCCCVGLTRHRFDSPKSRLTN      399
Kikkawei          YVQHMYLGFYWLAMSNAMVNPIIYYWMNKRFRMYFQRIICCCCVGLTRHRFDSPKSRLTN      399
Rhopaloa          YVQHMYLGFYWLAMSNAMVNPLIYYWMNKRFRMYFQRIICCCCVSLTRHRFDSPKSRLTN      399
Elegans           YVQHMYLGFYWLAMSNAMVNPLIYYWMNKRFRMYFQRIICCCCVGLTRHRFDSPKSRLTN      399
Suzuki            YVQHMYLGFYWLAMSNAMVNPLIYYWMNKRFRMYFQRIICCCCVSLTRHRFDSPKSRLTN      420
Biarmipes         YVQHMYLGFYWLAMSNAMVNPLIYYWMNKRFRMYFQRIICCCCVGLTRHRFDSPKSRLTN      420
Takahashi         YVQHMYLGFYWLAMSNAMVNPLIYYWMNKRFRMYFQRIICCCCVGLTRHRFDSPKSRLTN      399
Eugracilis        YVQHMYLGFYWLAMSNAMVNPLIYYWMNKRFRMYFQRIICCCCVGLTRHRFDSPKSRLTN      399
Fichsuphila       YVQHMYLGFYWLAMSNAMVNPLIYYWMNKRFRMYFQRIICCCCVGLTRHRFDSPKSRLTN      420
Erecta            YVQHMYLGFYWLAMSNAMVNPLIYYWMNKRFRMYFQRIICCCCVGLTRHRFDSPKSRLTN      399
Mauritania        YVQHMYLGFYWLAMSNAMVNPLIYYWMNKRFRMYFQRIICCCCVGLTRHRFDSPKSRLTN      399
Melanogaster      YVQHMYLGFYWLAMSNAMVNPLIYYWMNKRFRMYFQRIICCCCVGLTRHRFDSPKSRLTN      399
Simulans          YVQHMYLGFYWLAMSNAMVNPLIYYWMNKRFRMYFQRIICCCCVGLTRHRFDSPKSRLTN      399
Sechellia         YVQHMYLGFYWLAMSNAMVNPLIYYWMNKRFRMYFQRIICCCCVGLTRHRFDSPKSRLTN      399
Mojavensis        YVQHMYLGFYWLAMSNAMVNPIIYYWMNKRFRMYFQRIIFCCCMGLTRYKFDSPKSRLAN      399
Virilis          YVQHMYLGFYWLAMSNAMVNPIIYYWMNKRFRMYFQRIIFCCCLGLMRYRFESPKSRMAN       399
                  *********************:****************: ***:.: *::*:****: :*

Grimshawi         KNSSHRNTR-------------------------------------------------AE      410
Bipectinate       KNSSNRHTR-------------------------------------------------AE      410
Anannassae        KNSSHRHTR-------------------------------------------------AE      410
Serrata           KNSSHRHTR-------------------------------------------------AE      410
Kikkawei          KNSSHRHTR-------------------------------------------------AE      410
Rhopaloa          KNSSHRHTR-------------------------------------------------AE      410
Elegans           KNSSHRHTR-------------------------------------------------AE      410
Suzuki            KNSSHRHTR-------------------------------------------------AE      431
Biarmipes         KNSSHRHTR-------------------------------------------------AE      431
Takahashi         KNSSHRHTR-------------------------------------------------AE      410
Eugracilis        KNSSHRHTR-------------------------------------------------AE      410
Fichsuphila       KNSSHRHTR-------------------------------------------------AE      431
Erecta            KNSSHRHTR-------------------------------------------------AE      410
Mauritania        KNSSHRHTRGGYTVAHSLPNSSPPTTQTILAVLAQTQPMPQTQLLLSHYSPHPTQPSAAE      459
Melanogaster      KNSSNRHTR-------------------------------------------------AE      410
Simulans          KNSSHRHTR-------------------------------------------------AE      410
Sechellia         KNSSHRHTR-------------------------------------------------AE      410
Mojavensis        KNSSNRNTR-------------------------------------------------VE      410
Virilis          KNSSNRHTR-------------------------------------------------AE       410
                  ****:*:**                                                 .*

Grimshawi         TKSQWKRSTMETQIQQVPTSSSRDR--------------QAGAVQGLNHTAVECIIERP-      455
Bipectinate       TKSQWKRSTMETQIQAPPITHTNSSKDKEREKHLQQV-----QTELHHRKAVECIMEGPG      465
Anannassae        TKSQWKRSTMETQIQAPPITHTNSSKDRERERHLQQ-------TELQHRKAVECIMEGPG      463
Serrata           TKSQWKRSTMETQIQQVPTTNSCRDQRQMTQSQS--L-----GGVATPRPAVECIMERP-      462
Kikkawei          TKSQWKRSTMETQIHQVPTTNSCRDQRQLTQSQS--LGAHSGGGVGTPRPAVECIMERP-      467
Rhopaloa          NKSQWKRSTMETQIQQAPLTNSGRDQRTAQP---------LKAVDGPSRVGVECIMERP-      460
Elegans           NKSQWKRSTMETQIQQAPLTNSGRDPRTPQP---------LKVVDGASRVGVECIMERP-      460
Suzuki            TKSQWKRSTMETQIQQAPVSNSGRDQRMRDGYANQPAQAGVGGAGGPNRAAVECIMERP-      490
Biarmipes         TKSQWKRSTMETQIQQAPVSNSGRDQRMRDGYPS---QAQAGVAGGPNRAAVECIMERP-      487
Takahashi         TKSQWKRSTMETQIQQAPVSNSCRDPRMREATA-QPQTCGVGGP-NANRSAVECIMERP-      467
Eugracilis        TKSQWKRSTMETQIQQAPANSTGRDQRN----A-QQTPTGAGIG-GPNRTAVECIMERP-      463
Fichsuphila       TKSQWKRSTMETQIQQAPLTSSGRDQRN----A-QTQ--SLGGA-GSNRTAVECIMERP-      482
Erecta            TKSQWKRSTMETQIQQAPVTSSGRDQRS----A-QQQQHHPSGA-ATNRAAVECIMERP-      463
Mauritania        TKSQWKRSTMETQIQQAPVTSSCREQRS----A-QQQ--QPPGS-GTNRAAVECIMERP-      510
Melanogaster      TKSQWKRSTMETQIQQAPVTSSCREQRS----A-QQQ--QPPGS-GTNRAAVECIMERP-      461
Simulans          TKSQWKRSTMETQIQQAPVTSSCREQRS----A-QQQ--QPPGS-GTNRAAVECIMERP-      461
Sechellia         TKSQWKRSTMETQIQQAPVTSSCREQRS----A-QQQ--QPPGS-ETNRAAVECIMERP-      461
Mojavensis        TKSQWKRSTMETQIQQVPTTSSRER---------------KAAGAAVNHMAVECVIERP-      454
Virilis          TKSQWKRSTMETQIQQMPKTSSRDK---------------DAGVQGLNHTAVECIIERP-       454
                  .*************:  *  .  :                          : .***::* *

Grimshawi         --VDESGSPICLSINNSAGERQRVKIKYISCDEDNNPIEHESESGHSHGHTHRHSHRHSL      513
Bipectinate       GNADGNSSPLCLSINNSMGERQRVKIKYISCDEDNNPVEHSPKQL---------------      510
Anannassae        GHADGNSSPLCLSINNSVGERQRVKIKYISCDEDNNPVENSPKQL---------------      508
Serrata           --MDGNSSPLCLSINNSVGERQRVKIKYISCDEDNNPVELEHSSGPKQL-----------      509
Kikkawei          --LDGNSSPLCLSINNSVGERQRVKIKYISCDEDNNPVELEHSSGPKQL-----------      514
Rhopaloa          --ADGNSSPLCLSINNSVGERQRVKIKYISCDEDNNPVEHTPKQ----L-----------      503
Elegans           --ADGNSSPLCLSINNSVGERQRVKIKYISCDEDNNPVEHSPKH----L-----------      503
Suzuki            --LDGNSSPLCLSINNSLGERQRMKIKYISCDEDNNPVELSPNQ----L-----------      533
Biarmipes         --LDGNSSPLCLSINNSLGERQRLKIKYISCDEDNNPVELSPNQ----L-----------      530
Takahashi         --VDGTSSPLCLSINNSLGERQRVKIKYISCDEDNNPVELSPKQ----L-----------      510
```

```
Eugracilis        --TDGTSSPLCLSINNSVGERQRVKIKYISCDEDNNPVELSPKQ----L-----------        506
Fichsuphila       --ADGTSSPLCLSINNSVGERQRVKIKYISCDEDNNPVEHSPKQ----L-----------        525
Erecta            --ADGSSSPLCLSINNSIGERQRVKIKYISCDEDNNPVELSPKQ----L-----------        506
Mauritania        --ADGSSSPLCLSINNSIGERQRVKIKYISCDEDNNPVELSPKQ----L-----------        553
Melanogaster      --ADGSSSPLCLSINNSIGERQRVKIKYISCDEDNNPVELSPKQ----M-----------        504
Simulans          --ADGSSSPLCLSINNSIGERQRVKIKYISCDEDNNPVELSPKQ----L-----------        504
Sechellia         --ADGSSSPLCLSINNSIGERQRVKIKYISCDEDNNPVELSPKQ----L-----------        504
Mojavensis        --MDDNSSPICLSINNSAGERQRLRIKYISCDEDNNPIEEASESSSNHSHGHSHNHSRSH        512
Virilis          --IDDNSSPICLSIKNSAGERQRVKIKYISCDEDNNPIEEGSENNSSHDSNHSHGHGHSC        512
                      * ..**:****:** *****::************:*   .
```

Melanogaster **NP_524304.2**

```
  1 mseivdtell vnctilavrr felnsivntt llgslnrtev vsllssiidn rdnlesinea
     61 kdflteclfp sptrpyelpw eqktiwaiif glmmfvaiag ngivlwivtg hrsmrtvtny
    121 fllnlsiadl lmsslncvfn fifmlnsdwp fgsiyctinn fvanvtvsts vftlvaisfd
    181 ryiaivhplk rrtsrrkvri ilvliwalsc vlsapcllys simtkhyyng ksrtvcfmmw
    241 pdgryptsma dyaynliilv ltygipmivm licyslmgrv lwgsrsigen tdrqmesmks
    301 krkvvrmfia ivsifaicwl pyhlffiyay hnnqvastky vqhmylgfyw lamsnamvnp
    361 liyywmnkrf rmyfqriicc ccvgltrhrf dspksrltnk nssnrhtrae tksqwkrstm
    421 etqiqqapvt sscreqrsaq qqqppgsgtn raavecimer padgsssplc lsinnsiger
    481 qrvkikyisc dednnpvels pkqm
```

Simulans **XP_002103960.1**

```
  1 mseivdtell vnctilavrr felnsivntt llgslnrtev vsllssiidn rdnlesinea
     61 kdflteclfp sptrpyelpw eqktiwaiif glmmfvaiag ngivlwivtg hrsmrtvtny
    121 fllnlsiadl lmsslncvfn fifmlnsdwp fgsiyctinn fvanvtvsts vftlvaisfd
    181 ryiaivhplk rrtsrrkvri ilvliwalsc vlsapcllys simtkhyyng ksrtvcfmmw
    241 pdgryptsma dyaynliilv ltygipmivm licyslmgrv lwgsrsigen tdrqmesmks
    301 krkvvrmfia ivsifaicwl pyhlffiyay hnnqvastky vqhmylgfyw lamsnamvnp
    361 liyywmnkrf rmyfqriicc ccvgltrhrf dspksrltnk nsshrhtrae tksqwkrstm
    421 etqiqqapvt sscreqrsaq qqqppgsgtn raavecimer padgsssplc lsinnsiger
    481 qrvkikyisc dednnpvels pkql
```

Suzuki **XP_036674593.1**

```
      1 mwhkcggiit qrslipikgs rmseivdtel lvnctilavr rfelnsivnt tllgslnrte
     61 vvsllssiid nrdnlesine akdflteclf psptrpyelp weqktiwaii fglmmfvaia
    121 gngivlwivt ghrsmrtvtn yfllnlsiad llmsslncvf nfifmlnsdw pfgsiyctin
    181 nfvanvtvst svftlvaisf dryiaivhpl krrtsrrkvr iilvliwals cvlsapclly
    241 ssimtkhyyn gksrtvcfmm wpdgryptsm adyaynliil vltygvpmiv mlicyslmgr
    301 vlwgsrsige ntdrqmesmk skrkvvrmfi aivsifaicw lpyhlffiya yhnnqvastk
    361 yvqhmylgfy wlamsnamvn pliyywmnkr frmyfqriic cccvsltrhr fdspksrltn
    421 knsshrhtra etksqwkrst metqiqqapv snsgrdqrmr dgyanqpaqa gvggaggpnr
    481 aavecimerp ldgnssplcl sinnslgerq rmkikyiscd ednnpvelsp nql
```

Mauritania **XP_033163933.1**

```
1 mseivdtell vnctilavrr felnsivntt llgslnrtev vsllssiidn rdnlesinea
     61 kdflteclfp sptrpyelpw eqktiwaiif glmmfvaiag ngivlwivtg hrsmrtvtny
    121 fllnlsiadl lmsslncvfn fifmlnsdwp fgsiyctinn fvanvtvsts vftlvaisfd
    181 ryiaivhplk rrtsrrkvri ilvliwalsc vlsapcllys simtkhyyng ksrtvcfmmw
    241 pdgryptsma dyaynliilv ltygipmivm licyslmgrv lwgsrsigen tdrqmesmks
    301 krkvvrmfia ivsifaicwl pyhlffiyay hnnqvastky vqhmylgfyw lamsnamvnp
    361 liyywmnkrf rmyfqriicc ccvgltrhrf dspksrltnk nsshrhtrgg ytvahslpns
    421 spttqtila vlaqtqpmpq tqlllshysp hptqpsaaet ksqwkrstme tqiqqapvts
    481 screqrsaqq qqppgsgtnr aavecimerp adgsssplcl sinnsigerq rvkikyiscd
    541 ednnpvelsp kql
```

Sechellia **XP_032578092.1**

```
  1 mseivdtell vnctilavrr felnsivntt llgslnrtev vsllssiidn rdnlesinea
     61 kdflteclfp sptrpyelpw eqktiwaiif glmmfvaiag ngivlwivtg hrsmrtvtny
    121 fllnlsiadl lmsslncvfn fifmlnsdwp fgsiyctinn fvanvtvsts vftlvaisfd
    181 ryiaivhplk rrtsrrkvri ilvliwalsc vlsapcllys simtkhyyng ksrtvcfmmw
    241 pdgryptsma dyaynliilv ltygipmivm licyslmgrv lwgsrsigen tdrqmesmks
    301 krkvvrmfia ivsifaicwl pyhlffiyay hnnqvastky vqhmylgfyw lamsnamvnp
    361 liyywmnkrf rmyfqriicc ccvgltrhrf dspksrltnk nsshrhtrae tksqwkrstm
    421 etqiqqapvt sscreqrsaq qqqppgsetn raavecimer padgsssplc lsinnsiger
```

481 qrvkikyisc dednnpvels pkql

Serrata XP_020798481.1

```
1 mseivdtell vnctilavrr felnsivntt llnslnrtev vsllssiidn rdnlgsinea
      61 kdflteclfp sptrpyelpw eqktiwaiif glmmfvaiag ngivlwivtg hrsmrtvtny
     121 fllnlsiadl lmsslncvfn fifmlnsdwp fgsiyctinn fvanvtvsts vftlvaisfd
     181 ryiaivhplk rrtsrrkvrf ilvliwvlsc vlsapcllys simtkhyyng ksrtvcfmmw
     241 pdgryptsma dyaynliilv ltygipmivm licyslmgrv lwgsrsigen tdrqmesmks
     301 krkvvrmfia ivsifaicwl pyhlffiyay hnnqvastky vqhmylgfyw lamsnamvnp
     361 iiyywmnkrf rmyfqriicc ccvgltrhrf dspksrltnk nsshrhtrae tksqwkrstm
     421 etqiqqvptt nscrdqrqmt qsqslggvat prpavecime rpmdgnsspl clsinnsvge
     481 rqrvkikyis cdednnpvel ehssgpkql
```

Erecta XP_001980652.1

```
1 mseivdtell vnctilavrr felnsivntt llgslnrtev vnllssiidn rdnlesinea
      61 kdflteclfp sptrpyelpw eqktiwaiif glmmfvaiag ngivlwivtg hrsmrtvtny
     121 fllnlsiadl lmsslncvfn fifmlnsdwp fgsiyctinn fvanvtvsts vftlvaisfd
     181 ryiaivhplk rrtsrrkvri ilvliwalsc vlsapcllys simtkhyyng ksrtvcfmmw
     241 pdgryptsma dyaynliilv ltygipmivm licyslmgrv lwgsrsigen tdrqmesmks
     301 krkvvrmfia ivsifaicwl pyhlffiyay hnnqvastky vqhmylgfyw lamsnamvnp
     361 liyywmnkrf rmyfqriicc ccvgltrhrf dspksrltnk nsshrhtrae tksqwkrstm
     421 etqiqqapvt ssgrdqrsaq qqqhhpsgaa tnraavecim erpadgsssp lclsinnsig
     481 erqrvkikyi scdednnpve lspkql
```

Takahashi XP_017004985.2

```
1 mseivdtell vnctilavrr felnsivntt llsslnrtev vsllssiidn rdnlesinea
      61 kdflteclfp sptrpyelpw eqktiwaiif glmmfvaiag ngivlwivtg hrsmrtvtny
     121 fllnlsiadl lmsslncvfn fifmlnsdwp fgsiyctinn fvanvtvsts vftlvaisfd
     181 ryiaivhplk rrtsrrkvri ilvliwalsc vlsapcllys simtkhyyng ksrtvcfmmw
     241 pdgryptsma dyaynliilv ltygipmivm licyslmgrv lwgsrsigen tdrqmesmks
     301 krkvvrmfia ivsifaicwl pyhlffiyay hnnqvastky vqhmylgfyw lamsnamvnp
     361 liyywmnkrf rmyfqriicc ccvgltrhrf dspksrltnk nsshrhtrae tksqwkrstm
     421 etqiqqapvs nscrdprmre ataqpqtcgv ggpnanrsav ecimerpvdg tssplclsin
     481 nslgerqrvk ikyiscdedn npvelspkql
```

Biarmipes XP_016959801.1

```
1 mwrkcggiit qrslipikgc rmseivdtel lvnctilavr rfelnsivnt tllgslnrte
      61 vvsllssiid nrdnlesine akdflteclf psptrpyelp weqktiwaii fglmmfvaia
     121 gngivlwivt ghrsmrtvtn yfllnlsiad llmsslncvf nfifmlnsdw pfgsiyctin
     181 nfvanvtvst svftlvaisf dryiaivhpl krrtsrrkvr iilvliwals cvlsapclly
     241 ssimtkhyyn gksrtvcfmm wpdgryptsm adyaynliil vltygipmiv mlicyslmgr
     301 vlwgsrsige ntdrqmesmk skrkvvrmfi aivsifaicw lpyhlffiya yhnnqvastk
     361 yvqhmylgfy wlamsnamvn pliyywmnkr frmyfqriic cccvgltrhr fdspksrltn
     421 knsshrhtra etksqwkrst metqiqqapv snsgrdqrmr dgypsqaqag vaggpnraav
     481 ecimerpldg nssplclsin nslgerqrlk ikyiscdedn npvelspnql
```

Eugracilis XP_017083539.1

```
1 mseivdtell vnctilavrr felnsivntt llgslnrtev vsllssiidn rdnlesinea
      61 kdflteclfp sptrpyelpw eqktiwaiif glmmfvaiag ngivlwivtg hrsmrtvtny
     121 fllnlsiadl lmsslncvfn fifmlnsdwp fgsiyctinn fvanvtvsts vftlvaisfd
     181 ryiaivhplk rrtsrrkvri ilvliwalsc vlsapcllys simtkhyyng ksrtvcfmmw
     241 pdgryptsma dyaynliilv ltygipmivm licyslmgrv lwgsrsigen tdrqmesmks
     301 krkvvrmfia ivsifaicwl pyhlffiyay hnnqvastky vqhmylgfyw lamsnamvnp
     361 liyywmnkrf rmyfqriicc ccvgltrhrf dspksrltnk nsshrhtrae tksqwkrstm
     421 etqiqqapan stgrdqrnaq qtptgagigg pnrtavecim erptdgtssp lclsinnsvg
     481 erqrvkikyi scdednnpve lspkql
```

Rhopaloa XP_016989968.2

```
1 mseivdtell vnctilavrr felnsivntt llgslnrtev vsllssiidn rdnldsinea
      61 kdflteclfp sptrpyelpw eqktiwaiif glmmfvaiag ngivlwivtg hrsmrtvtny
     121 fllnlsiadl lmsslncvfn fifmlnsdwp fgsiyctinn fvanvtvsts vftlvaisfd
     181 ryiaivhplk rrtsrrkvri ilvliwalsc vlsapcllys simtkhyyng ksrtvcfmmw
```

241 pdgryptsma dyaynliili ltygipmivm licyslmgrv lwgsrsigen tdrqmesmks
301 krkvvrmfia ivsifaicwl pyhlffiyay hnnqvastky vqhmylgfyw lamsnamvnp
361 liyywmnkrf rmyfqriicc ccvsltrhrf dspksrltnk nsshrhtrae nksqwkrstm
421 etqiqqaplt nsgrdqrtaq plkavdgpsr vgvecimerp adgnssplcl sinnsvgerq
481 rvkikyiscd ednnpvehtp kql

Fichsuphila **XP_017057393.1**

1 mwhecsgiit qrprivikvw kmseivdtel lvnctilavr rfelnsivnt tllgtlnrte
61 vvsllssiie nrdnlqsine akdflteclf psptrpyelp weqktiwaii fglmmfvaia
121 gngivlwivt ghrsmrtvtn yfllnlsiad llmsslncvf nfifmlnsdw pfgsiyctin
181 nfvanvtvst svftlvaisf dryiaivhpl krrtsrrkvr iilvliwvls cvlsapclly
241 ssimtkhyyn gksrtvcfmm wpdgryptsm adyaynliil vltygipmiv mlicyslmgr
301 vlwgsrsige ntdrqmesmk skrkvvrmfi aivsifaicw lpyhlffiya yhnnqvastk
361 yvqhmylgfy wlamsnamvn pliyywmnkr frmyfqriic cccvgltrhr fdspksrltn
421 knsshrhtra etksqwkrst metqiqqapl tssgrdqrna qtqslggags nrtavecime
481 rpadgtsspl clsinnsvge rqrvkikyis cdednnpveh spkql

Elegans **XP_017112407.1**

1 mseivdtell vnctilavrr felnsivntt llgslnrtev vsllssiien rdnlqsinea
61 kdflteclfp sptrpyelpw eqktiwaiif glmmfvaiag ngivlwivtg hrsmrtvtny
121 fllnlsiadl lmsslncvfn fifmlnsdwp fgsiyctinn fvanvtvsts vftlvaisfd
181 ryiaivhplk rrtsrrkvri ilvliwalsc vlsapcllys simtkhyyng ksrtvcfmmw
241 pdgryptsma dyaynliilv ltygipmivm licyslmgrv lwgsrsigen tdrqmesmks
301 krkvvrmfia ivsifaicwl pyhlffiyay hnnqvastky vqhmylgfyw lamsnamvnp
361 liyywmnkrf rmyfqriicc ccvgltrhrf dspksrltnk nsshrhtrae nksqwkrstm
421 etqiqqaplt nsgrdprtpq plkvvdgasr vgvecimerp adgnssplcl sinnsvgerq
481 rvkikyiscd ednnpvehsp khl

Kikkawei **XP_017020386.1**

1 mseivdtell vnctilavrr felnsivntt llnslnrtev vsllssiidn pdnlgsinea
61 kdflteclfp sptrpyelpw eqktiwaiif glmmfvaiag ngivlwivtg hrsmrtvtny
121 fllnlsiadl lmsslncvfn fifmlnsdwp fgsiyctinn fvanvtvsts vftlvaisfd
181 ryiaivhplk rrtsrrkvri ilvliwalsc vlsapcllys simtkqyyng ksrtvcfmmw
241 pdgryptsma dyvynliilv ltygipmivm licyslmgrv lwgsrsigen tdrqmesmks
301 krkvvrmfia ivsifaicwl pyhlffiyay hnnqvastky vqhmylgfyw lamsnamvnp
361 iiyywmnkrf rmyfqriicc ccvgltrhrf dspksrltnk nsshrhtrae tksqwkrstm
421 etqihqvptt nscrdqrqlt qsqslgahsg ggvgtprpav ecimerpldg nssplclsin
481 nsvgerqrvk ikyiscdedn npvelehssg pkql

Bipectinate **XP_017104778.2**

1 msetvdtell vnctilavrr felnsivntt llsslnrtev vgllsgiien rdnleninea
61 kdflteclfp sptrpyelpw eqktiwaiif glmmfvaiag ngivlwivtg hrsmrtvtny
121 fllnlsiadl lmsslncvfn fifmvnsdwp fgsiyctinn fvanvtvsts vftlvaisfd
181 ryiaivhplk rrtsrrkvrf ilvliwalsc vlsapcllys simtkhyyng ksrtvcfmmw
241 pdgryptsma dyaynliilv ltygipmivm licytlmgrv lwgsrsigen tdrqmesmks
301 krkvvrmfia ivsifaicwl pyhlffiyay hnnhvastky vqhmylgfyw lamsnamvnp
361 iiyywmnkrf rmyfqriicc ccmgftrhrf dspksrltnk nssnrhtrae tksqwkrstm
421 etqiqappit htnsskdker ekhlqqvqte lhhrkaveci megpggnadg nssplclsin
481 nsmgerqrvk ikyiscdedn npvehspkql

Anannassae **XP_001952989.1**

1 msetvdtell vnctilavrr felnsivntt llsslnrsev vgllsgiien rdnlesinea
61 kdflteclfp sptrpyelpw eqktiwaiif glmmfvaiag ngivlwivtg hrsmrtvtny
121 fllnlsiadl lmsslncvfn fifmvnsdwp fgsiyctinn fvanvtvsts vftlvaisfd
181 ryiaivhplk rrtsrrkvrf ilvliwalsc vlsapcllys simtkhyyng ksrtvcfmmw
241 pdgryptsma dyaynliilv ltygipmivm licytlmgrv lwgsrsigen tdrqmesmks
301 krkvvrmfia ivsifaicwl pyhlffiyay hnnhvastky vqhmylgfyw lamsnamvnp
361 iiyywmnkrf rmyfqriicc ccmgftrhrf dspksrltnk nsshrhtrae tksqwkrstm
421 etqiqappit htnsskdrer erhlqqtelq hrkavecime gpgghadgns splclsinns
481 vgerqrvkik yiscdednnp venspkql

Mojavensis **XP_001998315.1**

```
1 mseivdtell vnctilavrr felnsivntt llnslnrtev vsllsgiien rdnlgsinea
       61 kdflteclfp sptrpyelpw eqktiwaivf glmmfvaiag ngivlwivtg hrsmrtvtny
      121 fllnlsiadl lmsslncvfn fifmlnsdwp fgsiyctinn fvanvtvsts vftlvaisfd
      181 ryiaivhplk rrtsrrkvrf ilvliwalsc vlsapcllys simtkhyyng ksrtvcfmmw
      241 pdgryptsma dyvynltllv ltygipmivm lvcyslmgrv lwgsrsigen terqiesmks
      301 krkvvrmfia ivsifaicwl pyhmffiyay hnnqvastky vqhmylgfyw lamsnamvnp
      361 iiyywmnkrf rmyfqriifc ccmgltrykf dspksrlank nssnrntrve tksqwkrstm
      421 etqiqqvptt ssrerkaaga avnhmavecv ierpmddnss piclsinnsa gerqrlriky
      481 iscdednnpi eeasesssnh shghshnhsr shthahrcsh rqkttgtqel
```

Virilis [XP_002053610.1]

```
1 mseivdtell vnctilavrr felntivntt llntlnrtev vgllsgiien rdnldsinea
       61 kdflteclfp sptrpyelpw eqktiwaivf glmmfvaiag ngivlwivtg hrsmrtvtny
      121 fllnlsiadl lmstlncvfn fifmvnsdwp fgsiyctinn fvanvtvsts vftlvaisfd
      181 ryiaivhplk rrtsrrkvrf ilvliwalsc vlsapcllys simtkhyyng ksrtvcfmmw
      241 pdgryptsmt dyvynvtilv ltygipmivm licyslmgrv lwgsrsigen tdrqmesmks
      301 krkvvrmfia ivsifaicwl pyhlffiyay hnnqvastky vqhmylgfyw lamsnamvnp
      361 iiyywmnkrf rmyfqriifc cclglmryrf espksrmank nssnrhtrae tksqwkrstm
      421 etqiqqmpkt ssrdkdagvq glnhtaveci ierpiddnss piclsiknsa gerqrvkiky
      481 iscdednnpi eegsennssh dsnhshghgh scgrshgqna kaiqql
```

Grimshawi [XP_001994653.1]

```
   1 mpeivdtell vnctilavlr felnsivnit llnslnrtev vslltsiidn rdnlesinea
      61 kdflteclfp sptrpyelpw eqktiwaivf glmmfvaiag ngivlwivtg hrsmrtvtny
     121 fllnlsiadl lmsslncvfn fifmvnsdwp fgsiyctinn fvanvtvsts vftlvaisfd
     181 ryiaivhplk rrtsrrkvrf ilvmiwalsc vlsapcllys simtkhyyng ksrtvcfmmw
     241 pdgryptsma dyvynltilv ltygipmivm licytlmgrv lwgsrsigen tdrqmesmks
     301 krkvvrmfia vvsifaicwl pyhlffiyay hnnqvastky vqhmylgfyw lamsnamvnp
     361 iiyywmnkrf rmyfqrivcc cclgfiryrf dspksqatnk nsshrntrae tksqwkrstm
     421 etqiqqvpts ssrdrqagav qglnhtavec iierpvdesg spiclsinns agerqrvkik
     481 yiscdednnp iehesesghs hghthrhshr hslsnsygrs raqnaalvqq l
```
