# Supplementary material for: The incidence of candidate binding sites for β-arrestin in Drosophila neuropeptide GPCRs
Source: PLoS One. 2022 Nov 1;17(11):e0275410. doi: 10.1371/journal.pone.0275410 (PMC9624432; doi:10.1371/journal.pone.0275410)
Supplement: S13 Text — (PDF) [file pone.0275410.s017.pdf]

S13. Text **Multi-species analysis of CCHa_R2s**

Supporting Figure 16

Blastp search retrieved PA and PB isoforms from *D virilis*; however, not from several of the other species in the extended evolutionary analyses.

The lack of a PA in all species is significant because *D suzuki* included a BBS in the additional (PA-specific) sequence. Direct inspection of genomic DNA from each of the missing species (below) revealed them. Genbank references are included for each. First is shown the Clustal alignment of all 19 species

CLUSTAL Line-ups; Genbank Reference IDs below

Predicted TM domains in YELLOW

BBS sequences in RED

Here is the Clustal line up of PB isoforms:

```
Mojavensis           ----MYAALMDMSQMLAASLAYAPAESSSAAA--------AAGVNLSQLSNSSQLDG---       45
Virilis             MPKNMLAALMDMSQTLAASLAYAPLESNAAATAAA-AAAAAVVLNVSQLGNSSQLDG---        56
Grimshawi            ----MYAALMDMSQTLAASLAYAPMESNSAAAAAAAAAAAALGMNVSQLSNSSQLDG---       53
Bipectinate          -----MSALMDMSQTLALSLLYAPPDATGGVSPNIISIGSGN------GSDGGGNDSSLG       49
Anannassae           ----MSAALMDMSQTLALSLVYAPLDATGGASPNIA-IGNGNDSN--GGGDGGANDSGLV       53
Serrata              ----VDTDVMDIAQALVASLAHAPLDGSGG---------------------GGGNGN---       32
Kikkawei             ----VNTNKMDIAQALVASLAHAPLDGSGN---------------------G----S---       28
Fichsuphila          ----MHAALMDVGQTLAGGS------SAND---------------------TGLLATGGE       29
Rhopaloa             ----MFAALMDVGQTLAGSL------A-----------------------------E-GN       20
Elegans              ----MFAALMDVGQTLAGSL------A-----------------------------TEGN       21
Suzuki               ----MYAALVDVGQTLVAGLVDRA--GANE---------------------SGLLASGQA       33
Biarmipes            ----MYVALVDVGQTLAAGLADG----ANE---------------------SVLMATGHA       31
Takahashi            ----MYAALMDVGQTLAASLAEGGGN---E---------------------SGLLATQ--       30
Erecta               ----MYAALMDVGQTLAARLADGEGN---D---------------------SGLLATRQG       32
Melanogaster         ----MYASLMDVGQTLAARLADSDGNGAND---------------------SGLLATGQG       35
Sechellia            ----MYASLMDVGQTLAARLADGEGNGAND---------------------SGLLATGQG       35
Eugracilis           ----MYASLMDVGQTLAARLADGEGNGAND---------------------SGLLATGQG       35
Simulans             ----MYASLMDVGQTLAARLADGEGNGAND---------------------SGLLATGQG       35
Mauritania           ----MYASLMDVGQTLAARLADGEGNGAND---------------------SGLLATGQG       35
                              :*:.* *.


Mojavensis           -SSVVTVATAAT------VGQHN-A-SIEESSYVKVLDRPETYIVTVLYTLIFIVGVLGN       96
Virilis             -SLATAAATTTTTAVTTSTSTHNAS-GEEYPQYVKVLDRPETYIVTVLYTLIFIVGVLGN        114
Grimshawi            -STGATAAT---------SIPHNVS-AEEYPQYVKVLDRPETYIVTVLYTLIFIVGVLGN       102
Bipectinate          LATG-QQGAATAVGGVLGLGQHNASADGGGGPYVPVLERPETYIVTVLYTLIFIVGVLGN       108
Anannassae           LATG-QQGVATVVGGVLGLGQHNGSADGGIGPYVPVLERPETYIVTVLYTLIFIVGVLGN       112
Serrata              -GSGANDSVLLATGTGAAAEQHNASIDGGMVPYVPVLDRPETYIVTVLYTLIFIVGVLGN       91
Kikkawei             -GSGGNDSVLLAT--GASAEQHNASIDGGMVPYVPVLDRPETYIVTVLYTLIFIVGVLGN       85
Fichsuphila          QEQEQGPGLGLGMGMGMEMGQHNASADGGMVPYVPVLDRPETYIVTVLYTLIFIVGVLGN       89
Rhopaloa             GSGVQGEGLELGPGMGVEVGQHNASADGGIVPYVPVLDRPETYIVTVLYTLIFIVGVLGN       80
Elegans              DSGLLATG-QYEGGVEVGMGQHNSSADGGMVPYVPVLDRPETYIVTVLYTLIFIVGVLGN       80
Suzuki               AEQEQG----------HG-VAHNASADDGMVPYVPVLDRPETYIVTVLYTLIFIVGVLGN       82
Biarmipes            AEQEQG----------HW-VGQNASGDGVMVPYVPVLDRPETYIVTVLYTLIFIVGVLGN       80
Takahashi            -------EGQ---GLDLE-TGHNASADGGIVPYVPVLDRPETYIVTVLYTLIFIVGVLGN       79
Erecta               LEQEQEQEQE---GLALG-MAHNASADGGMVPYVPVLDRPETYIVTVLYTLIFVVGVLGN       88
Melanogaster         LEQ----EQE---GLALD-MGHNASADGGIVPYVPVLDRPETYIVTVLYTLIFIVGVLGN       87
Sechellia            LEQ----EQE---GLALD-MGHNASADGGIVPYVPVLDRPETYIVTVLYTLIFIVGVLGN       87
Eugracilis           LEQ----EQE---GLALD-MGHNASADGGIVPYVPVLDRPETYIVTVLYTLIFIVGVLGN       87
Simulans             LEQ----EQE---GLALD-MGHNASADGGIVPYVPVLDRPETYIVTVLYTLIFIVGVLGN       87
Mauritania           LEQ----EQE---GLALD-MGHNASADGGIVPYVPVLDRPETYIVTVLYTLIFIVGVLGN       87
                                          :* :       ** **:***************:******

Mojavensis           GTLVIIFFRHRSMRNIPNTYILSLALADLLVILVCVPVATIVYTQESWPFERNMCRISEF       156
Virilis             GTLVIIFFRHRSMRNIPNTYILSLALADLLVILVCVPVATIVYTQESWPFERNMCRITEF        174
Grimshawi            GTLVIIFFRHRSMRNIPNTYILSLALADLLVILVCVPVATIVYTQESWPFERNMCRISEF       162
```

```
Bipectinate        GTLVIIFFRHRSMRNIPNTYILSLALADLLVILVCVPVATIVYTQESWPFERNMCRISEF        168
Anannassae         GTLVIIFFRHRSMRNIPNTYILSLALADLLVILVCVPVATIVYTQESWPFERNMCRISEF        172
Serrata            GTLVIIFFRHRSMRNIPNTYILSLALADLLVILVCVPVATIVYTQESWPFERNMCRISEF        151
Kikkawei           GTLVIIFFRHRSMRNIPNTYILSLALADLLVILVCVPVATIVYTQESWPFERNMCRISEF        145
Fichsuphila        GTLVIIFFRHRSMRNIPNTYILSLALADLLVILVCVPVATIVYTQESWPFERNMCRISEF        149
Rhopaloa           GTLVIIFFRHRSMRNIPNTYILSLALADLLVILVCVPVATIVYTQESWPFERNMCRISEF        140
Elegans            GTLVIIFFRHRSMRNIPNTYILSLALADLLVILVCVPVATIVYTQESWPFERNMCRISEF        140
Suzuki             GTLVIIFFRHRSMRNIPNTYILSLALADLLVILVCVPVATIVYTQESWPFDRNMCRISEF        142
Biarmipes          GTLVIIFFRHRSMRNIPNTYILSLALADLLVILVCVPVATIVYTQESWPFERNMCRISEF        140
Takahashi          GTLVIIFFRHRSMRNIPNTYILSLALADLLVILVCVPVATIVYTQESWPFERNMCRISEF        139
Erecta             GTLVIIFFRHRSMRNIPNTYILSLALADLLVILVCVPVATIVYTQESWPFERNMCRISEF        148
Melanogaster       GTLVIIFFRHRSMRNIPNTYILSLALADLLVILVCVPVATIVYTQESWPFERNMCRISEF        147
Sechellia          GTLVIIFFRHRSMRNIPNTYILSLALADLLVILVCVPVATIVYTQESWPFERNMCRISEF        147
Eugracilis         GTLVIIFFRHRSMRNIPNTYILSLALADLLVILVCVPVATIVYTQESWPFERNMCRISEF        147
Simulans           GTLVIIFFRHRSMRNIPNTYILSLALADLLVILVCVPVATIVYTQESWPFERNMCRISEF        147
Mauritania         GTLVIIFFRHRSMRNIPNTYILSLALADLLVILVCVPVATIVYTQESWPFERNMCRISEF        147
                   **************************************************:******:**

Mojavensis         FKDISIGVSVFTLTALSGERYCAIVNPLRKLQTKPLTVFTAVIIWVFAIMLGMPSFVVSD        216
Virilis           FKDISIGVSVFTLTALSGERYCAIVNPLRKLQTKPLTVFTAVIIWVFAIMLGMPSFVVSD         234
Grimshawi          FKDISIGVSVFTLTALSGERYCAIVNPLRKLQTKPLTVFTAVIIWVLAIMLGMPSFVVSD        222
Bipectinate        FKDISIGVSVFTLTALSGERYCAIVNPLRKLQTKPLTVFTAAMIWVLAILLGMPSFLVSN        228
Anannassae         FKDISIGVSVFTLTALSGERYCAIVNPLRKLQTKPLTVFTAAMIWVLAILLGMPSFLFSN        232
Serrata            FKDISIGVSVFTLTALSGERYCAIVNPLRKLQTKPLTVFTAVMIWILAILLGMPSFLVSD        211
Kikkawei           FKDISIGVSVFTLTALSGERYCAIVNPLRKLQTKPLTVFTAVMIWILAILLGMPSFLVSD        205
Fichsuphila        FKDISIGVSVFTLTALSGERYCAIVNPLRKLQTKPLTVFTAVIIWILAILLGMPSFLVSD        209
Rhopaloa           FKDISIGVSVFTLTALSGERYCAIVNPLRKLQTKPLTVFTAVIIWILAILLGMPSFLVSD        200
Elegans            FKDISIGVSVFTLTALSGERYCAIVNPLRKLQTKPLTVFTAVIIWILAILLGMPSFLVSD        200
Suzuki             FKDISIGVSVFTLTALSGERYCAIVNPLRKLQTKPLTVFTAVIIWILAILLGMPSVLFSD        202
Biarmipes          FKDISIGVSVFTLTALSGERYCAIVNPLRKLQTKPLTVFTAAIIWILAILLGMPSVLFSD        200
Takahashi          FKDISIGVSVFTLTALSGERYCAIVNPLRKLQTKPLTVFTAVIIWILAILLGMPSVLFSD        199
Erecta             FKDISIGVSVFTLTALSGERYCAIVNPLRKLQTKPLTVFTAVMIWILAILLGMPSVLFSD        208
Melanogaster       FKDISIGVSVFTLTALSGERYCAIVNPLRKLQTKPLTVFTAVMIWILAILLGMPSVLFSD        207
Sechellia          FKDISIGVSVFTLTALSGERYCAIVNPLRKLQTKPLTVFTAVMIWILAILLGMPSVLFSD        207
Eugracilis         FKDISIGVSVFTLTALSGERYCAIVNPLRKLQTKPLTVFTAVMIWILAILLGMPSVLFSD        207
Simulans           FKDISIGVSVFTLTALSGERYCAIVNPLRKLQTKPLTVFTAVMIWILAILLGMPSVLFSD        207
Mauritania         FKDISIGVSVFTLTALSGERYCAIVNPLRKLQTKPLTVFTAVMIWILAILLGMPSVLFSD        207
                   *****************************************.:**::**:*****.:.*:

Mojavensis         IQSYNITTPNGNITIGVCSPFRSKIYAKYMVVAKASIYYLVPLSIIGVLYIMMAKRLHIS        276
Virilis           IQGYTLPTPNGNITIEVCSPFRSKIYAKYMVVAKASIYYLVPLSIIGVLYIMMAKRLHIS         294
Grimshawi          LQGYTLPTNKGNITIEVCSPFRSKIYAKYMVVAKASIYYFVPLSIIGVLYIMMAKRLHIS        282
Bipectinate        IKSYTVLTPNGNMSIEVCDPFRDPEYAKYMVAAKASIYYLLPLSIIGALYIMMAKRLHIS        288
Anannassae         IKSYTVLTPNGNMSIEVCDPYRDPEYAKYMVAAKASIYYLVPLSIIGALYIMMAKRLHIS        292
Serrata            IKSYPVLTATGNMTIEVCSPFRDPEYAQYMVAAKAFIYYLLPLSIIGALYIMMAKRLHIS        271
Kikkawei           IKSYPVLTANGNMTIEVCSPYRDPEYAQCMVAAKAFIYYLLPLSIIGALYIMMAKRLHIS        265
Fichsuphila        IKSYPVLTAKGNMTIEVCSPFRDPEYAKCMVAAKAFIYYLLPLSIIGALYIMMAKRLHMS        269
Rhopaloa           IKSYPVVTVKGNITIEVCSPYSDPEYAKYMVAAKATIYYLLPLSIIGALYIMMAKRLHMS        260
Elegans            IKSYPVYTANGNMTIEVCSPFRDPEYAKCMVAAKAFIYYLLPLSIIGALYIMMAKRLHMS        260
Suzuki             IKSYPVFTAMGNITIEVCSPFRDPEYAKFMVAAKALIYYLLPLSIIGALYIMMANRLHMS        262
Biarmipes          IKSYPVLTAMGNMTIEVCSPFRDAEYAKFMVAAKALIYYLLPLSIIGALYIMMAKRLHMS        260
Takahashi          IKSYPVATPMGNITIEVCSPFRDREYAKFMVAAKALIYYLLPLSIIGALYIMMAKRLHMS        259
Erecta             IKSYPVFTATGNMTIEVCSPFRDPEYAKFMVAGKALVYYLLPLSIIGALYIMMAKRLHMS        268
Melanogaster       IKSYPVFTATGNMTIEVCSPFRDPEYAKFMVAGKALVYYLLPLSIIGALYIMMAKRLHMS        267
Sechellia          IKSYPVFTATGNMTIEVCSPFRDPEYAKFMVAGKALVYYLLPLSIIGALYIMMAKRLHMS        267
Eugracilis         IKSYPVFTATGNMTIEVCSPFRDPEYAKFMVAGKALVYYLLPLSIIGALYIMMAKRLHMS        267
Simulans           IKSYPVFTATGNMTIEVCSPFRDPEYAKFMVAGKALVYYLLPLSIIGALYIMMAKRLHMS        267
Mauritania         IKSYPVFTATGNMTIEVCSPFRDPEYAKFMVAGKALVYYLLPLSIIGALYIMMAKRLHMS        267
                   ::.* : *  **::* **.*: .  **: **..** :**::******.******:***:*

Mojavensis         ARDMPGEQLSIQSRSQARARRHVARMVVAFVVVFFICFFPYHVFELWYHFYPTAEDDFDD        336
Virilis           ARDMPGEQLSIQSRSQARARRHVARMVVAFVVVFFICFFPYHVFELWYHFYPTAEEDFDD         354
Grimshawi          ARDMPGEQLSIQSRSQARARRHVARMVVAFVVVFFICFFPYHVFELWYHFYPTAEDDFDD        342
Bipectinate        ARDMPGEQQSMQSRSQARARRHVARMVVAFVVVFFICFFPYHVFELWYHFYPTAEEDFDE        348
Anannassae         ARDMPGEQQSMQSRSQARARRYVARMVVAFVVVFFICLFPYYVFELWYHFYPTAEEDFDE        352
Serrata            ARDMPGEQQSMQSRTQARARRHVARMVVAFVVVFFICFFPYHVFELWYHFYPTAEEDFDD        331
Kikkawei           ARDMPGEQQSMQSRTQARARRHVARMVVAFVVVFFICFFPYHVFELWYHFYPTAEEDFDD        325
Fichsuphila        ARNMPGEQQSMQSRTQARARRHVARMVVAFVVVFFICFFPYHVFELWYHFYPTAEEDFDD        329
Rhopaloa           ARNMPGEQQSMQSRTQARARRHVARMVVAFVVVFFICFFPYHVFELWYHFYPTAEEDFDD        320
Elegans            ARNMPGEQQSMQSRTQARARRHVARMVVAFVVVFFICFFPYHVFELWYHFYPTAEEDFDD        320
Suzuki             ARNMPGEQQSMQSRTQARARRHVARMVVAFVVVFFICFFPYHVFELWYHFYPTAEEDFDD        322
```

```
Biarmipes          ARNMPGEQQSMQSRTQARARRHVARMVVAFVVVFFICFFPYHVFELWYHFYPTAEEDFDD        320
Takahashi          ARNMPGEQQSMQSRTQARARRHVARMVVAFVVVFFICFFPYHVFELWYHFYPTAEEDFDD        319
Erecta             ARNMPGEQQSMQSRTQARARLHVARMVVAFVVVFFICFFPYHVFELWYHFYPTAEEDFDE        328
Melanogaster       ARNMPGEQQSMQSRTQARARLHVARMVVAFVVVFFICFFPYHVFELWYHFYPTAEEDFDE        327
Sechellia          ARNMPGEQQSMQSRTQARARLHVARMVVAFVVVFFICFFPYHVFELWYHFYPTAEEDFDE        327
Eugracilis         ARNMPGEQQSMQSRTQARARLHVARMVVAFVVVFFICFFPYHVFELWYHFYPTAEEDFDE        327
Simulans           ARNMPGEQQSMQSRTQARARLHVARMVVAFVVVFFICFFPYHVFELWYHFYPTAEEDFDE        327
Mauritania         ARNMPGEQQSMQSRTQARARLHVARMVVAFVVVFFICFFPYHVFELWYHFYPTAEEDFDE        327
                   **:***** *:***:***** :***************:***:*************:***:

Mojavensis         FWNVVRIVGFCTSFLNSCVNPVALYCVSGVFRQHFNRYLCCICVKRQPHLRQHSTATGIM        396
Virilis           FWHVVRIVGFCTSFLNSCVNPVALYCVSGVFRQHFNRYLCCICVKRQPHLRQHSTATGVM         414
Grimshawi          FWHVVRIVGFCTSFLNSCVNPVALYCVSGVFRQHFNRYLCCFCVKRQPHLRQHSTATGVM        402
Bipectinate        FWNVLRIVGFCTSFLNSCVNPVALYCVSGVFRQHFNRYLCCFCVKRQPHLRQHSTATGIM        408
Anannassae         FWNVLRIVGFCTSFLNSCVNPVALYCVSGVFRQHFNRYLCCFCVKRQPHLRQHSTATGIM        412
Serrata            FWNVLRIVGFCTSFLNSCVNPVALYCVSGVFRQHFNRYLCCICVKRQPHLRQHSTATGMM        391
Kikkawei           FWNVLRIVGFCTSFLNSCVNPVALYCVSGVFRQHFNRYLCCICVKRQPHLRQHSTATGMM        385
Fichsuphila        FWNVLRIVGFCTSFLNSCVNPVALYCVSGVFRQHFNRYLCCICVKRQPHLRQHSTATGMM        389
Rhopaloa           FWNVLRIVGFCTSFLNSCVNPVALYCVSGVFRQHFNRYLCCICVKRQPHLRQHSTATGMM        380
Elegans            FWNVLRIVGFCTSFLNSCVNPVALYCVSGVFRQHFNRYLCCICVKRQPHLRQHSTATGMM        380
Suzuki             FWNVLRIVGFCTSFLNSCVNPVALYCVSGVFRLHFNRYLCCVCVKRHPHLRQHSTG---M        379
Biarmipes          FWNVLRIVGFCTSFLNSCVNPVALYCVSGVFRQHFNRYLCCFCVKRQPHVRQHSTATGMM        380
Takahashi          FWNVLRIVGFCTSFLNSCVNPVALYCVSGVFRQHFNRYLCCICVKRQPHLRQHSTATGMM        379
Erecta             FWNVLRIVGFCTSFLNSCVNPVALYCVSGVFRQHFNRYLCCICVKRQPHLRQHSTATGMM        388
Melanogaster       FWNVLRIVGFCTSFLNSCVNPVALYCVSGVFRQHFNRYLCCICVKRQPHLRQHSTATGMM        387
Sechellia          FWNVLRIVGFCTSFLNSCVNPVALYCVSGVFRQHFNRYLCCICVKRQPHLRQHSTATGMM        387
Eugracilis         FWNVLRIVGFCTSFLNSCVNPVALYCVSGVFRQHFNRYLCCICVKRQPHLRQHSTATGMM        387
Simulans           FWNVLRIVGFCTSFLNSCVNPVALYCVSGVFRQHFNRYLCCICVKRQPHLRQHSTATGMM        387
Mauritania         FWNVLRIVGFCTSFLNSCVNPVALYCVSGVFRQHFNRYLCCICVKRQPHLRQHSTATGMM        387
                   **:*:*************************** ********.****:**:*****.   *

Mojavensis         D-TSVTSMRRSTYVGGGIAAG--GAREGGPRASVHMNNNNHGVS--------GAAGGRGGS       445
Virilis           D-TSVTSMRRSTYVGGGGG-GAVGGSLAAHRASLHMNNNNHGVAVGGG----GGGGGRGGS        468
Grimshawi          D-TSVTSMRRSTYVGGGGGVGGATGSLAAHRASLHMNNNNHGSG---A----GGPGGRTGS       454
Bipectinate        DNTSVMSMRRSTYVGGAG---------GNLRASMHRNSNHGGGGG-----SGLSAGRGAS       454
Anannassae         DNTSVMSMRRSTYVGGAG---------GNLRSSMHRNSNHGGGGG-----AGLPAGRGAS       458
Serrata            DNTSVMSMRRSTYVGGCGTG-------GNLRASLHRNSNQG--GGG---GLGGGAGRGGS        439
Kikkawei           DNTSVMSMRRSTYVGGCGAG-------GNLRASLHRNSNQGVGGGG---ALGGGTGRVGS        435
Fichsuphila        DNTSVMSMRRSTYVGV-GG--------GNLRASLHRNSNHGVGA------AGGGPGRXGS        434
Rhopaloa           DNTSVMSMRRSTYVGGATA--------GHLRASLHRNSNHGGGGGGVGGAGGLGSGRVGS        432
Elegans            DNTSVMSMRRSTYVGGAAA--------GQLRASLHRNSSNHGGG------VGFGSGRVGS        426
Suzuki             DNTTVMSMRRSTYIGSAGGG------VANLRASQHRDSCHGVG-------GGGGTGHADS        426
Biarmipes          DNTSVMSMRRSTYVGGAGGV----GA--NLRASQHRN---------------------SS        413
Takahashi          DNTSVMSMRRSTYVGGAVGC----GPAGNLRASQHRNSNHG---------GGGGSGRVGS        426
Erecta             DNTSVMSMRRSTYVGGT---------AGNLRASLHRSSNHGVGGAAGGG-GGGGSGRVGS        438
Melanogaster       DNTSVMSMRRSTYVGGT---------AGNLRASLHRNSNHGVGGAGGGVGGGVGSGRVGS        438
Sechellia          DNTSVMSMRRSTYVGGT---------AGNLRASLHRNSNHGVAGAGGGVGGG--SGRVGS        436
Eugracilis         DNTSVMSMRRSTYVGGT---------AGNLRASLHRNSNHGVAGAGGGVGGG--SGRVGS        436
Simulans           DNTSVMSMRRSTYVGGT---------AGNLRASLHRNSNHGVGGAGGGVGGG--SGRVGS        436
Mauritania         DNTSVMSMRRSTYVGGT---------AGNLRASLHRNSNHGVGGAGGGVGGG--SGRVGS        436
                   * *:* *******:*               *:* * .                       *

Mojavensis         FHRQDSMALQHAGSVNGHSH-----NANVGAGAGIGRASIINEKR             485
Virilis           FHRQDSMPLQHAGSGNGHAH-----NV-GGPGAGIGRASIINEKS              518
Grimshawi          FHRQDSMPLQHAGSGNGHAH-----NV-GG----IGRASIINEKS             500
Bipectinate        FHGGDSMPMQHSNGHGAAGGTLGGAGVGATGGPSSGRAAAVGEKR             499
Anannassae         FHGGDSMPLQHSNGHGATGGNSGGAGASATGGPSLGRTAAVGEKR             503
Serrata            FHRQDSMPLQHGNGHGAA-GGGAA--SGPSCGPAGR-AVAVGEKR             479
Kikkawei           FHRQDSMPLQHGNGHGAAAGGGTA--SGPSCGSAGR-AVAVGEKR             476
Fichsuphila        FHRQDSMPLQHGNGHGAVASAAG-----C--GSGGR-AAAGGEKR             471
Rhopaloa           FHRQDSMPLQHGNGHGSVAGGAA-------SGSGGR-ATAVGEKR             481
Elegans            FHRQDSMALQHGNGHGAATGGAS-----CAPGSGGR-GPAGGEKR             465
Suzuki             LHRQESMPLQHGNGCVSG----------------GR-AASVGEKS             466
Biarmipes          LHRQASTPLQHGNGHGTGGDETGH---GPGWVSGGRAAA-VGEKR             454
Takahashi          FHRQDSMPLQHGNGHGTGIGAGVG---GAACGSGGRASAAVGEKR             468
Erecta             FHRQDSMPLQHGNAHGAGAGGGSC---GL--GSGGRTAP-VCEKS             489
Melanogaster       FHRQDSMPLQHGNAHGGGAGGGSS---GL--GAGGRTAA-VSEKS             489
Sechellia          FYRQDSMPLQHGNAHGTGAGVGSS---GL--GAGGRTAS-VSEKR             475
Eugracilis         FYRQDSMPLQHGNAHGTGAGVGSS---GL--GAGGRTAS-VSEKR             475
Simulans           FHRQDSMPLQHGNAHGAGAGGGSS---GL--GAGGRTAS-VSEKS             487
Mauritania         FHRQDSMPLQHGNAHGAGAGGGSS---GL--GAGGRTAP-VSEKS             487
```

The above are all PB isoforms; which are listed individual below.
Here are the three isoforms predicted from D melanogaster:
PA (ALTERNATIVE SPLICE AT FINAL AA)

```
MYASLMDVGQ TLAARLADSD GNGANDSGLL ATGQGLEQEQ EGLALDMGHN ASADGGIVPY
VPVLDRPETY IVTVLYTLIF IVGVLGNGTL VIIFFRHRSM RNIPNTYILS LALADLLVIL
VCVPVATIVY TQESWPFERN MCRISEFFKD ISIGVSVFTL TALSGERYCA IVNPLRKLQT
KPLTVFTAVM IWILAILLGM PSVLFSDIKS YPVFTATGNM TIEVCSPFRD PEYAKFMVAG
KALVYYLLPL SIIGALYIMM AKRLHMSARN MPGEQQSMQS RTQARARLHV ARMVVAFVVV
FFICFFPYHV FELWYHFYPT AEEDFDEFWN VLRIVGFCTS FLNSCVNPVA LYCVSGVFRQ
HFNRYLCCIC VKRQPHLRQH STATGMMDNT SVMSMRRSTY VGGTAGNLRA SLHRNSNHGV
GGAGGGVGGG VGSGRVGSFH RQDSMPLQHG NAHGGGAGGG SSGLGAGGRT AAVSEKSFIN
RYESGVMRY
```

PB (SHORTEST)

```
MYASLMDVGQ TLAARLADSD GNGANDSGLL ATGQGLEQEQ EGLALDMGHN ASADGGIVPY
VPVLDRPETY IVTVLYTLIF IVGVLGNGTL VIIFFRHRSM RNIPNTYILS LALADLLVIL
VCVPVATIVY TQESWPFERN MCRISEFFKD ISIGVSVFTL TALSGERYCA IVNPLRKLQT
KPLTVFTAVM IWILAILLGM PSVLFSDIKS YPVFTATGNM TIEVCSPFRD PEYAKFMVAG
KALVYYLLPL SIIGALYIMM AKRLHMSARN MPGEQQSMQS RTQARARLHV ARMVVAFVVV
FFICFFPYHV FELWYHFYPT AEEDFDEFWN VLRIVGFCTS FLNSCVNPVA LYCVSGVFRQ
HFNRYLCCIC VKRQPHLRQH STATGMMDNT SVMSMRRSTY VGGTAGNLRA SLHRNSNHGV
GGAGGGVGGG VGSGRVGSFH RQDSMPLQHG NAHGGGAGGG SSGLGAGGRT AAVSEKR
```

PC Stop suppressed PB

```
1   MYASLMDVGQ TLAARLADSD GNGANDSGLL ATGQGLEQEQ EGLALDMGHN ASADGGIVPY
 61 VPVLDRPETY IVTVLYTLIF IVGVLGNGTL VIIFFRHRSM RNIPNTYILS LALADLLVIL
121 VCVPVATIVY TQESWPFERN MCRISEFFKD ISIGVSVFTL TALSGERYCA IVNPLRKLQT
181 KPLTVFTAVM IWILAILLGM PSVLFSDIKS YPVFTATGNM TIEVCSPFRD PEYAKFMVAG
241 KALVYYLLPL SIIGALYIMM AKRLHMSARN MPGEQQSMQS RTQARARLHV ARMVVAFVVV
301 FFICFFPYHV FELWYHFYPT AEEDFDEFWN VLRIVGFCTS FLNSCVNPVA LYCVSGVFRQ
361 HFNRYLCCIC VKRQPHLRQH STATGMMDNT SVMSMRRSTY VGGTAGNLRA SLHRNSNHGV
421 GGAGGGVGGG VGSGRVGSFH RQDSMPLQHG NAHGGGAGGG SSGLGAGGRT AAVSEKRXGT
481 LIVELLGEEE VVVSLQADEQ L
```

Some but not all PA isoforms were recovered in the original blastp search using melanogaster PA, and the Suzuki PA form contained a BBS in the added 12 AA. So I searched for the missing PA isoforms (sechelia, biarmipes, anannassae, mojavensis, serrata, kikkawae, ficusphelia, bipectinate, Takahashi, eugracilis). Found all - the documentation and data analysis are at the bottom of this file in section called:

"CCHa2-R PA isoform – data capture"

Here is the 1 conserved BBS in biarmipes and Suzuki PA proteins:

```
Mojavensis         FHRQDSMALQHAGSVNGHSH-----NANVGAGAGIGRASIINEKR----YDER-TRY  492
Virilis           FHRQDSMPLQHAGSGNGHAH-----NV-GGPGAGIGRASIINEKSLIKRYDER-TRY  529
Grimshawi          FHRQDSMPLQHAGSGNGHAH-----NV-GG----IGRASIINEKSLIKRYEER-ARY  511
Bipectinate        FHGGDSMPMQHSNGHGAAGGTLGGAGVGATGGPSSGRAAAVGEKSINRFESNR-LRY  510
Anannassae         FHGGDSMPLQHSNGHGATGGNSGGAGASATGGPSLGRTAAVGEKRINRFESNR-MRY  514
Serrata            FHRQDSMPLQHGNGHGAA-GGGAA--SGPSCGPAGR-AVAVGEKSLMGRYVSDRLRY 491
Kikkawei           FHRQDSMPLQHGNGHGAAAGGGTA--SGPSCGSAGR-AVAVGEKSLMGRYVSDRLRY 482
Fichsuphila        FHRQDSMPLQHGNGHGAVASAAG-----C--GSGGR-AAAGGEKSIINHYENDRLRY  482
Rhopaloa           FHRQDSMPLQHGNGHGSVAGGAA-------SGSGGR-ATAVGEKSLINRYESDRIRY  481
Elegans            FHRQDSMALQHGNGHGAATGGAS-----CAPGSGGR-GPAGGEKSLINRYESERIRY  477
Suzuki             LHRQESMPLQHGNGCVSG----------------GR-AASVGEKSLLSRYENDRIRY  478
Biarmipes          LHRQASTPLQHGNGHGTGGDETGH---GPGWVSGGRAAA-VGEKSFISRYENDRIRF 466
Takahashi          FHRQDSMPLQHGNGHGTGIGAGVG---GAACGSGGRASAAVGEKSFINRYESGVMRY 470
Erecta             FHRQDSMPLQHGNAHGAGAGGGSC---GL--GSGGRTAP-VCEKSFINRYESGRMRY  501
Melanogaster       FHRQDSMPLQHGNAHGGGAGGGSS---GL--GAGGRTAA-VSEKSFINRYESGVMRY  501
Sechellia          FYRQDSMPLQHGNAHGTGAGVGSS---GL--GAGGRTAS-VSEKSFINRYESGVMRY 489
Eugracilis         FYRQDSMPLQHGNAHGTGAGVGSS---GL--GAGGRTAS-VSEKSLINKR--DRIRY  485
Simulans           FHRQDSMPLQHGNAHGAGAGGGSS---GL--GAGGRTAS-VSEKSFINRYESGVMRY  499
Mauritania         FHRQDSMPLQHGNAHGAGAGGGSS---GL--GAGGRTAP-VSEKSFINRYESGVMRY  499
```

Melanogaster **NP_610199.2**

```
    1 myaslmdvgq tlaarladsd gngandsgll atgqgleqeq eglaldmghn asadggivpy
   61 vpvldrpety ivtvlytlif ivgvlgngtl viiffrhrsm rnipntyils laladllvil
  121 vcvpvativy tqeswpfern mcriseffkd isigvsvftl talsgeryca ivnplrklqt
  181 kpltvftavm iwilaillgm psvlfsdiks ypvftatgnm tievcspfrd peyakfmvag
  241 kalvyyllpl siigalyimm akrlhmsarn mpgeqqsmqs rtqararlhv armvvafvvv
  301 fficffpyhv felwyhfypt aeedfdefwn vlrivgfcts flnscvnpva lycvsgvfrq
  361 hfnrylccic vkrqphlrqh statgmmdnt svmsmrrsty vggtagnlra slhrnsnhgv
  421 ggagggvggg vgsgrvgsfh rqdsmplqhg nahgggaggg ssglgaggrt aavseksfin
  481 ryesgvmry
```

Simulans **XP_016026076.2**

```
1 myaslmdvgq tlaarladge gngandsgll atgqgleqeq eglaldmghn asadggivpy
   61 vpvldrpety ivtvlytlif ivgvlgngtl viiffrhrsm rnipntyils laladllvil
  121 vcvpvativy tqeswpfern mcriseffkd isigvsvftl talsgeryca ivnplrklqt
  181 kpltvftavm iwilaillgm psvlfsdiks ypvftatgnm tievcspfrd peyakfmvag
  241 kalvyyllpl siigalyimm akrlhmsarn mpgeqqsmqs rtqararlhv armvvafvvv
  301 fficffpyhv felwyhfypt aeedfdefwn vlrivgfcts flnscvnpva lycvsgvfrq
  361 hfnrylccic vkrqphlrqh statgmmdnt svmsmrrsty vggtagnlra slhrnsnhgv
  421 ggagggvggg sgrvgsfhrq dsmplqhgna hgagagggss glgaggrtas vseksfinry
  481 esgvmry
```

Suzuki **XP_036669887.1**

```
1 myaalvdvgq tlvaglvdra ganesgllas gqaaeqeqgh gvahnasadd gmvpyvpvld
   61 rpetyivtvl ytlifivgvl gngtlviiff rhrsmrnipn tyilslalad llvilvcvpv
  121 ativytqesw pfdrnmcris effkdisigv svftltalsg erycaivnpl rklqtkpltv
  181 ftaviiwila illgmpsvlf sdiksypvft amgnitievc spfrdpeyak fmvaakaliy
  241 yllplsiiga lyimmanrlh msarnmpgeq qsmqsrtqar arrhvarmvv afvvvfficf
  301 fpyhvfelwy hfyptaeedf ddfwnvlriv gfctsflnsc vnpvalycvs gvfrlhfnry
  361 lccvcvkrhp hlrqhstgmd nttvmsmrrs tyigsagggv anlrasqhrd schgvggggg
  421 tghadslhrq esmplqhgng cvsggraasv geksllsrye ndriry
```

Mauritania **XP_033155286.1**

```
1 myaslmdvgq tlaarladge gngandsgll atgqgleqeq eglaldmghn asadggivpy
   61 vpvldrpety ivtvlytlif ivgvlgngtl viiffrhrsm rnipntyils laladllvil
  121 vcvpvativy tqeswpfern mcriseffkd isigvsvftl talsgeryca ivnplrklqt
  181 kpltvftavm iwilaillgm psvlfsdiks ypvftatgnm tievcspfrd peyakfmvag
  241 kalvyyllpl siigalyimm akrlhmsarn mpgeqqsmqs rtqararlhv armvvafvvv
  301 fficffpyhv felwyhfypt aeedfdefwn vlrivgfcts flnscvnpva lycvsgvfrq
  361 hfnrylccic vkrqphlrqh statgmmdnt svmsmrrsty vggtagnlra slhrnsnhgv
  421 ggagggvggg sgrvgsfhrq dsmplqhgna hgagagggss glgaggrtap vseksfinry
  481 esgvmry
```

Sechellia **XP_002043393.1**

```
1 myaslmdvgq tlaarladge gngandsgll atgqgleqeq eglaldmghn asadggivpy
   61 vpvldrpety ivtvlytlif ivgvlgngtl viiffrhrsm rnipntyils laladllvil
  121 vcvpvativy tqeswpfern mcriseffkd isigvsvftl talsgeryca ivnplrklqt
  181 kpltvftavm iwilaillgm psvlfsdiks ypvftatgnm tievcspfrd peyakfmvag
  241 kalvyyllpl siigalyimm akrlhmsarn mpgeqqsmqs rtqararlhv armvvafvvv
  301 fficffpyhv felwyhfypt aeedfdefwn vlrivgfcts flnscvnpva lycvsgvfrq
  361 hfnrylccic vkrqphlrqh statgmmdnt svmsmrrsty vggtagnlra slhrnsnhgv
  421 agagggvggg sgrvgsfyrq dsmplqhgna hgtgagvgss glgaggrtas vsekr
```

Serrata **KAH8390260.1**

```
1 vdtdvmdiaq alvaslahap ldgsggggn gngsgandsv llatgtgaaa eqhnasidgg
   61 mvpyvpvldr petyivtvly tlifivgvlg ngtlviiffr hrsmrnipnt yilslaladl
  121 lvilvcvpva tivytqeswp fernmcrise ffkdisigvs vftltalsge rycaivnplr
  181 klqtkpltvf tavmiwilai llgmpsflvs diksypvlta tgnmtievcs pfrdpeyaqy
  241 mvaakafiyy llplsiigal yimmakrlhi sardmpgeqq smqsrtqara rrhvarmvva
  301 fvvvfficff pyhvfelwyh fyptaeedfd dfwnvlrivg fctsflnscv npvalycvsg
  361 vfrqhfnryl ccicvkrqph lrqhstatgm mdntsvmsmr rstyvggcgt ggnlraslhr
  421 nsnqgggggl gggagrggsf hrqdsmplqh gnghgaaggg aasgpscgpa gravavgek
```

Erecta **XP_001970784.2**

1 myaalmdvgq tlaarladge gndsgllatr qgleqeqeqe qeglalgmah nasadggmvp
61 yvpvldrpet yivtvlytli fvvgvlgngt lviiffrhrs mrnipntyil slaladllvi
121 lvcvpvativ ytqeswpfer nmcriseffk disigvsvft ltalsgeryc aivnplrklq
181 tkpltvftav miwilaillg mpsvlfsdik sypvftatgn mtievcspfr dpeyakfmva
241 gkalvyyllp lsiigalyim makrlhmsar nmpgeqqsmq srtqararlh varmvvafvv
301 vfficffpyh vfelwyhfyp taeedfdefw nvlrivgfct sflnscvnpv alycvsgvfr
361 qhfnrylcci cvkrqphlrq hstatgmmdn tsvmsmrrst yvggtagnlr aslhrssnhg
421 vggaaggggg ggsgrvgsfh rqdsmplqhg nahgagaggg scglgsggrt apvceksfin
481 ryesgrmry

Takahashi XP_017003388.2

1 myaalmdvgq tlaaslaegg gnesgllatq egqgldletg hnasadggiv pyvpvldrpe
61 tyivtvlytl ifivgvlgng tlviiffrhr smrnipntyi lslaladllv ilvcvpvati
121 vytqeswpfe rnmcriseff kdisigvsvf tltalsgery caivnplrkl qtkpltvfta
181 viiwilailll gmpsvlfsdi ksypvatpmg nitievcspf rdreyakfmv aakaliyyll
241 plsiigalyi mmakrlhmsa rnmpgeqqsm qsrtqararr hvarmvvafv vvfficffpy
301 hvfelwyhfy ptaeedfddf wnvlrivgfc tsflnscvnp valycvsgvf rqhfnrylcc
361 icvkrqphlr qhstatgmmd ntsvmsmrrs tyvggavgcg pagnlrasqh rnsnhggggg
421 sgrvgsfhrq dsmplqhgng hgtgigagvg gaacgsggra saavgekr

Biarmipes XP_016966623.1

1 myvalvdvgq tlaagladga nesvlmatgh aaeqeqghwv gqnasgdgvm vpyvpvldrp
61 etyivtvlyt lifivgvlgn gtlviiffrh rsmrnipnty ilslaladll vilvcvpvat
121 ivytqeswpf ernmcrisef fkdisigvsv ftltalsger ycaivnplrk lqtkpltvft
181 aaiiwilail lgmpsvlfsd iksypvltam gnmtievcsp frdaeyakfm vaakaliyyl
241 lplsiigaly immakrlhms arnmpgeqqs mqsrtqarar rhvarmvvaf vvvfficffp
301 yhvfelwyhf yptaeedfdd fwnvlrivgf ctsflnscvn pvalycvsgv frqhfnrylc
361 cfcvkrqphv rqhstatgmm dntsvmsmrr styvggaggv ganlrasqhr nsslhrqast
421 plqhgnghgt ggdetghgpg wvsggraaav gekr

Eugracilis XP_017074910.2

1 myaslmdvgq tlaarladge gngandsgll atgqgleqeq eglaldmghn asadggivpy
61 vpvldrpety ivtvlytlif ivgvlgngtl viiffrhrsm rnipntyils laladllvil
121 vcvpvativy tqeswpfern mcriseffkd isigvsvftl talsgeryca ivnplrklqt
181 kpltvftavm iwilaillgm psvlfsdiks ypvftatgnm tievcspfrd peyakfmvag
241 kalvyyllpl siigalyimm akrlhmsarn mpgeqqsmqs rtqararlhv armvvafvvv
301 fficffpyhv felwyhfypt aeedfdefwn vlrivgfcts flnscvnpva lycvsgvfrq
361 hfnrylccic vkrqphlrqh statgmmdnt svmsmrrsty vggtagnlra slhrnsnhgv
421 agagggvggg sgrvgsfyrq dsmplqhgna hgtgagvgss glgaggrtas vsekr

Rhopaloa XP_044315908.1

1 mfaalmdvgq tlagslaegn gsgvqgegle lgpgmgvevg qhnasadggi vpyvpvldrp
61 etyivtvlyt lifivgvlgn gtlviiffrh rsmrnipnty ilslaladll vilvcvpvat
121 ivytqeswpf ernmcrisef fkdisigvsv ftltalsger ycaivnplrk lqtkpltvft
181 aviiwilail lgmpsflvsd iksypvvtvk gnitievcsp ysdpeyakym vaakatiyyl
241 lplsiigaly immakrlhms arnmpgeqqs mqsrtqarar rhvarmvvaf vvvfficffp
301 yhvfelwyhf yptaeedfdd fwnvlrivgf ctsflnscvn pvalycvsgv frqhfnrylc
361 cicvkrqphl rqhstatgmm dntsvmsmrr styvggatag hlraslhrns nhggggggvg
421 gagglgsgrv gsfhrqdsmp lqhgnghgsv aggaasgsgg ratavgeksl inryesdrir
481 y

Fichsuphila XP_017051484.2

1 mhaalmdvgq tlaggssand tgllatggeq eqeqgpglgl gmgmgmemgq hnasadggmv
61 pyvpvldrpe tyivtvlytl ifivgvlgng tlviiffrhr smrnipntyi lslaladllv
121 ilvcvpvati vytqeswpfe rnmcriseff kdisigvsvf tltalsgery caivnplrkl
181 qtkpltvfta viiwilailll gmpsflvsdi ksypvltakg nmtievcspf rdpeyakcmv
241 aakafiyyll plsiigalyi mmakrlhmsa rnmpgeqqsm qsrtqararr hvarmvvafv
301 vvfficffpy hvfelwyhfy ptaeedfddf wnvlrivgfc tsflnscvnp valycvsgvf
361 rqhfnrylcc icvkrqphlr qhstatgmmd ntsvmsmrrs tyvgvgggnl raslhrnsnh
421 gvgaaggpg rxgsfhrqds mplqhgnghg avasaagcgs ggraaaggek r

Elegans XP_041565851.1

1 mfaalmdvgq tlagslateg ndsgllatgq yeggvevgmg qhnssadggm vpyvpvldrp

```
   61 etyivtvlyt lifivgvlgn gtlviiffrh rsmrnipnty ilslaladll vilvcvpvat
  121 ivytqeswpf ernmcrisef fkdisigvsv ftltalsger ycaivnplrk lqtkpltvft
  181 aviiwilail lgmpsflvsd iksypvytan gnmtievcsp frdpeyakcm vaakafiyyl
  241 lplsiigaly immakrlhms arnmpgeqqs mqsrtqarar rhvarmvvaf vvvfficffp
  301 yhvfelwyhf yptaeedfdd fwnvlrivgf ctsflnscvn pvalycvsgv frqhfnrylc
  361 cicvkrqphl rqhstatgmm dntsvmsmrr styvggaaag qlraslhrns snhgggvgfg
  421 sgrvgsfhrq dsmalqhgng hgaatggasc apgsggrgpa ggekr
```

Kikkawei **KAH8308790.1**

```
 1 vntnkmdiaq alvaslahap ldgsgngsgs ggndsvllat gasaeqhnas idggmvpyvp
   61 vldrpetyiv tvlytlifiv gvlgngtlvi iffrhrsmrn ipntyilsla ladllvilvc
  121 vpvativytq eswpfernmc riseffkdis igvsvftlta lsgerycaiv nplrklqtkp
  181 ltvftavmiw ilaillgmps flvsdiksyp vltangnmti evcspyrdpe yaqcmvaaka
  241 fiyyllplsi igalyimmak rlhisardmp geqqsmqsrt qararrhvar mvvafvvvff
  301 icffpyhvfe lwyhfyptae edfddfwnvl rivgfctsfl nscvnpvaly cvsgvfrqhf
  361 nrylccicvk rqphlrqhst atgmmdntsv msmrrstyvg gcgaggnlra slhrnsnqgv
  421 ggggalgggt grvgsfhrqd smplqhgngh gaaagggtas gpscgsagra vavgek
```

Bipectinate **XP_017092331.2**

```
1 msalmdmsqt lalsllyapp datggvspni isigsgngsd gggndsslgl atgqqgaata
   61 vggvlglgqh nasadggggp yvpvlerpet yivtvlytli fivgvlgngt lviiffrhrs
  121 mrnipntyil slaladllvi lvcvpvativ ytqeswpfer nmcriseffk disigvsvft
  181 ltalsgeryc aivnplrklq tkpltvftaa miwvlaillg mpsflvsnik sytvltpngn
  241 msievcdpfr dpeyakymva akasiyyllp lsiigalyim makrlhisar dmpgeqqsmq
  301 srsqararrh varmvvafvv vfficffpyh vfelwyhfyp taeedfdefw nvlrivgfct
  361 sflnscvnpv alycvsgvfr qhfnrylccf cvkrqphlrq hstatgimdn tsvmsmrrst
  421 yvggaggnlr asmhrnsnhg ggggsglsag rgasfhggds mpmqhsnghg aaggtlggag
  481 vgatggpssg raaavgekr
```

Anannassae **XP_001961345.2**

```
1 msaalmdmsq tlalslvyap ldatggaspn iaigngndsn gggdggands glvlatgqqg
   61 vatvvggvlg lgqhngsadg gigpyvpvle rpetyivtvl ytlifivgvl gngtlviiff
  121 rhrsmrnipn tyilslalad llvilvcvpv ativytqesw pfernmcris effkdisigv
  181 svftltalsg erycaivnpl rklqtkpltv ftaamiwvla illgmpsflf sniksytvlt
  241 pngnmsievc dpyrdpeyak ymvaakasiy ylvplsiiga lyimmakrlh isardmpgeq
  301 qsmqsrsqar arryvarmvv afvvvfficl fpyyvfelwy hfyptaeedf defwnvlriv
  361 gfctsflnsc vnpvalycvs gvfrqhfnry lccfcvkrqp hlrqhstatg imdntsvmsm
  421 rrstyvggag gnlrssmhrn snhgggggag lpagrgasfh ggdsmplqhs nghgatggns
  481 ggagasatgg pslgrtaavg ekr
```

Mojavensis **XP_002006847.2**

```
1 myaalmdmsq mlaaslayap aesssaaaaa gvnlsqlsns sqldgssvvt vataatvgqh
   61 nasieessyv kvldrpetyi vtvlytlifi vgvlgngtlv iiffrhrsmr nipntyilsl
  121 aladllvilv cvpvativyt qeswpfernm criseffkdi sigvsvftlt alsgerycai
  181 vnplrklqtk pltvftavii wvfaimlgmp sfvvsdiqsy nittpngnit igvcspfrsk
  241 iyakymvvak asiyylvpls iigvlyimma krlhisardm pgeqlsiqsr sqararrhva
  301 rmvvafvvvf ficffpyhvf elwyhfypta eddfddfwnv vrivgfctsf lnscvnpval
  361 ycvsgvfrqh fnrylccicv krqphlrqhs tatgimdtsv tsmrrstyvg ggiaaggare
  421 ggprasvhmn nnhgvsgaag grggsfhrqd smalqhagsv nghshnanvg agagigrasi
  481 inekr
```

Virilis **XP_002049178.2**

```
1 mpknmlaalm dmsqtlaasl ayaplesnaa ataaaaaaaa vvlnvsqlgn ssqldgslat
   61 aaatttttav ttststhnas geeypqyvkv ldrpetyivt vlytlifivg vlgngtlvii
  121 ffrhrsmrni pntyilslal adllvilvcv pvativytqe swpfernmcr iteffkdisi
  181 gvsvftltal sgerycaivn plrklqtkpl tvftaviiwv faimlgmpsf vvsdiqgytl
  241 ptpngnitie vcspfrskiy akymvvakas iyylvplsii gvlyimmakr lhisardmpg
  301 eqlsiqsrsq ararrhvarm vvafvvvffi cffpyhvfel wyhfyptaee dfddfwhvvr
  361 ivgfctsfln scvnpvalyc vsgvfrqhfn rylccicvkr qphlrqhsta tgvmdtsvts
  421 mrrstyvggg gggavggsla ahraslhmnn nhgvavgggg ggggrggsfh rqdsmplqha
  481 gsgnghahnv ggpgagigra siinekslik rydertry
```

Grimshawi **XP_001985716.1**

```
1 myaalmdmsq tlaaslayap mesnsaaaaa aaaaaaalgm nvsqlsnssq ldgstgataa
```

```
 61 tsiphnvsae eypqyvkvld rpetyivtvl ytlifivgvl gngtlviiff rhrsmrnipn
121 tyilslalad llvilvcvpv ativytqesw pfernmcris effkdisigv svftltalsg
181 erycaivnpl rklqtkpltv ftaviiwvla imlgmpsfvv sdlqgytlpt nkgnitievc
241 spfrskiyak ymvvakasiy yfvplsiigv lyimmakrlh isardmpgeq lsiqsrsqar
301 arrhvarmvv afvvvfficf fpyhvfelwy hfyptaeddf ddfwhvvriv gfctsflnsc
361 vnpvalycvs gvfrqhfnry lccfcvkrqp hlrqhstatg vmdtsvtsmr rstyvggggg
421 vggatgslaa hraslhmnnn hgsgaggpgg rtgsfhrqds mplqhagsgn ghahnvggig
481 rasiineksl ikryeerary
```

"CCHa2-R PA isoform – data capture":
