# Supplementary material for: The incidence of candidate binding sites for β-arrestin in Drosophila neuropeptide GPCRs
Source: PLoS One. 2022 Nov 1;17(11):e0275410. doi: 10.1371/journal.pone.0275410 (PMC9624432; doi:10.1371/journal.pone.0275410)
Supplement: S14 Text — (PDF) [file pone.0275410.s018.pdf]

S14. Text **Multi-species analysis of LGR1**
Supporting Figure 17

CLUSTAL Line-ups; Genbank Reference IDs below
Predicted TM domains in YELLOW
BBS sequences in RED

```
Mojavensis        --------------------------MKCTPIIARFDFSAV--I-----------LLSLV      21
Virilis           --------------------------MKCMVIISQLDFLTF--I-----------LLSLA      21
Bipectinata       --------------------------MKCVLPKFVFRLLL-HHLLLTKLCWLHGVYATS-      32
Anannassae        --------------------------MKCVLPKFVYRLLL-HHLLLTRICWPHGVYATS-      32
Kikkawei          ------MGRRQTNWVRRKGRGQPKTALTCLSIGFLFRFLFVYHLLLAPLCGPHCVYAMLA      54
Serrata           ------MGRRRTHWVRKGDRRPVKTGIKCLSIGYLLRLLFVHHLLLGSFCGSHRVYAMLA      54
Ficusphelia       MKKH-------TNLHRKEVTWRSRRGTKCLTFEFLFRLLY-NHLLLTSMFGPDCVYATSA      52
Erecta            MEKHPSLPPSQTQTQRRDTTCRSKKGLKCLSFEFQCRLLL-HHLLLTSLSGRHCVYAM--      57
Melanogaster      MEKHPSLS------QRMGTTYRPRKGLKCLSFEFQCRLLL-HHLLLTSLSGRHFVYATSA      53
Sechellia         MEKHPSMS------QRLETTCRPRKGLKCLSFEFQCRLLL-HHLLLTSLSGRHCVYATSA      53
Mauritania        MEKHPSLS------QRMDTTCRPRKGLKCLSFEFQCRLLL-HHLLLTSLSGRHCVYATSA      53
Simulans          MEKHPSLS------QRMETTCRPRKGLKCLSFEFQCRLLL-HHLLLTSLSGRHCVYATSA      53
Eugracilis        MEKHTNLC-------RTESTWRPKKGLKCLSFEYLFRLLL-HHLLLTSLSGPHCVYATSD      52
Takahashi         MEKHTSLP-------RKEASESSRKGLKCLSLEFLFRLLF-HQLLFTSLCGPHCVYATSA      52
Suzuki            MEKHTSQY-------PKESPWRTKRGLKCLTFEFQFRLLL-HHLMLSSLCGPHCVYATSA      52
Biarmipes         MEKHTSQC-------PKEAPWRPKRGLKCLTIEFQFRLLL-HHLLLSSLCGPHCVYATSA      52
Elegans           MEKPAILAR----KDVLRRSRKCSKGLKCLSFDFQFRLLL-HYLLLSSLSGPHCVYATMA      55
Rhopaloa          MEKYTILAR----KRAR---RPSKRILKCLSLEFLYRLLL-HHLLLTSLCDHHYVYATSA      52
                                             .*        :     :

Mojavensis        YCSQQASTNCYDNHDGFNAIINNLPRDAGNA-----------IDTLDTLAQAPPMTTNPP      70
Virilis           HGTRQANTNCHDNNNGFNTVFNNLSIDNGNE-----------TDMSVIMTQAPTMSTNPT      70
Bipectinata       -------ASCHDSYNGFNVFPNGSSDLRP---DP------NPTDVSSGQAP------PPT      70
Anannassae        -------AVCHDSHNGFNVSPDGNSDLSP---DR------NPTTMSSGQAP------PPT      70
Kikkawei          AEGPSDGSNCHDIHHGFDVPSSLNAVT-------------------EAQGGDAPMAVTLT      95
Serrata           AEGQSVRSNSHDVHHGFDVPGRQP-VS-------------------DNQVRGAPITVILP      94
Ficusphelia       VGKALSASNCHDIHHGFDVFPNPNPTPALNAVDSNPNTSSNATAMWLGQSTDTP--VTLT      110
Erecta            -----SASSCHDIHHGFDVYPNPNPT-----------------TVSLGQSTDTP-Q-AEA      93
Melanogaster      VGGALSANNCHDIHHGFDVYPNL--T-----------------AVSLAQSTDTP-L-TAT      92
Sechellia         AGGALSASNCHDIHHGFDVYPNP--T-----------------AVSLAQSTDTP-L-TAA      92
Mauritania        AGGALSASNCHDIHHGFDVYPNP--T-----------------AVSLAQSTDTP-L-TAA      92
Simulans          AGGALSASNCHDIHHGFDVYPNP--T-----------------AVSLAQSTDTP-L-TAA      92
Eugracilis        IGEALSASNCHDIHHGFDVYPNPNAL-------------------SLGQSTDTP--LTQT      91
Takahashi         VGEALGASNCHDILHGFDVYPNPTAG-------------------ALGQSTDSP--LTVT      91
Suzuki            VGEALSASNCHDIHHGFDVYPNPNPSP-------------NLTAVTLGQSTDTP-SVTLT      98
Biarmipes         VGEALSASNCHDIHHGFDVYPNPNLNPTS---------SANGTTVTLGQSRDTP-SVPLT      102
Elegans           GSEALSASNCHDIHHGFDVYPNPTAV-------------------SQGQSTDTP--LTLT      94
Rhopaloa          VGEPISASDCHDIHHGFDVFPNHTAL-------------------PLGQSTDTP--LTST      91
                           .:*  .**:.

Mojavensis        VDASVWKCCCWEATNHN-EFECRCEGEALTRVPQTLKLPLQRLTIASAGLPRLRSMGLKV      129
Virilis           SDASVWKCCCWEATNQN-EFECRCEGEALTRVPQTLKLPLLRLTIASAGLPRLRSMGLKV      129
Bipectinata       LTLPPWKCCCWNATNQSEEAECRCEGEALTRVPQTLTMAMQRLTIATAGLPRLRATGLKV      130
Anannassae        LTLPPWKCCCWNATNQSEEVECRCEGEALTRVPQTLTMAMQRLTIASAGLPRLRATGLKV      130
Kikkawei          PAPSGWKCCCWKAPNQGEEVECRCEGDALTRVPQTLQLPIQRLTIASAGLPRLRFTGLKV      155
Serrata           KVTSRWKCFCSKTPNQSEELECRCEGDSLTRVPQTLQLPIQRLTIASAGLPRLRSTGLKV      154
Ficusphelia       LPPSAWKCCCWNASNQNEEVECRCEGDGLNRVPQTLKLPLQRLTIASAGLPRLRYTGLKV      170
Erecta            MPPSAWKCCCWNASNQVEEVECRCEGDGLNRVPQTLKLPIQRLTIASAGLPRLRHTGLKV      153
Melanogaster      MPRSAWKCCCWNASNQAEEVECRCEGDGLNRVPQTLTLPIQRLTIASAGLPRLRHTGLKV      152
Sechellia         MPRSAWKCCCWNASNQAEEVECRCEGDGLNRVPQTLTLPIQRLTIASAGLPRLRHTGLKV      152
Mauritania        MPRSAWKCCCWNASNQAEEVECRCEGDGLNRVPQTLTLPIQRLTIASAGLPRLRHTGLKV      152
Simulans          MPRSAWKCCCWNASNQAEEVECRCEGDGLNRVPQTLTLPIQRLTIASAGLPRLRHTGLKV      152
Eugracilis        LPPSAWKCCCWNASNQMEEVECRCEGDGLNRVPQTLKLPIQRLTIASAGLPRLRYTGLKV      151
Takahashi         LPPSGWKCCCWNASNQMEEVECRCEGDGLNRVPQTLKLPIQRLTIASAGLPRLRYTGLKV      151
Suzuki            LPPSGWKCCCWNASNQAEEVECRCEGDGLNRVPQTLKLPIQRLTIASAGLPRLRYTGLKV      158
Biarmipes         LPPSGWKCCCWNGGNQAEEVECRCEGDGLNRVPQTLKLPLQRLTIASAGLPRLRYTGLKV      162
Elegans           LPPSGWKCCCWNASNQNEEVECRCGGDGLNRVPQTLKRPIQRLTIASAGLPRLRNTGLKV      154
Rhopaloa          LPPSGWKCCCWKSSNQNEEVECRCEGDGLNRVPQSLKLPIQRLTIASAGLPRVRFTGLKV      151
                       *** * :  *:  * **** *:.*.****:*   : *****:*****:*  ****
```

```
Mojavensis      YAPTLLDVAFIDCLQLETIQSGAFSNLTVLRAIYISNAPKLSYLAKNVFEGISDTIEIIR    189
Virilis         YATTLLDVAFIDCLQLEAIQNGAFSNLTFLRTIYISNAPKLTYLPKNVFEGISDTIEIIR    189
Bipectinata     YAQTLMDVAFTDCLQLELIQDGAFANLKLLRTIYIANAPKLTFLSKDVFFGISDTVEIIR    190
Anannassae      YAQTLLDVAFTDCLQLELIQDGAFANLKLLRTIYIANAPKLSFLSKDVFSGISDTVEIIR    190
Kikkawei        YGPTLLDVAFTDCLKLELIQDGAFANLTLLRTIYISNAPKLTFLSKDVFAGISETVEIIR    215
Serrata         YGPTLLDVAFTDCLKLELIQDGAFANLTMLRTIYISNAPKLTFLSKDVFAGISETVEVIR    214
Ficusphelia     YGPTLLDVTFTDCLQLELIQDGAFANLTLLRTIYISNAPKLTFLSKDVFAGISESVEIIR    230
Erecta          YGPTLLDVAFTDCLQLELIQDGAFANLTLLRTIYITNAPKLTFLSKDVFLGISDTVEIIR    213
Melanogaster    YGSTLLDVAFTDCLQLELIQDGAFANLTLLRTIYITNAPKLTFLSKDVFLGISDTVDIIR    212
Sechellia       YGSTLLDVAFTDCLQLELIQDGAFANLTLLRTIYITNAPKLTFLSRDVFLGISDTVDIIR    212
Mauritania      YGSTLLDVAFTDCLQLELIQDGAFANLTLLRTIYITNAPKLTFLSKDVFLGISDTVDIIR    212
Simulans        YGSTLLDVAFTDCLQLELIQDGAFANLTLLRTIYITNAPKLTFLSKDVFLGISDTVDIIR    212
Eugracilis      YGPTLLDVAFTDCQQLELIQDGAFSNLTLLRTIYITNAPKLTFLSKDVFLGISDTVEIIR    211
Takahashi       YGPTLLDVTFTDCLQLELIQDGAFANLTLLRTIYITNAPKLTFLSKDVFFGISDTVEIIR    211
Suzuki          YGPTLLDVAFTDCLQLELIQDGAFANLTLLRTIYITNAPKLTFLSKDVFFGISGTVEIIR    218
Biarmipes       YGATLLDVAFTDCLQLELIQDGAFANLTLLRTIYITNAPKLTFLSKDVFLGISGTVEVIR    222
Elegans         YGSTLLDVAFTDCLQLELIQDGAFANLTLLRTIYISNAPKLTFLSKDVFFGISGSVEIIR    214
Rhopaloa        YGSSLLDVAFIDCLQLELIQDGAFANLALLRTIYISNAPKLTFLSKDVFSGISGSVEIIR    211
                *. :*:**:* ** :** **.***:** .**:***:*****::* ::** *** ::::**

Mojavensis      IINSGLKTVPDLGDLPPYNILQMIDLDNNQISRIDSKSIQVKTAQLVLANNEITFIDDSA    249
Virilis         IINSGLTSVPDFGYLPPNNILQMIDLDNNQISRIDSKSIQVKTAQFVLANNDIHFIDDSA    249
Bipectinata     IINSGLTRVPDLTHLPPYNILQMIDLDNNQITRIDAKSIKVKTAQLILANNDISYVDDSA    250
Anannassae      IINSGLTSVPDLTHLPPYNILQMIDLDNNQITRIDSKSIKVKTAQLILANNDISYVDDSA    250
Kikkawei        IINSGLTSVPDLGHLPPHNILQMIDLDNNQITRIDTKSINVKTAQFILANNDISYVDDSA    275
Serrata         IINSGLTRVPDLGHLPPHNILQMIDLDNNQITRIDTKSINVKTAQLILANNDISYVDDSA    274
Ficusphelia     IINSGLTRVPDLGHLPPHNILQMIDLDNNQISRIDTKSIKVKTAQLILANNDISYVDDSA    290
Erecta          IINSGLTRVPDLGHLPPHNILQMIDLDNNQITRIDSKSIKVKTAQLILANNEISYVDDSA    273
Melanogaster    IINSGLTRVPDLGHLPPHNILQMIDLDNNQITRIDSKSIKVKTAQLILTNNEISYVDDSA    272
Sechellia       IINSGLTRVPDLGHLPPHNILQMIDLDNNQITRIDSKSIKVKTAQLILTNNEISYVDDSA    272
Mauritania      IINSGLTRVPDLGHLPPHNILQMIDLDNNQITRIDSKSIKVKTAQLILTNNEISYVDDSA    272
Simulans        IINSGLTRVPDLGHLPPHNILQMIDLDNNQITRIDSKSIKVKTAQLILTNNEISYVDDSA    272
Eugracilis      IINSGLTRVPDLSHLPPNNILQMIDLDNNQITRIDTKSIKVKTAQLILANNEISYIDDSA    271
Takahashi       IINSGLTRVPDLSHLPPHNILQMIDLDNNQITRIDTKSIKVKTAQLILANNDISYVDDSA    271
Suzuki          IINSGLTRVPDLSHLPPHNILQMIDLDNNQITRIDTKSIKVKTAQLILANNDISYVDDSA    278
Biarmipes       IINSGLTRVPDLSHLPPHNILQMIDLDNNQITRIDTKSIKVKTAQLILANNDISYVDDSA    282
Elegans         IINSGLTRVPDLGHLPPHNILQMIDLDNNQITRIDTKSIKVKTAQLILANNDISYVDDSA    274
Rhopaloa        IINSGLTRVPDLGHLPPHNILQMIDLDNNQITRIDTKSINVKTAQLILANNDISYVDDSA    271
                ******. ***:  *** *************:***:***:*****::*:**:* ::****

Mojavensis      FLGSKIAKLSLNDNHKLTEIHPNAFNGIIDMTELDLSSTSLVRLPSAGLQTLEVLYIANT    309
Virilis         FLGSKIAKLSLKDNRRLTDVHPNAFYGIIDITELDLSSTSLVSLPSAGLQTVEVLYIINT    309
Bipectinata     FCGSKIAKLSLKENRKLKELHTNAFHGIIDITELDLSSTSIVEMPSAGLQTIEALYILNT    310
Anannassae      FFGSKIAKLSLKENRKLKELHTNAFHGIIDITELDLSSTSIVEMPSAGLQTIEALYILNT    310
Kikkawei        FFGSKIAKLSLKDNRKLTDMHPNAFNGIIDIAELDLSSTSLVGLPSVGLQTIEALYIMNT    335
Serrata         FFGSKIAKLSLKDNWKLTEMHPEAFNGIIDITELDLSSTSLVGLPSVGLQTIEALYIMNT    334
Ficusphelia     FFGSKIAKLSLQNNRKLKQMHPNAFDGIIDITELDLSSTSLVGLPSVGLQNIEALYITNT    350
Erecta          FFGSKIAKLSLKENKRLKMMHPNAFDGIIDIAELDLSSTSLVGLPSAGLQNIEALYIQNT    333
Melanogaster    FFGSKIAKLSLKENKKLQMMHPNAFDGIIDITELDLSSTSLVGLPSAGLQNIEALYIQNT    332
Sechellia       FYGSKIAKLSLKENKKLEMMHPNAFDGIIDIAELDLSSTSLVGLPSAGLQNIEALYIQNT    332
Mauritania      FFGSKIAKLSLKENKKLQMMHPNAFDGIIDIAELDLSSTSLVGLPSAGLQNIEALYIQNT    332
Simulans        FFGSKIAKLSLKENKKLQEMHPNAFDGIIDIAELDLSSTSLVGLPSAGLQNIEALYIQNT    332
Eugracilis      FFGSKIAKLSLKDNLKLEMMHPKAFDGIIDITELDLSSTSLVGLPSVGLQTIEALYIQNT    331
Takahashi       FFGSKIAKLSLNDNQKLEHMHPNAFDGIIDITELDLSSTSLVGLPSSGLQTIEALYIQNT    331
Suzuki          FFGSKIAKLSLNDNLRLEHMHPNAFDGI-DITELDLSSTSLVAMPSVGLQNIEALYIQNT    337
Biarmipes       FYGSKIAKLSLNDNLRLEHMHPNAFDGIIDITELDLSSTSLVAMPSVGLQNIEALYIQNT    342
Elegans         FFGSKIAKLSLKDNPKLKELHPNAFDGIIDITELDLSSTSLVGMPSVGLQTIEALYIQNT    334
Rhopaloa        FFGSKIAKLSLNDNPKLKELHPNAFNGIIDITALDLSSTSLIGMPSAGLQTIEALYIQNT    331
                * *********::* :*  :* :** ** *:: *******:: :** ***.:*.*** **

Mojavensis      HTLKTIPSIYNFQNLQRAHLTHSFHCCAFQFPSRHDPQRHAQRMQEVEKWRDQCKNNRDV    369
Virilis         HTLKTIPSIYNFQNLQRAHLTHSFHCCAFQFPSRHDPKRHAERLQELQKWREQCNVERDL    369
Bipectinata     HTLKTIPSIYNFRNLQRAYLTHSFHCCAFQFPSRHDPLRHAQRMDEIEKWRTQCKGERIS    370
Anannassae      HTLKTIPSIYNFRNLQRAYLTHSFHCCAFQFPSRHDPLRHAQRMLEIEKWRTQCNGERNS    370
Kikkawei        HTLKTIPSIYNFRNLQRAYLTHSFHCCAFQFPSRHDPRRHAQRMLEIEKWREQCNK--GS    393
Serrata         HTLKTIPSIYNFRNLQRAYLTHSFHCCAFQLPSRHDPRRHALRMLEIEKWREQCNK--CS    392
Ficusphelia     HTLKTIPSIYNFRNLQRAYLTHSFHCCAFQFPSRHDPQRHAERMREIEKWREQCKSDHGS    410
Erecta          HTLKTIPSIYNFRNLQRAYLTHSFHCCAFQFPSRHDPQRHAQRMLEIEKWRKQCKSESGS    393
Melanogaster    HTLKTIPSIYNFRNLQRAYLTHSFHCCAFQFPSRHDPQRHAQRMLEIEKWRKQCKSDSGT    392
Sechellia       HTLKTIPSIYNFRNLQRAYLTHSFHCCAFQFPSRHDPQRHAQRMLEIEKWRKQCKSDSGS    392
```

```
Mauritania       HTLKTIPSIYNFRNLQRAYLTHSFHCCAFQFPSRHDPQRHAQRMLEIEKWRKQCKSDSGS    392
Simulans         HTLKTIPSIYNFRNLQRAYLTHSFHCCAFQFPSRHDPQRHAQRMLEIEKWRKQCKSDSGS    392
Eugracilis       HTLKTIPSIYNFRNLQRAYLTHSFHCCAFQFPSRHDPQRHAQRMLEIEKWRKQCKSDSGS    391
Takahashi        HTLKTIPSIYNFRNLQRAYLTHSFHCCAFQFPSRHDPQRHAKRMREIEKWREQCKSDSAS    391
Suzuki           HTLKTIPSIYNFRNLQRAYLTHSFHCCAFQFPSRHDPQRHAQRMLEIEKWRKQCKSESVS    397
Biarmipes        HTLKTIPSIYNFRNLQRAHLTHSFHCCAFQFPSRHDPQRHAERMLEIEKWRKQCKSDSVT    402
Elegans          HTLKTIPSIYNFRNLQRAYLTHSFHCCAFQFPSRHDPQRHAQRMLEIQKWREQCKSDHVS    394
Rhopaloa         HTLKTIPSIYNFRNLQRAYLTHSFHCCAFQFPSRHDPERHAQRMLEIEKWRKQCKSDQVS    391
                 ************:*****:***********:****** *** *: *::*** **:

Mojavensis       HTNYERSLSMS-NLTPLANVSEVQPSGSTINNLLAESTPNTYAYMADSTLNNIGIFHEEI    428
Virilis          YRDVDKKLKKNTSVKDTAGVHYTNQSGTATDNLLTDAASNSYDYMADSTMNNIGIFHEQI    429
Bipectinata      RKERSILNNLKSQSDGYGIFG-SEASPT----DVVSTQFASVDYMADAAMN-LGYFHEEI    424
Anannassae       RRERNIAKKIESQQDDLGTLGGSEALSA----DVLSTPFSSVDYMADAAMN-LGYFHEEI    425
Kikkawei         RKERSLLDNFEAQPEDFGSFGSTEPSVT----EITPYPYASVDYMADS-TN-LGYFHEQI    447
Serrata          RKERALLDYLEAQPEDFGSFGSTEPSMT----ENTPFPIASIDYMADS-TN-LGYFHEQI    446
Ficusphelia      RKERSALDGFEGLPEDFGTFGMPEQSGT----DDTSITYASFDYMSDDTLN-KGTFHEKI    465
Erecta           RQERSTLDSSLSMPEDFGTFSGTDDSAT----DITPITFVSPDYMADDTMN-KGTFHEKI    448
Melanogaster     RKERSTLDNPFNMPEDFGSFGGTDDSAT----DITPITFASFDYMADDTMN-KGTFHEKI    447
Sechellia        RKERSTLDNSFSMPEDFGSFGGTDDSST----DITPITFASFDYMADDTMN-KGTFHEKI    447
Mauritania       RKERSTLDNSFDMPEDFGSFGGTDDSAI----DITPITFASFDYMADDTMN-KGTFHEKI    447
Simulans         RKERSTLDNSFNMPEDFGSFGGTDDSAT----DMTPITFASFDYMADDTMN-KGTFHEKI    447
Eugracilis       RKERSTLDGLDNQPEDFGSFGGSYQSAT----DISPITFSSIDYMADDTMN-KGTFHEKI    446
Takahashi        RKERSTSENGFEMPEDFGSFGSPDEATT----DIVPIKFASFDYMADDTLN-KGTFHEKI    446
Suzuki           RQERSTLDNAYTMPEDFGTFESTDESST----DLVPITFASFDYMADDSLN-KGTFHEKI    452
Biarmipes        RKERSTLENGFTLPEDFGTFGGTDDSST----DLVPITFASFDYMADDTLI-KGTFHEKI    457
Elegans          RKERSTVDNLNSMPEEFGSFGSTDQSAT----DMTSMTYASFDYMSDDTMN-KGTFHEKI    449
Rhopaloa         RKERSIADNLNSVPEEFGSFGMTDQ---------------SLDYMSDDTMN-KGTFHEKI    435
                   :              . .                    :  **:*      * ***:*

Mojavensis       TINPDDDQLAEYCGNFTFRKPHVECYPMPNALNPCEDVMGYQWLRIAVWIVVALAIVGNV    488
Virilis          TINPDDNQLAEYCGNFTFRNPDIQCFPMPNALNPCEDVMGYQWLRISVWIVVALAIVGNL    489
Bipectinata      TLNPDDEQFAEFCGNFTFRKPNVECYPMPNALNPCEDVMGYQWLRISVWVVVALAVVGNV    484
Anannassae       TINPDDEQFAEFCGNFTFRKPSVECYPMPDALNPCEDVMGYQWLRISVWVVVALAVVGNV    485
Kikkawei         TINPDDDQSAEFCGNFTFRKPNIECYPMPNDLNPCEDVMGYQWLRISVWIVVALAVVGNV    507
Serrata          TINPDDKQSAEFCGNFTFRKPNIECYPMPNDLNPCEDVMGYQWLRISVWIVVALSAVGNV    506
Ficusphelia      TLNPEDDMTAELCGNFTFRKPNIECYPMPNDLNPCEDVMGYQWLRISVWIVVALAVVGNV    525
Erecta           VLNPEDDSSAELCGNFTFRKPNIECYPMPNDLNPCEDVMGYQWLRISVWIVVALAVVGNV    508
Melanogaster     ILNPGDDSSAELCGNFTFRKPNIECYPMPNDLNPCEDVMGYQWLRISVWIVVALAVVGNV    507
Sechellia        ILNPGDDSSAELCGNFTFRKPNIECYPMPNDLNPCEDVMGYQWLRISVWIVVALAVVGNV    507
Mauritania       ILNPGDDSSAELCGNFTFRKPNIECYPMPNDLNPCEDVMGYQWLRISVWIVVALAVVGNV    507
Simulans         ILNPDDDSSAELCGNFTFRKPNIECYPMPNDLNPCEDVMGYQWLRISVWIVVALAVVGNV    507
Eugracilis       TLNPEDDMSAELCGNFTFRKPNIECYPMPNDLNPCEDVMGYQWLRISVWIVVALAVVGNV    506
Takahashi        TLDPADDMSAELCGNFTFRKPNIECYPMPNDLNPCEDVMGYQWLRISVWIVVALAVVGNV    506
Suzuki           TLNPEDDMSAELCGNFTFRKPNIECYPMPNDLNPCEDVMGYQWLRISVWIVVALAVVGNV    512
Biarmipes        ILNPEDDMSAELCGNFTFRKPNIECYPMPNDLNPCEDVMGYQWLRISVWIVVALAVVGNV    517
Elegans          TLNPEDDI-DELCGNFTFRKPNIECYPMPNDLNPCEDVMGYQWLRISVWIVVALAVVGNV    508
Rhopaloa         TLNPEDDI-DELCGNFTFRKPNIECYPMPNDLNPCEDVMGYQWLRISVWIVVALAVVGNV    494
                  ::* *.   * *******:* ::*:***: ***************:**:****: ***:

Mojavensis       AVLTVILSIKSESPSVPRFLICHLAFADLCLGLYLLLIASIDAHSMGEYFNYAFDWQYGL    548
Virilis          AVLTVTLSIKSESPSVPRFLICHLAFADLCLGLYLLLIASIDAHSMGEYFNYAFDWQYGL    549
Bipectinata      AVLTVNLSIRPEITPVARFLMCHLAFADLCLGLYLFLVASIDAHSMGEYFNYAYDWQYGL    544
Anannassae       AVLTVNLSIRPEITPVARFLMCHLAFADLCLGLYLFLVASIDAHSMGEYFNYAYDWQYGL    545
Kikkawei         AVLTVILSIRPESTPVPRFLMCHLAFADLCLGVYLLLVASIDAHSMGEYFNYAYDWQYGL    567
Serrata          AVLTVILSIRPESSPVPRFLMCHLAFADLCLGVYLLLVASIDAHSIGEYFNYAYDWQYGL    566
Ficusphelia      AVLTVILSIRPESTPVPRFLMCHLAFADLCLGVYLLLVASIDAHSMGEYFNYAYDWQYGL    585
Erecta           AVLTVILSIRPESTPVPRFLMCHLAFADLCLGLYLLLVACIDAHSMGEYFNFAYDWQYGL    568
Melanogaster     AVLTVILSIRPESTPVPRFLMCHLAFADLCLGLYLLLVACIDAHSMGEYFNFAYDWQYGL    567
Sechellia        AVLTVILSIRPESTPVPRFLMCHLAFADLCLGLYLLLVACIDAHSMGEYFNFAYDWQYGL    567
Mauritania       AVLTVILSIRPESTPVPRFLMCHLAFADLCLGLYLLLVACIDAHSMGEYFNFAYDWQYGL    567
Simulans         AVLTVILSIRAESTPVPRFLMCHLAFADLCLGLYLLLVACIDAHSMGEYFNFAYDWQYGL    567
Eugracilis       AVLTVILSIRPETTPVPRFLMCHLAFADLCLGVYLLLVASIDAHSKGEYFNFAYDWQYGL    566
Takahashi        AVLTVILSIRPESTPVPRFLMCHLAFADLCLGVYLLLVASIDAHSMGEYFNYAYDWQYGL    566
Suzuki           AVLTVILSIRPESTPVPRFLMCHLAFADLCLGVYLLLVASIDAHSMGEYFNYAYDWQYGL    572
Biarmipes        AVLTVILSIRPESTPVPRFLMCHLAFADLCLGVYLLLVASIDAHSMGEYFNYAYDWQYGL    577
Elegans          AVLTVILSIRPESTPVPRFLMCHLAFADLCLGVYLLFVASIDAHSMGEYFNFAYDWQYGL    568
Rhopaloa         AVLTVILSIRPESTPVPRFLMCHLAFADLCLGVYLLLVASIDAHSMGAYFNYAYDWQYGL    554
                 ***** ***: *   * ***:***********:**:::*.***** * ***:*:******
```

```
Mojavensis      GCKIAGFLTVFASHLSVFTLTVITIERWFAITHAMYLNKRIKLRPAAVIMLMGWIYSIVM     608
Virilis         GCKIAGFLTVFASHLSIFTLTIITIERWFAITHAMYLNKRITLRQAAGIMLTGWIYSIIM     609
Bipectinata     GCKVAGFLTVFASHLSVFTLTLITIERWVAITQAMYLNKRIRLRSASIIMLGGWIYSMVM     604
Anannassae      GCKVAGFLTVFASHLSVFTLTLITIERWVAITQAMYLNKRIRLRSASIIMLGGWIYSMVM     605
Kikkawei        GCKAAGFLTVFASHLSVFTLTVITIERWLAITQAMYLNHRIKLRQAALIMLGGWLYSMFM     627
Serrata         GCKVAGFLTVFASHLSVFTLTVITIERWLAITQAMYLNHRIKMRQAALIMLGGWLYSMVM     626
Ficusphelia     GCKVAGFLTVFASHLSVFTLTVITIERCMAITQAMYLNHRIKLRPAAIIMLCGWIYSMLM     645
Erecta          GCKVAGFLTVFASHLSVFTLTVITIERWLAITQAMYLNHRIKLRPAALIMLGGWIYSMLM     628
Melanogaster    GCKVAGFLTVFASHLSVFTLTVITIERWLAITQAMYLNHRIKLRPAALIMLGGWIYSMLM     627
Sechellia       GCKVAGFLTVFASHLSVFTLTVITIERWLAITQAMYLNHRIKLRPAALIMLGGWIYSMLM     627
Mauritania      GCKVAGFLTVFASHLSVFTLTVITIERWLAITQAMYLNHRIKLRPAALIMLGGWIYSMLM     627
Simulans        GCKVAGFLTVFASHLSVFTLTVITIERWLAITQAMYLNHRIKLRPAALIMLGGWIYSMLM     627
Eugracilis      GCKVAGFLTVFASHLSVFTLTVITIERWLAITQAMYLNHRIKLRPAALIMLGGWIYSMFM     626
Takahashi       GCKVAGFLTVFASHLSVFTLTVITIERWLAITQAMYLNHRIKLRPAALIMLGGWIYSMFM     626
Suzuki          GCKAAGFLTVFASHLSVFTLTVITIERWLAITQAMYLNHRIKLRPAALIMLGGWIYSMFM     632
Biarmipes       GCKVAGFLTVFASHLSVFTLTVITIERWLAITQAMYLNHRIKLRPAALIMLGGWIYSMFM     637
Elegans         GCKVAGFLTVFASHLSVFTLTVITIERWLAITQAMYLTHRIKLRQASIIMLGGWIYSVLM     628
Rhopaloa        GCKVAGFLTVFASLLSVFTLTVITIERWLAITQAMYLTHRIKLRPASFIMLGGWIYSILM     614
                *** ********* **:****:***** .***:****.:** :* *: *** **:**:.*

Mojavensis      SSLPLLGISNYSSTSICLPMEKRDIYDSIYLVLILGCNFLAFTIIAICYSQIYLSLGKET     668
Virilis         SSLPLFGISNYSSTSICLPMEIRDIYDSIYLILILGCNFVAFTIIAICYSQIYLSLGQET     669
Bipectinata     SSLPLFGISNYSSTSICLPMEVRDSFDTVYLIAILGCNGVAFFIIAVCYAKIYLSLGRET     664
Anannassae      SSLPLFGISNYSSTSICLPMEVRDTFDTVYLIGILGCNGVAFFIIAVCYAKIYFSLGRET     665
Kikkawei        SSLPLFGISNYSSTSICLPMENRDAFDTMYLIAILGCNGVAFSIIAVCYAQIYLSLGRET     687
Serrata         SSLPLFGISNYSSTSICLPMENRDAFDTMYLIAILGCNGVAFSIIAVCYAQIYLSLGRET     686
Ficusphelia     SSLPLFGISNYSSTSICLPMENRDVFDTMYLIAILGCNGVAFSIIAVCYAQIYLSLGRET     705
Erecta          SSLPLFGISNYSSTSICLPMENRDVYDTVYLIAILGCNGVAFSIIAVCYAQIYLSLGRET     688
Melanogaster    SSLPLFGISNYSSTSICLPMENRDVYDTIYLIAILGSNGVAFSIIAVCYAQIYLSLGRET     687
Sechellia       SSMPLFGISNYSSTSICLPMENRDVYDTIYLIAILGSNGVAFSIIAVCYAQIYLSLGRET     687
Mauritania      SSMPLFGISNYSSTSICLPMENRDVYDTIYLIAILGSNGVAFSIIAVCYAQIYLSLGRET     687
Simulans        SSMPLFGISNYSSTSICLPMENRDVYDTIYLIAILGSNGVAFSIIAVCYAQIYLSLGRET     687
Eugracilis      SSMPLFGISNYSSTSICLPMENRDVYDTVYLVAIMVCNGVAFSIIAVCYAKIYLSLGRET     686
Takahashi       SSLPLFGISNYSSTSICLPMENRDIYDTVYLIAIMVCNGVAFSIIAVCYAQIYLSLGRET     686
Suzuki          SSLPLFGISNYSSTSICLPMENRDVYDTVYLIAIMVCNGVAFSIIAVCYAQIYLSLGRET     692
Biarmipes       SSLPLFGISNYSSTSICLPMENRDGYDTVYLIAIMVCNGVAFSIIAVCYAQIYLSLGRET     697
Elegans         SSLPLFGISNYSSTSICLPMEIRDGFDTAYLIAILACNGVAFTIIAVCYAQIYLSLGRET     688
Rhopaloa        SSLPLFGISNYSSTSICLPMEIRDVFDTVYLIAILACNGVAFSIIAVCYAQIYLSLGRET     674
                **:**:*************** ** :*: **: *: .* :** ***:**::**:***:**

Mojavensis      RRARQNHLGEMSVAKKMALLVFINFSCGAPIAFFGLTALAGYPLINVTKSKILLVFFYPL     728
Virilis         RRARRNNPGEMSVAKKMLLLVFINFTCGAPIAFFGLTALAGCPLINVTKSKILLVFFYPL     729
Bipectinata     RHARQNNPGELSVAKKMALLVFTNFACWSPIAFFGLTALAGFPLINVTNSKILLVFFYPL     724
Anannassae      RHARQNNPGELSVAKKMSLLVFTNFACWSPIAFFGLTALAGYPLINVTNSKILLVFFYPL     725
Kikkawei        RQARQNNPGELSVAKKMALLVFTNFACWSPIAFFGLTALAGFPLINVTKSKILLVFFYPL     747
Serrata         RQAHQNNPGELSVAKKMALLVFTNFACWSPIAFFGLTALAGFPLINVTKSKILLVFFYPL     746
Ficusphelia     RQARHNNPGELSVAKKMALLVFTNFACWSPIAFFGLTALAGYPLINVTKSKILLVFFYPL     765
Erecta          RQAHQNNPGELSVAKKMALLVFTNFACWSPIAFFGLTALAGYPLINVTKSKILLVFFYPL     748
Melanogaster    RQAHQNSPGELSVAKKMALLVFTNFACWSPIAFFGLTALAGYPLINVTKSKILLVFFYPL     747
Sechellia       RQAHQNNPGELSVAKKMALLVFTNFACWSPIAFFGLTALAGYPLINVTKSKILLVFFYPL     747
Mauritania      RQTHQNNPGELSVAKKMALLVFTNFACWSPIAFFGLTALAGYPLINVTKSKILLVFFYPL     747
Simulans        RQAHQNNPGELSVAKKMALLVFTNFACWSPIAFFGLTALAGYPLINVTKSKILLVFFYPL     747
Eugracilis      RQARHNNPGELSVAKKMALLVFTNFACWSPIAFFGLTALAGYPLINVTKSKILLVFFYPL     746
Takahashi       RQAHHNSPGELSVAKKMALLVFTNFACWSPIAFFGLTALAGYPLINVTKSKILLVFFYPL     746
Suzuki          RQAHHNSPGELSVAKKMALLVFTNFACWSPIAFFGLTALAGYPLINVTKSKILLVFFYPL     752
Biarmipes       RQAHQNSPGELSVAKKMALLVFTNFACWSPIAFFGLTALAGYPLINVTKSKILLVFFYPL     757
Elegans         RQARQNSPGELSVAKKMALLVFTNFACWSPIAFFGLTALAGFPLINVTKSKILLVFFYPL     748
Rhopaloa        RQSRQNNPGEMSVAKKMALLVFTNFACWSPIAFFGLTALAGFPLINVTKSKILLVFFYPL     734
                *::::*  **:****** **** **:* :*********** ******:***********

Mojavensis      NSCADPYLYAILTSQYRQDLLTLLSKFGLCRQRALKYKHSDSMHGTSHYTIRGSIEREHS     788
Virilis         NSCADPYLYAILTSQYQQDLLTFLSKLGICRQNALKYKHSDSLHGTSHYTIRGSIEQQSS     789
Bipectinata     NSCADPYLYAILTAQYRQDLYTLLSKLGLCRKNAVNSKDNSSGMGTTRFTIH----RHSS     780
Anannassae      NSCADPYLYAILTAQYRQDLYTLLSKLGLCRTNAVNSKDNSSGMGTTRFTIH----RHSS     781
Kikkawei        NSCADPYLYAILTSQYRQDLFTLLSKIGLCRQNALNYKHSSSAQATTRFTIH----RHSS     803
Serrata         NSCADPYLYAILTSQYRQDLLTLLSKLGLCRQNALNYNHSSSAPATTRFTIH----RHSS     802
Ficusphelia     NSCADPYLYAILTSQYRQDLFTLLSKLGLCRQSALKYKDSLSGQATTRFTIHGSIQRHGS     825
Erecta          NSCADPYLYAILTSQYRQDLFTLLSKLGLCRQSALKYKDSLSGQATTRFTIHGSIQRHSS     808
Melanogaster    NSCADPYLYAILTSQYRQDLFTLLSKLGLCQQSALKYKDSLSGQATTRFTIHGSIQRHSS     807
Sechellia       NSCADPYLYAILTSQYRQDLFTLLSKLGLCRQSALKYKASLSGQATTRFTIHGSIQRHSS     807
Mauritania      NSCADPYLYAILTSQYRQDLFTLLSKLGLCRQSALKYKDSLSGQATTRFTIHGSIQRHSS     807
```

```
Simulans          NSCADPYLYAILTSQYRQDLFTLLSKLGLCRQSALKYKDSLSGQATTRFTIHGSIQRHSS        807
Eugracilis        NSCADPYLYAILTSQYRQDLFTLLSKLGLCRQSALKYKDSLSGHPTSRFTIHGSIQRHGS        806
Takahashi         NSCADPYLYAILTSQYRQDLFTLLSKLGLCRQSALKYKDSLSGHATTRFTIHGSIQRHGS        806
Suzuki            NSCADPYLYAILTSQYRQDLFTLLSKLGLCRQSALKYKDSLSGHATTRFTIHGSIQRHSS        812
Biarmipes         NSCADPYLYAILTSQYRQDLFTLLSKLGLCRQSALKYKDSLSGHATTRFTIHGSIQRHGS        817
Elegans           NSCADPYLYAILTSQYRQDLFTLLSKLGLCRQSALKDKDGSSTRGTTRYTINGSIQRHAS        808
Rhopaloa          NSCADPYLYALLTSQYRQDLFTLLSKLGLCRQSALKYKDSSSAHATSRFTIHNSIHRHGS        794
                  **********:**:**:*** *:***:*:*:  *:: : . *   *:::**.    :. *

Mojavensis        VGQKCQKI-VAAEAQNMLRNNEDYV     812
Virilis           LCQKPQQE-GAAETQTMLKNNEDYV     813
Bipectinata       LTCRIPPALEV-ETQKMLTCNEDYV     804
Anannassae        LTCRIPPALEV-ETQKMLTYSEEYV     805
Kikkawei          LTCKMQAAISV-EAQKMLKNGEDYV     827
Serrata           LTCKMQTVLNA-EAQKMLKNGEDYI     826
Ficusphelia       LTCKMQTVMGT-ETQKMLKCGEDYV     849
Erecta            LTCKMQTVMGA-ETQKMLKNSEDYV     832
Melanogaster      LTCKMQTVMGA-ETQKMLKNSEDYV     831
Sechellia         LTCKMQTLMGA-ETQKMLKNSEDYV     831
Mauritania        LTCKMQTVMGA-ETQKMLKNSEDYV     831
Simulans          LTCKMQTVMGA-ETQKMLKNSEDYV     831
Eugracilis        LTCKMQTTMT-TETQKMLKNSEDYV     830
Takahashi         LTCKMQTVMGATETQKMLKSSEDYV     831
Suzuki            LTCKMQTVMGT-ETQKMLKKSEEYV     836
Biarmipes         LTCKMQTVMGA-ESQKMLKNTEDYV     841
Elegans           LTCKMQTVVGA-ETQKMLKNGEDYV     832
Rhopaloa          LTSKMQTVISA-ETQKMLKNSEDYV     818
```

Variants at this putative BBS do not all have 2 S or Ts, some only 1 – meaning the other species do not contain partial BBS codes at this positions

Melanogaster NP_524393.2

```
mekhpslsqr mgttyrprkg lkclsfefqc rlllhhlllt slsgrhfvya tsavggalsa
       61 nnchdihhgf dvypnltavs laqstdtplt atmprsawkc ccwnasnqae evecrcegdg
      121 lnrvpqtltl piqrltiasa glprlrhtgl kvygstlldv aftdclqlel iqdgafanlt
      181 llrtiyitna pkltflskdv flgisdtvdi iriinsgltr vpdlghlpph nilqmidldn
      241 nqitridsks ikvktaqlil tnneisyvdd saffgskiak lslkenkklq mmhpnafdgi
      301 iditeldlss tslvglpsag lqniealyiq nthtlktips iynfrnlqra ylthsfhcca
      361 fqfpsrhdpq rhaqrmleie kwrkqcksds gtrkerstld npfnmpedfg sfggtddsat
      421 ditpitfasf dymaddtmnk gtfhekiiln pgddssaelc gnftfrkpni ecypmpndln
      481 pcedvmgyqw lrisvwivva lavvgnvavl tvilsirpes tpvprflmch lafadlclgl
      541 ylllvacida hsmgeyfnfa ydwqyglgck vagfltvfas hlsvftltvi tierwlaitq
      601 amylnhrikl rpaalimlgg wiysmlmssl plfgisnyss tsiclpmenr dvydtiylia
      661 ilgsngvafs iiavcyaqiy lslgretrqa hqnspgelsv akkmallvft nfacwspiaf
      721 fgltalagyp linvtkskil lvffyplnsc adpylyailt sqyrqdlftl lsklglcqqs
      781 alkykdslsg qattrftihg siqrhssltc kmqtvmgaet qkmlknsedy v
```

Mauritania XP_033166657.1

```
   1 mekhpslsqr mdttcrprkg lkclsfefqc rlllhhlllt slsgrhcvya tsaaggalsa
       61 snchdihhgf dvypnptavs laqstdtplt aamprsawkc ccwnasnqae evecrcegdg
      121 lnrvpqtltl piqrltiasa glprlrhtgl kvygstlldv aftdclqlel iqdgafanlt
      181 llrtiyitna pkltflskdv flgisdtvdi iriinsgltr vpdlghlpph nilqmidldn
      241 nqitridsks ikvktaqlil tnneisyvdd saffgskiak lslkenkklq mmhpnafdgi
      301 idiaeldlss tslvglpsag lqniealyiq nthtlktips iynfrnlqra ylthsfhcca
      361 fqfpsrhdpq rhaqrmleie kwrkqcksds gsrkerstld nsfdmpedfg sfggtddsai
      421 ditpitfasf dymaddtmnk gtfhekiiln pgddssaelc gnftfrkpni ecypmpndln
      481 pcedvmgyqw lrisvwivva lavvgnvavl tvilsirpes tpvprflmch lafadlclgl
      541 ylllvacida hsmgeyfnfa ydwqyglgck vagfltvfas hlsvftltvi tierwlaitq
      601 amylnhrikl rpaalimlgg wiysmlmssm plfgisnyss tsiclpmenr dvydtiylia
      661 ilgsngvafs iiavcyaqiy lslgretrqt hqnnpgelsv akkmallvft nfacwspiaf
      721 fgltalagyp linvtkskil lvffyplnsc adpylyailt sqyrqdlftl lsklglcrqs
      781 alkykdslsg qattrftihg siqrhssltc kmqtvmgaet qkmlknsedy v
```

Simulans XP_016034243.1

```
1 mekhpslsqr mettcrprkg lkclsfefqc rlllhhlllt slsgrhcvya tsaaggalsa
       61 snchdihhgf dvypnptavs laqstdtplt aamprsawkc ccwnasnqae evecrcegdg
      121 lnrvpqtltl piqrltiasa glprlrhtgl kvygstlldv aftdclqlel iqdgafanlt
      181 llrtiyitna pkltflskdv flgisdtvdi iriinsgltr vpdlghlpph nilqmidldn
```

```
241 nqitridsks ikvktaqlil tnneisyvdd saffgskiak lslkenkklq emhpnafdgi
301 idiaeldlss tslvglpsag lqniealyiq nthtlktips iynfrnlqra ylthsfhcca
361 fqfpsrhdpq rhaqrmleie kwrkqcksds gsrkerstld nsfnmpedfg sfggtddsat
421 dmtpitfasf dymaddtmnk gtfhekiiln pdddssaelc gnftfrkpni ecypmpndln
481 pcedvmgyqw lrisvwivva lavvgnvavl tvilsiraes tpvprflmch lafadlclgl
541 ylllvacida hsmgeyfnfa ydwqyglgck vagfltvfas hlsvftltvi tierwlaitq
601 amylnhrikl rpaalimlgg wiysmlmssm plfgisnyss tsiclpmenr dvydtiylia
661 ilgsngvafs iiavcyaqiy lslgretrqa hqnnpgelsv akkmallvft nfacwspiaf
721 fgltalagyp linvtkskil lvffyplnsc adpylyailt sqyrqdlftl lsklglcrqs
781 alkykdslsg qattrftihg siqrhssltc kmqtvmgaet qkmlknsedy v
```

Sechellia **XP_002041048.1**

```
  1 mekhpsmsqr lettcrprkg lkclsfefqc rlllhhlllt slsgrhcvya tsaaggalsa
 61 snchdihhgf dvypnptavs laqstdtplt aamprsawkc ccwnasnqae evecrcegdg
121 lnrvpqtltl piqrltiasa glprlrhtgl kvygstlldv aftdclqlel iqdgafanlt
181 llrtiyitna pkltflsrdv flgisdtvdi iriinsgltr vpdlghlpph nilqmidldn
241 nqitridsks ikvktaqlil tnneisyvdd safygskiak lslkenkkle mmhpnafdgi
301 idiaeldlss tslvglpsag lqniealyiq nthtlktips iynfrnlqra ylthsfhcca
361 fqfpsrhdpq rhaqrmleie kwrkqcksds gsrkerstld nsfsmpedfg sfggtddsst
421 ditpitfasf dymaddtmnk gtfhekiiln pgddssaelc gnftfrkpni ecypmpndln
481 pcedvmgyqw lrisvwivva lavvgnvavl tvilsirpes tpvprflmch lafadlclgl
541 ylllvacida hsmgeyfnfa ydwqyglgck vagfltvfas hlsvftltvi tierwlaitq
601 amylnhrikl rpaalimlgg wiysmlmssm plfgisnyss tsiclpmenr dvydtiylia
661 ilgsngvafs iiavcyaqiy lslgretrqa hqnnpgelsv akkmallvft nfacwspiaf
721 fgltalagyp linvtkskil lvffyplnsc adpylyailt sqyrqdlftl lsklglcrqs
781 alkykaslsg qattrftihg siqrhssltc kmqtlmgaet qkmlknsedy v
```

Erecta **XP_001979725.1**

```
1 mekhpslpps qtqtqrrdtt crskkglkcl sfefqcrlll hhllltslsg rhcvyamsas
 61 schdihhgfd vypnpnpttv slgqstdtpq aeamppsawk cccwnasnqv eevecrcegd
121 glnrvpqtlk lpiqrltias aglprlrhtg lkvygptlld vaftdclqle liqdgafanl
181 tllrtiyitn apkltflskd vflgisdtve iiriinsglt rvpdlghlpp hnilqmidld
241 nnqitridsk sikvktaqli lanneisyvd dsaffgskia klslkenkrl kmmhpnafdg
301 iidiaeldls stslvglpsa glqniealyi qnthtlktip siynfrnlqr aylthsfhcc
361 afqfpsrhdp qrhaqrmlei ekwrkqckse sgsrqerstl dsslsmpedf gtfsgtddsa
421 tditpitfvs pdymaddtmn kgtfhekivl npeddssael cgnftfrkpn iecypmpndl
481 npcedvmgyq wlrisvwivv alavvgnvav ltvilsirpe stpvprflmc hlafadlclg
541 lylllvacid ahsmgeyfnf aydwqyglgc kvagfltvfa shlsvftltv itierwlait
601 qamylnhrik lrpaalimlg gwiysmlmss lplfgisnys stsiclpmen rdvydtvyli
661 ailgcngvaf siiavcyaqi ylslgretrq ahqnnpgels vakkmallvf tnfacwspia
721 ffgltalagy plinvtkski llvffyplns cadpylyail tsqyrqdlft llsklglcrq
781 salkykdsls gqattrftih gsiqrhsslt ckmqtvmgae tqkmlknsed yv
```

Takahashi **XP_017013746.2**

```
1 mekhtslprk easessrkgl kclsleflfr llfhqllfts lcgphcvyat savgealgas
 61 nchdilhgfd vypnptagal gqstdspltv tlppsgwkcc cwnasnqmee vecrcegdgl
121 nrvpqtlklp iqrltiasag lprlrytglk vygptlldvt ftdclqleli qdgafanltl
181 lrtiyitnap kltflskdvf fgisdtveii riinsgltrv pdlshlpphn ilqmidldnn
241 qitridtksi kvktaqlila nndisyvdds affgskiakl slndnqkleh mhpnafdgii
301 diteldlsst slvglpssgl qtiealyiqn thtlktipsi ynfrnlqray lthsfhccaf
361 qfpsrhdpqr hakrmreiek wreqcksdsa srkerstsen gfempedfgs fgspdeattd
421 ivpikfasfd ymaddtlnkg tfhekitldp addmsaelcg nftfrkpnie cypmpndlnp
481 cedvmgyqwl risvwivval avvgnvavlt vilsirpest pvprflmchl afadlclgvy
541 lllvasidah smgeyfnyay dwqyglgckv agfltvfash lsvftltvit ierwlaitqa
601 mylnhriklr paalimlggw iysmfmsslp lfgisnysst siclpmenrd iydtvyliai
661 mvcngvafsi iavcyaqiyl slgretrqah hnspgelsva kkmallvftn facwspiaff
721 gltalagypl invtkskill vffyplnsca dpylyailts qyrqdlftll sklglcrqsa
781 lkykdslsgh attrftihgs iqrhgsltck mqtvmgatet qkmlkssedy v
```

Suzuki **XP_016932820.2**

```
1 mekhtsqypk espwrtkrgl kcltfefqfr lllhhlmlss lcgphcvyat savgealsas
 61 nchdihhgfd vypnpnpspn ltavtlgqst dtpsvtltlp psgwkcccwn asnqaeevec
121 rcegdglnrv pqtlklpiqr ltiasaglpr lrytglkvyg ptlldvaftd clqleliqdg
181 afanltllrt iyitnapklt flskdvffgi sgtveiirii nsgltrvpdl shlpphnilq
241 midldnnqit ridtksikvk taqlilannd isyvddsaff gskiaklsln dnlrlehmhp
301 nafdgidite ldlsstslva mpsvglqnie alyiqnthtl ktipsiynfr nlqraylths
```

361 fhccafqfps rhdpqrhaqr mleiekwrkq cksesvsrqe rstldnaytm pedfgtfest
421 desstdlvpi tfasfdymad dslnkgtfhe kitlnpeddm saelcgnftf rkpniecypm
481 pndlnpcedv mgyqwlrisv wivvalavvg nvavltvils irpestpvpr flmchlafad
541 lclgvylllv asidahsmge yfnyaydwqy glgckaagfl tvfashlsvf tltvitierw
601 laitqamyln hriklrpaal imlggwiysm fmsslplfgi snysstsicl pmenrdvydt
661 vyliaimvcn gvafsiiavc yaqiylslgr etrqahhnsp gelsvakkma llvftnfacw
721 spiaffglta lagyplinvt kskillvffy plnscadpyl yailtsqyrq dlftllsklg
781 lcrqsalkyk dslsghattr ftihgsiqrh ssltckmqtv mgtetqkmlk kseeyv

Biarmipes XP_016951482.1

1 mekhtsqcpk eapwrpkrgl kcltiefqfr lllhhlllss lcgphcvyat savgealsas
61 nchdihhgfd vypnpnlnpt ssangttvtl gqsrdtpsvp ltlppsgwkc ccwnggnqae
121 evecrcegdg lnrvpqtlkl plqrltiasa glprlrytgl kvygatlldv aftdclqlel
181 iqdgafanlt llrtiyitna pkltflskdv flgisgtvev iriinsgltr vpdlshlpph
241 nilqmidldn nqitridtks ikvktaqlil anndisyvdd safygskiak lslndnlrle
301 hmhpnafdgi iditeldlss tslvampsvg lqniealyiq nthtlktips iynfrnlqra
361 hlthsfhcca fqfpsrhdpq rhaermleie kwrkqcksds vtrkerstle ngftlpedfg
421 tfggtddsst dlvpitfasf dymaddtlik gtfhekiiln peddmsaelc gnftfrkpni
481 ecypmpndln pcedvmgyqw lrisvwivva lavvgnvavl tvilsirpes tpvprflmch
541 lafadlclgv ylllvasida hsmgeyfnya ydwqyglgck vagfltvfas hlsvftltvi
601 tierwlaitq amylnhrikl rpaalimlgg wiysmfmssl plfgisnyss tsiclpmenr
661 dgydtvylia imvcngvafs iiavcyaqiy lslgretrqa hqnspgelsv akkmallvft
721 nfacwspiaf fgltalagyp linvtkskil lvffyplnsc adpylyailt sqyrqdlftl
781 lsklglcrqs alkykdslsg hattrftihg siqrhgsltc kmqtvmgaes qkmlkntedy
841 v

Eugracilis XP_041675437.1

1 mekhtnlcrt estwrpkkgl kclsfeylfr lllhhlllts lsgphcvyat sdigealsas
61 nchdihhgfd vypnpnalsl gqstdtpltq tlppsawkcc cwnasnqmee vecrcegdgl
121 nrvpqtlklp iqrltiasag lprlrytglk vygptlldva ftdcqqleli qdgafsnltl
181 lrtiyitnap kltflskdvf lgisdtveii riinsgltrv pdlshlppnn ilqmidldnn
241 qitridtksi kvktaqlila nneisyidds affgskiakl slkdnlklem mhpkafdgii
301 diteldlsst slvglpsvgl qtiealyiqn thtlktipsi ynfrnlqray lthsfhccaf
361 qfpsrhdpqr haqrmleiek wrkqcksdsg srkerstldg ldnqpedfgs fggsyqsatd
421 ispitfssid ymaddtmnkg tfhekitlnp eddmsaelcg nftfrkpnie cypmpndlnp
481 cedvmgyqwl risvwivval avvgnvavlt vilsirpett pvprflmchl afadlclgvy
541 lllvasidah skgeyfnfay dwqyglgckv agfltvfash lsvftltvit ierwlaitqa
601 mylnhriklr paalimlggw iysmfmssmp lfgisnysst siclpmenrd vydtvylvai
661 mvcngvafsi iavcyakiyl slgretrqar hnnpgelsva kkmallvftn facwspiaff
721 gltalagypl invtkskill vffyplnsca dpylyailts qyrqdlftll sklglcrqsa
781 lkykdslsgh ptsrftihgs iqrhgsltck mqttmttetq kmlknsedyv

Ficusphelia XP_017047222.1

1 mkkhtnlhrk evtwrsrrgt kcltfeflfr llynhlllts mfgpdcvyat savgkalsas
61 nchdihhgfd vfpnpnptpa lnavdsnpnt ssnatamwlg qstdtpvtlt lppsawkccc
121 wnasnqneev ecrcegdgln rvpqtlklpl qrltiasagl prlrytglkv ygptlldvtf
181 tdclqleliq dgafanltll rtiyisnapk ltflskdvfa gisesveiir iinsgltrvp
241 dlghlpphni lqmidldnnq isridtksik vktaqlilan ndisyvddsa ffgskiakls
301 lqnnrklkqm hpnafdgiid iteldlssts lvglpsvglq niealyitnt htlktipsiy
361 nfrnlqrayl thsfhccafq fpsrhdpqrh aermreiekw reqcksdhgs rkersaldgf
421 eglpedfgtf gmpeqsgtdd tsityasfdy msddtlnkgt fhekitlnpe ddmtaelcgn
481 ftfrkpniec ypmpndlnpc edvmgyqwlr isvwivvala vvgnvavltv ilsirpestp
541 vprflmchla fadlclgvyl llvasidahs mgeyfnyayd wqyglgckva gfltvfashl
601 svftltviti ercmaitqam ylnhriklrp aaiimlcgwi ysmlmsslpl fgisnyssts
661 iclpmenrdv fdtmyliail gcngvafsii avcyaqiyls lgretrqarh nnpgelsvak
721 kmallvftnf acwspiaffg ltalagypli nvtkskillv ffyplnscad pylyailtsq
781 yrqdlftlls klglcrqsal kykdslsgqa ttrftihgsi qrhgsltckm qtvmgtetqk
841 mlkcgedyv

Elegans XP_017119258.1

1 mekpailark dvlrrsrkcs kglkclsfdf qfrlllhyll lsslsgphcv yatmagseal
61 sasnchdihh gfdvypnpta vsqgqstdtp ltltlppsgw kcccwnasnq neevecrcgg
121 dglnrvpqtl krpiqrltia saglprlrnt glkvygstll dvaftdclql eliqdgafan
181 ltllrtiyis napkltflsk dvffgisgsv eiiriinsgl trvpdlghlp phnilqmidl
241 dnnqitridt ksikvktaql ilanndisyv ddsaffgski aklslkdnpk lkelhpnafd
301 giiditeldl sstslvgmps vglqtiealy iqnthtlkti psiynfrnlq raylthsfhc

361 cafqfpsrhd pqrhaqrmle iqkwreqcks dhvsrkerst vdnlnsmpee fgsfgstdqs
421 atdmtsmtya sfdymsddtm nkgtfhekit lnpeddidel cgnftfrkpn iecypmpndl
481 npcedvmgyq wlrisvwivv alavvgnvav ltvilsirpe stpvprflmc hlafadlclg
541 vyllfvasid ahsmgeyfnf aydwqyglgc kvagfltvfa shlsvftltv itierwlait
601 qamylthrik lrqasiimlg gwiysvlmss lplfgisnys stsiclpmei rdgfdtayli
661 ailacngvaf tiiavcyaqi ylslgretrq arqnspgels vakkmallvf tnfacwspia
721 ffgltalagf plinvtkski llvffyplns cadpylyail tsqyrqdlft llsklglcrq
781 salkdkdgss trgttrytin gsiqrhaslt ckmqtvvgae tqkmlknged yv

Rhopaloa XP_016976568.1

1 mekytilark rarrpskril kclsleflyr lllhhlllts lcdhhyvyat savgepisas
61 dchdihhgfd vfpnhtalpl gqstdtplts tlppsgwkcc cwkssnqnee vecrcegdgl
121 nrvpqslklp iqrltiasag lprvrftglk vygsslldva fidclqleli qdgafanlal
181 lrtiyisnap kltflskdvf sgisgsveii riinsgltrv pdlghlpphn ilqmidldnn
241 qitridtksi nvktaqlila nndisyvdds affgskiakl slndnpklke lhpnafngii
301 ditaldlsst sligmpsagl qtiealyiqn thtlktipsi ynfrnlqray lthsfhccaf
361 qfpsrhdper haqrmleiek wrkqcksdqv srkersiadn lnsvpeefgs fgmtdqsldy
421 msddtmnkgt fhekitlnpe ddidelcgnf tfrkpniecy pmpndlnpce dvmgyqwlri
481 svwivvalav vgnvavltvi lsirpestpv prflmchlaf adlclgvyll lvasidahsm
541 gayfnyaydw qyglgckvag fltvfaslls vftltvitie rwlaitqamy lthriklrpa
601 sfimlggwiy silmsslplf gisnysstsi clpmeirdvf dtvyliaila cngvafsiia
661 vcyaqiylsl gretrqsrqn npgemsvakk mallvftnfa cwspiaffgl talagfplin
721 vtkskillvf fyplnscadp ylyalltsqy rqdlftllsk lglcrqsalk ykdsssahat
781 srftihnsih rhgsltskmq tvisaetqkm lknsedyv

Kikkawei KAH8340697.1

1 mgrrqtnwvr rkgrgqpkta ltclsigflf rflfvyhlll aplcgphcvy amlaaegpsd
61 gsnchdihhg fdvpsslnav teaqggdapm avtltpapsg wkcccwkapn qgeevecrce
121 gdaltrvpqt lqlpiqrlti asaglprlrf tglkvygptl ldvaftdclk leliqdgafa
181 nltllrtiyi snapkltfls kdvfagiset veiiriinsg ltsvpdlghl pphnilqmid
241 ldnnqitrid tksinvktaq filanndisy vddsaffgsk iaklslkdnr kltdmhpnaf
301 ngiidiaeld lsstslvglp svglqtieal yimnthtlkt ipsiynfrnl qraylthsfh
361 ccafqfpsrh dprrhaqrml eiekwreqcn kgsrkersll dnfeaqpedf gsfgstepsv
421 teitpypyas vdymadstnl gyfheqitin pdddqsaefc gnftfrkpni ecypmpndln
481 pcedvmgyqw lrisvwivva lavvgnvavl tvilsirpes tpvprflmch lafadlclgv
541 ylllvasida hsmgeyfnya ydwqyglgck aagfltvfas hlsvftltvi tierwlaitq
601 amylnhrikl rqaalimlgg wlysmfmssl plfgisnyss tsiclpmenr dafdtmylia
661 ilgcngvafs iiavcyaqiy lslgretrqa rqnnpgelsv akkmallvft nfacwspiaf
721 fgltalagfp linvtkskil lvffyplnsc adpylyailt sqyrqdlftl lskiglcrqn
781 alnykhsssa qattrftihr hssltckmqa aisveaqkml kngedyv

Bipectinata XP_017105030.2

1 mkcvlpkfvf rlllhhlllt klcwlhgvya tsaschdsyn gfnvfpngss dlrpdpnptd
61 vssgqapppt ltlppwkccc wnatnqseea ecrcegealt rvpqtltmam qrltiatagl
121 prlratglkv yaqtlmdvaf tdclqleliq dgafanlkll rtiyianapk ltflskdvff
181 gisdtveiir iinsgltrvp dlthlppyni lqmidldnnq itridaksik vktaqlilan
241 ndisyvddsa fcgskiakls lkenrklkel htnafhgiid iteldlssts ivempsaglq
301 tiealyilnt htlktipsiy nfrnlqrayl thsfhccafq fpsrhdplrh aqrmdeiekw
361 rtqckgeris rkersilnnl ksqsdgygif gseasptdvv stqfasvdym adaamnlgyf
421 heeitlnpdd eqfaefcgnf tfrkpnvecy pmpnalnpce dvmgyqwlri svwvvvalav
481 vgnvavltvn lsirpeitpv arflmchlaf adlclglylf lvasidahsm geyfnyaydw
541 qyglgckvag fltvfashls vftltlitie rwvaitqamy lnkrirlrsa siimlggwiy
601 smvmsslplf gisnysstsi clpmevrdsf dtvyliailg cngvaffiia vcyakiylsl
661 gretrharqn npgelsvakk mallvftnfa cwspiaffgl talagfplin vtnskillvf
721 fyplnscadp ylyailtaqy rqdlytllsk lglcrknavn skdnssgmgt trftihrhss
781 ltcrippale vetqkmltcn edyv

Anannassae XP_001964371.3

1 mkcvlpkfvy rlllhhlllt ricwphgvya tsavchdshn gfnvspdgns dlspdrnptt
61 mssgqapppt ltlppwkccc wnatnqseev ecrcegealt rvpqtltmam qrltiasagl
121 prlratglkv yaqtlldvaf tdclqleliq dgafanlkll rtiyianapk lsflskdvfs
181 gisdtveiir iinsgltsvp dlthlppyni lqmidldnnq itridsksik vktaqlilan
241 ndisyvddsa ffgskiakls lkenrklkel htnafhgiid iteldlssts ivempsaglq
301 tiealyilnt htlktipsiy nfrnlqrayl thsfhccafq fpsrhdplrh aqrmleiekw
361 rtqcngerns rrerniakki esqqddlgtl ggsealsadv lstpfssvdy madaamnlgy
421 fheeitinpd deqfaefcgn ftfrkpsvec ypmpdalnpc edvmgyqwlr isvwvvvala
481 vvgnvavltv nlsirpeitp varflmchla fadlclglyl flvasidahs mgeyfnyayd

541 wqyglgckva gfltvfashl svftltliti erwvaitqam ylnkrirlrs asiimlggwi
601 ysmvmsslpl fgisnyssts iclpmevrdt fdtvyligil gcngvaffii avcyakiyfs
661 lgretrharq nnpgelsvak kmsllvftnf acwspiaffg ltalagypli nvtnskillv
721 ffyplnscad pylyailtaq yrqdlytlls klglcrtnav nskdnssgmg ttrftihrhs
781 sltcrippal evetqkmlty seeyv

Mojavensis XP_001998425.1

1 mkctpiiarf dfsavillsl vycsqqastn cydnhdgfna iinnlprdag naidtldtla
61 qappmttnpp vdasvwkccc weatnhnefe crcegealtr vpqtlklplq rltiasaglp
121 rlrsmglkvy aptlldvafi dclqletiqs gafsnltvlr aiyisnapkl sylaknvfeg
181 isdtieiiri insglktvpd lgdlppynil qmidldnnqi sridsksiqv ktaqlvlann
241 eitfiddsaf lgskiaklsl ndnhklteih pnafngiidm teldlsstsl vrlpsaglqt
301 levlyianth tlktipsiyn fqnlqrahlt hsfhccafqf psrhdpqrha qrmqevekwr
361 dqcknnrdvh tnyerslsms nltplanvse vqpsgstinn llaestpnty aymadstlnn
421 igifheeiti npdddqlaey cgnftfrkph vecypmpnal npcedvmgyq wlriavwivv
481 alaivgnvav ltvilsikse spsvprflic hlafadlclg lyllliasid ahsmgeyfny
541 afdwqyglgc kiagfltvfa shlsvftltv itierwfait hamylnkrik lrpaavimlm
601 gwiysivmss lpllgisnys stsiclpmek rdiydsiylv lilgcnflaf tiiaicysqi
661 ylslgketrr arqnhlgems vakkmallvf infscgapia ffgltalagy plinvtkski
721 llvffyplns cadpylyail tsqyrqdllt llskfglcrq ralkykhsds mhgtshytir
781 gsierehsvg qkcqkivaae aqnmlrnned yv

Virilism XP_032289845.1

1 mkcmviisql dfltfillsl ahgtrqantn chdnnngfnt vfnnlsidng netdmsvimt
61 qaptmstnpt sdasvwkccc weatnqnefe crcegealtr vpqtlklpll rltiasaglp
121 rlrsmglkvy attlldvafi dclqleaiqn gafsnltflr tiyisnapkl tylpknvfeg
181 isdtieiiri insgltsvpd fgylppnnil qmidldnnqi sridsksiqv ktaqfvlann
241 dihfiddsaf lgskiaklsl kdnrrltdvh pnafygiidi teldlsstsl vslpsaglqt
301 vevlyiinth tlktipsiyn fqnlqrahlt hsfhccafqf psrhdpkrha erlqelqkwr
361 eqcnverdly rdvdkklkkn tsvkdtagvh ytnqsgtatd nlltdaasns ydymadstmn
421 nigifheqit inpddnqlae ycgnftfrnp diqcfpmpna lnpcedvmgy qwlrisvwiv
481 valaivgnla vltvtlsiks espsvprfli chlafadlcl glyllliasi dahsmgeyfn
541 yafdwqyglg ckiagfltvf ashlsiftlt iitierwfai thamylnkri tlrqaagiml
601 tgwiysiims slplfgisny sstsiclpme irdiydsiyl ililgcnfva ftiiaicysq
661 iylslgqetr rarrnnpgem svakkmlllv finftcgapi affgltalag cplinvtksk
721 illvffypln scadpylyai ltsqyqqdll tflsklgicr qnalkykhsd slhgtshyti
781 rgsieqqssl cqkpqqegaa etqtmlknne dyv

Grimshawi EDV91130.1

1 mkwttfglqf diltlfvlwv lvccahqast ncydsssgfn tnpnalpaed gnetyttdtt
61 pmptadasvw kcccweatnq nlfpedefec rcegealtrv pqtlklplqr ltiasaglpr
121 lrsiglkvya stlldvaftd cpqletieng afsnltilrt iyisnapklk ylskdvfegi
181 snkidiirii nsgltsvpdl ghlppntilq midldnnqis riesksiqvk taqfvlannd
241 ityiddsafl dskiaklslk qnrrlseihp nafngiidmt eldlsstslv rlpaaglqti
301 evlyimntht lktipsiynf qnlqkahlth sfhccafkfp srhdpqrhkk heeelrklqe
361 qcksdrdmhn nvatniiipt ksmedavgmp ngkgnwgien gdwnnnelid spealpnmdd
421 ymadatmnnl gvfhteitin pddnqmtefc gnftfrkpni ecypvpnaln pcedvmgyew
481 lriavwivva ltivgnvavl tvilsikses psvprflich lafadlclgl yllliasida
541 hsmgqyfnya ydwqygfgck vagfltvfas hlsvftltvi tierwfaith amylnkrikl
601 grasvimitg wlyaitmssl plfgisnyss tsiclpmeqr diydsvyllm ilgsnfiaft
661 iiaicysqiy lslgqetrna rqnnpgemsi akkmallvfi nfscgapiaf fgltalagyp
721 linvtkskil lvffyplnsc adpylyailt sqyrqdlfll fsklgicrks vmkykysdsm
781 phtshftirn sieqpnafph kapngagaet qkmlinnedy v

Serrata XP_020813515.1

1 mgrrrthwvr kgdrrpvktg ikclsigyll rllfvhhlll gsfcgshrvy amlaaegqsv
61 rsnshdvhhg fdvpgrqpvs dnqvrgapit vilpkvtsrw kcfcsktpnq seelecrceg
121 dsltrvpqtl qlpiqrltia saglprlrst glkvygptll dvaftdclkl eliqdgafan
181 ltmlrtiyis napkltflsk dvfagisetv eviriinsgl trvpdlghlp phnilqmidl
241 dnnqitridt ksinvktaql ilanndisyv ddsaffgski aklslkdnwk ltemhpeafn
301 giiditeldl sstslvglps vglqtiealy imnthtlkti psiynfrnlq raylthsfhc
361 cafqlpsrhd prrhalrmle iekwreqcnk csrkeralld yleaqpedfg sfgstepsmt
421 entpfpiasi dymadstnlg yfheqitinp ddkqsaefcg nftfrkpnie cypmpndlnp
481 cedvmgyqwl risvwivval savgnvavlt vilsirpess pvprflmchl afadlclgvy
541 lllvasidah sigeyfnyay dwqyglgckv agfltvfash lsvftltvit ierwlaitqa
601 mylnhrikmr qaalimlggw lysmvmsslp lfgisnysst siclpmenrd afdtmyliai
661 lgcngvafsi iavcyaqiyl slgretrqah qnnpgelsva kkmallvftn facwspiaff

721 gltalagfpl invtkskill vffyplnsca dpylyailts qyrqdlltll sklglcrqna
781 lnynhsssap attrftihrh ssltckmqtv lnaeaqkmlk ngedyi
