# Supplementary material for: The incidence of candidate binding sites for β-arrestin in Drosophila neuropeptide GPCRs
Source: PLoS One. 2022 Nov 1;17(11):e0275410. doi: 10.1371/journal.pone.0275410 (PMC9624432; doi:10.1371/journal.pone.0275410)
Supplement: S16 Text — (PDF) [file pone.0275410.s020.pdf]

S16. Text **Multi-species analysis of Hector**
Supporting Figure 18\9

CLUSTAL Line-ups; Genbank Reference IDs below
Predicted TM domains in YELLOW
BBS sequences in RED
missing mauritania

CLUSTAL

```
bipectinata      -----------mgglsasvtep-----------dmessraqdapqpqdnlriflkhlyae    38
anannasae        -----------mgglsasvtep-----------dme----qeapqpqdnlriflkhlyae    34
kikkawei         mtsi--eeapptmgtnasdsdsdsgsenvamatatasqqsytqpqaqdnlriflrhlyae    58
seratta          ----------------------------mamakasgsqqsssqtqaqdnlriflrhlyae    32
eugracilis       mtrvthietptmvgttasese----senv---------emasqaqtqdnllvflthlyae    47
ficusphelia      ---------mgt-gasssdsg----sekl---------etaaqaqtqdnlriflkhlyae    37
erecta           mtavthteqptm-attssdse----plnl---------dvasqaqtqdnlriflkhlyae    46
melanogaster     -----------m-attssdse----sqnv---------dvasqaqtqdnlriflkhlyae    35
simulans         -----------m-attssdsd----venv---------dvasqaqtqdnlriflkhlyae    35
sechellia        -----------m-attssdsd----venv---------dvasqaqtqdnlriflrhlyae    35
elegans          mttvthfdtptm-gtvasdst----menv---------etatqsqtqdnlriflkhlyae    46
rhopaloa         -----------m-gtvasdsv----penv---------etatqsqtqdnlriflkhlyae    35
takahashi        mtrvshtepptm-gaaasdsq----senv---------eaasqsmqdnlriflkhlyae    46
Suzuki           mtrvspteaptm-gtaasvse----senv---------evasqtqtqdnlriflkhlyae    46
biarmipes        mtraspieaptm-gtaasese----senv---------eaasqtqtqdnlriflkhlyae    46
grimshawi        ---------------------------------mtasasasmspqpqdnlltflwhlyae    27
virilism         -----------mallraaltma--taayfemtvsga-----mppqpqdnlrtflkhlyae    42
mojavensis       -----------mallraalamt--taayietamtasltkpqprpqpqdnlriflrhlyve    47
                                                             * ****  ** ***.*

bipectinata      cvyryqndtyddpste------satdyesdfpenfspvprylenaalnegsidmrnvdek    92
anannasae        cvyryqndtyddpmte------tatdyesdlpenfspvprylenaalnegsidmrnvdek    88
kikkawei         cvyryqndtyddpsvsv-vgeaaatdydldlpenfspvprylenavlnegaidmrnvdve    117
seratta          cvyryqndtyddpsvsv-dga-aatdyeselpenfspvprylenavlnegaidmrnvdve    90
eugracilis       cvhryqnvtndtedsslslgaatdsend--lkgnfstvprylenaalnegtidmrnvdek    105
ficusphelia      cvyryqnvtyeaddpsvplgaateydvdanapdnfspvpryledavlnegaidmrhvdee    97
erecta           cvfryqnvtydtgdpsfslgpatdydsd--lpenfspvprylenaamnegvidmrsvdee    104
melanogaster     cvfryqnvtydtddpsfslgpatdydsd--lpenfspvprylenaamnegvidmrnvdee    93
simulans         cvfryqnvtydtddpsfslgpatdydsd--lpenfspvprylenaamnegvidmrnvdee    93
sechellia        cvfryqnvtydtddpsfslgpatdydsd--lpenfspvprylenaamnegvidmsnvdek    93
elegans          cvyryknvtydaadpsvslvaatdyesd--ltenfspvpryledavlnegaidmrnvdee    104
rhopaloa         cvyryqndtydaddpsvslgaatdydld--lpesfspvpryledavlnegaidmrnvdee    93
takahashi        cvyryqnvtydtedpsvplgaatdydfd--lpenfspvprylenaalnegaidmrnvdav    104
Suzuki           cvyryqnvtydtddpsvslgaatdydfd--lpenfspvprylenaamnegaidmrnvdae    104
biarmipes        cvyryqnvtydtddpsvslgaatdydfd--lpenfspvprylenaalnegaidmrnvdae    104
grimshawi        cvyryqndtspgqqat------dpgdgniqllepytrmpnylemavlnegtidmqdvnee    81
virilism         cvyryqndtataqlat------epddggl-lletytmipryleqavlnegtidmqdvdee    95
mojavensis       cvyryqndtakgda-k------dsgdeel-mlatfttvpryleqavlnegtidmqdvdee    99
                 **.**:* *                :        :: :*.*** *.:*** ***  *:

bipectinata      kaereelfatiltatmatnqnqddpagqrspgsns-sssrrlfcpldfdgylcwprtpag    151
anannasae        qaekeelfatiltatmatnqnqdepagqrssgnssssgskrlfcpldfdgylcwprtpag    148
kikkawei         qaekeellatvlsgtmatnqqkqshqatt------tttgerlfcplnfdgylcwprtpag    171
seratta          laekeellatvlsvtmatnqqesehhevt--ttnnrsagerlfcplnfdgylcwprtpag    148
eugracilis       laeqeelmatvssatmatnqr-----------------drrlfcpldfdgylcwprtpag    148
ficusphelia      aaekeelmatvftatmatnqr-----------------eerlfcplnfdgylcwprtpag    140
erecta           laekeelmatvvsatmatnqk-----------------ehrlfcplnfdgylcwprtpag    147
melanogaster     laekeelmatvvsatmatnqk-----------------enrlfcplnfdgylcwprtpag    136
simulans         laekeelmatvvsatmatnqk-----------------esrlfcplnfdgylcwprtpag    136
sechellia        laekeelmatvvsatmatnqk-----------------esrlfcplnfdgylcwprtpag    136
elegans          laekeelmatvlsatmatnqq-----------------epllfcplnfdgylcwprtpag    147
rhopaloa         laekeelmatvlsatmatnqrp----------------qqrlfcplnfdgylcwprtpag    137
takahashi        laekeelmatvvsatmatnqrqaeeg---------geeerrlfcplnfdgylcwprtpag    155
Suzuki           laekeelvatvvsatmatnqr----------------eeprlfcplnfdgylcwprtpag    148
biarmipes        qaekeelvatvvsatmatnqr----------------aeprlfcplnfdgylcwprtpag    148
grimshawi        asslnelyatvlsatmapnqhsiqyngnnsna-nttremeilycpvnfdgylcwprtpag    140
virilism         aasenelyatvlsatmatnhhs---------h-n-tsemetlycpvnfdgylcwprtpag    144
mojavensis       aasenelyatihsmtmetnqhs---------r-natsemetlycpvnfdgylcwprtpag    149
```

```
                      :. :** **: : **  *:.                    *:**::*************

bipectinata      TVLSQYCPDFVEGFNSKFLAHKTCQENGSWYRHPETNKTWSNYTNCVDYVDFEFRQFINE     211
anannasae        TVLSQYCPDFVEGFNSKFLAHKTCQENGSWYRHPESNKTWSNYTNCVDYVDLEFRQFINE     208
kikkawei         TVLSQYCPDFVEGFNSKFLAHKTCLENGSWFRHPMTNRTWSNYTNCVDYEDLEFRQFINE     231
seratta          TVLSQYCPDFVEGFNSKFLAHKTCLENGSWFRHPMSNQTWSNYTNCVDYEDLEFRQFINE     208
eugracilis       TVLSQYCPDFVEGFNKKFLAHKTCLENGSWFRHPATNQTWSNYTNCVDHEDLEFRKFINE     208
ficusphelia      TVLSQYCPDFVEGFNSKFLAHKTCLENGSWFRHPVSNQTWSNYTSCVDYEDLEFRQFINE     200
erecta           TVLSQYCPDFVEGFNRKFLAHKTCLENGSWYRHPVSNQTWSNYTNCVDYKDLEFRQFINE     207
melanogaster     TVLSQYCPDFVEGFNRKFLAHKTCLENGSWYRHPVSNQTWSNYTNCVDYEDLEFRQFINE     196
simulans         TVLSQYCPDFVEGFNRKFLAHKTCLENGSWYRHPVSNQTWSNYTNCVDYEDLEFRQFINE     196
sechellia        TVLSQYCPDFVEGFNRKFLAHKTCLENGSWYRHPVSNQTWSNYTNCVDYEDLEFRQFINE     196
elegans          TVLSQYCPDFVEGFNSKFLAHKTCLENGSWFRHPVSNQTWSNYTNCVDYEDLEFRQFINE     207
rhopaloa         TVLSQYCPEFVEGFNSKFLAHKTCLENGSWFRHPVSNQTWSNYTNCVDYEDLEFRQFINE     197
takahashi        TVLSQYCPDFVEGFNSKFLAHKTCLENGSWFRHPVSNQTWSNYTNCVDYEDLEFRQFINE     215
Suzuki           TVLSQYCPDFVEGFNRKFLAHKTCLENGSWFRHPESNQTWSNYTNCVDYEDLEFRQFVNE     208
biarmipes        TVLSQYCPDFVEGFNRKFLAHKTCLENGSWFRHPESNQTWSNYTNCVDYEDLEFRQFVNE     208
grimshawi        TVLSQYCPDFVEGFNTKFLAHKTCMETGSWFRHPVSNQTWSNYTNCVDYEDLQFRQIVNE     200
virilism         TVLSQYCPDFVEGFNSKFLAHKTCLETGSWFRHPVSNQTWSNYTNCVDYEDFQFRQFVNE     204
mojavensis       TVLSQYCPDFVEGFSSKFLAHKTCLENGTWYRHPVSNQTWSNYTNCVDYDDFQFRQFVNE     209
                 ********:*****. ******** *.*:*:*** :*:******.***: *::**:::**

bipectinata      LYVKGYALSLLALLISIIIFLGFKSLRCTRIRIHVHLFASLACTCVAWILWYRLVVEQPE     271
anannasae        LYVKGYAISLLALLISIIIFLGFKSLRCTRIRIHVHLFASLACTCVAWILWYRLVVERPE     268
kikkawei         LYVKGYALSLLALLVSIIIFLGFKSLRCTRIRIHVHLFASLAFTCVAWILWYRLVVERPE     291
seratta          LYVKGYALSLLALLVSILIFLGFKSLRCTRIRIHVHLFASLAFTCVAWILWYRLVVERPE     268
eugracilis       LYVKGYALSLLALLISIIIFLGFKSLRCTRIRIHVHLFASLACTCVAWILWYRLVVERHE     268
ficusphelia      LYVKGYALSLLALLISIIIFLGFKSLRCTRIRIHVHLFASLACTCVAWILWYRLVVERPE     260
erecta           LYVKGYALSLLALLISIIIFLGFKSLRCTRIRIHVHLFASLACTCVAWILWYRLVVERSE     267
melanogaster     LYVKGYALSLLALLISIIIFLGFKSLRCTRIRIHVHLFASLACTCVAWILWYRLVVERSE     256
simulans         LYVKGYALSLLALLISIIIFLGFKSLRCTRIRIHVHLFASLACTCVAWILWYRLVVERSE     256
sechellia        LYVKGYALSLLALLISIIIFLGFKSLRCTRIRIHVHLFASLACTCVAWILWYRLVVERSE     256
elegans          LYVKGYALSLLALLISIIIFLGFKSLRCTRIRIHVHLFASLACTCVAWILWYRLVVERNE     267
rhopaloa         LYVKGYALSLLALLISIIIFLGFKSLRCTRIRIHVHLFASLACTCVAWILWYRLVVERNE     257
takahashi        LYVKGYTLSLLALLISIIIFLGFKSLRCTRIRIHVHLFASLACTCVAWILWYRLVVERGE     275
Suzuki           LYVRGYALSLLALLISIIIFLGFKSLRCTRIRIHVHLFASLACTCVAWILWYRLVVERPE     268
biarmipes        LYVRGYALSLLALLISIIIFLGFKSLRCTRIRIHVHLFASLACTCVAWILWYRLVVERPE     268
grimshawi        LYVKGYALSLLALLISIIIFVGFKSLRCNRIRIHVHLFASLACTCLTWILWYRLVVEHSE     260
virilism         LYVKGYALSLLALFISIVIFLGFKSLRCTRIRIHVHLFASLACTCIAWILWYRLVVEHTE     264
mojavensis       LYVKGYALSLLALIISIVIFLGFKSLRCTRIRIHVHLFGSLACTCIAWILWYRLVVEQTD     269
                 ***:**::*****::**:**:*******.*********.*** **::**********: :

bipectinata      ITAENPLWCIVLHLVVHYFMLVNYFWMFCEGLHLHLVLVVVFVKDTIVMRWFIIISWLSP     331
anannasae        ITAENPLWCILLHLVVHYFMLVNYFWMFCEGLHLHLVLVVVFVKDTIVMRWFIIISWLSP     328
kikkawei         IIADNPLWCIGLHLVVHYFMLVNYFWMFCEGLHLHLVLVVVFVKDTIVMRWFIIISWFSP     351
seratta          IIADNPLWCIGLHLVVHYFMLVNYFWMFCEGLHLHLVLVVVFVKDTIVMRWFIIISWFSP     328
eugracilis       TITENPLWCIGLHLVVHYFMLVNYFWMFCEGLHLHLVLVVVFVKDTIVMRWFIVISWFSP     328
ficusphelia      TIAENQLWCIGLHLVVHYFMLVNYFWMFCEGLHLHLVLVVVFVKDTIVMRWFIVISWFSP     320
erecta           TIAENPLWCIGLHLVVHYFMLVNYFWMFCEGLHLHLVLVVVFVKDAIVMRWFILISWFLP     327
melanogaster     TIAENPLWCIGLHLVVHYFMLVNYFWMFCEGLHLHLVLVVVFVKDTIVMRWFIVISWFSP     316
simulans         TIAENPLWCIGLHLVVHYFMLVNYFWMFCEGLHLHLVLVVVFVKDTIVMRWFIVISWFSP     316
sechellia        TIAENPLWCIGLHLVVHYFMLVNYFWMFCEGLHLHLVLVVVFVKDTIVMRWFIVISWFSP     316
elegans          TIAENPLWCIGLHLVVHYFMLVNYFWMFCEGLHLHLVLVVVFVKDTIVMRWFIVISWLSP     327
rhopaloa         TIAENPLWCSGLHLVVHYFMLVNYFWMFCEGLHLHLVLVVVFVKDTIVMRWFIVISWLSP     317
takahashi        AIAENPLWCIGLHLVVHYFMLVNYFWMFCEGLHLHLVLVVVFVKDTIVMRWFIVISWFSP     335
Suzuki           TIADNPLWCIGLHLVVHYFMLVNYFWMFCEGLHLHLVLVVVFVKDTIVMRWFMVISWLSP     328
biarmipes        TIADNPLWCIGLHLVVHYFMLVNYFWMFCEGLHLHLVLVVVFVKDTIVMRWFMVISWLSP     328
grimshawi        RIAENPNWCIALHLVVHYFMLVNYFWMFCEGLHLHLVLVVVFVKDTIVLRWFKFFSWLSP     320
virilism         QIAENPPWCIALHLVVHYFMLVNYFWMFCEGLHLHLVLVVVFVKDTIVMRWFKLLSWLLP     324
mojavensis       QIAENPPWCIALHLVVHYFMLVNYFWMFCEGLHLHLVLVVVFVKDTIVMRWFKLLSWLSP     329
                   ::*  **  **********************************:**:*** .:**: *

bipectinata      VHIAVVYGLARHFSSPDNEHCWINDSLYLWIFSVPITLSLLASFIFLINVLRVIVRKLHP     391
anannasae        VHFAVVYGLARHFSSPDNEHCWINDSLYLWIFSVPITLSLLASFIFLINVLRVIVRKLHP     388
kikkawei         IHFAIVYGLARHFSDSDNEHCWINDSLYLWIFSVPITLSLVASFIFLINVLRVIVRKLHP     411
seratta          IHFAIVYGLSRHFSDSDNEHCWINDSLYLWIFSVPITLSLLASFIFLINVLRVIVRKLHP     388
eugracilis       IPIAAVYGLARHFSNPDNKHCWINDSLYLWMFSVPITLSLLASFIFLINVLRVIVRKLHP     388
ficusphelia      IPIAIVYGLARHFSSPDNKHCWINDSLYLWIFSVPITLSLLASFIFLINVLRVIVRKLHP     380
erecta           IHITILYGLARHFSTPDNKHCWITDSLYLWIFSVPITLSLLASFIFLINVLRVIVRKLHP     387
melanogaster     IPIAIVYGLARHFSSPDNKHCWITDSLYLWIFSVPITLSLLASFIFLINVLRVIVRKLHP     376
simulans         IPIAIVYGLARHFSSPDNKHCWITDSLYLWIFSVPITLSLLASFIFLINVLRVIVRKLHP     376
sechellia        IPIAIVYGLARHFSSPDNKHCWITDSLYLWIFSVPITLSLLASFIFLINVLRVIVRKLHP     376
elegans          IPVAIVYGLARHFSGPDNKHCWINDSLYLWIFSVPITLSLLASFIFLINVLRVIVRKLHP     387
```

```
rhopaloa        IPIAVVYGLARHFTSPDNKHCWINDSLYLWIFSVPITLSLLASFIFLINVLRVIVRKLHP     377
takahashi       IPVAIVYGLARHFSSPDNKHCWINDSLYLWIFSVPITLSLLASFIFLINVLRVIVRKLHP     395
Suzuki          IPIAVVYGLARHFSSPDNKHCWINDSLYLWIFSVPITLSLLASFIFLINVLRVIVRKLHP     388
biarmipes       IPIAIVYGLARHFSSPDNKHCWINDSLYLWIFSVPITLSLLASFIFLINVLRVIVRKLHP     388
grimshawi       LLFIIPYGVVRHFSANDNKHCWISESFYLWILSVPITLSLLASFIFLINVLRVIVRKLHP     380
virilism        LLFVLPYGVARHFSANDNAHCWMNDSFYLWIFSVPITLSLLASFIFLINVLRVIVRKLHP     384
mojavensis      LVFVLPYGVARHLSVNDNEHCWINDSLYLWIFSVPITLSLLASFIFLINVLRVIVRKLHP     389

bipectinata     QSAQPAPLAIRKAVRATIILVPLFGLQHFLLPYRPDAGTQLDRFYQLLSVVLVSLQGFVV     451
anannasae       QSAQPAPLAIRKAVRATIILVPLFGLQHFLLPYRPDAGTQLDRFYQLLSVVLVSLQGFVV     448
kikkawei        QSAQPAPLAIRKAVRATIILVPLFGLQHFLLPYRPDAGTQLDRFYQLLSVVLVSLQGFVV     471
seratta         QSAQPAPLAIRKAVRATIILVPLFGLQHFLLPYRPDAGTQLDRFYQLLSVVLVSLQGFVV     448
eugracilis      QSAQPAPLAIRKAVRATIILVPLFGLQHFLLPYRPDAGSQLDRFYQMLSVALVSPQGFVV     448
ficusphelia     QSAQPAPLAIRKAVRATIILVPLFGLQHFLLPYRPDAGTQLDRFYQLLSVVLVSLQGFVV     440
erecta          QSAQPAPLAIRKAVRATIILVPLFGLQHFLLPYRPDAGTQLDHFYQMLSVVLVSLQGFVV     447
melanogaster    QSAQPAPLAIRKAVRATIILVPLFGLQHFLLPYRPDAGTQLDHFYQMLSVVLVSLQGFVV     436
simulans        QSAQPAPLAIRKAVRATIILVPLFGLQHFLLPYRPDAGTQLDHFYQMLSVVLVSLQGFVV     436
sechellia       QSAQPAPLAIRKAVRATIILVPLFGLQHFLLPYRPDAGTQLDHFYQMLSVVLVSLQGFVV     436
elegans         QSAQPAPLAIRKAVRATIILVPLFGLQHFLLPYRPDAGTQLDRFYQLLSVVLVSLQGFVV     447
rhopaloa        QSAQPAPLAIRKAVRATIILVPLFGLQHFLLPYRPDAGTRLDRFYQLLSVVLVSLQGLVV     437
takahashi       QSAQPAPLAIRKAVRATIILVPLFGLQHFLLPYRPDAGTQLDHFYQMLSVVLVSLQGFVV     455
Suzuki          QSAQPAPLAIRKAVRATIILVPLFGLQHFLLPYRPDAGTQLDHFYQMLSVILVSLQGFVV     448
biarmipes       QSAQPAPLAIRKAVRATIILVPLFGLQHFLLPYRPDAGTQLDHFYQMLSVILVSLQGFVV     448
grimshawi       QSAHPAPMAIRKAVRATIILVPLFGLQHFLLPYRPEAGTKLDRFYQLMSVVLVSLQGFVV     440
virilism        QSAQPAPLAIRKAVRATIILVPLFGLQHFLLPYRPDAGTQLDRFYQLLSVVLVSLQGFVV     444
mojavensis      QSAQPAPLAIRKAVRATIILVPLFGLQHFLLPYRPEAGTQLDRFYQLLSVVLVSLQGFVV     449

bipectinata     SFLFCFANHDVLFAVRTLLNKLLPSLVSPPPAGSNTGQMATTTPSRELGV     501
anannasae       SFLFCFANHDVLFAVRTLLNKLMPSLVSPPPAGSNTGQMATTTPSRELGV     498
kikkawei        SFLFCFANHDVTFAIRTLLNKWLPSLVAAPPAGSNTGQMATTTPSRELGV     521
seratta         SFLFCFANHDVTFAIRTLLNKWLPSVVAPPPAGSNTGQMATTTPSRELGV     498
eugracilis      SFLFCFANHDVTFAIRTLLNKWLPSLVTPPPAGSNTGQMATTTPSRELGV     498
ficusphelia     SFLFCFVNHDVTFAIRTLLNKMMPTLVTAPPAGSNTGQMATTTPSRELGV     490
erecta          SFLFCFANHDVTFAVRTLLNKWLPSLVIAPPAGSNTGQMATTTPSRELGV     497
melanogaster    SFLFCFANHDVTFAIRTLLNKLLPSLVTPPPAGSNTGQMATTTPSRELGV     486
simulans        SFLFCFANHDVTFAIRTLLNKLLPSLVTPPPAGSNTGQMATTTPSRELGV     486
sechellia       SFLFCFANHDVTFAIRTLLNKLLPSLVTPPPAGSNTGQMATTTPSRELGV     486
elegans         SFLFCFANHDVTFAIRTLLNKWLPRLVTAPPAGSNTGQMATTTPSRELGV     497
rhopaloa        SFLFCFANHDVTFAIRTLLNKWLPRLVTAPPAGSNTGQMATTTPSRELGV     487
takahashi       SFLFCFANHDVTFAIRTLLNKWLPSLVTPPPAGSNTGQMATTTPSRELGV     505
Suzuki          SFLFCFANHDVTFAIRTLLNKLLPSLVTPPPAGSNTGQMATTTPSRELGV     498
biarmipes       SFLFCFANHDVTFAIRTLLNKLLPSLVTPPPAGSNTGQMATTTPSRELGV     498
grimshawi       SFVFCFVNQDVIVAIRTLLNKWMPSLVSAPPAGSNTGQMATTTPSRELGV     490
virilism        SFLFCFANHDVTFAMRTLLNKLMPTLVAPPPAGSNTGQLATTTPSRELGV     494
mojavensis      SFLFCFANHDVTFAIRTMLNKWMPNLIAPPPAGSNTGQLATTTPSRELGV     499
```

melanogaster

```
1 mattssdses qnvdvasqaq tqdnlriflk hlyaecvfry qnvtydtddp sfslgpatdy

       61 dsdlpenfsp vprylenaam negvidmrnv deelaekeel matvvsatma tnqkenrlfc

      121 plnfdgylcw prtpagtvls qycpdfvegf nrkflahktc lengswyrhp vsnqtwsnyt

      181 ncvdyedlef rqfinelyvk gyalsllall isiiiflgfk slrctririh vhlfaslact

      241 cvawilwyrl vversetiae nplwciglhl vvhyfmlvny fwmfceglhl hlvlvvvfvk

      301 dtivmrwfiv iswfspipia ivyglarhfs spdnkhcwit dslylwifsv pitlsllasf

      361 iflinvlrvi vrklhpqsaq paplairkav ratiilvplf glqhfllpyr pdagtqldhf

      421 yqmlsvvlvs lqgfvvsflf cfanhdvtfa irtllnkllp slvtpppags ntgqmatttp

      481 srelgv
```

simulans XP_016039137.1

```
  1 mattssdsdv envdvasqaq tqdnlriflk hlyaecvfry qnvtydtddp sfslgpatdy
       61 dsdlpenfsp vprylenaam negvidmrnv deelaekeel matvvsatma tnqkesrlfc
      121 plnfdgylcw prtpagtvls qycpdfvegf nrkflahktc lengswyrhp vsnqtwsnyt
      181 ncvdyedlef rqfinelyvk gyalsllall isiiiflgfk slrctririh vhlfaslact
      241 cvawilwyrl vversetiae nplwciglhl vvhyfmlvny fwmfceglhl hlvlvvvfvk
```

```
301 dtivmrwfiv iswfspipia ivyglarhfs spdnkhcwit dslylwifsv pitlsllasf
361 iflinvlrvi vrklhpqsaq paplairkav ratiilvplf glqhfllpyr pdagtqldhf
421 yqmlsvvlvs lqgfvvsflf cfanhdvtfa irtllnkllp slvtpppags ntgqmatttp
481 srelgv
```

sechellia **XP_002042869.1**

```
1 mattssdsdv envdvasqaq tqdnlriflr hlyaecvfry qnvtydtddp sfslgpatdy
61 dsdlpenfsp vprylenaam negvidmsnv deklaekeel matvvsatma tnqkesrlfc
121 plnfdgylcw prtpagtvls qycpdfvegf nrkflahktc lengswyrhp vsnqtwsnyt
181 ncvdyedlef rqfinelyvk gyalsllall isiiiflgfk slrctririh vhlfaslact
241 cvawilwyrl vversetiae nplwciglhl vvhyfmlvny fwmfceglhl hlvlvvvfvk
301 dtivmrwfiv iswfspipia ivyglarhfs spdnkhcwit dslylwifsv pitlsllasf
361 iflinvlrvi vrklhpqsaq paplairkav ratiilvplf glqhfllpyr pdagtqldhf
421 yqmlsvvlvs lqgfvvsflf cfanhdvtfa irtllnkllp slvtpppags ntgqmatttp
481 srelgv
```

erecta **XP_001978328.2**

```
1 mtavthteqp tmattssdse plnldvasqa qtqdnlrifl khlyaecvfr yqnvtydtgd
61 psfslgpatd ydsdlpenfs pvprylenaa mnegvidmrs vdeelaekee lmatvvsatm
121 atnqkehrlf cplnfdgylc wprtpagtvl sqycpdfveg fnrkflahkt clengswyrh
181 pvsnqtwsny tncvdykdle frqfinelyv kgyalsllal lisiiiflgf kslrctriri
241 hvhlfaslac tcvawilwyr lvversetia enplwciglh lvvhyfmlvn yfwmfceglh
301 lhlvlvvvfv kdaivmrwfi liswflpihi tilyglarhf stpdnkhcwi tdslylwifs
361 vpitlsllas fiflinvlrv ivrklhpqsa qpaplairka vratiilvpl fglqhfllpy
421 rpdagtqldh fyqmlsvvlv slqgfvvsfl fcfanhdvtf avrtllnkwl pslviappag
481 sntgqmattt psrelgv
```

Suzuki **XP_016923892.1**

```
1 mtrvspteap tmgtaasvse senvevasqt qtqdnlrifl khlyaecvyr yqnvtydtdd
61 psvslgaatd ydfdlpenfs pvprylenaa mnegaidmrn vdaelaekee lvatvvsatm
121 atnqreeprl fcplnfdgyl cwprtpagtv lsqycpdfve gfnrkflahk tclengswfr
181 hpesnqtwsn ytncvdyedl efrqfvnely vrgyalslla llisiiiflg fkslrctrir
241 ihvhlfasla ctcvawilwy rlvverpeti adnplwcigl hlvvhyfmlv nyfwmfcegl
301 hlhlvlvvvf vkdtivmrwf mviswlspip iavvyglarh fsspdnkhcw indslylwif
361 svpitlslla sfiflinvlr vivrklhpqs aqpaplairk avratiilvp lfglqhfllp
421 yrpdagtqld hfyqmlsvil vslqgfvvsf lfcfanhdvt fairtllnkl lpslvtpppa
481 gsntgqmatt tpsrelgv
```

biarmipes **XP_016956301.1**

```
1 mtraspieap tmgtaasese senveaasqt qtqdnlrifl khlyaecvyr yqnvtydtdd
61 psvslgaatd ydfdlpenfs pvprylenaa lnegaidmrn vdaeqaekee lvatvvsatm
121 atnqraeprl fcplnfdgyl cwprtpagtv lsqycpdfve gfnrkflahk tclengswfr
181 hpesnqtwsn ytncvdyedl efrqfvnely vrgyalslla llisiiiflg fkslrctrir
241 ihvhlfasla ctcvawilwy rlvverpeti adnplwcigl hlvvhyfmlv nyfwmfcegl
301 hlhlvlvvvf vkdtivmrwf mviswlspip iaivyglarh fsspdnkhcw indslylwif
361 svpitlslla sfiflinvlr vivrklhpqs aqpaplairk avratiilvp lfglqhfllp
421 yrpdagtqld hfyqmlsvil vslqgfvvsf lfcfanhdvt fairtllnkl lpslvtpppa
481 gsntgqmatt tpsrelgv
```

takahashi **XP_016995732.2**

```
1 mtrvshtepp tmgaaasdsq senveaasqs qmqdnlrifl khlyaecvyr yqnvtydted
61 psvplgaatd ydfdlpenfs pvprylenaa lnegaidmrn vdavlaekee lmatvvsatm
121 atnqrqaeeg geeerrlfcp lnfdgylcwp rtpagtvlsq ycpdfvegfn skflahktcl
181 engswfrhpv snqtwsnytn cvdyedlefr qfinelyvkg ytlsllalli siiiflgfks
241 lrctririhv hlfaslactc vawilwyrlv vergeaiaen plwciglhlv vhyfmlvnyf
301 wmfceglhlh lvlvvvfvkd tivmrwfivi swfspipvai vyglarhfss pdnkhcwind
361 slylwifsvp itlsllasfi flinvlrviv rklhpqsaqp aplairkavr atiilvplfg
421 lqhfllpyrp dagtqldhfy qmlsvvlvsl qgfvvsflfc fanhdvtfai rtllnkwlps
481 lvtpppagsn tgqmatttps relgv
```

elegans **XP_017133493.1**

```
1 mttvthfdtp tmgtvasdst menvetatqs qtqdnlrifl khlyaecvyr yknvtydaad
61 psvslvaatd yesdltenfs pvpryledav lnegaidmrn vdeelaekee lmatvlsatm
121 atnqqepllf cplnfdgylc wprtpagtvl sqycpdfveg fnskflahkt clengswfrh
```

181 pvsnqtwsny tncvdyedle frqfinelyv kgyalsllal lisiiiflgf kslrctriri
241 hvhlfaslac tcvawilwyr lvvernetia enplwciglh lvvhyfmlvn yfwmfceglh
301 lhlvlvvvfv kdtivmrwfi viswlspipv aivyglarhf sgpdnkhcwi ndslylwifs
361 vpitlsllas fiflinvlrv ivrklhpqsa qpaplairka vratiilvpl fglqhfllpy
421 rpdagtqldr fyqllsvvlv slqgfvvsfl fcfanhdvtf airtllnkwl prlvtappag
481 sntgqmattt psrelgv

rhopaloa XP_016985474.1
1 mgtvasdsvp envetatqsq tqdnlriflk hlyaecvyry qndtydaddp svslgaatdy
61 dldlpesfsp vpryledavl negaidmrnv deelaekeel matvlsatma tnqrpqqrlf
121 cplnfdgylc wprtpagtvl sqycpefveg fnskflahkt clengswfrh pvsnqtwsny
181 tncvdyedle frqfinelyv kgyalsllal lisiiiflgf kslrctriri hvhlfaslac
241 tcvawilwyr lvvernetia enplwcsglh lvvhyfmlvn yfwmfceglh lhlvlvvvfv
301 kdtivmrwfi viswlspipi avvyglarhf tspdnkhcwi ndslylwifs vpitlsllas
361 fiflinvlrv ivrklhpqsa qpaplairka vratiilvpl fglqhfllpy rpdagtrldr
421 fyqllsvvlv slqglvvsfl fcfanhdvtf airtllnkwl prlvtappag sntgqmattt
481 psrelgv

ficusphelia XP_017052090.1
1 mgtgasssds gsekletaaq aqtqdnlrif lkhlyaecvy ryqnvtyead dpsvplgaat
61 eydvdanapd nfspvpryle davlnegaid mrhvdeeaae keelmatvft atmatnqree
121 rlfcplnfdg ylcwprtpag tvlsqycpdf vegfnskfla hktclengsw frhpvsnqtw
181 snytscvdye dlefrqfine lyvkgyalsl lallisiiif lgfkslrctr irihvhlfas
241 lactcvawil wyrlvverpe tiaenqlwci glhlvvhyfm lvnyfwmfce glhlhlvlvv
301 vfvkdtivmr wfiviswfsp ipiaivygla rhfsspdnkh cwindslylw ifsvpitlsl
361 lasfiflinv lrvivrklhp qsaqpaplai rkavratiil vplfglqhfl lpyrpdagtq
421 ldrfyqllsv vlvslqgfvv sflfcfvnhd vtfairtlln kmmptlvtap pagsntgqma
481 tttpsrelgv

eugracilis XP_017065034.1
1 mtrvthietp tmvgttases esenvemasq aqtqdnllvf lthlyaecvh ryqnvtndte
61 dsslslgaat dsendlkgnf stvprylena alnegtidmr nvdeklaeqe elmatvssat
121 matnqrdrrl fcpldfdgyl cwprtpagtv lsqycpdfve gfnkkflahk tclengswfr
181 hpatnqtwsn ytncvdhedl efrkfinely vkgyalslla llisiiiflg fkslrctrir
241 ihvhlfasla ctcvawilwy rlvverheti tenplwcigl hlvvhyfmlv nyfwmfcegl
301 hlhlvlvvvf vkdtivmrwf iviswfspip iaavyglarh fsnpdnkhcw indslylwmf
361 svpitlslla sfiflinvlr vivrklhpqs aqpaplairk avratiilvp lfglqhfllp
421 yrpdagsqld rfyqmlsval vspqgfvvsf lfcfanhdvt fairtllnkw lpslvtpppa
481 gsntgqmatt tpsrelgv

kikkawei XP_017029945.1
1 mtsieeappt mgtnasdsds dsgsenvama tatasqqsyt qpqaqdnlri flrhlyaecv
61 yryqndtydd psvsvvgeaa atdydldlpe nfspvpryle navlnegaid mrnvdveqae
121 keellatvls gtmatnqqkq shqattttg erlfcplnfd gylcwprtpa gtvlsqycpd
181 fvegfnskfl ahktclengs wfrhpmtnrt wsnytncvdy edlefrqfin elyvkgyals
241 llallvsiii flgfkslrct ririhvhlfa slaftcvawi lwyrlvverp eiiadnplwc
301 iglhlvvhyf mlvnyfwmfc eglhlhlvlv vvfvkdtivm rwfiiiswfs pihfaivygl
361 arhfsdsdne hcwindslyl wifsvpitls lvasfiflin vlrvivrklh pqsaqpapla
421 irkavratii lvplfglqhf llpyrpdagt qldrfyqlls vvlvslqgfv vsflfcfanh
481 dvtfairtll nkwlpslvaa ppagsntgqm atttpsrelg v

seratta KAH8380938.1
1 mamakasgsq qsssqtqaqd nlriflrhly aecvyryqnd tyddpsvsvd gaaatdyese
61 lpenfspvpr ylenavlneg aidmrnvdve laekeellat vlsvtmatnq qesehhevtt
121 tnnrsagerl fcplnfdgyl cwprtpagtv lsqycpdfve gfnskflahk tclengswfr
181 hpmsnqtwsn ytncvdyedl efrqfinely vkgyalslla llvsiliflg fkslrctrir
241 ihvhlfasla ftcvawilwy rlvverpeii adnplwcigl hlvvhyfmlv nyfwmfcegl
301 hlhlvlvvvf vkdtivmrwf iiiswfspih faivyglsrh fsdsdnehcw indslylwif
361 svpitlslla sfiflinvlr vivrklhpqs aqpaplairk avratiilvp lfglqhfllp
421 yrpdagtqld rfyqllsvvl vslqgfvvsf lfcfanhdvt fairtllnkw lpsvvapppa
481 gsntgqmatt tpsrelgv

bipectinata XP_017095188.2
1 mgglsasvte pdmessraqd apqpqdnlri flkhlyaecv yryqndtydd pstesatdye
61 sdfpenfspv prylenaaln egsidmrnvd ekkaereelf atiltatmat nqnqddpagq
121 rspgsnssss rrlfcpldfd gylcwprtpa gtvlsqycpd fvegfnskfl ahktcqengs
181 wyrhpetnkt wsnytncvdy vdfefrqfin elyvkgyals llallisiii flgfkslrct
241 ririhvhlfa slactcvawi lwyrlvveqp eitaenplwc ivlhlvvhyf mlvnyfwmfc
301 eglhlhlvlv vvfvkdtivm rwfiiiswls pvhiavvygl arhfsspdne hcwindslyl

```
361 wifsvpitls llasfiflin vlrvivrklh pqsaqpapla irkavratii lvplfglqhf
421 llpyrpdagt qldrfyqlls vvlvslqgfv vsflfcfanh dvlfavrtll nkllpslvsp
481 ppagsntgqm atttpsrelg v
```

ananassae **XP_001964132.2**

```
  1 mgglsasvte pdmeqeapqp qdnlriflkh lyaecvyryq ndtyddpmte tatdyesdlp
 61 enfspvpryl enaalnegsi dmrnvdekqa ekeelfatil tatmatnqnq depagqrssg
121 nssssgskrl fcpldfdgyl cwprtpagtv lsqycpdfve gfnskflahk tcqengswyr
181 hpesnktwsn ytncvdyvdl efrqfinely vkgyaislla llisiiiflg fkslrctrir
241 ihvhlfasla ctcvawilwy rlvverpeit aenplwcill hlvvhyfmlv nyfwmfcegl
301 hlhlvlvvvf vkdtivmrwf iiiswlspvh favvyglarh fsspdnehcw indslylwif
361 svpitlslla sfiflinvlr vivrklhpqs aqpaplairk avratiilvp lfglqhfllp
421 yrpdagtqld rfyqllsvvl vslqgfvvsf lfcfanhdvl favrtllnkl mpslvspppa
481 gsntgqmatt tpsrelgv
```

virilism **XP_032296576.1**

```
  1 mallraaltm ataayfemtv sgamppqpqd nlrtflkhly aecvyryqnd tataqlatep
 61 ddggllllety tmipryleqa vlnegtidmq dvdeeaasen elyatvlsat matnhhshnt
121 semetlycpv nfdgylcwpr tpagtvlsqy cpdfvegfns kflahktcle tgswfrhpvs
181 nqtwsnytnc vdyedfqfrq fvnelyvkgy alsllalfis iviflgfksl rctririhvh
241 lfaslactci awilwyrlvv ehteqiaenp pwcialhlvv hyfmlvnyfw mfceglhlhl
301 vlvvvfvkdt ivmrwfklls wllpllfvlp ygvarhfsan dnahcwmnds fylwifsvpi
361 tlsllasfif linvlrvivr klhpqsaqpa plairkavra tiilvplfgl qhfllpyrpd
421 agtqldrfyq llsvvlvslq gfvvsflfcf anhdvtfamr tllnklmptl vapppagsnt
481 gqlatttpsr elgv
```

mojavensis **XP_015016349.1**

```
  1 mallraalam ttaayietam tasltkpqpr pqpqdnlrif lrhlyvecvy ryqndtakgd
 61 akdsgdeelm latfttvpry leqavlnegt idmqdvdeea asenelyati hsmtmetnqh
121 srnatsemet lycpvnfdgy lcwprtpagt vlsqycpdfv egfsskflah ktclengtwy
181 rhpvsnqtws nytncvdydd fqfrqfvnel yvkgyalsll aliisivifl gfkslrctri
241 rihvhlfgsl actciawilw yrlvveqtdq iaenppwcia lhlvvhyfml vnyfwmfceg
301 lhlhlvlvvv fvkdtivmrw fkllswlspl vfvlpygvar hlsvndnehc windslylwi
361 fsvpitlsll asfiflinvl rvivrklhpq saqpaplair kavratiilv plfglqhfll
421 pyrpeagtql drfyqllsvv lvslqgfvvs flfcfanhdv tfairtmlnk wmpnliappp
481 agsntgqlat ttpsrelgv
```

grimshawi **XP_001992054.1**

```
1 mtasasasms pqpqdnlltf lwhlyaecvy ryqndtspgq qatdpgdgni qllepytrmp
 61 nylemavlne gtidmqdvne easslnelya tvlsatmapn qhsiqyngnn snanttreme
121 ilycpvnfdg ylcwprtpag tvlsqycpdf vegfntkfla hktcmetgsw frhpvsnqtw
181 snytncvdye dlqfrqivne lyvkgyalsl lallisiiif vgfkslrcnr irihvhlfas
241 lactcltwil wyrlvvehse riaenpnwci alhlvvhyfm lvnyfwmfce glhlhlvlvv
301 vfvkdtivlr wfkffswlsp llfiipygvv rhfsandnkh cwisesfylw ilsvpitlsl
361 lasfiflinv lrvivrklhp qsahpapmai rkavratiil vplfglqhfl lpyrpeagtk
421 ldrfyqlmsv vlvslqgfvv sfvfcfvnqd vivairtlln kwmpslvsap pagsntgqma
481 tttpsrelgv
```
