# Supplementary material for: The incidence of candidate binding sites for β-arrestin in Drosophila neuropeptide GPCRs
Source: PLoS One. 2022 Nov 1;17(11):e0275410. doi: 10.1371/journal.pone.0275410 (PMC9624432; doi:10.1371/journal.pone.0275410)
Supplement: S17 Text — (PDF) [file pone.0275410.s021.pdf]

***S17 Text.* Multi-species analysis of CG33639 R**
Supporting Figure 20

CLUSTAL Line-ups; Genbank Reference IDs below
Predicted TM domains in YELLOW
BBS sequences in RED

```
Grimshawi          ------------MITRLYNTEEDPVYCSFVWGANVSNGRS-----------HAANSSALY     37
Mojavensis         MERIVRNHQSKTMITRLYNTEEDPAYCSVIWGANLTSSSD-------LVLGTATNATHIY     53
Virilism           ------------MITRLYNTEEDPAYCSFIWGANLTSSND-------LVLGTTANSTHIY     41
Bipectinate        ------------MITRLYHTEEDPAYCSFIGGSNLSS--------SSDVLATSANATPVF     40
Anannassae         ------------MITRLYHTEEDPAYCSFIGGSNISISSSSSSISSSDVLATSANATPVF     48
Serrata            ------------MITRLYNTEEDPAYCSFIWGSNLSSTT--------DVLA-AAGNSSPV     39
Kikkawei           ------------MITRLYNTEEDPAYCSFIWGSNLSSTT--------DVLA-AAGNSSPV     39
Fichsuphila        ------------MITRLYNTEEDPAYCSFIWGGSNLTAT--------AAEVLAANATSIF     40
Eugracilis         ------------MITSLYTTQEDPAYCSFIWGSNL--TT--------SADVLTANATSVF     38
Rhopaloa           ------------MITRLYNTEEDPAYCSFIWGANL--TS--------SADVLA-NATSVF     37
Elegans            ------------MITRLYNTEEDPAYCSFIWGSNL--TS--------SADVLA-NATSAF     37
Erecta             ------------MITRLYNTEEDPAYCSFIWGSNL--TS--------SVDVLAANATSVF     38
Suzuki             ------------MITRLYNTEEDPAYCSFIWGSNL--TS--------SAEVLAANATSVF     38
Biarmipes          ------------MITRLYNTEEDPAYCSFIWGSNL--TS--------SAEVLAANATSIF     38
Takahashi          ------------MITRLYNTEEDPAYCSFIWGSNL--TS--------SADVLAANATSVF     38
Sechellia          ------------MITRLYNTEEDPAYCSFIWGSNL--TS--------SVDVLAANATSVF     38
Melanogaster       ------------MITRLYNTEEDPAYCSFIWGSNL--TS--------SVDVLAANATSVF     38
Simulans           ------------MITRLYNTEEDPAYCSFIWGSNL--TS--------SVDVLAANATSVF     38
Mauritania         ------------MITRLYNTEEDPAYCSFIWGSNL--TS--------SVDVLAANATSVF     38
                               *** ** *:***.***.: *..               : . :

Grimshawi          PDDMRDDWFADAEDPRTELLRQYCYGYFLPFICASGIIGNVLNLIVLTRRNMRGPSYIYM     97
Mojavensis         ANDLRDDLYADVEDPRTEALREYCYGLMLPIICALGIIGNVLNLIVLTRRNMRGTAYIYM     113
Virilism           ANDLRDDLYADVEDPRTESLREYCYGLMLPVICALGIIGNVLNLIVLTRRNMRGTAYIYM     101
Bipectinate        GSDLRDDFYRDVEDPRTESLREYCYGLLLPIICAMGIIGNVLNLIVLTRRNMRGIAYIYM     100
Anannassae         GSDLRDDFYRDVEDPRTESLREYCYGLLLPIICAMGIIGNVLNLIVLTRRNMRGIAYIYM     108
Serrata            FTDLRDDFYRDVEDPRTESLREYCYGLLLPIICAMGIIGNVLNLIVLTRRNMRGTAYIYM     99
Kikkawei           FTDLRDDFYRDVEDPRTESLREYCYGLLLPITCAMGIIGNVLNLIVLTRRNMRGTAYIYM     99
Fichsuphila        GTDLRDDFYRDVEDPRTESLREYCYGLLLPIICAMGIIGNLLNLVVLTRRNMRGTAYIYM     100
Eugracilis         SSDLRDDFYRDVEDPRTESLREYCYGLVLPIICAMGIIGNVLNLIVLTRRNMRGTAYIYM     98
Rhopaloa           NTDLRDDFYRDVEDPRTESLREYCYGLVLPIICAMGIIGNVFNLIVLTRRNMRGTAYIYM     97
Elegans            SSDMRDDFYRDVEDPRTESLREYCYGLVLPIICAMGIIGNVLNLIVLTRRNMRGTAYIYM     97
Erecta             SSDLRDDFYRDVEDPRTESLREYCYGLVLPIICAMGIIGNVLNLIVLTRRNMRGTAYIYM     98
Suzuki             SSDLRDDFYRDVEDPRTESLREYCYGLVLPIICAMGIIGNVLNLIVLTRRNMRGTAYIYM     98
Biarmipes          SSDLRDDFYRDVEDPRTESLREYCYGLVLPIICAMGIIGNVLNLIVLTRRNMRGTAYIYM     98
Takahashi          SSDLRDDFYRDVEDPRTESLREYCYGLVLPIICAMGIIGNVLNLIVLTRRNMRGTAYIYM     98
Sechellia          SSDLRDDFYRDVEDPRTESLREYCYGLVLPIICAMGIIGNVLNLVVLTRRNMRGTAYIYM     98
Melanogaster       SSDLRDDFYRDVEDPRTESLREYCYGLVLPIICAMGIIGNVLNLVVLTRRNMRGTAYIYM     98
Simulans           GSDLRDDFYRDVEDPRTESLREYCYGLVLPIICAMGIIGNVLNLVVLTRRNMRGTAYIYM     98
Mauritania         SSDLRDDFYRDVEDPRTESLREYCYGLVLPIICAMGIIGNVLNLVVLTRRNMRGTAYIYM     98
                     *:*** : *.****** **:**** .**. ** *****::**:********* :****

Grimshawi          RAYSTAALLAIVFAIPFGIRMLVHKDRGQWEEIGPAFYTAHLELFLGNGCLGVGVMMLLV     157
Mojavensis         RAYSTAALLAIVFAIPFGIRMLVHKDRGQWEEFGPAFYTAHLELFLGNGCLGVGVMMLLV     173
Virilism           RAYSTAALLAIVFAIPFGIRMLVHKDRGQWEEFGPAFYTAHLELFLGNGCLGVGVMMLLV     161
Bipectinate        RAYSTAALLAIVFAIPFGIRMLVHKDRGQWEEFGPAFYTAHLELYLGNGCLGVGVMMLLV     160
Anannassae         RAYSTAALLAIVFAIPFGIRMLVHKDRGQWEEFGPAFYTAHLELYLGNGCLGVGVMMLLV     168
Serrata            RAYSTAALLAIVFAIPFGIRMLVHKDRGQWEEFGPAFYTAHLELYLGNGCLGVGVMMLLV     159
Kikkawei           RAYSTAALLAIVFAIPFGIRMLVHKDRGQWEEFGPAFYTAHLELYLGNGCLGVGVMMLLV     159
Fichsuphila        RAYSTAALLAIVFAIPFGIRMLVHKDRGQWEEFGPAFYTAHLELYLGNGCLGVGVMMLLV     160
Eugracilis         RAYSTAALLAIVFAIPFGIRMLVHKDRGQWEKFGPAFYTAHLELYLGNGCLGIGVMMLLV     158
Rhopaloa           RAYSTAALLAIVFAIPFGIRMLVHKDRGQWEEFGPAFYTAHLELYLGNGCLGVGVMMLLV     157
Elegans            RAYSTAALLAIVFAIPFGIRMLVHKDRGQWEEFGPAFYTAHLELYLGNGCLGVGVMMLLV     157
Erecta             RAYSTAALLAIVFAIPFGIRMLVHKDRGQWEEFGPAFYTAHLELYLGNGCLGVGVMMLLV     158
Suzuki             RAYSTAALLAIVFAIPFGIRMLVHKDRGQWEEFGPAFYTAHLELYLGNGCLGVGVMMLLV     158
Biarmipes          RAYSTAALLAIVFAIPFGIRMLVHKDRGQWEEFGPAFYTAHLELYLGNGCLGVGVMMLLV     158
Takahashi          RAYSTAALLAIVFAIPFGIRMLVHKDRGQWEEFGPAFYTAHLELYLGNGCLGVGVMMLLV     158
Sechellia          RAYSTAALLAIVFAIPFGIRMLVHKDRGQWEEFGPAFYTAHLELYLGNGCLGVGVMMLLV     158
Melanogaster       RAYSTAALLAIVFAIPFGIRMLVHKDRGQWEEFGPAFYTAHLELYLGNGCLGVGVMMLLV     158
Simulans           RAYSTAALLAIVFAIPFGIRMLVHKDRGQWEEFGPAFYTAHLELYLGNGCLGVGVMMLLV     158
Mauritania         RAYSTAALLAIVFAIPFGIRMLVHKDRGQWEEFGPAFYTAHLELYLGNGCLGVGVMMLLV     158
```

```
                  *******************************::***********:*******:*******

Grimshawi         LTIERYVSVCRPGATRPAIGQPGVVIFIICVLTFIFYLPSIFRGELIKCMLTSKNVYVYL   217
Mojavensis        LTIERYVSVCHPGFTRPVMGPPGVVVFVTCLVTFIIYLPSIFRGELIKCMLTSNNVYVYL   233
Virilism          LTIERYVSVCHPGFTRPVMGPPGVVVFLTCFATFIIYLPSIFRGELIKCMLTSNNVYVYL   221
Bipectinate       LTIERYVSVCHPGFSRPVMGPPGVVVFFTCLATVIVYLPSIFRGELIKCMLGSSDVYVYL   220
Anannassae        LTIERYVSVCHPGFSRPVMGPPGVVVFFTCLATVIVYLPSIFRGELIKCMLGSSDVYVYL   228
Serrata           LTIERYVSVCHPGFARPVMGPPGVVVFLTCLATVIVYLPSIFRGELIKCILGSSDVYVYL   219
Kikkawei          LTIERYVSVCHPGFARPVMGPPGVVVFLTCLATVIVYLPSIFRGELIKCILGSSDVYVYL   219
Fichsuphila       LTIERYVSVCHPGFVRPVMGPPGVVVFLTCLATVIVYLPSIFRGELIKCVLHASDVYVYL   220
Eugracilis        LTIERYVSVCHPGFARPVMGPPGVVVFLTCLATVIVYLPSIFRGELIKCMYGSRDVYVYL   218
Rhopaloa          LTIERYVSVCHPGFARPVMGPPGVVVFFTCLATVIVYLPSIFRGELIKCIFGSSDAFVYL   217
Elegans           LTIERYVSVCHPGFARPVMGPPGVVVFLTCLATVIVYLPSIFRGELIKCIFGSSDVFVYL   217
Erecta            LTIERYVSVCHPGFARPVMGPPGVVVFLTCLATVIVYLPSIFRGELIKCILGSSDVYVYL   218
Suzuki            LTIERYVSVCHPGFARPVMGPPGVVVFLTCLATVIVYLPSIFRGELIKCIFGSSDVYVYL   218
Biarmipes         LTIERYVSVCHPGFARPVMGPPGVVVFLTCLATVIVYLPSIFRGELIKCIFGSSDVYVYL   218
Takahashi         LTIERYVSVCHPGFARPVMGPPGVVVFLTCLATVIVYLPSIFRGELIKCIFGSSDVYVYL   218
Sechellia         LTIERYVSVCHPGFARPVMGPPGVVVFLTCLATVIVYLPSIFRGELIKCILGSSDVYVYL   218
Melanogaster      LTIERYVSVCHPGFARPVMGPPGVVVFLTCLATVIVYLPSIFRGELIKCILGSSDVYVYL   218
Simulans          LTIERYVSVCHPGFARPVMGPPGVVVFLTCLATVIVYLPSIFRGELIKCILGSSDVYVYL   218
Mauritania        LTIERYVSVCHPGFARPVMGPPGVVVFLTCLATVIVYLPSIFRGELIKCILGSSDVYVYL   218
                  **********:**  **.:* ****:*. *. *.*.*************:  : :.:***

Grimshawi         RRDNIIYQQTLFYSVYKVLVEIIFKIIPTFIIAGLNLRIMWVYRRTCARRRQMVLTR---   274
Mojavensis        RRDNNIYQRTIFYSVYKIMLEVIFKLVPTVVIAGLNLRIMLVYRRTCERRRQMVLSR---   290
Virilism          RRDNNIYQRTIFYSVYKIMLEVIFKLIPTVLIAGLNLRIMLVYRRTCERRRQMVLTR---   278
Bipectinate       RRDNTIYQQTLFYRVYKIMLEVIFKLIPTVLIGGLNMRIMMVYRRTCERRRQMVLSRPQA   280
Anannassae        RRDNTIYQQTLFYRVYKIMLEVIFKLIPTVLIGGLNMRIMMVYRRTCERRRQMVLSRPQ-   287
Serrata           RRDNTIYQQTIFYRIYKIMLEVIFKLVPTVLIGGLNMRIMMVYRRTCERRRQMVLSRNHG   279
Kikkawei          RRDNTIYQQTIFYRIYKIMLEVIFKLVPTVLIGGLNMRIMMVYRRTCERRRQMVLSRNHG   279
Fichsuphila       RRDNTIYQQTIFYRVYKIMLEVIFKLVPTLVIGFLNMRIMMVYRRTCERRRQMVLSRPH-   279
Eugracilis        RRDNTIYQQTIFYRVYKIMLEVIFKLIPTLVIGGLNMRIMMVYRRTCERRRQMVLSRPH-   277
Rhopaloa          RRDNTIYQQTIFYRVYKIMLEVIFKLVPTLVIGGLNMRIMMVYRRTCERRRQMVLSRPHA   277
Elegans           RRDNTIYQQTIFYRVYKIMLEVIFKLVPTLVIGGLNMRIMMVYRRTCERRRQMVLSRPHG   277
Erecta            RRDNTIYQQTIFYRVYKIMLEVIFKLVPTLVIGGLNMRIMMVYRRTCERRRQMVLTRSHA   278
Suzuki            RRDNTIYQQTIFYRVYKIMLEVIFKLVPTLVIGGLNMRIMMVYRRTCERRRQMVLSRPHA   278
Biarmipes         RRDNTIYQQTIFYRVYKIMLEVIFKLVPTLVIGGLNMRIMMVYRRTCERRRQMVLSRPHA   278
Takahashi         RRDNTIYQQTIFYRVYKIMLEVIFKLVPTLVIGGLNLRIMMVYRRTCERRRQMVLSRPHA   278
Sechellia         RRDNTIYQQTIFYRVYKIMLEVIFKLVPTLVIGGLNMRIMMVYRRTCERRRKMVLSRSHA   278
Melanogaster      RRDNTIYQQTIFYRVYKIMLEVIFKLVPTLVIGGLNMRIMMVYRRTCERRRKMVLSRPHA   278
Simulans          RRDNTIYQQTIFYRVYKIMLEVIFKLVPTLVIGGLNMRIMMVYRRTCERRRKMVLSRPHA   278
Mauritania        RRDNTIYQQTIFYRVYKIMLEVIFKLVPTLVIGGLNMRIMMVYRRTCERRRKMVLSRPHA   278
                  **** ***:*:** :**:::*:***::**.:*. **:*** ****** ***:***:*

Grimshawi         ------------------------AIYAKNEDPRKFAEERRLFLLLGSTSILFLLCISPM   310
Mojavensis        ------------------------ATYVKDDDPRKFAEERRLFLLLGSTSILFLLCVSPM   326
Virilism          ------------------------ANYVKDDDPRKFAEERRLFLLLGSTSILFLLCVSPM   314
Bipectinate       HHHHHHN--------------ANGPGYVKDDDPRKFAEERRLFLLLGSTSILFLVCVSPM   326
Anannassae        ---AHHH--------------QNGPGYVKDDDPRKFAEERRLFLLLGSTSILFLVCVSPM   330
Serrata           HGHGHSHGQGHAQGHA------HTNGYVKDDDPRKFAEERRLFLLLGSTSILFLVCVSPM   333
Kikkawei          HGHGHSHAQGQAQGHA------HTNGYVKDDDPRKFAEERRLFLLLGSTSILFLVCVSPM   333
Fichsuphila       -----------------------AHGYLKDDDPRKFAEERRLFLLLGSTSILFLVCVSPM   316
Eugracilis        -----------------------AHGYMKDDDPRKFAEERRLFLLLGSTSILFLVCVSPM   314
Rhopaloa          HAHGHGHAHGQGHG--------HSHGYLKDDDPRKFAEERRLFLLLGSTSILFLVCVSPM   329
Elegans           HGHGHGHGHGHGHGAQGHGHAHGGHGYLKDDDPRKFAEERRLFLLLGSTSILFLVCVSPM   337
Erecta            YGHGH---------------G-HGHGYLKDDDPRKFAEERRLFLLLGSTSILFLVCVSPM   322
Suzuki            ------------------------HGYMKDDDPRKFAEERRLFLLLGSTSILFLVCVSPM   314
Biarmipes         ------------------------HGYMKDDDPRKFAEERRLFLLLGSTSILFLVCVSPM   314
Takahashi         HGHGHGHGHGHGHGH---GHG-HGHGYMKDDDPRKFAEERRLFLLLGSTSILFLVCVSPM   334
Sechellia         QGHGHGH----GHGH---GHA-HGHGYLKDDDPRKFAEERRLFLLLGSTSILFLVCVSPM   330
Melanogaster      QGHGHGHGHGHGHGH---GHA-HGHGYLKDDDPRKFAEERRLFLLLGSTSILFLVCVSPM   334
Simulans          QGHGHGHGHGHGHGH---GHA-HGHGYLKDDDPRKFAEERRLFLLLGSTSILFLVCVSPM   334
Mauritania        QGHGHGHGHGHGHGH---GHA-HGHGYLKDDDPRKFAEERRLFLLLGSTSILFLVCVSPM   334
                                            * *::***********************:*:***


Grimshawi         AILHMTIASEVLPSFPFQVFRALANLLELINYSITFYIYCLFSEDFRNTLLRTFNWPWVK   370
Mojavensis        AILHMTIASEVLPSFPFQVFRAMANLLELINYSITFYIYCLFSEDFRNTLMRTIKWPWLK   386
Virilism          AILHMTIASEVLPSFPFQVFRALANLLELINYSITFYIYCLFSEDFRNTLMRTIKWPWLK   374
```

```
Bipectinate       AILHMTIASEVYPSFPFQVFRASANLLELINYSLTFYIYCLFSEDFRNTLVRTIKWPWLK        386
Anannassae        AILHMTIASEVYPSFPFQVFRASANLLELINYSLTFYIYCLFSEDFRNTLVRTIKWPWLK        390
Serrata           AILHMTIASEVYPSFPFQVFRASANLLELINYSLTFYIYCLFSEDFRNTLVRTIKWPWLK        393
Kikkawei          AILHMTIASEVYPSFPFQVFRASANLLELINYSLTFYIYCLFSEDFRNTLVRTIKWPWLK        393
Fichsuphila       AILHMTIASEVYPSFPFQVFRASANLLELINYSLTFYIYCLFSEDFRNTLVRTIKWPWLK        376
Eugracilis        AILHMTIASEVYPSFPFQVFRASANLLELINYSLTFYIYCLFSEDFRNTLVRTIKWPWLK        374
Rhopaloa          AILHMTIASEVYPSFPFQVFRASANLLELINYSLTFYIYCLFSEDFRNTLVRTIKWPWLK        389
Elegans           AILHMTIASEVYPSFPFQVFRASANLLELINYSLTFYIYCLFSEDFRNTLVRTIKWPWLK        397
Erecta            AILHMTIASEVYPSFPFQVFRASANLLELINYSLTFYIYCLFSEDFRNTLVRTIKWPWLK        382
Suzuki            AILHMTIASEVYPSFPFQVFRASANLLELINYSLTFYIYCLFSEDFRNTLVRTIKWPWLK        374
Biarmipes         AILHMTIASEVYPSFPFQVFRASANLLELINYSLTFYIYCLFSEDFRNTLVRTIKWPWLK        374
Takahashi         AILHMTIASEVYPSFPFQVFRASANLLELINYSLTFYIYCLFSEDFRNTLVRTIKWPWLK        394
Sechellia         AILHMTIASEVYPSFPFQVFRASANLLELINYSLTFYIYCLFSEDFRNTLVRTIKWPWLK        390
Melanogaster      AILHMTIASEVYPSFPFQVFRASANLLELINYSLTFYIYCLFSEDFRNTLVRTIKWPWLK        394
Simulans          AILHMTIASEVYPSFPFQVFRASANLLELINYSLTFYIYCLFSEDFRNTLVRTIKWPWLK        394
Mauritania        AILHMTIASEVYPSFPFQVFRASANLLELINYSLTFYIYCLFSEDFRNTLVRTIKWPWLK        394
                  *********** ********** **********:***************:**::***:*

Grimshawi         SKLFRRQV-DEVSASPPATATAAVCNPVCPTIHIEHS----------------------        406
Mojavensis        GKLCHQVDETTQAIMGVPMVRFEKVNMRNHHITTTSGIGR------------SSSI----        430
Virilism          SKLCHQVDET-QTIKGVPMVRFDKVTLRNHHITTTSGIGR------------SSSI----        417
Bipectinate       GKFCHQAE-HEVSASP--PATAGTVAA---AGTATTATANP-----------SPAIPANP        429
Anannassae        GKLCHQADQHEVSASP--PATAGTVAA---GIGTAPATANP-----------SPAIPANP        434
Serrata           GKFCHQAE-HEVSASP--PATAGTVAV---AVAVGHGTGP---------VSNPQPIPAIP        438
Kikkawei          GKFCHQAE-HEVSASP--PATAGTVAV---AVAVGHGTGP---------VSNPQPIPAIP        438
Fichsuphila       GKFCHQVE-HEVSASP--PATAGTVAV---AGTGT-GAG----------HVSYYH-PAIP        418
Eugracilis        GKLCHQAE-NEVSASP--PATAGTVAV---AA----GTG--------TGHISKYHAASIP        416
Rhopaloa          GKCCHQAE-HEVSASP--PATAGTVAV---AVAVA-GTGT------APGHVTNFN-PAIP        435
Elegans           GKFCHQGE-HEVSASP--PATAGTVAV---AVAAT-GTA--------SGHVSNLH-PAIP        441
Erecta            GKFCHQGE-HEVSASP--PATAGTVAV---AGIGT-GTGTGTG------NVSHFH-PVIP        428
Suzuki            GKFCHQAE-HEVSGSP--PATAGTVAV---AGIGI-GPGTGNGPGTSSRTKSNFH-PAIP        426
Biarmipes         GKFCHQAE-HEVSASP--PATAGTVAV---AGIVA-GTGPGT------GTKSNFH-PAIP        420
Takahashi         GKFCHQAE-HEVSASP--PATAGTVAV---AGLGT-GTGIG--------QVSNFH-PAIP        438
Sechellia         GKFCHQAE-HEVSASP--PATAGTVAV---AGT-----GTG--------HVSNFH-PAIP        430
Melanogaster      GKFCHQAE-HEVSASP--PATAGTVAV---AGT-----GNG--------HVSIFH-PAIP        434
Simulans          GKFCHQAE-HEVSASP--PATAGTVAV---AGT-----GTG--------HVSNFH-PAIP        434
Mauritania        GKFCHQAE-HEVSASP--PATAGTVAV---AGT-----GTG--------HVSNFH-PAIP        434
                  .*  ::      :      .

Grimshawi         ------NHPEHANGPFRKGQY-------A---        422
Mojavensis        --------------------------------        430
Virilism          --------------------------------        417
Bipectinate       APILPTDP-HGE-EPERLANGTVR--------        451
Anannassae        APILPTDHPHGEKSPQRIPNGTVR--------        458
Serrata           AVTFTPADPEDHTRPANGVLR-----------        459
Kikkawei          AVTFTPADPEDHARPPNGVLR-----------        459
Fichsuphila       SLTFTPAEPEYRPPVCQWPTSLSWQRDNTNIT        450
Eugracilis        ALTLTPAEPAEDRRP-RIANGVHH--------        439
Rhopaloa          ALILTPAEHEE--RPIRLANGVLH--------        457
Elegans           ALTLTPAEPEDRDRPNRLANGVLH--------        465
Erecta            ALTLTPAEPKDRH---RCANGVLH--------        449
Suzuki            ALTLTPAEPEDRP---RLANGVLH--------        447
Biarmipes         ALTLTPAEPEDRP---RLANGVLH--------        441
Takahashi         ALTLTPAEPEERP---RLANGVLH--------        459
Sechellia         ALTLTPAEPDERP---RCANGVLH--------        451
Melanogaster      ALTLTPAEPDERP---RCANGVLH--------        455
Simulans          ALTLTPAEPDERP---RCANGVLH--------        455
Mauritania        ALTLTPAEPDERP---RCANGVLH--------        455
```

Melanogaster NP_001027070.1

```
1 mitrlyntee dpaycsfiwg snltssvdvl aanatsvfss dlrddfyrdv edprteslre
     61 ycyglvlpii camgiignvl nlvvltrrnm rgtayiymra ystaallaiv faipfgirml
    121 vhkdrgqwee fgpafytahl elylgngclg vgvmmllvlt ieryvsvchp gfarpvmgpp
    181 gvvvfltcla tvivylpsif rgelikcilg ssdvyvylrr dntiyqqtif yrvykimlev
    241 ifklvptlvi gglnmrimmv yrrtcerrrk mvlsrphaqg hghghghghg hghghahghg
    301 ylkdddprkf aeerrlflll gstsilflvc vspmailhmt iasevypsfp fqvfrasanl
    361 lelinysltf yiyclfsedf rntlvrtikw pwlkgkfchq aehevsaspp atagtvavag
    421 tgnghvsifh paipaltltp aepderprca ngvlh
```

Simulans XP_016039903.1

```
1 mitrlyntee dpaycsfiwg snltssvdvl aanatsvfgs dlrddfyrdv edprteslre
      61 ycyglvlpii camgiignvl nlvvltrrnm rgtayiymra ystaallaiv faipfgirml
     121 vhkdrgqwee fgpafytahl elylgngclg vgvmmllvlt ieryvsvchp gfarpvmgpp
     181 gvvvfltcla tvivylpsif rgelikcilg ssdvyvylrr dntiyqqtif yrvykimlev
     241 ifklvptlvi gglnmrimmv yrrtcerrrk mvlsrphaqg hghghghghg hghghahghg
     301 ylkdddprkf aeerrlflll gstsilflvc vspmailhmt iasevypsfp fqvfrasanl
     361 lelinysltf yiyclfsedf rntlvrtikw pwlkgkfchq aehevsaspp atagtvavag
     421 tgtghvsnfh paipaltltp aepderprca ngvlh
```

Suzuki XP_036676037.1

```
1 mitrlyntee dpaycsfiwg snltssaevl aanatsvfss dlrddfyrdv edprteslre
      61 ycyglvlpii camgiignvl nlivltrrnm rgtayiymra ystaallaiv faipfgirml
     121 vhkdrgqwee fgpafytahl elylgngclg vgvmmllvlt ieryvsvchp gfarpvmgpp
     181 gvvvfltcla tvivylpsif rgelikcifg ssdvyvylrr dntiyqqtif yrvykimlev
     241 ifklvptlvi gglnmrimmv yrrtcerrrq mvlsrphahg ymkdddprkf aeerrlflll
     301 gstsilflvc vspmailhmt iasevypsfp fqvfrasanl lelinysltf yiyclfsedf
     361 rntlvrtikw pwlkgkfchq aehevsgspp atagtvavag igigpgtgng pgtssrtksn
     421 fhpaipaltl tpaepedrpr langvlh
```

Mauritania XP_033171498.1

```
1 mitrlyntee dpaycsfiwg snltssvdvl aanatsvfss dlrddfyrdv edprteslre
      61 ycyglvlpii camgiignvl nlvvltrrnm rgtayiymra ystaallaiv faipfgirml
     121 vhkdrgqwee fgpafytahl elylgngclg vgvmmllvlt ieryvsvchp gfarpvmgpp
     181 gvvvfltcla tvivylpsif rgelikcilg ssdvyvylrr dntiyqqtif yrvykimlev
     241 ifklvptlvi gglnmrimmv yrrtcerrrk mvlsrphaqg hghghghghg hghghahghg
     301 ylkdddprkf aeerrlflll gstsilflvc vspmailhmt iasevypsfp fqvfrasanl
     361 lelinysltf yiyclfsedf rntlvrtikw pwlkgkfchq aehevsaspp atagtvavag
     421 tgtghvsnfh paipaltltp aepderprca ngvlh
```

Sechellia XP_002039256.1

```
    1 mitrlyntee dpaycsfiwg snltssvdvl aanatsvfss dlrddfyrdv edprteslre
      61 ycyglvlpii camgiignvl nlvvltrrnm rgtayiymra ystaallaiv faipfgirml
     121 vhkdrgqwee fgpafytahl elylgngclg vgvmmllvlt ieryvsvchp gfarpvmgpp
     181 gvvvfltcla tvivylpsif rgelikcilg ssdvyvylrr dntiyqqtif yrvykimlev
     241 ifklvptlvi gglnmrimmv yrrtcerrrk mvlsrshaqg hghghghghg hahghgylkd
     301 ddprkfaeer rlflllgsts ilflvcvspm ailhmtiase vypsfpfqvf rasanllelí
     361 nysltfyiyc lfsedfrntl vrtikwpwlk gkfchqaehe vsasppatag tvavagtgtg
     421 hvsnfhpaip altltpaepd erprcangvl h
```

Serrata KAH8362383.1

```
  1 mitrlyntee dpaycsfiwg snlssttdvl aaagnsspvf tdlrddfyrd vedprteslr
      61 eycyglllpi icamgiignv lnlivltrrn mrgtayiymr aystaallai vfaipfgirm
     121 lvhkdrgqwe efgpafytah lelylgngcl gvgvmmllvl tieryvsvch pgfarpvmgp
     181 pgvvvfltcl atvivylpsi frgelikcil gssdvyvylr rdntiyqqti fyriykimle
     241 vifklvptvl igglnmrimm vyrrtcerrr qmvlsrnhgh ghghshgqgh aqghahtngy
     301 vkdddprkfa eerrlflllg stsilflvcv spmailhmti asevypsfpf qvfrasanll
     361 elinysltfy iyclfsedfr ntlvrtikwp wlkgkfchqa ehevsasppa tagtvavava
     421 vghgtgpvsn pqpipaipav tftpadpedh trpangvlr
```

Erecta XP_026837687.1

```
   1 mitrlyntee dpaycsfiwg snltssvdvl aanatsvfss dlrddfyrdv edprteslre
      61 ycyglvlpii camgiignvl nlivltrrnm rgtayiymra ystaallaiv faipfgirml
     121 vhkdrgqwee fgpafytahl elylgngclg vgvmmllvlt ieryvsvchp gfarpvmgpp
     181 gvvvfltcla tvivylpsif rgelikcilg ssdvyvylrr dntiyqqtif yrvykimlev
     241 ifklvptlvi gglnmrimmv yrrtcerrrq mvltrshayg hghghghgyl kdddprkfae
     301 errlflllgs tsilflvcvs pmailhmtia sevypsfpfq vfrasanlle linysltfyi
     361 yclfsedfrn tlvrtikwpw lkgkfchqge hevsasppat agtvavagig tgtgtgtgnv
     421 shfhpvipal tltpaepkdr hrcangvlh
```

Takahashi XP_017002450.2

```
  1 mitrlyntee dpaycsfiwg snltssadvl aanatsvfss dlrddfyrdv edprteslre
       61 ycyglvlpii camgiignvl nlivltrrnm rgtayiymra ystaallaiv faipfgirml
      121 vhkdrgqwee fgpafytahl elylgngclg vgvmmllvlt ieryvsvchp gfarpvmgpp
      181 gvvvfltcla tvivylpsif rgelikcifg ssdvyvylrr dntiyqqtif yrvykimlev
      241 ifklvptlvi gglnlrimmv yrrtcerrrq mvlsrphahg hghghghghg hghghghghg
      301 ymkdddprkf aeerrlflll gstsilflvc vspmailhmt iasevypsfp fqvfrasanl
      361 lelinysltf yiyclfsedf rntlvrtikw pwlkgkfchq aehevsaspp atagtvavag
      421 lgtgtgigqv snfhpaipal tltpaepeer prlangvlh
```

Biarmipes XP_016962943.1

```
  1 mitrlyntee dpaycsfiwg snltssaevl aanatsifss dlrddfyrdv edprteslre
       61 ycyglvlpii camgiignvl nlivltrrnm rgtayiymra ystaallaiv faipfgirml
      121 vhkdrgqwee fgpafytahl elylgngclg vgvmmllvlt ieryvsvchp gfarpvmgpp
      181 gvvvfltcla tvivylpsif rgelikcifg ssdvyvylrr dntiyqqtif yrvykimlev
      241 ifklvptlvi gglnmrimmv yrrtcerrrq mvlsrphahg ymkdddprkf aeerrlflll
      301 gstsilflvc vspmailhmt iasevypsfp fqvfrasanl lelinysltf yiyclfsedf
      361 rntlvrtikw pwlkgkfchq aehevsaspp atagtvavag ivagtgpgtg tksnfhpaip
      421 altltpaepe drprlangvl h
```

Eugracilis XP_017074309.1

```
1 mitslyttqe dpaycsfiwg snlttsadvl tanatsvfss dlrddfyrdv edprteslre
       61 ycyglvlpii camgiignvl nlivltrrnm rgtayiymra ystaallaiv faipfgirml
      121 vhkdrgqwek fgpafytahl elylgngclg igvmmllvlt ieryvsvchp gfarpvmgpp
      181 gvvvfltcla tvivylpsif rgelikcmyg srdvyvylrr dntiyqqtif yrvykimlev
      241 ifkliptlvi gglnmrimmv yrrtcerrrq mvlsrphahg ymkdddprkf aeerrlflll
      301 gstsilflvc vspmailhmt iasevypsfp fqvfrasanl lelinysltf yiyclfsedf
      361 rntlvrtikw pwlkgklchq aenevsaspp atagtvavaa gtgtghisky haasipaltl
      421 tpaepaedrr priangvhh
```

Rhopaloa XP_016979814.1

```
1 mitrlyntee dpaycsfiwg anltssadvl anatsvfntd lrddfyrdve dprteslrey
       61 cyglvlpiic amgiignvfn livltrrnmr gtayiymray staallaivf aipfgirmlv
      121 hkdrgqweef gpafytahle lylgngclgv gvmmllvlti eryvsvchpg farpvmgppg
      181 vvvfftclat vivylpsifr gelikcifgs sdafvylrrd ntiyqqtify rvykimlevi
      241 fklvptlvig glnmrimmvy rrtcerrrqm vlsrphahah ghghahgqgh ghshgylkdd
      301 dprkfaeerr lflllgstsi lflvcvspma ilhmtiasev ypsfpfqvfr asanllelin
      361 ysltfyiycl fsedfrntlv rtikwpwlkg kcchqaehev sasppatagt vavavavagt
      421 gtapghvtnf npaipalilt paeheerpir langvlh
```

Fichsuphila XP_017042138.2

```
1 mitrlyntee dpaycsfiwg gsnltataae vlaanatsif gtdlrddfyr dvedprtesl
       61 reycyglllp iicamgiign llnlvvltrr nmrgtayiym raystaalla ivfaipfgir
      121 mlvhkdrgqw eefgpafyta hlelylgngc lgvgvmmllv ltieryvsvc hpgfvrpvmg
      181 ppgvvvfltc latvivylps ifrgelikcv lhasdvyvyl rrdntiyqqt ifyrvykiml
      241 evifklvptl vigflnmrim mvyrrtcerr rqmvlsrpha hgylkdddpr kfaeerrlfl
      301 llgstsilfl vcvspmailh mtiasevyps fpfqvfrasa nllelinysl tfyiyclfse
      361 dfrntlvrti kwpwlkgkfc hqvehevsas ppatagtvav agtgtgaghv syyhpaipsl
      421 tftpaepeyr ppvcqwptsl swqrdntnit
```

Elegans XP_017113339.1

```
1 mitrlyntee dpaycsfiwg snltssadvl anatsafssd mrddfyrdve dprteslrey
       61 cyglvlpiic amgiignvln livltrrnmr gtayiymray staallaivf aipfgirmlv
      121 hkdrgqweef gpafytahle lylgngclgv gvmmllvlti eryvsvchpg farpvmgppg
      181 vvvfltclat vivylpsifr gelikcifgs sdvfvylrrd ntiyqqtify rvykimlevi
      241 fklvptlvig glnmrimmvy rrtcerrrqm vlsrphghgh ghghghghgh gaqghghahg
      301 ghgylkdddp rkfaeerrlf lllgstsilf lvcvspmail hmtiasevyp sfpfqvfras
      361 anllelinys ltfyiyclfs edfrntlvrt ikwpwlkgkf chqgehevsa sppatagtva
      421 vavaatgtas ghvsnlhpai paltltpaep edrdrpnrla ngvlh
```

Kikkawei KAH8343513.1

```
1 mitrlyntee dpaycsfiwg snlssttdvl aaagnsspvf tdlrddfyrd vedprteslr
       61 eycyglllpi tcamgiignv lnlivltrrn mrgtayiymr aystaallai vfaipfgirm
```

121 lvhkdrgqwe efgpafytah lelylgngcl gvgvmmllvl tieryvsvch pgfarpvmgp
181 pgvvvfltcl atvivylpsi frgelikcil gssdvyvylr rdntiyqqti fyriykimle
241 vifklvptvl igglnmrimm vyrrtcerrr qmvlsrnhgh ghghshaqgq aqghahtngy
301 vkdddprkfa eerrlflllg stsilflvcv spmailhmti asevypsfpf qvfrasanll
361 elinysltfy iyclfsedfr ntlvrtikwp wlkgkfchqa ehevsasppa tagtvavava
421 vghgtgpvsn pqpipaipav tftpadpedh arppngvlr

Bipectinate XP_017097721.2
1 mitrlyhtee dpaycsfigg snlssssdvl atsanatpvf gsdlrddfyr dvedprtesl
61 reycyglllp iicamgiign vlnlivltrr nmrgiayiym raystaalla ivfaipfgir
121 mlvhkdrgqw eefgpafyta hlelylgngc lgvgvmmllv ltieryvsvc hpgfsrpvmg
181 ppgvvvfftc latvivylps ifrgelikcm lgssdvyvyl rrdntiyqqt lfyrvykiml
241 evifkliptv ligglnmrim mvyrrtcerr rqmvlsrpqa hhhhhhnang pgyvkdddpr
301 kfaeerrlfl llgstsilfl vcvspmailh mtiasevyps fpfqvfrasa nllelinysl
361 tfyiyclfse dfrntlvrti kwpwlkgkfc hqaehevsas ppatagtvaa agtattatan
421 pspaipanpa pilptdphge eperlangtv r

Anannassae XP_044573352.1
1 mitrlyhtee dpaycsfigg snisisssss sisssdvlat sanatpvfgs dlrddfyrdv
61 edprteslre ycygllllpii camgiignvl nlivltrrnm rgiayiymra ystaallaiv
121 faipfgirml vhkdrgqwee fgpafytahl elylgngclg vgvmmllvlt ieryvsvchp
181 gfsrpvmgpp gvvvfftcla tvivylpsif rgelikcmlg ssdvyvylrr dntiyqqtlf
241 yrvykimlev ifkliptvli gglnmrimmv yrrtcerrrq mvlsrpqahh hqngpgyvkd
301 ddprkfaeer rlflllgsts ilflvcvspm ailhmtiase vypsfpfqvf rasanlleli
361 nysltfyiyc lfsedfrntl vrtikwpwlk gklchqadqh evsasppata gtvaagigta
421 patanpspai panpapilpt dhphgekspq ripngtvr

Mojavensis XP_015016833.1
1 merivrnhqs ktmitrlynt eedpaycsvi wganltsssd lvlgtatnat hiyandlrdd
61 lyadvedprt ealreycygl mlpiicalgi ignvlnlivl trrnmrgtay iymraystaa
121 llaivfaipf girmlvhkdr gqweefgpaf ytahlelflg ngclgvgvmm llvltieryv
181 svchpgftrp vmgppgvvvf vtclvtfiiy lpsifrgeli kcmltsnnvy vylrrdnniy
241 qrtifysvyk imlevifklv ptvviaglnl rimlvyrrtc errrqmvlsr atyvkdddpr
301 kfaeerrlfl llgstsilfl lcvspmailh mtiasevlps fpfqvframa nllelinysi
361 tfyiyclfse dfrntlmrti kwpwlkgklc hqvdettqai mgvpmvrfek vnmrnhhitt
421 tsgigrsssi

Virilism XP_032295448.1
1 mitrlyntee dpaycsfiwg anltssndlv lgttansthi yandlrddly advedprtes
61 lreycyglml pvicalgiig nvlnlivltr rnmrgtayiy mraystaall aivfaipfgi
121 rmlvhkdrgq weefgpafyt ahlelflgng clgvgvmmll vltieryvsv chpgftrpvm
181 gppgvvvflt cfatfiiylp sifrgelikc mltsnnvyvy lrrdnniyqr tifysvykim
241 levifklipt vliaglnlri mlvyrrtcer rrqmvltran yvkdddprkf aeerrlflll
301 gstsilfllc vspmailhmt iasevlpsfp fqvfralanl lelinysitf yiyclfsedf
361 rntlmrtikw pwlksklchq vdetqtikgv pmvrfdkvtl rnhhitttsg igrsssi

Grimshawi XP_032597329.1

1 mitrlyntee dpvycsfvwg anvsngrsha anssalypdd mrddwfadae dprtellrqy
61 cygyflpfic asgiignvln livltrrnmr gpsyiymray staallaivf aipfgirmlv
121 hkdrgqweei gpafytahle lflgngclgv gvmmllvlti eryvsvcrpg atrpaigqpg
181 vvifiicvlt fifylpsifr gelikcmlts knvyvylrrd niiyqqtlfy svykvlveii
241 fkiiptfiia glnlrimwvy rrtcarrrqm vltraiyakn edprkfaeer rlflllgsts
301 ilfllcispm ailhmtiase vlpsfpfqvf ralanlleli nysitfyiyc lfsedfrntl
361 lrtfnwpwvk sklfrrqvde vsasppatat aavcnpvcpt ihiehsnhpe hangpfrkgq
421 ya
