# Supplementary material for: The incidence of candidate binding sites for β-arrestin in Drosophila neuropeptide GPCRs
Source: PLoS One. 2022 Nov 1;17(11):e0275410. doi: 10.1371/journal.pone.0275410 (PMC9624432; doi:10.1371/journal.pone.0275410)
Supplement: S19 Text — (PDF) [file pone.0275410.s023.pdf]

***S19. Text*** **Multi-species analysis of PDF-R PD isoforms**
**Supporting Figure 22**

CLUSTAL Line-ups; Genbank Reference IDs below
5th, 6th and 7th Predicted TM domain in YELLOW
BBS sequences in RED

```
Serrata           MTLLSSILDSGG--------GISAQRFTRLLRQSSTSSSSSSSS---------SASASGS       43
Kikkawei          MTLLSSILDSGG--------SISAQRFTRLLRQSSTSSSS-------------SVSASAS       39
Rhopaloa          MTLLSNILDCGG--------GISAQCLTRSLRESSSPGS-P-------GSPESGAFESKS       44
Elegans           MTLLSNILDCGG--------GIPAQRLARLLRQSSSSGSTP-------SGSASGPFESKS       45
Fichsuphila       MTLLASILDCGG--------GISAQRFTRLLAQSSLLASSSASASASASASASGTFASES       52
Takahashi         MTLLSNILDCGG--------GISAQRFARLLRQSSSSGSPSASASSSASSSSGTSFESKS       52
Suzuki            MTLLSNILDCGG--------GISAQRFTRLLRQSSSSGSPSS-------SSSGTTFESKS       45
Biarmipes         MTLLSNILDCGG--------GISAQRFTRLLRQSSSPGFLSS-------SSLGTTFESKS       45
Eugracilis        MTLLSNILDSGG--------GISAQRFTRLLRQSSSSGSSSASAS----ASSYGTLESKS       48
Erecta            MTLLSNILDCGG--------CISAQRFTRLLRQSGSSVP---SPS----APAPGTFESIS       45
Melanogaster      MTLLSNILDCGG--------CISAQRFTRLLRQSGSSGP---SPS----APTAGTFESKS       45
Sechellia         MTLLSNILDCGG--------CISDQRFTHLLRQSGSSGP---SPS----APAPGTFESKS       45
Simulans          ------------------------------------------------------------       0
Mauritania        MTLLSNILDCGG--------CISAQRFTRLLRQSGSSGP---SPS----APAPGTFESKS       45
Bipectinate       MTLLSSILDSGG--------SVSVQRLTRLLRQSSPSASA-----------SGTALASES       41
Anannassae        MTLLSSILDSGG--------SVSVQRLTRLLRQSSTSSSA-----------SGPSLASDS       41
Mojavensis        -MQLAGDSEAGSMPTIITTSTSSNSFLQRIALKAVTAATTTKDR---------L----IS       46
Virilism          ------------------------------------------------------------       0
Grimshawi         MMQLPGINKPGCIPTISTSSIIIPQ---RFARRAARVASTSTAT---------VTTLSMS       48

Serrata           MLEPTSSQLPINDVLGGGRIPFLHDNGTGES----------LPLPDADALDPNFVLDGV-       92
Kikkawei          MLEPTSSQLPNNDVLGGGRIPFLPGNATGESSSSSSPPLPSPSLPDADALDPNFVLDGV-       98
Rhopaloa          MLEPTSSHI-LPT----GRVPVLHDFNASS---------------TDSP--GTFLLDGV-       81
Elegans           MLEPTTSHI-LPT----GRVPILHDFGSSS---------------TESPLTGTYLLDGV-       84
Fichsuphila       MLEPTSSHI-LPT----GRVPVLHGFDST----------------TTESTTSTYVLDGV-       90
Takahashi         MLEPTSSQN-LPT----GRVPILHDFDFDFDPT------------TTTESPGNYVLDGV-       94
Suzuki            MLEPTSSHI-LPT----GRVPILHDFDFDS---------------LTTESTGTHVLDGV-       84
Biarmipes         MLEPTSSHV-LPT----GRVPVLHDFDFDS---------------STTELPRILVLDGV-       84
Eugracilis        MLEPTSSHT-LPG----GRVPILHDFDS-----------------STTESPGAYLLDGV-       85
Erecta            MLEPTSLHS-LPT----GRVPLLHDFDA-----------------STTESPGTYVLDGV-       82
Melanogaster      MLEPTSSHS-LAT----GRVPLLHDFDA-----------------STTESPGTYVLDGV-       82
Sechellia         MLEPTSPHS-LAT----GRVPLLHDFDA-----------------STTESPGTYVLDGV-       82
Simulans          --------------------PLLHDFDA-----------------STTESPGTYVLDGV-       22
Mauritania        MLEPTSSHS-LAT----GRVPLLHDFDA-----------------TTTESPGTHVLDGV-       82
Bipectinate       MVEPTASQM-LNEITDGRRVPSL---GQDLATT------------TETTGQSNVIIDRV-       84
Anannassae        ISSMVE--------ATSLRVPSL---GHDLSTT------------T----ETNVIIDRV-       73
Mojavensis        IVETDSPA--GS-----H-MDP--STAMTTT--S--ITATATATPAATATA-S--ELSRS       89
Virilism          MPT-AAPS--SN-----RTVSTHHSTTMTSTSTT--YGTTSTTQAALTS------FE---       41
Grimshawi         MTQSGRSM--LN-----RTMTSIATATTTSTSTS--ISTHSTTSSSTSKLP-SQVISGLA       98

Serrata           --TSVAKMALEATVK-EVLRDPDPEQILANANATAPWNITL--ASAAATNYENCSALFAN       147
Kikkawei          --TSVAKMALEATVK--VLRDPDPEQILANANASAPWNITL--AAAAATNYENCSALFAN       152
Rhopaloa          --VSVAQMALEPTLT-DLLPDPDPDQVLSNLNASAPWNLTL--ASAAATNFENCSALFAN       136
Elegans           --ASVAEMALEPTVR-DILSDPDPDKVLSNLNASAPWNLTL--ASSAATNFENCSALFAN       139
Fichsuphila       --ASVAQMPLLESTVMDALPDSDPDQAFGNLNVTAPWNLTL--ASAAATNFENCSALFAN       146
Takahashi         --ASVAKMALEPTVMDTVLPDSDPDQVLSNLNVSAPWNLTL--ASAAATNFENCSALFVN       150
Suzuki            --ASVAKMALEPTVMDAVLPDSDTDQVLSNLNISAPWNITL--ASAAATNFENCSSLFVN       140
Biarmipes         --AGVATMALEPTAMDAVLPDSDPDQVLSNFNISAPWNITL--ASAAATNFENCSSLFVN       140
Eugracilis        --ASVAKMALEPAVMD-VLPDPDTDQVLSNLNITAPWNLTL--ASAAATNFENCSALFVN       140
Erecta            --ARVAQLALEPTVMD-GLPDPDTEQVLSNLNSSAPWNLTL--SSAAATNFENCSALFVN       137
Melanogaster      --ARVAQLALEPTVMD-ALPDSDTEQVLGNLNSSAPWNLTL--ASAAATNFENCSALFVN       137
Sechellia         --ARVAQLALEPTVMD-ALPDPDTEQVLGNLNSSAPWNLTL--ASAAATNFENCSALFVN       137
Simulans          --ARVAQLALEPTVMD-ALPDPDTEQVLGNLNSSAPWNLTL--ASAAATNFENCSALFVN       77
Mauritania        --ARVAQLALEPTVMD-ALPDPDTEQVLGNLNSNAPWNLTL--ASAAATNFENCSALFVN       137
Bipectinate       --TNVAKMALEATVMD--------DQVLAGVNASAHWNMT--LSSSTATNYENCSALFAN       132
Anannassae        --TNVAKMALEATVMDV-LPDPEPDQVLTGVNASSHWNMNMTLSSASATNYENCSALFAN       130
Mojavensis        DVLNASIEATTAVGMDTVKTATESGLPTVSGTTLPSWNSSF--NSALANNYDNCSAMFAN       147
```

```
Virilism        ------PMAVTAAGMDV---TMAGTEPLSSTSIPTFWNSSI--NTASANSYDNCSALFAN        90
Grimshawi       DVINGTELDMTAAATAV---GLDAAVP-DPSTTSSSWNSTF--STASANSYDNCAAMFAN        152
                                               .    ** .    :: *..::**:::*.*

Serrata         YTLPQTGLYCNWTWDTLLCWPPTPAGVLARMNCPGGYHGVDTRKFANRKCELDGRWGSRP        207
Kikkawei        YTLPQTGLYCNWTWDTLLCWPPTPAGVLARMNCPGGYHGVDTRKFANRKCELDGRWGSRP        212
Rhopaloa        YTLPQTGLYCNWTWDTLLCWPPTPAGVLARMNCPAGFHGVDTRKFAIRKCELDGRWGSRP        196
Elegans         YTLPQTGLYCNWTWDTLLCWPPTPAGVLARMNCPGGFHGVDTRKFAIRKCELDGRWGSRP        199
Fichsuphila     YTLPQSGLYCNWTWDTLLCWPPTPAGVLARMNCPGGFHGVDTRKFAIRKCELDGRWGSRP        206
Takahashi       YTLPQTGLYCNWTWDTLLCWPPTPAGVLARMNCPAGFHGVDTRKFAIRKCELDGRWGSRP        210
Suzuki          YTLPQSGLYCNWTWDTLLCWPPTPAGVLARMNCPGGFHGVDTRKFAIRKCELDGRWGSRP        200
Biarmipes       YTLPQTGLYCNWTWDTLLCWPPTPAGVLARMNCPGGFHGVDTRKFAIRKCELDGRWGSRP        200
Eugracilis      YTLPQTGLYCNWTWDTLLCWPPTPAGVLARMNCPGGFHGVDTRKFAIRKCELDGRWGSRP        200
Erecta          YTLPQTGLYCNWTWDTLLCWPPTPAGVLARMNCPGGFHGVDTRKFAIRKCELDGRWGSRP        197
Melanogaster    YTLPQTGLYCNWTWDTLLCWPPTPAGVLARMNCPGGFHGVDTRKFAIRKCELDGRWGSRP        197
Sechellia       YTLPQTGLYCNWTWDTLLCWPPTPAGVLARMNCPGGFHGVDTRKFANRKCELDGRWGSRP        197
Simulans        YTLPQTGLYCNWTWDTLLCWPPTPAGVLARMNCPGGFHGVDTRKFAIRKCELDGRWGSRP        137
Mauritania      YTLPQTGLYCNWTWDTLLCWPPTPAGVLARMNCPGGFHGVDTRKFAIRKCELDGRWGSRP        197
Bipectinate     YTLPQTGLYCNWTWDTLLCWPPTPAGVLARMHCPGGYHGVDTRKFANRKCELDGRWGSRP        192
Anannassae      YTLPQTGLYCNWTWDTLLCWPPTPAGVLARMNCPGGYHGVDTRKFANRKCELDGRWGSRP        190
Mojavensis      YTHPQTGLYCNWTWDSLLCWPPTPAGNLARMNCPAGYHGVDTRKFANRKCELDGHWGGRP        207
Virilism        YTQPTTVIYCNWTWDSLLCWPPTPAGATAHMHCPAGYHGVDTRKFANRKCELDGHWAGRP        150
Grimshawi       YTQPVTGLYCNWTWDSLLCWPPTPAGVMARMYCPAGYHGVDTRKFAIRKCELDGHWGRRP        212
                ** * : :*******:**********  *:* **.*:********* *******:*. **

Serrata         NATETNATGWTDYGPCYKPEVIRLMQQMGSREDLDLYIEIARRTRTLEIVGLCLSLFALI        267
Kikkawei        NATETNATGWTDYGPCYKPEVIRLMQQMGSREDLDLYIEIAKRTRTLEIVGLCLSLFALI        272
Rhopaloa        NATEVSPPGWTDYGPCYKPEIIRLMQQMGSR-DFDTFIDIAKKTRTLEIVGLCLSLFALI        255
Elegans         NATEVSPPGWTDYGPCYKPEIIRLMQQMGSK-DFDLYIDIARKTRTLEIVGLCLSLFALI        258
Fichsuphila     NATEVSPPGWTDYGPCYKPEIIRLMQQMGNK-DFDLYIDIARKTRTLEIVGLCLSLFALI        265
Takahashi       NATEVSPPGWTDYGPCYKPEIIRLMEQMGSK-NFDLYIDIAWKTRTLEIVGLCLSLFALI        269
Suzuki          NATEVSPPGWTDYGPCYKPEIIRLMQQMGSK-DFDLYIDIARKTRTLEIVGLCLSLFALI        259
Biarmipes       NATEVSPPGWTDYGPCYKPEIIRLMQQMGSK-DFDLYIDIARKTRTLEIVGLCLSLFALI        259
Eugracilis      NATEASPPGWTDYGPCYKPEIIRLMQQMGNK-DFDLYIDIARKTRTLEIVGLCLSLFALI        259
Erecta          NATEVSPPGWTDYGPCYKPEIIRLMQQMGSK-DFDAYIDIARRTRTLEIVGLCISLFALI        256
Melanogaster    NATEVNPPGWTDYGPCYKPEIIRLMQQMGSK-DFDAYIDIARRTRTLEIVGLCLSLFALI        256
Sechellia       NATEVSPPGWTDYGPCYKPEIIRLMQQMGSK-DFDAYIDIARRTRTLEIVGLCLSLFALI        256
Simulans        NATEVSPPGWTDYGPCYKPEIIRLMQQMGSK-DFDAYIDIARRTRTLEIVGLCLSLFALI        196
Mauritania      NATEVSPPGWTDYGPCYKPEIIRLMQQMGSK-DFDAYIDIARRTRTLEIVGLCLSLFALI        256
Bipectinate     NATEPSPAGWTDYGPCYKPEVIRLMQSMGSK-DIDIYIDIARRTRTLEIVGLWVSLFALV        251
Anannassae      NATEPSPAGWTDYGPCYKPEVIRLMQSMGSK-DIDIYIDIARRTRTLEIVGLWVSLFALV        249
Mojavensis      NETEPNHAGWTDYGPCYKPEVIRLMQEIK---DVNLYIDIAQRTRTLEIIGLCLSLLALI        264
Virilism        NSTEQKPTGWTDYGPCYKPEVIRLMQEIK---DVNLYMDIAQRTRTLEIIGLCLSLFALI        207
Grimshawi       NDTELNTGGWTDYAPCYKPEVIRLMQEIK---DVNVYMDIAQRTRTLEIIGLCLSLLALI        269
                * ** .  *****.******:****:.:    :.: :::** :******:** :**:**:

Serrata         VSLLIFCTFRSLRNNRTKIHKNLFVAMVLQVIIRLTLYLDQFRRGSKEAATNTSLSVIEN        327
Kikkawei        VSLLIFCTFRSLRNNRTKIHKNLFIAMVLQVIIRLTLYLDQFRRGNKEAATNTSLSVIEN        332
Rhopaloa        VSLLIFCTFRSLRNNRTKIHKNLFVAMVLQVIIRLTLYLDQYRRGNKEAATNTSLSVIEN        315
Elegans         VSLLIFCTFRSLRNNRTKIHKNLFVAMVLQVIIRLTLYLDQYRRGNKEAATNTSLSVIEN        318
Fichsuphila     VSLLIFCTFRSLRNNRTKIHKNLFVAMVLQVIIRLTLYLDQFRRGNKEAATNTSLSVIEN        325
Takahashi       VSLLIFCTFRSLRNNRTKIHKNLFVAMVLQVIIRLTLYLDQYRRGNKEAATNTSLSVIEN        329
Suzuki          VSLLIFCTFRSLRNNRTKIHKNLFVAMVLQVIIRLTLYLDQFRRGNKEAATNTSLSVIEN        319
Biarmipes       VSLLIFCTFRSLRNNRTKIHKNLFVAMVLQVIIRLTLYLDQFRRGNKEAATNTSLSVIEN        319
Eugracilis      VSLLIFCTFRSLRNNRTKIHKNLFVAMVLQVIIRLTLYLDQFRRGNKEAATNTSLSVIEN        319
Erecta          VSLLIFCTFRSLRNNRTKIHKNLFVAMVLQVIIRLTLYLDQFRRGNKEAATNTSLSAIEN        316
Melanogaster    VSLLIFCTFRSLRNNRTKIHKNLFVAMVLQVIIRLTLYLDQFRRGNKEAATNTSLSVIEN        316
Sechellia       VSLLIFCTFRSLRNNRTKIHKNLFVAMVLQVIIRLTLYLDQFRRGNKEAATNTSLSVIEN        316
Simulans        VSLLIFCTFRSLRNNRTKIHKNLFVAMVLQVIIRLTLYLDQFRRGNKEAATNTSLSVIEN        256
Mauritania      VSLLIFCTFRSLRNNRTKIHKNLFVAMVLQVIIRLTLYLDQFRRGNKEAATNTSLSVIEN        316
Bipectinate     ISLLIFCTFRSLRNNRTKIHKNLFIAMVLQVIVRLTLYLDQYRRGNKEAATNTSLSAIEN        311
Anannassae      ISLLIFCTFRSLRNNRTKIHKNLFVAMVLQVIVRLTLYLDQYRRGNKEAATNTSLSAIEN        309
Mojavensis      ISLVIFCAFRSLRNNRTKIHKNLFIAMVLQVIVRLTLYLDQFRRGIQ---YNSSLSAIEN        321
Virilism        ISLMIFCAFRSLRNNRTKIHKNLFVAMVLQVIVRLTLYLDQFRRGKSDSANNTSLSVIEN        267
Grimshawi       ISLVIFCAFRSLRNNRTKIHKNLFVAMVLQVIVRLTLYLDQFRRGRPETATNASVSIIEN        329
                :**:***:****************:*******:********:***      *:*:* ***

Serrata         TPYLCEASYVLLEYARTAMFMWMFIEGLYLHNMVTVAVFQGSFPLKFFSRLGWCVPILMT        387
Kikkawei        TPYLCEASYVLLEYARTAMFMWMFIEGLYLHNMVTVAVFQGSFPLKFFSRLGWCVPILMT        392
Rhopaloa        TPYLCEASYVLLEYARTAMFMWMFIEGLYLHNMVTVAVFQGSFPLKFFSRLGWCVPILMT        375
Elegans         TPYLCEASYVLLEYARTAMFMWMFIEGLYLHNMVTVAVFQGSFPLKFFSRLGWCAPILMT        378
```

```
Fichsuphila       TPYLCEASYVLLEYARTAMFMWMFIEGLYLHNMVTVAVFQGSFPLKFFSRLGWCVPILMT      385
Takahashi         TPYLCEASYVLLEYARTAMFMWMFIEGLYLHNMVTVAVFQGSFPLKFFSRLGWGFPILMT      389
Suzuki            TPYLCEASYVLLEYARTAMFMWMFIEGLYLHNMVTVAVFQGSFPLKFFSRLGWGFPILMT      379
Biarmipes         TPYLCEASYVLLEYARTAMFMWMFIEGLYLHNMVTVAVFQGSFPLKFFSRLGWGFPILMT      379
Eugracilis        TPYLCEASYVLLEYARTAMFMWMFIEGLYLHNMVTVAVFQGSFPLKFFSRLGWCVPILMT      379
Erecta            TPYLCEASYVLLEYARTAMFMWMFIEGLYLHNMVTVAVFQGSFPLKFFSRLGWCVPILMT      376
Melanogaster      TPYLCEASYVLLEYARTAMFMWMFIEGLYLHNMVTVAVFQGSFPLKFFSRLGWCVPILMT      376
Sechellia         TPYLCEASYVLLEYARTAMFMWMFIEGLYLHNMVTVAVFQGSFPLKFFSRLGWCVPILMT      376
Simulans          TPYLCEASYVLLEYARTAMFMWMFIEGLYLHNMVTVAVFQGSFPLKFFSRLGWCVPILMT      316
Mauritania        TPYLCEASYVLLEYARTAMFMWMFIEGLYLHNMVTVAVFQGSFPLKFFSRLGWCVPILMT      376
Bipectinate       TPYLCEASYVLLEYARTAMFMWMFIEGLYLHNMVTVAVFQGSFPLKFFSRLGWCVPILMT      371
Anannassae        TPYLCEASYVLLEYARTAMFMWMFIEGLYLHNMVTVAVFQGSFPLKFFSRLGWCVPILMT      369
Mojavensis        TPYLCEASYVLLEYARTAMFMWMFIEGLYLHNMVTVAVFQGSFPLIFFSLLGWGMPVVMT      381
Virilism          TPYLCEASYVLLEYARTAMFMWMFIEGLYLHNMITVAVFQGNFPLVFFSLLGWGMPVLMT      327
Grimshawi         TPYLCEASYVLLEYARTAMFMWMFIEGLYLHNMVTVAVFQGNFPLKLFALLGWGLPVLMT      389
                  *********************************:*******.*** :*: ***  *::**

Serrata           TVWARCTVIYMDTSLGDCLWNYNLTPYYWILEGPRLAVILLNFCFLVNIIRVLVMKLRQS      447
Kikkawei          TVWARCTVIYMDTSLGDCLWNYNLTPYYWILEGPRLAVILLNFCFLVNIIRVLVMKLRQS      452
Rhopaloa          TVWARCTVMYMDTSLGDCLWNYNLTPYYWILEGPRLAVILLNFCFLVNIIRVLVMKLRQS      435
Elegans           TVWARCTVMYMDTSLGECLWNYNLTPYYWILEGPRLAVILLNFCFLVNIIRVLVMKLRQS      438
Fichsuphila       TVWARCTVMYMDTSLGECLWNYNLTPYYWILEGPRLAVILLNFCFLVNIIRVLVMKLRQS      445
Takahashi         TVWARCTVMYMDTSLGECLWNYNLTPYYWILEGPRLAVILLNFCFLVNIIRVLVMKLRQS      449
Suzuki            TVWARCTVMYMDTSLGECLWNYNLTPYYWILEGPRLAVILLNFCFLVNIIRVLVMKLRQS      439
Biarmipes         TVWARCTVMYMDTSLGECLWNYNLTPYYWILEGPRLAVILLNFCFLVNIIRVLVMKLRQS      439
Eugracilis        TVWARCTVMYMDTTLGECLWNYNLTPYYWILEGPRLAVILLNFCFLVNIIRVLVMKLRQS      439
Erecta            TVWARCTVMYMDTSLGECLWNYNLTPYYWILEGPRLAVILLNFCFLVNIIRVLVMKLRQS      436
Melanogaster      TVWARCTVMYMDTSLGECLWNYNLTPYYWILEGPRLAVILLNFCFLVNIIRVLVMKLRQS      436
Sechellia         TVWARCTVMYMDTSLGECLWNYNLTPYYWILEGPRLAVILLNFCFLVNIIRVLVMKLRQS      436
Simulans          TVWARCTVMYMDTSLGECLWNYNLTPYYWILEGPRLAVILLNFCFLVNIIRVLVMKLRQS      376
Mauritania        TVWARCTVMYMDTSLGECLWNYNLTPYYWILEGPRLAVILLNFCFLVNIIRVLVMKLRQS      436
Bipectinate       FVWARCTVMYMDTSMGECLWNYNLTPYYWILEGPRLAVILLNFCFLVNIIRVLVVKLRQS      431
Anannassae        FVWARCTVMYMDTSMGECLWNYNLTPYYWILEGPRLAVILLNFCFLVNIIRVLVVKLRQS      429
Mojavensis        FVWVQCTAIFMDTALGDCMWNYNLTPYYWILEGPRLAVILLNFFFLVNIIRVLVVKLRQS      441
Virilism          FVWVQCTAIFMDTSLGDCLWNYNLTPYYWILEGPRLTVIMLNFFFLVNIIRVLVMKLRQS      387
Grimshawi         FVWVQCTAIFMDTTVGECMWNYNLTPYYWILEGPRLAVILLNFFFLVNIIRVLVVKLRQS      449
                   **.:**.::***::*:*:*****************:**:*** **********:*****

Serrata           QASDIEQTRKAVRAAIVLLPLLGITNILHQVAPLKTATNFAVWSYSTYFLTSFQGFFIAL      507
Kikkawei          QASDIEQTRKAVRAAIVLLPLLGITNILHQLAPLKTATNFAVWSYSTYFLTSFQGFFIAL      512
Rhopaloa          QASDIEQTRKAVRAAIVLLPLLGITNILHQMAPLKTATNFAVWSYGTHFLTSFQGFFIAL      495
Elegans           QASDIEQTRKAVRAAIVLLPLLGITNLLHQLAPLKTATNFAVWSYGTHFLTSFQGFFIAL      498
Fichsuphila       QASDIEQTRKAVRAAIVLLPLLGITNLLHQLAPLKTATNFAVWSYGTHFLTSFQGFFIAL      505
Takahashi         QASDIEQTRKAVRAAIVLLPLLGITNILHQMAPLKTATNFAVWSYVTHFLTSFQGFFIAL      509
Suzuki            QASDIEQTRKAVRAAIVLLPLLGITNLLHQLAPLKTATNFAVWSYGTHFLTSFQGFFIAL      499
Biarmipes         QASDIEQTRKAVRAAIVLLPLLGITNLLHQLAPLKTATNFAVWSYGTHFLTSFQGFFIAL      499
Eugracilis        QASDIEQTRKAVRAAIVLLPLLGITNLLHQLAPLKTATNFAVWSYGTHFLTSFQGFFIAL      499
Erecta            QASDIEQTRKAVRAAIVLLPLLGITNILHQLAPLKTATNFAVWSYGTHFLTSFQGFFIAL      496
Melanogaster      QASDIEQTRKAVRAAIVLLPLLGITNLLHQLAPLKTATNFAVWSYGTHFLTSFQGFFIAL      496
Sechellia         QASDIEQTRKAVRAAIVLLPLLGITNLLHQLAPLKTATNFAVWSYGTHFLTSFQGFFIAL      496
Simulans          QASDIEQTRKAVRAAIVLLPLLGITNLLHQLAPLKTATNFAVWSYGTHFLTSFQGFFIAL      436
Mauritania        QASDIEQTRKAVRAAIVLLPLLGITNLLHQLAPLKTATNFAVWSYGTHFLTSFQGFFIAL      496
Bipectinate       QASDIEQTRKAVRAAIVLLPLLGITNLLHQLAPLKTATNFAVWSYGTHFLTSFQGFFIAL      491
Anannassae        QASDIEQTRKAVRAAIVLLPLLGITNLLHQLAPLKTATNFAVWSYGTHFLTSFQGFFIAL      489
Mojavensis        QASDIEQTRKAVRAAIVLLPLLGITNLLHLVPALKTAWKFAIWSYVTHFLTSFQGFFIAL      501
Virilism          QASEIEQTRKAVRAAIVLLPLLGITNLLHLVPALKTAWKFAIWSYVTHFLTSFQGFFIAL      447
Grimshawi         QASDIEQTRKAVRAAIVLLPLLGITNLLHLVPALKTAWKFAVWSYVTHFLTSFQGFFIAL      509
                  ***:**********************:** :  **** :**:*** *:************

Serrata           IYCFLNGEVRAVLLKSLATQLSVRGHPEWAPKRASMYSGAYNTAPDTDAVQ-QHQQTGEN      566
Kikkawei          IYCFLNGEVRAVLLKSLATQLSVRGHPEWAPKRASMYSGAYNTAPDTDAVLQQQQQPGEN      572
Rhopaloa          IYCFLNGEVRAVLLKSLATQLSVRGHPEWAPKRASMYSGAYNTAPDTDAV----QPAGDP      551
Elegans           IYCFLNGEVRAVLLKSLATQLSVRGHPEWAPKRASMYSGAYNTAPDTDAV----QPAGDP      554
Fichsuphila       IYCFLNGEVRAVLLKSLATQLSVRGHPEWAPKRASMYSGAYNTAPDTDAV----HPAGDP      561
Takahashi         IYCFLNGEVRAVLLKSLATQLSVRGHPEWAPKRASMYSGAYNTAPDTDAV----QPAGDP      565
Suzuki            IYCFLNGEVRAVLLKSLATQLSVRGHPEWAPKRASMYSGAYNTAPDTDAV----QPAGDP      555
Biarmipes         IYCFLNGEVRAVLLKSLATQLSVRGHPEWAPKRASMYSGAYNTAPDTDAV----QPAGDP      555
Eugracilis        IYCFLNGEVRAVLLKSLATQLSVRGHPEWAPKRASMYSGAYNTAPDTDAV----QPAGDP      555
Erecta            IYCFLNGEVRAVLLKSLATQLSVRGHPEWAPKRASMYSGAYNTAPDTDAV----QPAGDP      552
Melanogaster      IYCFLNGEVRAVLLKSLATQLSVRGHPEWAPKRASMYSGAYNTAPDTDAV----QPAGDP      552
Sechellia         IYCFLNGEVRAVLLKSLATQLSVRGHPEWAPKRASMYSGAYNTAPDTDAV----QPVGDP      552
```

```
Simulans          IYCFLNGEVRAVLLKSLATQLSVRGHPEWAPKRASMYSGAYNTAPDTDAV----QPAGDP        492
Mauritania        IYCFLNGEVRAVLLKSLATQLSVRGHPEWAPKRASMYSGAYNTAPDTDAV----QPAGDP        552
Bipectinate       IYCFLNGEVRAVLLKSLATQMSVRGHPEWVPKRASMYSGAYNTAPDTDAVI---QQPGDN        548
Anannassae        IYCFLNGEVRAVLLKSLATQMSVRGHPEWVPKRASMYSGAYNTAPDTDAVI---QQPGEN        546
Mojavensis        IYCFLNGEVRAVLLKSLAVWMSVRGHPEWVPKRASMYSAAYNTAPDTEPPA---QQSVEA        558
Virilism          IYCFLNGEVRAVMLKSIAVWLSVRGHPEWAPKRPSMYSGAYNTAPDTDPQL---KQ-GDP        503
Grimshawi         IYCFLNGEVRTVLLKSLAVWMSVRGHPEWAPKRASMYSGAYNTAPDTDVVQ---QP-GEA        565
                  **********:*:***:*. :********.*** ****.********:      :   :

Serrata           PATGKRISPPNKRLNGRKASSASIVLIHEPQQRQRLIPRLQSKAREKDKSL--DKDRGKR        624
Kikkawei          PATGKRISPPNKRLNGRKASSASIVMIHEPQQRQRLIPRLRSRTREKDKSL--DKDREKR        630
Rhopaloa          LATGKRISPPNKRLNGRKPSSASIVMIHEPQQRQRLIPRLQNKAREKG--R--DRVEKAD        607
Elegans           SATGKRISPPHKRLNGRKPSSASIVMIHEPPQRQRLIPRLQSKAREKG--R--DRAERAD        610
Fichsuphila       SAPGKRISPPNKRLNGRKPSSASIVMIHEPQQRHRLIPRLQNKNQEKG--K--DRVEKAD        617
Takahashi         SATGKRISPPHKRLNGRKPSSASIVMIHEPQQRQRLIPRLQNKAREKS--R--DRVDKAD        621
Suzuki            SATGKRISPPHKRLNGRKPSSASIVMIHEPQQRHRLIPRLQNKEREKS--K--DRVEKAD        611
Biarmipes         LATGKRISPPHKRLNGRKPSSASIVMIHEPQQRHRLIPRLQNKAREKS--R--DRVDKAD        611
Eugracilis        SATGKRISPPNKRLNGRKPSSASIVMIHEPQQRQRLIPRLQNKAREKS--K--DRVEKAE        611
Erecta            SATGKRISPPNKRLNGRKPSSASIVMIHEPQQRQRLMPRLQNKAREKG--K--ERVEKTD        608
Melanogaster      SATGKRISPPNKRLNGRKPSSASIVMIHEPQQRQRLMPRLQNKAREKG--K--DRVEKTD        608
Sechellia         SATGKRISPPNKRLNGRKPSSASIVMIHEPQQRQRLMPRMQNKAREKG--K--DRVEKTD        608
Simulans          SATGKRISPPNKRLNGRKPSSASIVMIHEPQQRQRLMPRMQNKAREKG--K--DRVEKTD        548
Mauritania        SATGKRISPPNKRLNGRKPSSASIVMIHEPQQRQRLMPRMQNKAREKG--K--DRVEKTD        608
Bipectinate       PATGKRISPPNKRLNGRKASSASIVMIHEPQQRHRLLPRLHSQRKDRDKIK--DRDKEND        606
Anannassae        PATGKRISPPNKRLNGRKASGASIVMIHEPQQRNRLLPRLHSQRKGRDKAK--DRDMEND        604
Mojavensis        LSSNIRISPQSRRLNCRKASSVIIVIANEPQRQRAAQQ----RNNNN-TANGNGNGNGNG        613
Virilism          QQSGKRLSQSTKRSNSRKASSVTIVISTEPQIHR----YVPRRNNNNRASTGSARVRGI-        558
Grimshawi         ISTSRRVPPPIKRLKSRKANNVTIVISNEPQQQQQQHQMLQQRNTNNNSTNGS-------        618
                     . *:    :* : ** ... **:  **  :.         :   .

Serrata           IQLQQSQAEAMVADPTTITTANRIRSKDDDGGNGGGGGGGGGSGSKWMMG-ICFRGQKDKC        683
Kikkawei          TQHEAVS---EPINNVTTTKANRIRSKDEDSSNGRGG----GGSKWMMG-ICFRGQKDKC        682
Rhopaloa          A----------ETEPEPDPAITRIHSKE--AGS--TASRNSRGSKWIMG-ICFRGQKDKC        652
Elegans           ADA-DAE-TNTKTEPEPDPAITRIHSKESTAGS--TGSRSNRGSKWIMG-ICFRGQKDKC        665
Fichsuphila       I------------DTELDPAITRIQSKESTG----STGSRSRGSKWIMG-ICFRGQKDKC        660
Takahashi         A----------ETDPQADPAISRIHSKESSGGGG-STGSRNRGSKWIMG-ICFRGQKDKC        669
Suzuki            AA---------ETDANPDPAISRIHSKES-GIAG-STGSRTRGSKWIMG-ICFRGQKDKC        659
Biarmipes         AA---------ETDPNQDPAISRIHSKET-GIAG-GTGSRTRGSKWIMG-ICFRGQKDKC        659
Eugracilis        PE------------TEPDPAISRIHSKET-GVGG-GTGSRTRGSKWIMG-ICFRGQKDKC        656
Erecta            KE----------AEPEPDPAISRIHSKEA-D----RARSRTRGSKWIMG-ICFRGQKDKC        652
Melanogaster      AE----------A--EPDPTISHIHSKEA-G----SARSRTRGSKWIMG-ICFRGQKDKC        650
Sechellia         AE----------A--EPDPAISRIHSKEA-G----SARSRTRGSKWIMG-ICFRGQKDKC        650
Simulans          AE----------A--EPDPAIARIHSKEA-G----SARSRTRGSKWIMG-ICFRGQKDKC        590
Mauritania        AE----------A--EPDPAISRIHSKEA-G----SARSRTRGSKWIMG-ICFRGQKDKC        650
Bipectinate       RDRQR-D-DRQLEETEADPATTRIHSKETAR------TGRNRGSKWMMDIICFRGQKDKC        658
Anannassae        KDRKRSD-DRQLEETEADPGTIRIHSKDTER------ASRNRGSKWMMDIICFRGQKDKC        657
Mojavensis        SRNQG----KDEPASGSARSGQRIRSKETP-------EQGRSTGNWMFS-LCFHGQKNKC        661
Virilism          --LKA----TEEPASGSA-VGQRIRSTDDGA------STTGRNSNWMFG-LCFRGQKNKC        604
Grimshawi         --SPR----TVEDDTGSA-MATRIRSKES----------SAGRSNWMTN-LCFRGKKNKC        660
                                        :*:*.:                .:*:  . :**:*:*:**

Serrata           VMAGGQKPQQIFMTSQLPPTSTLAAIATTTITTTTGSATSATAT-------AAAKATTTI        736
Kikkawei          VMSGGQKPQQIFMTSQLPPTSTLAAVATTTITTT-GSATSATAA-------AAAK-ATAI        733
Rhopaloa          VMPGSQKTQQIFMTSQLAPTSTLAAVATTTITTTTT-----------AAAKTTIATIATI        701
Elegans           VMPGSQKTQQIFMTSQLAPTSTLAAVATTTITTTTT-----------AAAKTTIATIATI        714
Fichsuphila       VMPGSQKTQQIFMTSQLPPTSTLTPVATTTITTTTT----------AAAAKTTIATSATI        710
Takahashi         VMPGSQKTQQIFMTSQLPPTSTLAAVATTTITTTTT----------AAAAKTTIATIATI        719
Suzuki            VMPGSQKTQQIFMTSQLPPTSTLAAVATTTITTSTTT----------AAAAKTTIATIATI       709
Biarmipes         VMPGSQKTQQIFMTSQLPPTSTLAAVATTTITTTTT----------AVAAKTTIATIATI        709
Eugracilis        VMPGSQKTQQIFMTSQMAPTSTLAAVATTTITTTTTTT--------AAAAKTTIATIATI        708
Erecta            VMPGSQKTQQIFMTSQMPPTSTLVVATTMATTATTTTS-------AAAAKTTIASIATI         705
Melanogaster      VMPGSQKTQQIFMTSQMPPTSTLAAVATTTITTTSTTTT----------AAKTTIASIATI       700
Sechellia         VMPGSQKTQQIFMTSQMPPTSTLAAVATTTITTTT-----------AAPAKTTIASIATI        699
Simulans          VMPGSQKTQQIFMTSQMPPTSTLAAVATTTITTTSTTTT--------AAAAKTTIASIATI       642
Mauritania        VMPGSQKTQQIFMTSQMPPTSTLAAVATTTITTTSTTTT--------AAAAKTTIASIATI       702
Bipectinate       VMPGSQKSQQIFMTSQLPPTSKLAAITTTSLSTTTATTTTTIATVAAAAASVKTTTATTI        718
Anannassae        VMPGPQKPQQIFMTSQLPPTSKLAAITTTSLSTTTIAATTTTIATIAAAASV--KTTTTI        715
Mojavensis        VMPGA---QQIFMTSQLSATTSMATPTTTTTTTTT----------------TAIATITTT        702
Virilism          VIPNAQVSQQIFMTSQLP---------TATAATTTTTTT-----VAAAAAATTTTAATAV        656
Grimshawi         VMPGAQVSQQIFMTSQLPTSTTALATITTTTATATAATTAAAAATTTT--RAAVAAVTTT        718
                  *: .    ********:  ::       **  ::                  .     ::
```

| | | |
|---|---|---|
| Serrata | **AT**ITNSK—AKAKAKAIAKNNAK-------PKA | 760 |
| Kikkawei | ATKTKSK—AKAKAKAITKNHAK-------PKA | 757 |
| Rhopaloa | **AT**ITKS----KAKAKAIAKSHQM------PKS | 723 |
| Elegans | **AT**ITKS----KAKAKAIAKGHQM------PKS | 736 |
| Fichsuphila | **AT**ITKS----KTKAKAISKNYQI------PKA | 732 |
| Takahashi | **AT**ITKS----KAKAKAIAKSHQM------PKA | 741 |
| Suzuki | **AT**ITKS----KAKAKAIAKSHQM------PKA | 731 |
| Biarmipes | **AT**ITKS----KARAKAIASSHQI------PKA | 731 |
| Eugracilis | **AT**ITKS----KAKAKAITK---------MPKA | 727 |
| Erecta | **AT**ITKS----KAKAKAISKSH----------- | 722 |
| Melanogaster | **AT**MTKS----KAKAKAISKSHQI----QMPKA | 724 |
| Sechellia | **AT**ITKS----KAKAKAISKSHQI----QMPKA | 723 |
| Simulans | **AT**ITKS----KAKAKAISKSHQI----QMPKA | 666 |
| Mauritania | **AT**ITKS----KAKAKAISKSHQI----QMPKA | 726 |
| Bipectinate | **ST**ITKS----KAKAKAITKNHRT------PKA | 740 |
| Anannassae | **ST**ITKS----KAKAKAITKNPRT------PKA | 737 |
| Mojavensis | **S**------------------------------ | 703 |
| Virilism | AAA**TTTSRIS**SAAAAAAAAAAAILQKQQTPKA | 682 |
| Grimshawi | TALAAAAAAAAVAAAANTTKAAIA-------- | 742 |

Length of PDFR-PD CT

| | |
|---|---|
| Serrata | =760-512 |
| Kikkawei | =757-517 |
| Rhopaloa | =723-500 |
| Elegans | =736-503 |
| Fichsuphila | =732-510 |
| Takahashi | =741-514 |
| Suzuki | =731-504 |
| Biarmipes | =731-504 |
| Eugracilis | =727-504 |
| Erecta | =722-501 |
| Melanogaster | =724-501 |
| Sechellia | =723-501 |
| Simulans | =666-441 |
| Mauritania | =726-501 |
| Bipectinate | =740-496 |
| Anannassae | =737-494 |
| Mojavensis | =703-506 |
| Virilism | =682-552 |
| Grimshawi | =742-514 |

| | |
|---|---|
| Serrata | 248 |
| Kikkawei | 240 |
| Rhopaloa | 223 |
| Elegans | 233 |
| Fichsuphila | 222 |
| Takahashi | 227 |
| Suzuki | 227 |
| Biarmipes | 227 |
| Eugracilis | 223 |
| Erecta | 221 |
| Melanogaster | 223 |
| Sechellia | 222 |
| Simulans | 225 |
| Mauritania | 225 |
| Bipectinate | 244 |
| Anannassae | 243 |
| Mojavensis | 200 |
| Virilism | 220 |
| Grimshawi | 228 |

Melanogaster **NP_001284826.1**

```
1 mtllsnildc ggcisaqrft rllrqsgssg pspsaptagt fesksmlept sshslatgrv
       61 pllhdfdast tespgtyvld gvarvaqlal eptvmdalpd sdteqvlgnl nssapwnltl
      121 asaaatnfen csalfvnytl pqtglycnwt wdtllcwppt pagvlarmnc pggfhgvdtr
      181 kfairkceld grwgsrpnat evnppgwtdy gpcykpeiir lmqqmgskdf dayidiarrt
```

241 rtleivglcl slfalivsll ifctfrslrn nrtkihknlf vamvlqviir ltlyldqfrr
301 gnkeaatnts lsvientpyl ceasyvlley artamfmwmf ieglylhnmv tvavfqgsfp
361 lkffsrlgwc vpilmttvwa rctvmymdts lgeclwnynl tpyywilegp rlavillnfc
421 flvniirvlv mklrqsqasd ieqtrkavra aivllpllgi tnllhqlapl ktatnfavws
481 ygthfltsfq gffialiycf lngevravll kslatqlsvr ghpewapkra smysgaynta
541 pdtdavqpag dpsatgkris ppnkrlngrk pssasivmih epqqrqrlmp rlqnkarekg
601 kdrvektdae aepdptishi hskeagsars rtrgskwimg icfrgqkdkc vmpgsqktqq
661 ifmtsqmppt stlaavatti tttsttttaa kttiasiati atmtkskaka kaiskshqiq
721 mpka

Simulans **XP_016037874.1**

61 pllhdfdast tespgtyvld gvarvaqlal eptvmdalpd pdteqvlgnl nssapwnltl
121 asaaatnfen csalfvnytl pqtglycnwt wdtllcwppt pagvlarmnc pggfhgvdtr
181 kfairkceld grwgsrpnat evsppgwtdy gpcykpeiir lmqqmgskdf dayidiarrt
241 rtleivglcl slfalivsll ifctfrslrn nrtkihknlf vamvlqviir ltlyldqfrr
301 gnkeaatnts lsvientpyl ceasyvlley artamfmwmf ieglylhnmv tvavfqgsfp
361 lkffsrlgwc vpilmttvwa rctvmymdts lgeclwnynl tpyywilegp rlavillnfc
421 flvniirvlv mklrqsqasd ieqtrkavra aivllpllgi tnllhqlapl ktatnfavws
481 ygthfltsfq gffialiycf lngevravll kslatqlsvr ghpewapkra smysgaynta
541 pdtdavqpag dpsatgkris ppnkrlngrk pssasivmih epqqrqrlmp rmqnkarekg
601 kdrvektdae aepdpaiari hskeagsars rtrgskwimg icfrgqkdkc vmpgsqktqq
661 ifmtsqmppt stlaavatti tttsttttaa aakttiasia tiatitkska kakaiskshq
721 iqmpka

Suzuki **XP_016939141.1**

1 mtllsnildc gggisaqrft rllrqsssg spsssssgtt fesksmlept sshilptgrv
61 pilhdfdfds lttestgthv ldgvasvakm aleptvmdav lpdsdtdqvl snlnisapwn
121 itlasaaatn fencsslfvn ytlpqsglyc nwtwdtllcw pptpagvlar mncpggfhgv
181 dtrkfairkc eldgrwgsrp natevsppgw tdygpcykpe iirlmqqmgs kdfdlyidia
241 rktrtleivg lclslfaliv sllifctfrs lrnnrtkihk nlfvamvlqv iirltlyldq
301 frrgnkeaat ntslsvient pylceasyvl leyartamfm wmfieglylh nmvtvavfqg
361 sfplkffsrl gwgfpilmtt vwarctvmym dtslgeclwn ynltpyywil egprlavill
421 nfcflvniir vlvmklrqsq asdieqtrka vraaivllpl lgitnllhql aplktatnfa
481 vwsygthflt sfqgffiali ycflngevra vllkslatql svrghpewap krasmysgay
541 ntapdtdavq pagdpsatgk risspphkrln grkpssasiv mihepqqrhr liprlqnker
601 ekskdrveka daaetdanpd paisrihske sgiagstgsr trgskwimgi cfrgqkdkcv
661 mpgsqktqqi fmtsqlppts tlaavattit tstttaaaak ttiatiatia titkskakak
721 aiakshqmpk a

Mauritania **XP_033169658.1**

1 mtllsnildc ggcisaqrft rllrqsgssg pspsapapgt fesksmlept sshslatgrv
61 pllhdfdatt tespgthvld gvarvaqlal eptvmdalpd pdteqvlgnl nsnapwnltl
121 asaaatnfen csalfvnytl pqtglycnwt wdtllcwppt pagvlarmnc pggfhgvdtr
181 kfairkceld grwgsrpnat evsppgwtdy gpcykpeiir lmqqmgskdf dayidiarrt
241 rtleivglcl slfalivsll ifctfrslrn nrtkihknlf vamvlqviir ltlyldqfrr
301 gnkeaatnts lsvientpyl ceasyvlley artamfmwmf ieglylhnmv tvavfqgsfp
361 lkffsrlgwc vpilmttvwa rctvmymdts lgeclwnynl tpyywilegp rlavillnfc
421 flvniirvlv mklrqsqasd ieqtrkavra aivllpllgi tnllhqlapl ktatnfavws
481 ygthfltsfq gffialiycf lngevravll kslatqlsvr ghpewapkra smysgaynta
541 pdtdavqpag dpsatgkris ppnkrlngrk pssasivmih epqqrqrlmp rmqnkarekg
601 kdrvektdae aepdpaisri hskeagsars rtrgskwimg icfrgqkdkc vmpgsqktqq
661 ifmtsqmppt stlaavatti tttsttttaa aakttiasia tiatitkska kakaiskshq
721 iqmpka

Sechellia **XP_032581353.1**

1 mtllsnildc ggcisdqrft hllrqsgssg pspsapapgt fesksmlept sphslatgrv
61 pllhdfdast tespgtyvld gvarvaqlal eptvmdalpd pdteqvlgnl nssapwnltl
121 asaaatnfen csalfvnytl pqtglycnwt wdtllcwppt pagvlarmnc pggfhgvdtr
181 kfanrkceld grwgsrpnat evsppgwtdy gpcykpeiir lmqqmgskdf dayidiarrt
241 rtleivglcl slfalivsll ifctfrslrn nrtkihknlf vamvlqviir ltlyldqfrr
301 gnkeaatnts lsvientpyl ceasyvlley artamfmwmf ieglylhnmv tvavfqgsfp
361 lkffsrlgwc vpilmttvwa rctvmymdts lgeclwnynl tpyywilegp rlavillnfc
421 flvniirvlv mklrqsqasd ieqtrkavra aivllpllgi tnllhqlapl ktatnfavws
481 ygthfltsfq gffialiycf lngevravll kslatqlsvr ghpewapkra smysgaynta
541 pdtdavqpvg dpsatgkris ppnkrlngrk pssasivmih epqqrqrlmp rmqnkarekg

601 kdrvektdae aepdpaisri hskeagsars rtrgskwimg icfrgqkdkc vmpgsqktqq
661 ifmtsqmppt stlaavatti tttttaapak ttiasiatia titkskakak aiskshqiqm
721 pka

Serrata **XP_020802784.1**
1 mtllssilds gggisaqrft rllrqsstss sssssssasa sgsmleptss qlpindvlgg
61 gripflhdng tgeslplpda daldpnfvld gvtsvakmal eatvkevlrd pdpeqilana
121 natapwnitl asaaatnyen csalfanytl pqtglycnwt wdtllcwppt pagvlarmnc
181 pggyhgvdtr kfanrkceld grwgsrpnat etnatgwtdy gpcykpevir lmqqmgsred
241 ldlyieiarr trtleivglc lslfalivsl lifctfrslr nnrtkihknl fvamvlqvii
301 rltlyldqfr rgskeaatnt slsvientpy lceasyvlle yartamfmwm fieglylhnm
361 vtvavfqgsf plkffsrlgw cvpilmttvw arctviymdt slgdclwnyn ltpyywileg
421 prlavillnf cflvniirvl vmklrqsqas dieqtrkavr aaivllpllg itnilhqvap
481 lktatnfavw systyfltsf qgffialiyc flngevravl lkslatqlsv rghpewapkr
541 asmysgaynt apdtdavqqh qqtgenpatg krisppnkrl ngrkassasi vlihepqqrq
601 rliprlqska rekdksldkd rgkriqlqqs qaeamvadpt tittanrirs kdddggnggg
661 gggggsgskw mmgicfrgqk dkcvmaggqk pqqifmtsql pptstlaaia tttittttgs
721 atsatataaa katttiatit nskakakaka iaknnakpka

Erecta **XP_026838096.1**
1 mtllsnildc ggcisaqrft rllrqsgssv pspsapapgt fesismlept slhslptgrv
61 pllhdfdast tespgtyvld gvarvaqlal eptvmdglpd pdteqvlsnl nssapwnltl
121 ssaaatnfen csalfvnytl pqtglycnwt wdtllcwppt pagvlarmnc pggfhgvdtr
181 kfairkceld grwgsrpnat evsppgwtdy gpcykpeiir lmqqmgskdf dayidiarrt
241 rtleivglci slfalivsll ifctfrslrn nrtkihknlf vamvlqviir ltlyldqfrr
301 gnkeaatnts lsaientpyl ceasyvlley artamfmwmf ieglylhnmv tvavfqgsfp
361 lkffsrlgwc vpilmttvwa rctvmymdts lgeclwnynl tpyywilegp rlavillnfc
421 flvniirvlv mklrqsqasd ieqtrkavra aivllpllgi tnilhqlapl ktatnfavws
481 ygthfltsfq gffialiycf lngevravll kslatqlsvr ghpewapkra smysgaynta
541 pdtdavqpag dpsatgkris ppnkrlngrk pssasivmih epqqrqrlmp rlqnkarekg
601 kervektdke aepepdpais rihskeadra rsrtrgskwi mgicfrgqkd kcvmpgsqkt
661 qqifmtsqmp ptstlavvat tmattatttt saaaakttia siatiatitk skakakaisk
721 sh

Takahashi **XP_016994844.2**
1 mtllsnildc gggisaqrfa rllrqsssg spsasasssa ssssgtsfes ksmleptssq
61 nlptgrvpil hdfdfdfdpt tttespgnyv ldgvasvakm aleptvmdtv lpdsdpdqvl
121 snlnvsapwn ltlasaaatn fencsalfvn ytlpqtglyc nwtwdtllcw pptpagvlar
181 mncpagfhgv dtrkfairkc eldgrwgsrp natevsppgw tdygpcykpe iirlmeqmgs
241 knfdlyidia wktrtleivg lclslfaliv sllifctfrs lrnnrtkihk nlfvamvlqv
301 iirltlyldq yrrgnkeaat ntslsvient pylceasyvl leyartamfm wmfieglylh
361 nmvtvavfqg sfplkffsrl gwgfpilmtt vwarctvmym dtslgeclwn ynltpyywil
421 egprlavill nfcflvniir vlvmklrqsq asdieqtrka vraaivllpl lgitnilhqm
481 aplktatnfa vwsyvthflt sfqgffiali ycflngevra vllkslatql svrghpewap
541 krasmysgay ntapdtdavq pagdpsatgk rispphkrln grkpssasiv mihepqqrqr
601 liprlqnkar eksrdrvdka daetdpqadp aisrihskes sggggstgsr nrgskwimgi
661 cfrgqkdkcv mpgsqktqqi fmtsqlppts tlaavattit tttttaaaak ttiatiatia
721 titkskakak aiakshqmpk a

Biarmipes **XP_016948714.1**
1 mtllsnildc gggisaqrft rllrqssspg flsssslgtt fesksmlept sshvlptgrv
61 pvlhdfdfds sttelprilv ldgvagvatm aleptamdav lpdsdpdqvl snfnisapwn
121 itlasaaatn fencsslfvn ytlpqtglyc nwtwdtllcw pptpagvlar mncpggfhgv
181 dtrkfairkc eldgrwgsrp natevsppgw tdygpcykpe iirlmqqmgs kdfdlyidia
241 rktrtleivg lclslfaliv sllifctfrs lrnnrtkihk nlfvamvlqv iirltlyldq
301 frrgnkeaat ntslsvient pylceasyvl leyartamfm wmfieglylh nmvtvavfqg
361 sfplkffsrl gwgfpilmtt vwarctvmym dtslgeclwn ynltpyywil egprlavill
421 nfcflvniir vlvmklrqsq asdieqtrka vraaivllpl lgitnllhql aplktatnfa
481 vwsygthflt sfqgffiali ycflngevra vllkslatql svrghpewap krasmysgay
541 ntapdtdavq pagdplatgk rispphkrln grkpssasiv mihepqqrhr liprlqnkar
601 eksrdrvdka daetdpnqd paisrihske tgiaggtgsr trgskwimgi cfrgqkdkcv
661 mpgsqktqqi fmtsqlppts tlaavattit tttttavaak ttiatiatia titkskarak
721 aiasshqipk a

Eugracilis **XP_017067614.1**

```
    1 mtllsnilds gggisaqrft rllrqssssg sssasasass ygtlesksml eptsshtlpg
   61 grvpilhdfd ssttespgay lldgvasvak malepavmdv lpdpdtdqvl snlnitapwn
  121 ltlasaaatn fencsalfvn ytlpqtglyc nwtwdtllcw pptpagvlar mncpggfhgv
  181 dtrkfairkc eldgrwgsrp nateasppgw tdygpcykpe iirlmqqmgn kdfdlyidia
  241 rktrtleivg lclslfaliv sllifctfrs lrnnrtkihk nlfvamvlqv iirltlyldq
  301 frrgnkeaat ntslsvient pylceasyvl leyartamfm wmfieglylh nmvtvavfqg
  361 sfplkffsrl gwcvpilmtt vwarctvmym dttlgeclwn ynltpyywil egprlavill
  421 nfcflvniir vlvmklrqsq asdieqtrka vraaivllpl lgitnllhql aplktatnfa
  481 vwsygthflt sfqgffiali ycflngevra vllkslatql svrghpewap krasmysgay
  541 ntapdtdavq pagdpsatgk risppnkrln grkpssasiv mihepqqrqr liprlqnkar
  601 ekskdrveka epetepdpai srihsketgv gggtgsrtrg skwimgicfr gqkdkcvmpg
  661 sqktqqifmt sqmaptstla avattitttt ttttaaaakt tiatiatiat itkskakaka
  721 itkmpka
```

Rhopaloa **XP_016982481.1**

```
    1 mtllsnildc gggisaqclt rslressspg spgspesgaf esksmlepts shilptgrvp
   61 vlhdfnasst dspgtflldg vvsvaqmale ptltdllpdp dpdqvlsnln asapwnltla
  121 saaatnfenc salfanytlp qtglycnwtw dtllcwpptp agvlarmncp agfhgvdtrk
  181 fairkceldg rwgsrpnate vsppgwtdyg pcykpeiirl mqqmgsrdfd tfidiakktr
  241 tleivglcls lfalivslli fctfrslrnn rtkihknlfv amvlqviirl tlyldqyrrg
  301 nkeaatntsl svientpylc easyvlleya rtamfmwmfi eglylhnmvt vavfqgsfpl
  361 kffsrlgwcv pilmttvwar ctvmymdtsl gdclwnynlt pyywilegpr lavillnfcf
  421 lvniirvlvm klrqsqasdi eqtrkavraa ivllpllgit nilhqmaplk tatnfavwsy
  481 gthfltsfqg ffialiycfl ngevravllk slatqlsvrg hpewapkras mysgayntap
  541 dtdavqpagd platgkrisp pnkrlngrkp ssasivmihe pqqrqrlipr lqnkarekgr
  601 drvekadaet epepdpaitr ihskeagsta srnsrgskwi mgicfrgqkd kcvmpgsqkt
  661 qqifmtsqla ptstlaavat titttttaa akttiatiat iatitkskak akaiakshqm
  721 pks
```

Fichsuphila **XP_017048315.1**

```
    1 mtllasildc gggisaqrft rllaqsslla sssasasasa sasasgtfas esmleptssh
   61 ilptgrvpvl hgfdsttes ttstyvldgv asvaqmplle stvmdalpds dpdqafgnln
  121 vtapwnltla saaatnfenc salfanytlp qsglycnwtw dtllcwpptp agvlarmncp
  181 ggfhgvdtrk fairkceldg rwgsrpnate vsppgwtdyg pcykpeiirl mqqmgnkdfd
  241 lyidiarktr tleivglcls lfalivslli fctfrslrnn rtkihknlfv amvlqviirl
  301 tlyldqfrrg nkeaatntsl svientpylc easyvlleya rtamfmwmfi eglylhnmvt
  361 vavfqgsfpl kffsrlgwcv pilmttvwar ctvmymdtsl geclwnynlt pyywilegpr
  421 lavillnfcf lvniirvlvm klrqsqasdi eqtrkavraa ivllpllgit nllhqlaplk
  481 tatnfavwsy gthfltsfqg ffialiycfl ngevravllk slatqlsvrg hpewapkras
  541 mysgayntap dtdavhpagd psapgkrisp pnkrlngrkp ssasivmihe pqqrhrlipr
  601 lqnknqekgk drvekadidt eldpaitriq skestgstgs rsrgskwimg icfrgqkdkc
  661 vmpgsqktqq ifmtsqlppt stltpvatti tttttaaaa kttiatsati atitksktka
  721 kaisknyqip ka
```

Elegans **XP_017114250.1**

```
    1 mtllsnildc gggipaqrla rllrqssssg stpsgsasgp fesksmlept tshilptgrv
   61 pilhdfgsss tespltgtyl ldgvasvaem aleptvrdil sdpdpdkvls nlnasapwnl
  121 tlassaatnf encsalfany tlpqtglycn wtwdtllcwp ptpagvlarm ncpggfhgvd
  181 trkfairkce ldgrwgsrpn atevsppgwt dygpcykpei irlmqqmgsk dfdlyidiar
  241 ktrtleivgl clslfalivs llifctfrsl rnnrtkihkn lfvamvlqvi irltlyldqy
  301 rrgnkeaatn tslsvientp ylceasyvll eyartamfmw mfieglylhn mvtvavfqgs
  361 fplkffsrlg wcapilmttv warctvmymd tslgeclwny nltpyywile gprlavilln
  421 fcflvniirv lvmklrqsqa sdieqtrkav raaivllpll gitnllhqla plktatnfav
  481 wsygthflts fqgffialiy cflngevrav llkslatqls vrghpewapk rasmysgayn
  541 tapdtdavqp agdpsatgkr ispphkrlng rkpssasivm iheppqrqrl iprlqskare
  601 kgrdraerad adadaetntk tepepdpait rihskestag stgsrsnrgs kwimgicfrg
  661 qkdkcvmpgs qktqqifmts qlaptstlaa vattittttt taaakttiat iatiatitks
  721 kakakaiakg hqmpks
```

Kikkawei **XP_017037981.1**

```
    1 mtllssilds ggsisaqrft rllrqsstss sssvsasasm leptssqlpn ndvlgggrip
   61 flpgnatges sssspplps pslpdadald pnfvldgvts vakmaleatv kvlrdpdpeq
  121 ilananasap wnitlaaaaa tnyencsalf anytlpqtgl ycnwtwdtll cwpptpagvl
  181 armncpggyh gvdtrkfanr kceldgrwgs rpnatetnat gwtdygpcyk pevirlmqqm
  241 gsredldlyi eiakrtrtle ivglclslfa livsllifct frslrnnrtk ihknlfiamv
```

```
301 lqviirltly ldqfrrgnke aatntslsvi entpylceas yvlleyarta mfmwmfiegl
361 ylhnmvtvav fqgsfplkff srlgwcvpil mttvwarctv iymdtslgdc lwnynltpyy
421 wilegprlav illnfcflvn iirvlvmklr qsqasdieqt rkavraaivl lpllgitnil
481 hqlaplktat nfavwsysty fltsfqgffi aliycflnge vravllksla tqlsvrghpe
541 wapkrasmys gayntapdtd avlqqqqqpg enpatgkris ppnkrlngrk assasivmih
601 epqqrqrlip rlrsrtrekd ksldkdrekr tqheavsepi nnvtttkanr irskdedssn
661 grggggskwm mgicfrgqkd kcvmsggqkp qqifmtsqlp ptstlaavat ttitttgsat
721 sataaaaaka taiatktksk akakakaitk nhakpka
```

Bipectinate XP_017095129.2

```
  1 mtllssilds ggsvsvqrlt rllrqsspsa sasgtalase smveptasqm lneitdgrrv
 61 pslgqdlatt tettgqsnvi idrvtnvakm aleatvmddq vlagvnasah wnmtlsssta
121 tnyencsalf anytlpqtgl ycnwtwdtll cwpptpagvl armhcpggyh gvdtrkfanr
181 kceldgrwgs rpnatepspa gwtdygpcyk pevirlmqsm gskdidiyid iarrtrtlei
241 vglwvslfal visllifctf rslrnnrtki hknlfiamvl qvivrltlyl dqyrrgnkea
301 atntslsaie ntpylceasy vlleyartam fmwmfiegly lhnmvtvavf qgsfplkffs
361 rlgwcvpilm tfvwarctvm ymdtsmgecl wnynltpyyw ilegprlavi llnfcflvni
421 irvlvvklrq sqasdieqtr kavraaivll pllgitnllh qlaplktatn favwsygthf
481 ltsfqgffia liycflngev ravllkslat qmsvrghpew vpkrasmysg ayntapdtda
541 viqqpgdnpa tgkrisppnk rlngrkassa sivmihepqq rhrllprlhs qrkdrdkikd
601 rdkendrdrq rddrqleete adpattrihs ketartgrnr gskwmmdiic frgqkdkcvm
661 pgsqksqqif mtsqlpptsk laaitttsls tttattttti atvaaaaasv ktttattist
721 itkskakaka itknhrtpka
```

Anannassae XP_014759839.1

```
  1 mtllssilds ggsvsvqrlt rllrqsstss sasgpslasd sissmveats lrvpslghdl
 61 sttteтnvii drvtnvakma leatvmdvlp dpepdqvltg vnasshwnmn mtlssasatn
121 yencsalfan ytlpqtglyc nwtwdtllcw pptpagvlar mncpggyhgv dtrkfanrkc
181 eldgrwgsrp natepspagw tdygpcykpe virlmqsmgs kdidiyidia rrtrtleivg
241 lwvslfalvi sllifctfrs lrnnrtkihk nlfvamvlqv ivrltlyldq yrrgnkeaat
301 ntslsaient pylceasyvl leyartamfm wmfieglylh nmvtvavfqg sfplkffsrl
361 gwcvpilmtf vwarctvmym dtsmgeclwn ynltpyywil egprlavill nfcflvniir
421 vlvvklrqsq asdieqtrka vraaivllpl lgitnllhql aplktatnfa vwsygthflt
481 sfqgffiali ycflngevra vllkslatqm svrghpewvp krasmysgay ntapdtdavi
541 qqpgenpatg krisppnkrl ngrkasgasi vmihepqqrn rllprlhsqr kgrdkakdrd
601 mendkdrkrs ddrqleetea dpgtirihsk dterasrnrg skwmmdiicf rgqkdkcvmp
661 gpqkpqqifm tsqlpptskl aaitttslst ttiaatttti atiaaaasvk ttttistitk
721 skakakaitk nprtpka
```

Mojavensis XP_015016735.1

```
1 mqlagdseag smptiittst ssnsflqria lkavtaattt kdrlisivet dspagshmdp
 61 stamtttsit atatatpaat ataselsrsd vlnasieatt avgmdtvkta tesglptvsg
121 ttlpswnssf nsalannydn csamfanyth pqtglycnwt wdsllcwppt pagnlarmnc
181 pagyhgvdtr kfanrkceld ghwggrpnet epnhagwtdy gpcykpevir lmqeikdvnl
241 yidiaqrtrt leiiglclsl laliislvif cafrslrnnr tkihknlfia mvlqvivrlt
301 lyldqfrrgi qynsslsaie ntpylceasy vlleyartam fmwmfiegly lhnmvtvavf
361 qgsfpliffs llgwgmpvvm tfvwvqctai fmdtalgdcm wnynltpyyw ilegprlavi
421 llnffflvni irvlvvklrq sqasdieqtr kavraaivll pllgitnllh lvpalktawk
481 faiwsyvthf ltsfqgffia liycflngev ravllkslav wmsvrghpew vpkrasmysa
541 ayntapdtep paqqsveals snirispqsr rlncrkassv iivianepqr qraaqqrnnn
601 ntangngngn gngsrnqgkd epasgsarsg qrirsketpe qgrstgnwmf slcfhgqknk
661 cvmpgaqqif mtsqlsatts matptttttt ttttaiatit tts
```

```
     R   N   H   N   N   N   K   L   N   S   R   S   S   S   S   R   E   N   I   A       F1
   A   T   I   T   T   T   S   *   T   A   A   A   A   A   V   V   K   I   L   Q       F2
     Q   P   *   Q   Q   Q   A   E   Q   P   Q   Q   Q   Q   S   *   K   Y   C   K     F3
  1 CGCAACCATAACAACAACAAGCTGAACAGCCGCAGCAGCAGCAGTCGTGAAAATATTGCA 60
    ----:----|----:----|----:----|----:----|----:----|----:----|

    K   A   K   I   L   T   V   N   L   K   N   C   *   T   P   K   A   *   R   E       F1
     K   P   K   Y   *   L   *   T   L   R   T   V   K   R   L   K   L   N   A   S     F2
      S   Q   N   T   N   C   K   P   *   E   L   L   N   A   *   S   L   T   R   A    F3
 61 AAAGCCAAAATACTAACTGTAAACCTTAAGAACTGTTAAACGCCTAAAGCTTAACGCGAG 120
```

Virilism XP_032288761.1

```
1 mptaapssnr tvsthhsttm tststtygtt sttqaaltsf epmavtaagm dvtmagtepl
      61 sstsiptfwn ssintasans ydncsalfan ytqpttviyc nwtwdsllcw pptpagatah
     121 mhcpagyhgv dtrkfanrkc eldghwagrp nsteqkptgw tdygpcykpe virlmqeikd
     181 vnlymdiaqr trtleiiglc lslfaliisl mifcafrslr nnrtkihknl fvamvlqviv
     241 rltlyldqfr rgksdsannt slsvientpy lceasyvlle yartamfmwm fieglylhnm
     301 itvavfqgnf plvffsllgw gmpvlmtfvw vqctaifmdt slgdclwnyn ltpyywileg
     361 prltvimlnf fflvniirvl vmklrqsqas eieqtrkavr aaivllpllg itnllhlvpa
     421 lktawkfaiw syvthfltsf qgffialiyc flngevravm lksiavwlsv rghpewapkr
     481 psmysgaynt apdtdpqlkq gdpqqsgkrl sqstkrsnsr kassvtivis tepqihryvp
     541 rrnnnnrast gsarvrgilk ateepasgsa vgqrirstdd gasttgrnsn wmfglcfrgq
     601 knkcvipnaq vsqqifmtsq lptataattt ttttvaaaaa attttaatav aaatttsris
     661 saaaaaaaaa aailqkqqtp ka
```

Grimshawi **XP_001991602.2**

```
  1 mmqlpginkp gciptistss iiipqrfarr aarvaststa tvttlsmsmt qsgrsmlnrt
     61 mtsiatattt ststsisths ttssstsklp sqvisgladv ingteldmta aatavgldaa
    121 vpdpsttsss wnstfstasa nsydncaamf anytqpvtgl ycnwtwdsll cwpptpagvm
    181 armycpagyh gvdtrkfair kceldghwgr rpndtelntg gwtdyapcyk pevirlmqei
    241 kdvnvymdia qrtrtleiig lclsllalii slvifcafrs lrnnrtkihk nlfvamvlqv
    301 ivrltlyldq frrgrpetat nasvsiient pylceasyvl leyartamfm wmfieglylh
    361 nmvtvavfqg nfplklfall gwglpvlmtf vwvqctaifm dttvgecmwn ynltpyywil
    421 egprlavill nffflvniir vlvvklrqsq asdieqtrka vraaivllpl lgitnllhlv
    481 palktawkfa vwsyvthflt sfqgffiali ycflngevrt vllkslavwm svrghpewap
    541 krasmysgay ntapdtdvvq qpgeaistsr rvpppikrlk srkannvtiv isnepqqqqq
    601 qhqmlqqrnt nnnstngssp rtveddtgsa matrirskes sagrsnwmtn lcfrgkknkc
    661 vmpgaqvsqq ifmtsqlpts ttalatittt tatataatta aaaattttra avaavtttta
    721 laaaaaaaav aaaanttkaa ia
```
