# Supplementary material for: The incidence of candidate binding sites for β-arrestin in Drosophila neuropeptide GPCRs
Source: PLoS One. 2022 Nov 1;17(11):e0275410. doi: 10.1371/journal.pone.0275410 (PMC9624432; doi:10.1371/journal.pone.0275410)
Supplement: S20 Text — (PDF) [file pone.0275410.s024.pdf]

S20. Text **Multi-species analysis of AstA-R2 PA isoforms**
Supporting Figure 23

CLUSTAL Line-ups; Genbank Reference IDs below
Predicted TM domains in YELLOW
BBS sequences in RED

```
Grimshawi         MNQSYIMENNTPSMSNSSWLELKLTT---NNSTGNSSLSLDA--EEEEAIRQTVRWLVPL      55
Virilis           MNLS---NITLTLPSNSSWLESQLELTTASSNLDNSTLSSFY--AEAEAIRATVRWVVPF      55
Mojavensis        MELS---NLTTTLATQLHQ----LATSSLNHSIGPDNSTALQ--SEEEAIRATVRWLVPI      51
Bipectinate       ------MDNTTLATPDP----------LATMATYDDNLTSFFSEEERQAIEGTLIWLVPF      44
Anannassae        ------MDNTTLATSEL----------LATMATYDDNFTSFFSEEERQAIEGTLIWLVPF      44
Serrata           ------MENTTLIAGET-----LANISLANITQSDENITSFFTEEEWLALTGILPWLVGF      49
Kikkawei          ------MENTTLITGET-----LANISLANTTRSDENITSFFTEEEWLALTGILPWLVGF      49
Eugracilis        ------MENM--DNSTM-----VVDLV-VNSTRNDENMTSFFTDEEWVAIRGTLPWIVIF      46
Ficusphila        ------MENATFFPAKM-----LGSLA-SNATRNDENLTTFFTDEEWLAINSTLPWVVGF      48
Takahashi         ------MENA------T-----IMLNK-INSSRMEENSTSYFTDEEWLSISSTLPWIVCF      42
Rhopaloa          ------MDNITVSPAEM-----LGNIS-INASRNDENLTSFFTDEEWLAINGTLPWIVGF      48
Elegans           ------MDNITESAAEM-----LANIS-LNASRNDENLTSFFTDEEWLAISGTLPWIVGF      48
Erecta            -----------MENTAL-----LANIS-LNATRNEENITSFFTDEEWLAISGTLPWIVGF      43
Mauritania        -----------MENTTM-----LANIS-LNATRNEENITSFFTDEEWLAINGTLPWIVGF      43
Sechelia          -----------MENTTM-----LANIS-LNATRNEENITSFFTDEEWLAINGTLPWIVGF      43
Melanogaster      -----------MENTTM-----LANIS-LNATRNEENITSFFTDEEWLAINGTLPWIVGF      43
simulans          -----------MENTTM-----LANIS-LNATRNEENITSFFTDEEWLAINGTLPWIVGF      43
Suzuki            --------MDTMENSTL-----LGKIS-LNASRSDENITSFFTDEEWLAISGTLPWIVVF      46
biarmipes         --------MDTMDNATL-----LGKIS-LNASRNDENITSFFTDEEWLAINGTLPWIVAF      46
                                               .     .. :      *  ::   : *:* :

Grimshawi         FFGIIAISGFFFGNLLVILVVLLNKNMHSTTNLLIVNLAAADLLFVIFCVPFTAVDYVAD      115
Virilis           FFGIIAISGF-FGNLLVILVVLLNKNMHSTTNLLIVNLAAADLLFVIFCVPFTAIDYVTQ      114
Mojavensis        FFGIIAVSGF-FGNLLVILVVLLNKNMHSTTNLLIVNLAAADLLFVIFCVPFTAVDYVTQ      110
Bipectinate       FFGIIALTGF-FGNLLVILVVVFNKNMRSTTNLMIVNLAVADLLFVIFCIPFTATDYVTK      103
Anannassae        FFGIIALTGF-FGNLLVILVVVFNKNMRSTTNLMIVNLAVADLLFVIFCIPFTATDYVTK      103
Serrata           TFGAIAITGF-LGNLLVILVVVFNNNMRSTTNLMIVNLAAADLLFVILCIPFTAADYVTD      108
Kikkawei          TFGAIAITGF-LGNLLVILVVVFNNNMRSTTNLMIVNLAVADLLFVILCIPFTAADYVTD      108
Eugracilis        LFGVIAITGL-LGNLLVILVVVFNKNMRSTTNLMIVNLAAADLLFVILCIPFTATDYMVY      105
Ficusphila        FFGAIAITGF-FGNLLVILVVVFNKSMRSTTNLMIVNLAVADLLFVILCIPFTATDYMVP      107
Takahashi         FFGVIAITGF-FGNLLVILVVVFNKNMRSTTNLMIVNLAAADLMFVILCIPFTATDYMVY      101
Rhopaloa          FFGAIAITGF-FGNLLVILVVVFNKNMRSTTNLMIVNLAAADLMFVILCIPFTATDYMVY      107
Elegans           FFGAIAITGF-FGNLLVILVVVFNKNMRSTTNLMIVNLAAADLMFVILCIPFTATDYMVY      107
Erecta            FFGVIAITGF-FGNLLVILVVVFNNNMRSTTNLMIVNLAAADLMFVILCIPFTATDYMVY      102
Mauritania        FFGVIAITGF-FGNLLVILVVVFNNNMRSTTNLMIVNLAAADLMFVILCIPFTATDYMVY      102
Sechelia          FFGVIAITGF-FGNLLVILVVVFNNNMRSTTNLMIVNLAAADLMFVILCIPFTATDYMVY      102
Melanogaster      FFGVIAITGF-FGNLLVILVVVFNNNMRSTTNLMIVNLAAADLMFVILCIPFTATDYMVY      102
simulans          FFGVIAITGF-FGNLLVILVVVFNNNMRSTTNLMIVNLAAADLMFVILCIPFTATDYMVY      102
Suzuki            FFGAIAITGF-FGNLLVILVVVFNKNMRSTTNLMIVNLAAADLMFVILCIPFTATDYMVY      105
biarmipes         FFGAIAITGF-FGNLLVILVVVFNKNMRSTTNLMIVNLAAADLMFVVLCIPFTATDYMVY      105
                   ** **::*: :*********::*:.*:*****:*****.***:**::*:**** **:.

Grimshawi         HWPFGTIWCRIVQYLIVVTAYASIYTLVLMSIDRFLAVVHPIRSRMLRTEHITKIAIFTL      175
Virilis           HWPFGKMWCRSVQYLIVVTAYASIYTLVLMSIDRFLAVVHPIRSRMLRTEHITKIAIFTL      174
Mojavensis        HWPFGKIWCRSVQYLIVVTAYASIYTLVLMSIDRFLAVVHPIRSRMLRTEHITKIAIFTL      170
Bipectinate       VWPFGLFWCRSVQYLIVVTAFASIYTLVLMSIDRFLAVVHPIRSRMMRTENITMIAIVTL      163
Anannassae        VWPFGLFWCRSVQYLIVVTAFASIYTLVLMSIDRFLAVVHPIRSRMMRTENITMIAIVTL      163
Serrata           YWPFGGIWCRCVQYLIVVTAFASIYTLVLMSIDRFLAVVHPIRSRMMRTENITMVAIVTL      168
Kikkawei          YWPFGGFWCRSVQYLIVVTAFASIYTLVLMSIDRFLAVVHPIRSRMMRTENITMVAIVTL      168
Eugracilis        YWPYGRFWCRSVQYLIVVTAFASIYTLVLMSIDRFLAVVHPIRSRMMRTENITLIAIVTL      165
Ficusphila        YWPYGRFWCHSVQYLIVVTAFASIYTLVLMSIDRFLAVVHPIRSRMMRTENITLIAIVTL      167
Takahashi         YWPYGVFWCRSVQYLIVVTAFASIYTLVLMSIDRFLAVVHPIRSRMMRTENITLIAIVTL      161
Rhopaloa          YWPFGRFWCRSVQYLIVVTAFASIYTLVLMSIDRFLAVVHPIRSRMLRTENITLIAIVTL      167
Elegans           YWPFGRFWCRSVQYLIVVTAFASIYTLVLMSIDRFLAVVHPIRSRMMRTENITLIAIVTL      167
Erecta            YWPYGRFWCRSVQYLIVVTAFASIYTLVLMSIDRFLAVVHPIRSRMMRTENITLIAIVTL      162
Mauritania        YWPYGRFWCRSVQYLIVVTAFASIYTLVLMSIDRFLAVVHPIRSRMMRTENITLIAIVTL      162
Sechelia          YWPYGRFWCRSVQYLIVVTAFASIYTLVLMSIDRFLAVVHPIRSRMMRTENITLIAIVTL      162
Melanogaster      YWPYGRFWCRSVQYLIVVTAFASIYTLVLMSIDRFLAVVHPIRSRMMRTENITLIAIVTL      162
simulans          YWPYGRFWCRSVQYLIVVTAFASIYTLVLMSIDRFLAVVHPIRSRMMRTENITLIAIVTL      162
Suzuki            YWPYGRFWCRSVQYLIVVTAFASIYTLVLMSIDRFLAVVHPIRSRMMRTENITLIAIVTL      165
biarmipes         YWPYGRFWCRSVQYLIVVTAFASIYTLVLMSIDRFLAVVHPIRSRMMRTENITLIAIVTL      165
                   **:* :**: *********:*************************:***:** :**.**
```

```
Grimshawi         WTVVLTVSMPVTFTHDLVVHHNYKTNSTYAKCHFIE-NDLLDWLTFQVSFFISSYLLPLM       234
Virilis           WTVVLTVSMPVTFAHDVVVDYDNQTNVTYAMCRYID-NDVLDLSTFQVSFFISSYLLPLM       233
Mojavensis        WTVVLTVSMPVTFAHDVVVDYDNQTNVTYAMCRFID-NEVLEQSTFQVSFFISSYLLPLM       229
Bipectinate       WAVILVVSTPVGFVNDMVVLYDNKTNITYVACTYDAGNELLSPRSFQISFFISSYLLPLM       223
Anannassae        WAVILVVSTPVGFVNDMVVSYDNKTNITYVLCTFDAGNELLSPRSFQISFFVSSYLLPLM       223
Serrata           WVVILVVSVPVAFINDFAVMSDGKR--SLGMCTFKP-NDYISPRTFQVSFFISSYLLPLM       225
Kikkawei          WVVILVVSVPVAFINDFAVMSDGKR--SIGMCTFKA-NDYISPRTFQVSFFISSYLLPLM       225
Eugracilis        WIVVLIISVPVALTHDVVVDHDVKKNITYGMCTFKP-NDYVTEKSYHVIFFITSYLLPLM       224
Ficusphila        WIVVLVVSVPVALNHDVVEAIDEKKNITYGLCTYSP-NEFLSSRTYHVTFFVSSYLMPLM       226
Takahashi         WIVVLVVSLPVFFAHDVMVQYDAKKNITYGMCQFTE-NDFMGPKTYQVTFFTSSYLLPLM       220
Rhopaloa          WIVVLVVSMPVAFTHDVVVDYDAKKNITYGMCMFTN-NDFLGSRTYQVTFFISSYMLPLM       226
Elegans           WIVVLVVSVPVAFTHDVVVESDAKKNITYGMCMFIT-NDIMDSRTYHVTFFISSYMLPLM       226
Erecta            WIVVLVVSVPVAFTHDVVVDYDAKKNITYGMCTFTS-NEFLSPRTYQVTFFISSYLLPLM       221
Mauritania        WIVVLVVSVPVAFTHDVVVDYDAKKNITYGMCTFTT-NDFLSPRTYQVTFFISSYLLPLM       221
Sechelia          WIVVLVVSVPVAFTHDVVVDYDAKKNITYGMCTFTT-NDFLSPRTYQVTFFISSYLLPLM       221
Melanogaster      WIVVLVVSVPVAFTHDVVVDYDAKKNITYGMCTFTT-NDFLGPRTYQVTFFISSYLLPLM       221
simulans          WIVVLVVSVPVAFTHDVVVDYDAKKNITYGMCTFTT-NDFLSPRTYQVTFFISSYLLPLM       221
Suzuki            WIVVLVISMPVFFTHDVMVDTDAKKNITYGMCTFAT-NDFLGPKTYQVTFFISSYLLPLM       224
biarmipes         WIVVLVVSMPVFFTHDVMEDTDAKKNITYGMCTFAT-NDFLGPKTYQVTFFVSSYLLPLM       224
                  * *:* :* ** : :*.    : :   :   * :   *: :   :::: ** :**::***

Grimshawi         VISGLYVRMIMRLWRQGSGVRMSKESQRGRKRVTRLVVVVVVIAFASLWLPVQL-------       287
Virilis           VISGLYVRMIMRLWHQGTGVRMSKESQRGRKRVTRLVVVVVVIAFASLWLPVQL-------       286
Mojavensis        VISGLYVRMIMRLWHQGTGVRMSKESQRGRKRVTRLVVVVVVIAFASLWLPVQL-------       282
Bipectinate       IISGLYVRMIMRLWHQGSGVRMSKESQRGRKRVTRLVVVVVVIAFASLWLPVQL-------       276
Anannassae        IISGLYVRMIMRLWHQGSGVRMSKESQRGRKRVTRLVVVVVVIAFASLWLPVQL-------       276
Serrata           IISGLYVRMIMRLWHQGSGVRMSKESQRGRKRVTRLVVVVVVIAFASLWLPIQL-------       278
Kikkawei          IISGLYVRMIMRLWHQGSGVRMSKESQRGRKRVTRLVVVVVVIAFASLWLPIQVSILFVHS       285
Eugracilis        IISGLYMRMIMRLWRQGTGVRMSKESQRGRKRVTRLVVVVVVIAFASLWLPIQL-------       277
Ficusphila        VISGLYMRMIMRLWRQGTGVRMSKESQRGRKRVTRLVVVVVVIAFASLWLPVQL-------       279
Takahashi         IISGLYMRMIMRLWRQGTGVRMSKESQRGRKRVTRLVVVVVVIAFASLWLPVQL-------       273
Rhopaloa          IISGLYMRMIMRLWRQGTGVRMSKESQRGRKRVTRLVIVVVVIAFASLWLPVQL-------       279
Elegans           IISGLYMRMIMRLWRQGTGVRMSKESQRGRKRVTRLVIVVVVIAFASLWLPVQL-------       279
Erecta            IISGLYMRMIMRLWRQGTGVRMSKESQRGRKRVTRLVVVVVVIAFASLWLPVQL-------       274
Mauritania        IISGLYMRMIMRLWHQGTGVRMSKESQRGRKRVTRLVVVVVVIAFASLWLPVQL-------       274
Sechelia          IISGLYMRMIMRLWRQGTGVRMSKESQRGRKRVTRLVVVVVVIAFASLWLPVQL-------       274
Melanogaster      IISGLYMRMIMRLWRQGTGVRMSKESQRGRKRVTRLVVVVVVIAFASLWLPVQL-------       274
simulans          IISGLYMRMIMRLWRQGTGVRMSKESQRGRKRVTRLVVVVVVIAFASLWLPVQL-------       274
Suzuki            IISGLYMRMIMRLWHQGTGVRMSKESQRGRKRVTRLVVVVVVIAFASLWLPVQL-------       277
biarmipes         IISGLYMRMIMRLWHQGTGVRMSKESQRGRKRVTRLVVVVVVIAFASLWLPVQL-------       277
                  :*****:*******:**:*******************:************:*:

Grimshawi         --------------ILLLKALNIYVANSMLSVILQIVAQTMAYTSSSINPLLYAFLSDNF       333
Virilis           --------------ILLLKALDMYEINSMFNVILQIVAHTMAYTSSCINPLLYAFLSDNF       332
Mojavensis        --------------ILLLKALDLYETNNMFSVILQIVAHTMAYTSSCINPLLYAFLSDNF       328
Bipectinate       --------------ILLLKALNLYKADTLFKIILQISAQTLAYSSSCINPLLYAFLSDNF       322
Anannassae        --------------ILLLKALNLYQADTLFKVILQISAQTLAYSSSCINPLLYAFLSDNF       322
Serrata           --------------ILLLKSLGVIQINSLPKVVMQVSAQTLAYSSSCINPLLYAFLSDNF       324
Kikkawei          LSNTIKFHLNCPQLILLLKSLDIIQINSLPKVVMQVSAQTLAYSSSCINPLLYAFLSDNF       345
Eugracilis        --------------ILLLKSLEILTTDTLIKLIIQVAAQTLAYTSSCINPLLYAFLSENF       323
Ficusphila        --------------ILLLKALDVIEMNSLNKLIIQVSAQTLAYSSSCINPLLYAFLSENF       325
Takahashi         --------------ILLLKSLNVIETNTLSKLIIQVTAQTLAYSSSCINPLLYAFLSENF       319
Rhopaloa          --------------ILLFKALDVIEMNSLTKLVIQVTAQTLAYSSSCINPLLYAFLSENF       325
Elegans           --------------ILLFKALGVVEMNSLTKLVIQVTAQTLAYSSSCINPLLYAFLSENF       325
Erecta            --------------ILLLKSLDVIETNTLTKLVIQVTAQTLAYSSSCINPLLYAFLSENF       320
Mauritania        --------------ILLLKSLDIIETNTLTKLVIQVTAQTLAYSSSCINPLLYAFLSENF       320
Sechelia          --------------ILLLKSLDVIETNTLTKLVIQVTAQTLAYSSSCINPLLYAFLSENF       320
Melanogaster      --------------ILLLKSLDVIETNTLTKLVIQVTAQTLAYSSSCINPLLYAFLSENF       320
simulans          --------------ILLLKSLDVIETNTLTKLVIQVTAQTLAYSSSCINPLLYAFLSENF       320
Suzuki            --------------LLLFKSLGVIETNTLTKLIIQVTAQTLAYSSSCINPLLYAFLSENF       323
biarmipes         --------------ILLFKTLGVIETNTLTKLIIQVTAQTLAYSSSCINPLLYAFLSENF       323
                                :**:*:* :   :.: .:::*: *:*:**:**.**********:**

Grimshawi         RKAFHKAINCSNRYQDYTSDLPPPRKTSCGRTSTTGL   370
Virilis           RKAFYKAINCSNRYHNYTSDLPPPRKTSCGRTSTTGL   369
Mojavensis        RKAFYKAINCSNRYQNYTSDLPPPRKTSCGRTSTTGL   365
Bipectinate       RKAFYKAINCSSRYQNYTSDLPPPRKTSCARTSTTGL   359
Anannassae        RKAFYKAINCSSRYQNYTSDLPPPRKTSCARTSTTGL   359
Serrata           RKAFYKAINCSPRYQNYTSDLPPPRKTSCARTSTTGL   361
Kikkawei          RKAFYKAINCSPRYQNYTSDLPPPRKTSCARTSTTGL   382
```

```
Eugracilis         RKAFYKAINCSSRYQNYTSDLPPPRKTSCARTSTTGL  360
Ficusphila         RKAFYKAINCSSRYQNYTSDLPPPRKTSCARTSTTGL  362
Takahashi          RKAFYKAINCSSRYQNYTSDLPPPRKTSCARTSTTGL  356
Rhopaloa           RKAFYKAINCSSRYQNYTSDLPPPRKTSCARTSTTGL  362
Elegans            RKAFYKAINCSSRYQNYTSDLPPPRKTSCARTSTTGL  362
Erecta             RKAFYKAVNCSSRYQNYTSDLPPPRKTSCARTSTTGL  357
Mauritania         RKAFYKAVNCSSRYQNYTSDLPPPRKTSCARTSTTGL  357
Sechelia           RKAFFKAVNCSSRYQNYTSDLPPPRKTSCARTSTTGL  357
Melanogaster       RKAFYKAVNCSSRYQNYTSDLPPPRKTSCARTSTTGL  357
simulans           RKAFYKAVNCSSRYQNYTSDLPPPRKTSCARTSTTGL  357
Suzuki             RKAFYKAINCSSRYQNYTSDLPPPRKTSCGRTSTTGL  360
biarmipes          RKAFYKAINCSSRYQNYTSDLPPPRKTSCARTSTTGL  360
```

Melanogaster NP_001263042.1

```
  1 menttmlani slnatrneen itsfftdeew laingtlpwi vgfffgviai tgffgnllvi
 61 lvvvfnnnmr sttnlmivnl aaadlmfvil cipftatdym vyywpygrfw crsvqylivv
121 tafasiytlv lmsidrflav vhpirsrmmr tenitliaiv tlwivvlvvs vpvafthdvv
181 vdydakknit ygmctfttnd flgprtyqvt ffissyllpl miisglymrm imrlwrqgtg
241 vrmskesqrg rkrvtrlvvv vviafaslwl pvqlillllks ldvietntlt klviqvtaqt
301 layssscinp llyaflsenf rkafykavnc ssryqnytsd lpppkrktsca rtsttgl
```

Erecta XP_001981439.1

```
  1 mentallani slnatrneen itsfftdeew laisgtlpwi vgfffgviai tgffgnllvi
 61 lvvvfnnnmr sttnlmivnl aaadlmfvil cipftatdym vyywpygrfw crsvqylivv
121 tafasiytlv lmsidrflav vhpirsrmmr tenitliaiv tlwivvlvvs vpvafthdvv
181 vdydakknit ygmctftsne flsprtyqvt ffissyllpl miisglymrm imrlwrqgtg
241 vrmskesqrg rkrvtrlvvv vviafaslwl pvqlillllks ldvietntlt klviqvtaqt
301 layssscinp llyaflsenf rkafykavnc ssryqnytsd lppprktsca rtsttgl
```

Suzuki XP_016926809.2

```
  1 mdtmenstll gkislnasrs denitsfftd eewlaisgtl pwivvfffga iaitgffgnl
 61 lvilvvvfnk nmrsttnlmi vnlaaadlmf vilcipftat dymvyywpyg rfwcrsvqyl
121 ivvtafasiy tlvlmsidrf lavvhpirsr mmrtenitli aivtlwivvl vismpvffth
181 dvmvdtdakk nitygmctfa tndflgpkty qvtffissyl lplmiisgly mrmimrlwhq
241 gtgvrmskes qrgrkrvtrl vvvvviafas lwlpvqllll fkslgvietn tltkliiqvt
301 aqtlaysssc inpllyafls enfrkafyka incssryqny tsdlppprkt scgrtsttgl
```

Sechelia XP_002037108.1

```
  1 menttmlani slnatrneen itsfftdeew laingtlpwi vgfffgviai tgffgnllvi
 61 lvvvfnnnmr sttnlmivnl aaadlmfvil cipftatdym vyywpygrfw crsvqylivv
121 tafasiytlv lmsidrflav vhpirsrmmr tenitliaiv tlwivvlvvs vpvafthdvv
181 vdydakknit ygmctfttnd flsprtyqvt ffissyllpl miisglymrm imrlwrqgtg
241 vrmskesqrg rkrvtrlvvv vviafaslwl pvqlillllks ldvietntlt klviqvtaqt
301 layssscinp llyaflsenf rkaffkavnc ssryqnytsd lppprktsca rtsttgl
```

simulans XP_002105245.1

```
  1 menttmlani slnatrneen itsfftdeew laingtlpwi vgfffgviai tgffgnllvi
 61 lvvvfnnnmr sttnlmivnl aaadlmfvil cipftatdym vyywpygrfw crsvqylivv
121 tafasiytlv lmsidrflav vhpirsrmmr tenitliaiv tlwivvlvvs vpvafthdvv
181 vdydakknit ygmctfttnd flsprtyqvt ffissyllpl miisglymrm imrlwrqgtg
241 vrmskesqrg rkrvtrlvvv vviafaslwl pvqlillllks ldvietntlt klviqvtaqt
301 layssscinp llyaflsenf rkafykavnc ssryqnytsd lppprktsca rtsttgl
```

biarmipes XP_016950481.1

```
  1 mdtmdnatll gkislnasrn denitsfftd eewlaingtl pwivafffga iaitgffgnl
 61 lvilvvvfnk nmrsttnlmi vnlaaadlmf vvlcipftat dymvyywpyg rfwcrsvqyl
121 ivvtafasiy tlvlmsidrf lavvhpirsr mmrtenitli aivtlwivvl vvsmpvffth
181 dvmedtdakk nitygmctfa tndflgpkty qvtffvssyl lplmiisgly mrmimrlwhq
241 gtgvrmskes qrgrkrvtrl vvvvviafas lwlpvqlill fktlgvietn tltkliiqvt
301 aqtlaysssc inpllyafls enfrkafyka incssryqny tsdlppprkt scartsttgl
```

Takahashi XP_017006243.1

```
  1 menatimlnk inssrmeens tsyftdeewl sisstlpwiv cfffgviait gffgnllvil
 61 vvvfnknmrs ttnlmivnla aadlmfvilc ipftatdymv yywpygvfwc rsvqylivvt
121 afasiytlvl msidrflavv hpirsrmmrt enitliaivt lwivvlvvsl pvffahdvmv
181 qydakknity gmcqftendf mgpktyqvtf ftssyllplm iisglymrmi mrlwrqgtgv
241 rmskesqrgr krvtrlvvvv viafaslwlp vqlillllksl nvietntlsk liiqvtaqtl
```

301 ayssscinpl lyaflsenfr kafykaincs sryqnytsdl ppprktscar tsttgl

Bipectinate XP_017092876.2
1 mdnttlatpd platmatydd nltsffseee rqaiegtliw lvpfffgiia ltgffgnllv
61 ilvvvfnknm rsttnlmivn lavadllfvi fcipftatdy vtkvwpfglf wcrsvqyliv
121 vtafasiytl vlmsidrfla vvhpirsrmm rtenitmiai vtlwavilvv stpvgfvndm
181 vvlydnktni tyvactydag nellsprsfq isffissyll plmiisglyv rmimrlwhqg
241 sgvrmskesq rgrkrvtrlv vvvviafasl wlpvqlilll kalnlykadt lfkiilqisa
301 qtlayssscі npllyaflsd nfrkafykai ncssryqnyt sdlppprkts cartsttgl

Rhopaloa XP_016982221.1
1 mdnitvspae mlgnisinas rndenltsff tdeewlaing tlpwivgfff gaiaitgffg
61 nllvilvvvf nknmrsttnl mivnlaaadl mfvilcipft atdymvyywp fgrfwcrsvq
121 ylivvtafas iytlvlmsid rflavvhpir srmlrtenit liaivtlwiv vlvvsmpvaf
181 thdvvvdyda kknitygmcm ftnndflgsr tyqvtffiss ymlplmiisg lymrmimrlw
241 rqgtgvrmsk esqrgrkrvt rlvivvviaf aslwlpvqli llfkaldvie mnsltklviq
301 vtaqtlayss scinpllyaf lsenfrkafy kaincssryq nytsdlpppr ktscartstt
361 gl

Eugracilis XP_017083059.1
1 menmdnstmv vdlvvnstrn denmtsfftd eewvairgtl pwiviflfgv iaitgllgnl
61 lvilvvvfnk nmrsttnlmi vnlaaadllf vilcipftat dymvyywpyg rfwcrsvqyl
121 ivvtafasiy tlvlmsidrf lavvhpirsr mmrtenitli aivtlwivvl iisvpvalth
181 dvvvdhdvkk nitygmctfk pndyvteksy hviffitsyl lplmiisgly mrmimrlwrq
241 gtgvrmskes qrgrkrvtrl vvvvviafas lwlpiqlill lksleilttd tlikliiqva
301 aqtlaytssc inpllyafls enfrkafyka incssryqny tsdlppprkt scartsttgl

Ficusphila XP_017038669.1
1 menatffpak mlgslasnat rndenltttff tdeewlains tlpwvvgfff gaiaitgffg
61 nllvilvvvf nksmrsttnl mivnlavadl lfvilcipft atdymvpywp ygrfwchsvq
121 ylivvtafas iytlvlmsid rflavvhpir srmmrtenit liaivtlwiv vlvvsvpval
181 nhdvveaide kknityglct yspneflssr tyhvtffvss ylmplmvisg lymrmimrlw
241 rqgtgvrmsk esqrgrkrvt rlvvvvviaf aslwlpvqli lllkaldvie mnslnkliiq
301 vsaqtlayss scinpllyaf lsenfrkafy kaincssryq nytsdlpppr ktscartstt
361 gl

Anannassae XP_001955066.1
1 mdnttlatse llatmatydd nftsffseee rqaiegtliw lvpfffgiia ltgffgnllv
61 ilvvvfnknm rsttnlmivn lavadllfvi fcipftatdy vtkvwpfglf wcrsvqyliv
121 vtafasiytl vlmsidrfla vvhpirsrmm rtenitmiai vtlwavilvv stpvgfvndm
181 vvsydnktni tyvlctfdag nellsprsfq isffvssyll plmiisglyv rmimrlwhqg
241 sgvrmskesq rgrkrvtrlv vvvviafasl wlpvqlilll kalnlyqadt lfkvilqisa
301 qtlayssscі npllyaflsd nfrkafykai ncssryqnyt sdlppprkts cartsttgl

Serrata XP_020809023.1
1 menttliage tlanislani tqsdenitsf fteeewlalt gilpwlvgft fgaiaitgfl
61 gnllvilvvv fnnnmrsttn lmivnlaaad llfvilcipf taadyvtdyw pfggiwcrcv
121 qylivvtafa siytlvlmsi drflavvhpi rsrmmrteni tmvaivtlwv vilvvsvpva
181 findfavmsd gkrslgmctf kpndyisprt fqvsffissy llplmiisgl yvrmimrlwh
241 qgsgvrmske sqrgrkrvtr lvvvvviafa slwlpiqlil llkslgviqi nslpkvvmqv
301 saqtlaysss cinpllyafl sdnfrkafyk aincspryqn ytsdlppprk tscartsttg
361 l

Kikkawei KAH8347379.1
menttlitge tlanislant trsdenitsf fteeewlalt gilpwlvgft fgaiaitgfl
61 gnllvilvvv fnnnmrsttn lmivnlavad llfvilcipf taadyvtdyw pfggfwcrsv
121 qylivvtafa siytlvlmsi drflavvhpi rsrmmrteni tmvaivtlwv vilvvsvpva
181 findfavmsd gkrsigmctf kandyisprt fqvsffissy llplmiisgl yvrmimrlwh
241 qgsgvrmske sqrgrkrvtr lvvvvviafa slwlpiqvsi lfvhslsnti kfhlncpqli
301 lllksldiiq inslpkvvmq vsaqtlayss scinpllyaf lsdnfrkafy kaincspryq
361 nytsdlpppr ktscartstt gl

Mauritania XP_033166501.1
1 menttmlani slnatrneen itsfftdeew laingtlpwi vgfffgviai tgffgnllvi
61 lvvvfnnnmr sttnlmivnl aaadlmfvil cipftatdym vyywpygrfw crsvqylivv

```
121 tafasiytlv lmsidrflav vhpirsrmmr tenitliaiv tlwivvlvvs vpvafthdvv
181 vdydakknit ygmctfttnd flsprtyqvt ffissyllpl miisglymrm imrlwhqgtg
241 vrmskesqrg rkrvtrlvvv vviafaslwl pvqlillllks ldiietntlt klviqvtaqt
301 layssscinp llyaflsenf rkafykavnc ssryqnytsd lppprktsca rtsttgl
```

Elegans **XP_017131735.1**

```
  1 mdnitesaae mlanislnas rndenltsff tdeewlaisg tlpwivgfff gaiaitgffg
 61 nllvilvvvf nknmrsttnl mivnlaaadl mfvilcipft atdymvyywp fgrfwcrsvq
121 ylivvtafas iytlvlmsid rflavvhpir srmmrtenit liaivtlwiv vlvvsvpvaf
181 thdvvvesda kknitygmcm fitndimdsr tyhvtffiss ymlplmiisg lymrmimrlw
241 rqgtgvrmsk esqrgrkrvt rlvivvviaf aslwlpvqli llfkalgvve mnsltklviq
301 vtaqtlayss scinpllyaf lsenfrkafy kaincssryq nytsdlpppr ktscartstt
361 gl
```

Grimshawi **XP_001995830.3**

```
  1 mnqsyimenn tpsmsnsswl elklttnnst gnsslsldae eeeairqtvr wlvplffgii
 61 aisgfffgnl lvilvvllnk nmhsttnlli vnlaaadllf vifcvpftav dyvadhwpfg
121 tiwcrivqyl ivvtayasiy tlvlmsidrf lavvhpirsr mlrtehitki aiftlwtvvl
181 tvsmpvtfth dlvvhhnykt nstyakchfi endlldwltf qvsffissyl lplmvisgly
241 vrmimrlwrq gsgvrmskes qrgrkrvtrl vvvvviafas lwlpvqlill lkalniyvan
301 smlsvilqiv aqtmaytsss inpllyafls dnfrkafhka incsnryqdy tsdlppprkt
361 scgrtsttgl
```

Virilis **XP_002053983.1**

```
  1 mnlsnitltl psnsswlesq lelttassnl dnstlssfya eaeairatvr wvvpfffgii
 61 aisgffgnll vilvvllnkn mhsttnlliv nlaaadllfv ifcvpftaid yvtqhwpfgk
121 mwcrsvqyli vvtayasiyt lvlmsidrfl avvhpirsrm lrtehitkia iftlwtvvlt
181 vsmpvtfahd vvvdydnqtn vtyamcryid ndvldlstfq vsffissyll plmvisglyv
241 rmimrlwhqg tgvrmskesq rgrkrvtrlv vvvviafasl wlpvqlilll kaldmyeins
301 mfnvilqiva htmaytssci npllyaflsd nfrkafykai ncsnryhnyt sdlppprkts
361 cgrtsttgl
```

Mojavensis **XP_002000968.1**

```
  1 melsnltttl atqlhqlats slnhsigpdn stalqseeea iratvrwlvp iffgiiavsg
 61 ffgnllvilv vllnknmhst tnllivnlaa adllfvifcv pftavdyvtq hwpfgkiwcr
121 svqylivvta yasiytlvlm sidrflavvh pirsrmlrte hitkiaiftl wtvvltvsmp
181 vtfahdvvvd ydnqtnvtya mcrfidnevl eqstfqvsff issyllplmv isglyvrmim
241 rlwhqgtgvr mskesqrgrk rvtrlvvvvv iafaslwlpv qlilllkald lyetnnmfsv
301 ilqivahtma ytsscinpll yaflsdnfrk afykaincsn ryqnytsdlp pprktscgrt
361 sttgl
```
